# Divergent Enantioselective Total Synthesis of (−)-Ajmalicine, (+)-Mayumbine, and (−)-Roxburghine C

**DOI:** 10.1021/acs.orglett.5c00715

**Published:** 2025-03-20

**Authors:** Vincent Goëlo, Qian Wang, Jieping Zhu

**Affiliations:** Laboratory of Synthesis and Natural Products (LSPN), Institute of Chemical Sciences and Engineering, Ecole Polytechnique Fédérale de Lausanne, EPFL-SB-ISIC-LSPN, BCH 5304, 1015 Lausanne, Switzerland

## Abstract

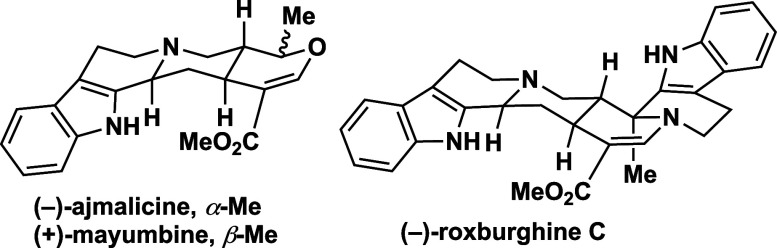

We report herein a divergent enantioselective total synthesis of (−)-ajmalicine, (+)-mayumbine, and (−)-roxburghine C. The synthesis employs Franzén’s organocatalytic reaction between *N*-acetoacetyl tryptamine and (*E*)-5-hydroxypent-2-enal to generate a functionalized pentacyclic compound with high diastereo- and enantioselectivity. This intermediate serves as a versatile platform for accessing the three heteroyohimbine alkaloids. Notably, a diastereoselective intramolecular Pictet–Spengler reaction of methyl ketone and chemoselective reduction of β-amidoester to β-enaminoester were exploited for the synthesis of (−)-roxburghine C.

Heteroyohimbines, a subfamily of monoterpene indole alkaloids, display a range of intriguing bioactivities.^1^ For instance, (−)-ajmalicine **1**,^2^ isolated from *Rauwolfia serpentina*, is marketed as an antihypertensive drug for the treatment of high blood pressure,^3^ while (+)-mayumbine, the 19-*epi*-ajmalicine **2**, is a ligand for the benzodiazepine receptor (Scheme 1a).^4^ These compounds feature four stereocenters, theoretically yielding 16 possible stereoisomers. However, only 8 stereoisomers have been reported to date, as the C15 stereocenter consistently retains an (*S*)-configuration in all known natural products. The total synthesis of these alkaloids has fascinated synthetic chemists for over half a century, inspiring the development of numerous elegant strategies, including enantioselective approaches.^5^

**Scheme 1:**
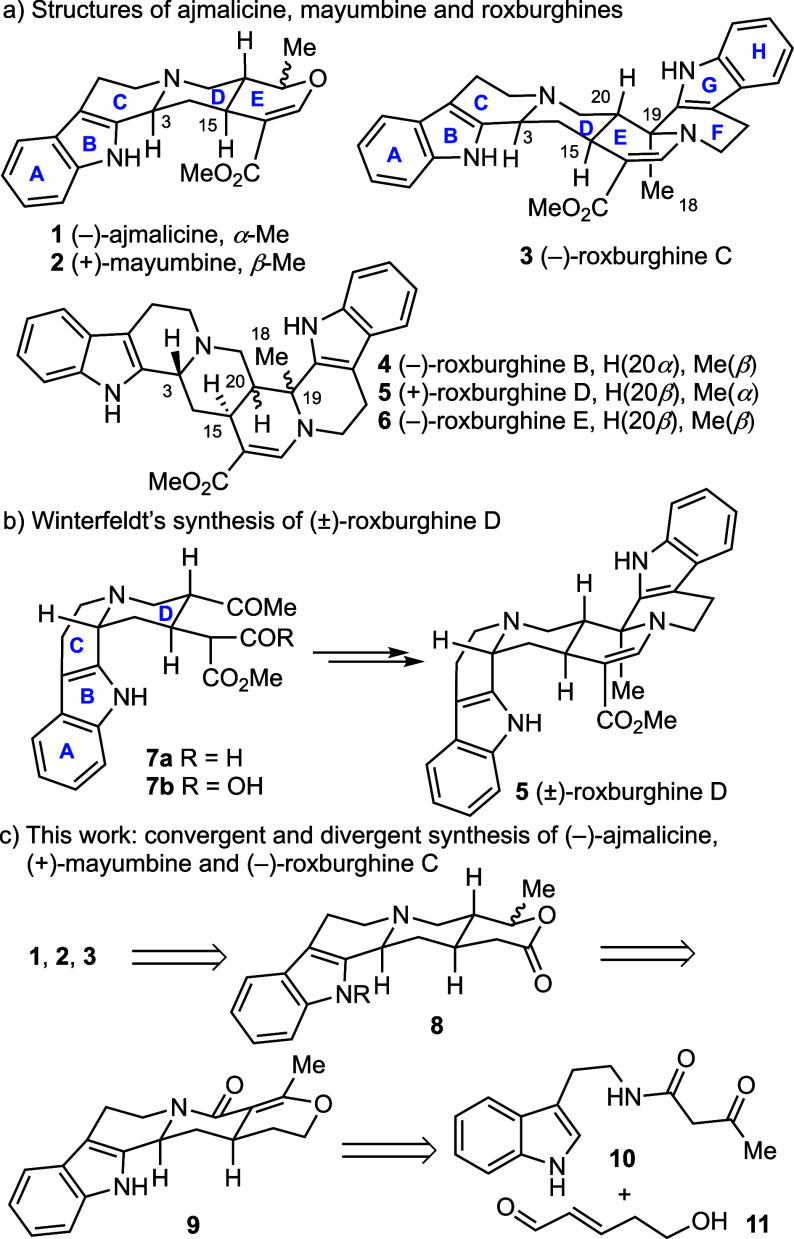
Ajmalicine, Mayumbine and Roxburghines: Divergent Synthesis of These Heteroyohimbines

Heteroyohimbines are structurally related to yohimbine, differing in the presence of a heteroatom in the E ring of their pentacyclic framework. In most cases, the E ring of this class of indole alkaloids contains an oxygen atom, consistent with their biosynthetic origin.^6^ However, polycyclic heteroyohimbines featuring a nitrogen atom in the E ring have also been isolated. For example, roxburghines B–E (**3**–**6**), octacyclic compounds composed of two tryptamine units and one monoterpenic moiety, have been isolated from *Uncaria elliptica* plants. Extracts of these plants are used in traditional medicine to treat inflammation.^7^ While the absolute configurations at C3, C19 and C20 vary among the roxburghines, all four share an (*S*)-configuration at C15, in line with all other members of this subfamily of indole alkaloids (Scheme 1a). Notably, (−)-roxburghine C **3** shares the same stereochemistry at C3, C15 and C20 as (−)-ajmalicine **1**, (+)-mayumbine **2**.

Merlini proposed that a C19-*oxo*-derivative of geissoschizine **7a** (R = H) serves as the biogenetic precursor of these alkaloids.^7a^ Building on this hypothesis, Winterfeldt accomplished the first synthesis of (±)-roxburghine D **5** from the carboxylic acid **7b** (R = OH, Scheme 1b).^8^ To date, this remains the only synthetic effort targeting these bisindolic alkaloids.

In line with our ongoing interest in developing divergent synthesis of indole alkaloids,^9^ including bisindolic natural products,^10^ we sought to establish a unified strategy for accessing (−)-ajmalicine **1**, (+)-mayumbine **2** and (−)-roxburghine C **3**. Central to our approach is pentacyclic lactone **8**, envisaged as a common intermediate for these targeted natural products. Compound **8** could be synthesized from **9** which in turn could be prepared in a single step from *N*-acetoacetyl tryptamine **(10)** and (*E*)-5-hydroxypent-2-enal (**11**). This elegant enantioselective transformation, pioneered by the groups of Franzén^11^ and Zhao,^12^ has been successfully applied to the total synthesis of corynantheine type alkaloids,^13^*epi*-geissoschizol,^14^ and spirooxindole alkaloid.^15^ We report herein the realization of this strategy allowing the access to these natural products in a short, divergent, and enantioselective manner. Notably, a diastereoselective intramolecular Pictet–Spengler reaction of methyl alkyl ketone and chemoselective reduction of β-amidoester to β-enaminoester were exploited for the synthesis of (−)-roxburghine C **3**.

The synthesis of pentacyclic lactone **8** is outlined in Scheme 2. Heating a *p*-xylene solution of tryptamine **12** and 2,2,6-trimethyl-4*H*-1,3-dioxin-4-one (**13**) at 130 °C for 45 min afforded the *N*-acetoacetyl tryptamine **(10**) in 84% yield.^16^ Cross-metathesis of but-3-en-1-ol (**14**) with crotonaldehyde (**15**) in the presence of a Grubbs II catalyst provided (*E*)-5-hydroxypent-2-enal (**11**) in 67% yield. Following Franzén’s procedure, stirring a dichloromethane solution of **10** and **11** with a catalytic amount of Hayashi–Jørgensen catalyst **16** (20 mol %)^17^ at −20 °C followed by addition of AcCl (10 equiv, rt) afforded the pentacyclic compound (3*S*, 15*R*)-**9** (97% *ee*, dr >20:1) which, without purification, was converted to *N*-Cbz derivative **17** in 72% isolated yield. Selective reduction of the amide **17** to tertiary amine **18** was realized by treatment of **17** with Meerwein’s salt followed by reduction of the resulting tetrafluoroborate salt of the imino ether with NaBH_4_.^18^ Treating the crude tertiary amine **18** with 2 N HCl in THF at room temperature afforded a single (3*S*, 15*R*, 20*R*)-diastereomer resulting from the highly diastereoselective hydration of the enol ether function. Reduction of the crude hydroxy ketone by NaBH_4_ afforded 1,5-diol **19** as a 3:1 mixture of two diastereoisomers in 76% yield from **17**. The absolute and relative stereochemistry of major isomer **19a** was determined by X-ray crystallographic analysis. Selective oxidation of 1,5-diol to δ-lactone proved challenging. After evaluating various oxidation conditions (PCC, Fétizon’s reagent, Swern oxidation, Cu-nitroxyl-air,^19^ etc.), the desired lactone **8** was isolated using either RuCl_2_(PPh_3_)_3_ (52% yield)^20^ or Ley oxidation [TPAP (cat.), NMO, molecular sieves, 70% yield],^21^ with the keto-aldehyde as the only byproduct (<10%). Using Ley oxidation, the full sequence from **17** was performed on gram-scale yielding the desired lactone **8** as two separable diastereoisomers (dr 3:1) in 57% yield, without requiring column chromatographic purification of the intermediates **18** and **19**.

**Scheme 2:**
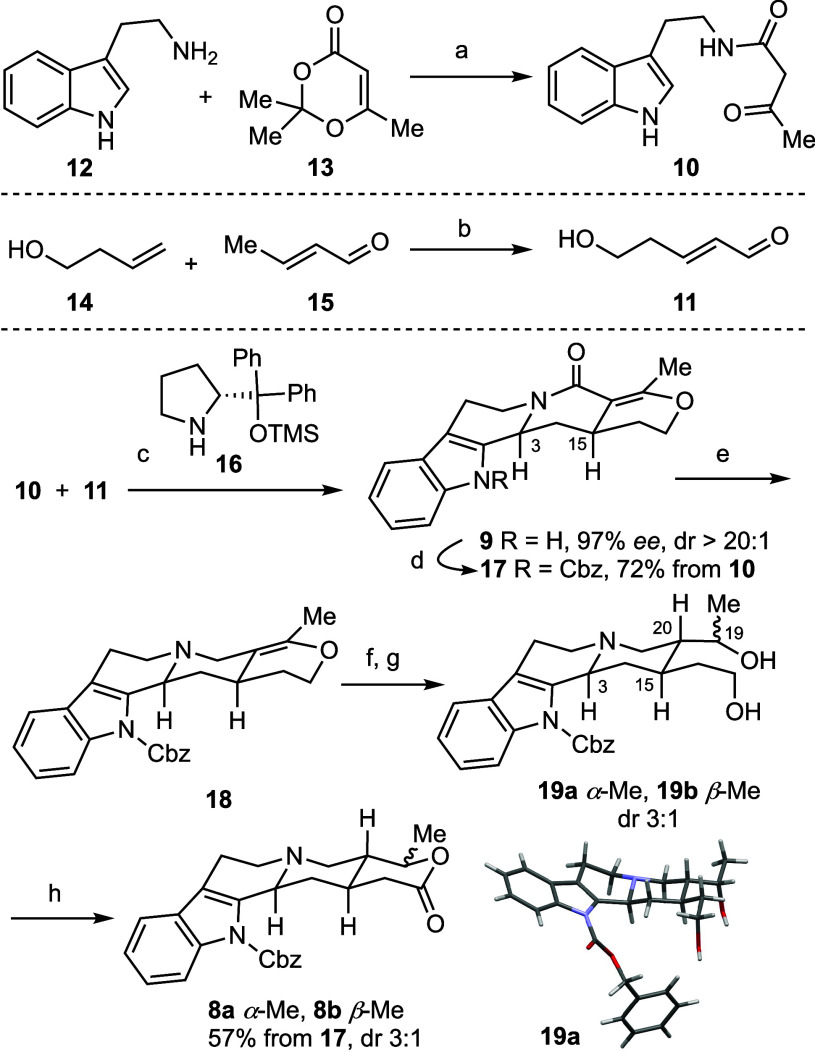
Enantioselective Synthesis of Pentacyclic Lactone **8** Reagents and conditions: a) *p*-xylene, 130 °C, 45 min, 84%; b) Grubbs II (0.8 mol %), crotonaldehyde (4.0 equiv), DCM, reflux, 24 h, 67%; c) (i) **11** (1.2 equiv), Hayashi–Jørgensen catalyst **16** (20 mol %), DCM, −20 °C, 12 h, (ii) AcCl (10.0 equiv), rt, 18 h, 97% *ee*, dr >20:1; d) NaH (60 wt %, 3.0 equiv), DMF, 0 °C, 1 h, then benzyl chloroformate (2.0 equiv), rt, 3 h, 72% over 2 steps; e) (i) Me_3_OBF_4_ (2.0 equiv), 2,6-di-*tert*-butyl-pyridine (3.0 equiv), 4 Å MS (1.0 g/mmol), DCM, rt, 6 h, (ii) NaBH_4_ (6.0 equiv), MeOH, 0 °C, 2 h; f) 2 N HCl/THF (v/v = 1:1), rt, 16 h; g) NaBH_4_ (6.0 equiv), MeOH, 0 °C, 2 h, **19a**/**19b** = 3:1, 76% over 3 steps; h) TPAP (30 mol %), NMO (3.0 equiv), 4 Å MS (3.0 g/mmol), DCM, rt, 2 h, 70% (57% over 4 steps from **17**).

Total syntheses of (−)-ajmalicine **1** and (+)-mayumbine **2** were accomplished from lactones **8a** and **8b**, respectively, as outlined in Scheme 3. Deprotonation of **8a** with LHMDS followed by enolate trapping with dimethyl carbonate yielded the β-ketoester, which was subsequently treated with sodium methoxide to afford *N*-unprotected pentacyclic lactone **20** in 75% yield (Scheme 3a). Although inconsequential, **20** was obtained as a single diastereomer, likely formed under thermodynamic control. Selective reduction of lactone **20** to lactol followed by dehydration yielded (−)-ajmalicine **1** (57% yield).^5p^ Its absolute and relative stereochemistry was confirmed by X-ray crystallographic analysis. Following the same synthetic route, compound **8b** was transformed to (+)-mayumbine **2** in 40% overall yield (Scheme 3b).

**Scheme 3:**
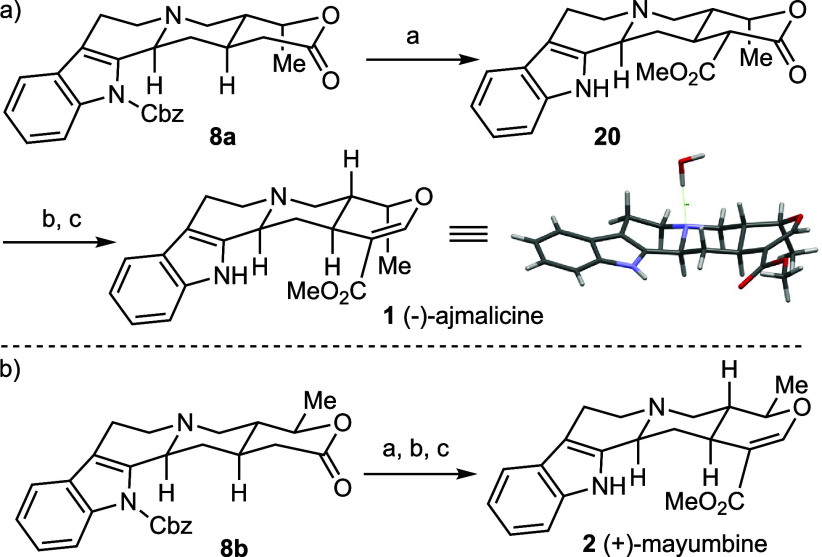
Total Synthesis of (−)-Ajmalicine **1** and (+)-Mayumbine **2** Reagents and conditions: a) LHMDS (4.0 equiv), −78 °C, 1 h, dimethyl carbonate (10.0 equiv), rt, 2 h, then NaOMe (5.4 N in MeOH) (5.0 equiv), rt, 30 min, 75%, dr >20:1; b) NaBH_4_ (5.0 equiv), DCM/MeOH, −10 °C, 1 h; c) *p*TSA·H_2_O (5.0 equiv), DCM, reflux, 8 h, 57% over 2 steps.

The total synthesis of (−)-roxburghine C **3** is summarized in Scheme 4. The AlMe_3_-mediated aminolysis of lactone **8** with *N*_*1*_-Boc tryptamine **21** (DCM, rt, 16 h) yielded **22** in 73% yield.^22^ Oxidation of the secondary alcohol **22** cleanly afforded the δ-ketoamide **23** which was used, without purification, in the subsequent one-pot *N*-Boc deprotection/intramolecular Pictet–Spengler reaction. Gratifyingly, stirring a dichloromethane solution of crude **23** in the presence of trifluoroacetic acid (40.0 equiv) afforded the octacyclic compound **24** in 68% yield with excellent diastereoselectivity (dr >20:1). Subsequent methoxycarbonylation of **24** (LTMP, dimethyl carbonate) followed by removal of the *N*-Cbz group (NaOMe) afforded the β-ketoester **25** in 75% yield as a single diastereoisomer. The absolute configuration of the newly formed stereocenter at C19 was determined to be (*S*) by X-ray crystallographic analysis of **25**, confirming the (3*S*, 15*S*, 20*R*, 19*S*) stereochemistry, which matches that of (−)-roxburghine C **3**. Conversion of β-amido ester to the corresponding β-enamino ester was realized following Ye-Huang’s procedure.^23^ Specifically, reduction of **25** using Vaska’s catalyst^24^ and tetramethyldisiloxane (TMDS) in dichloromethane afforded (−)-roxburghine C **3** in 91% isolated yield. Approximately 200 mg of this natural product was prepared in a single batch from lactone **8** in 34% yield and 14% overall yield from the starting material, *N*-acetoacetyl tryptamine **(10)**.

**Scheme 4:**
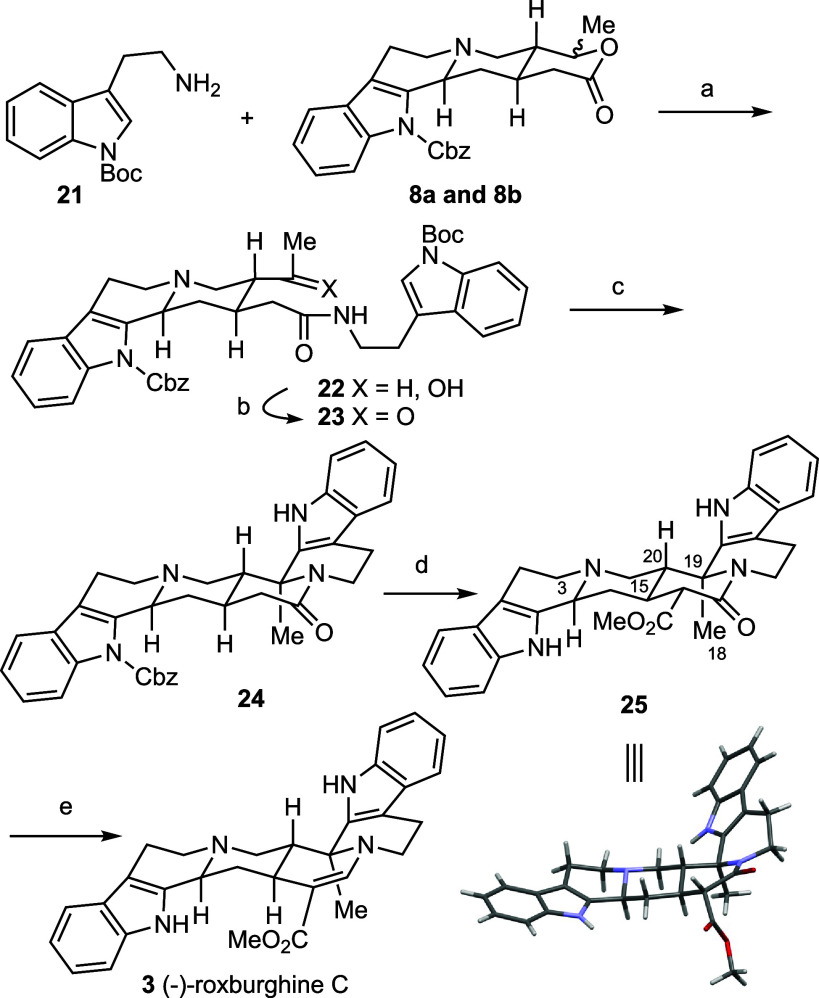
Total Synthesis of (−)-Roxburghine C **3** Reagents and conditions: a) **21** (4.0 equiv), AlMe_3_ (2.0 equiv), toluene, DCM, then **8**, rt, 16 h, 73%; b) IBX (5.0 equiv), THF-DMSO (v/v = 1:1), rt, 2 h; c) TFA (40.0 equiv), DCM, rt, 16 h, 68% over 2 steps, dr >20:1; d) (i) LiTMP (8.0 equiv), THF, −78 °C, 1 h, (ii) dimethyl carbonate (20.0 equiv), −78 °C, 1 h, rt, 2 h, (iii) then NaOMe (5.4 M MeOH) (8.0 equiv), rt, 30 min, 75%; e) Ir(CO)Cl(PPh_3_)_2_ (5.0 mol %), TMDS (20.0 equiv), DCM, rt, 4 h, 91%.

In Winterfeldt’s total synthesis of (±)-roxburghine D, the Pictet–Spengler reaction (PSR) of **26** exhibits high diastereoselectivity, which can be rationalized through conformational analysis. Specifically, the *cis*-fused C/D ring constrain compound **26** into an L-like conformation (Scheme 5a). Consequently, the *si*-face of *N*-acylketiminium **27** is sterically blocked by the A–B–C ring, favoring nucleophilic addition of the indole ring from the *re*-face, ultimately leading to observed product **28**. In contrast, the high diastereoselectivity observed in the PSR of **29** was unexpected at the outset of this study. The steric effects between the two faces of *N*-acylketiminium (**31A** and **31B**) are not immediately obvious unless one assumed that the *N*-CBz group on the B-ring disfavors the *si*-face attack through intermediate **31B** (Scheme 5b). To verify this hypothesis, compound **30** (R = H) was synthesized (*cf*Supporting Information) and submitted to the PSR under identical conditions (TFA, DCM, rt). However, diastereoisomer **33A**, resulting from the *re*-face attack of the indole to the *N*-acylketiminium, was also formed with excellent diastereoselectivity (65%, dr >20:1). Interestingly, we found that the diastereoselectivity of the PSR of **29** is condition-dependent. Stirring a toluene solution of **29** with TMSCl (50.0 equiv) at −30 °C for 24 h afforded a 1:1 mixture of compounds **24** and **24B** (95%). Under these conditions, compound **30** was similarly converted to **33A** and **33B** (dr = 1:1) in 97% yield. Remarkably, when the reaction was conducted at room temperature, high diastereoselectivity was restored (dr >20:1), albeit with a moderated yield. In contrast, performing the reaction at −40 °C resulted in low conversion and a 1:1 mixture of the two diastereoisomers.

**Scheme 5:**
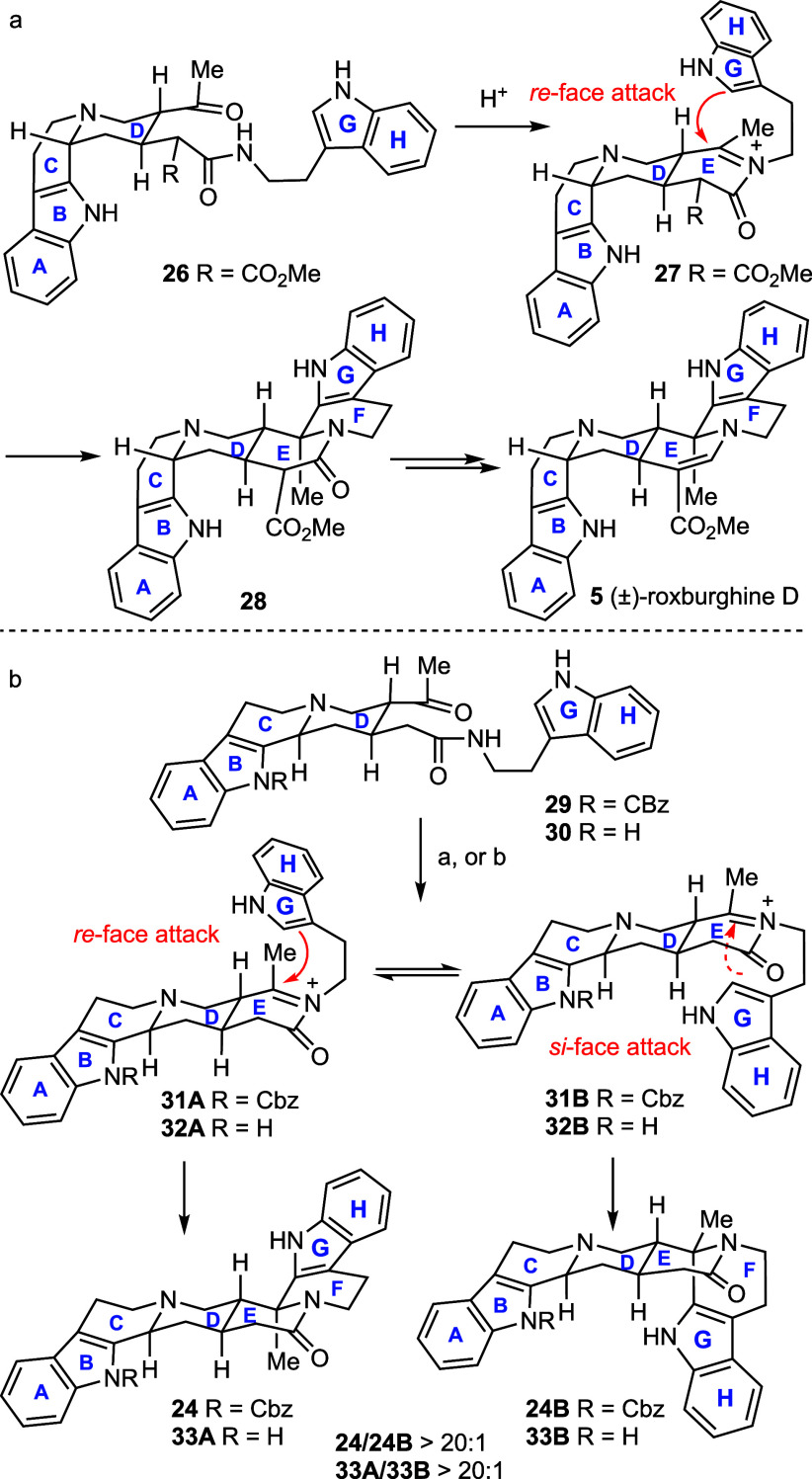
Stereoselectivity of the Pictet–Spengler Reaction Reagents and conditions: a) TFA (40 equiv), DCM, rt, **24**, 68%; **33A**, 65%; b) TMSCl (50.0 equiv), toluene, −30 °C, 24 h, 95%, **24**/**24B** = 1:1; 97%, **33A**/**33B** = 1:1.

Without detailed mechanistic studies, rationalizing the observed high diastereoselectivity for the reaction performed in TFA at room temperature is challenging. One possible explanation is that, in this Pictet–Spengler reaction, the rate-determining step is the rearomatization via deprotonation of pentahydro-β-carbolinium ion intermediate, and that the C–C bond formation is reversible, ultimately favoring the formation of observed products following the Curtin–Hammett principle (**29** to **24** and **30** to **33A**). This unconventional mechanistic scenario has been observed in the catalytic enantioselective PSRs of aldehydes^25^ and ketones.^26^

In summary, we have accomplished the divergent total syntheses of (−)-ajmalicine **1**, (+)-mayumbine **2**, and (−)-roxburghine C **3** using pentacyclic lactone **8** as a common intermediate. The synthesis hinges on the highly diastereo- and enantioselective construction of pentacyclic compound **9** from *N*-acetoacetyl tryptamine (**10**) and (*E*)-5-hydroxypent-2-enal (**11**), a transformation pioneered by Franzén. Notably, a diastereoselective intramolecular Pictet–Spengler reaction of methyl alkyl ketone and a chemoselective reduction of β-amidoester to β-enaminoester were exploited to achieve the total synthesis of (−)-roxburghine C **3**.

## Data Availability

The data underlying this study are available in the published article and its Supporting Information.

## Supporting Information Available

Experimental procedures and characterization data, additional experimental details, crystallographic data of **1**, **19a**, **25**, copies of ^1^H and ^13^C NMR spectra (PDF)
