# Supplementary material for: Divergent Enantioselective Total Synthesis of (−)-Ajmalicine, (+)-Mayumbine, and (−)-Roxburghine C
Source: Org Lett. 2025 Mar 20;27(13):3326–31. doi: 10.1021/acs.orglett.5c00715 (PMC11976861; doi:10.1021/acs.orglett.5c00715)
Supplement: Supplementary file 1 — ol5c00715_si_001.pdf [file ol5c00715_si_001.pdf]

Supporting information

Divergent Enantioselective Total Synthesis of (–)-Ajmalicine, (+)-Mayumbine and (–)-Roxburghine C

Vincent Goëlo, Qian Wang, and Jieping Zhu*

Laboratory of Synthesis and Natural Products (LSPN), Institute of Chemical Sciences and Engineering, Ecole Polytechnique Fédérale de Lausanne, EPFL-SB-ISIC-LSPN, BCH 5304, 1015 Lausanne (Switzerland)

*Correspondence to: jieping.zhu@epfl.ch

TABLE OF CONTENTS

1) General information 2

2) Experimental procedures and characterization data 3
   a) Lactone 8 synthesis 3
   b) (-)-Ajmalicine and (+)-mayumbine total synthesis 13
   c) (-)-Roxburghine C total synthesis 24
   d) Pictet-Spengler reaction selectivity 35

3) References 42

4) Copies of NMR spectra 43

5) X-Ray crystallographic data 93

1) General information

Reagents and solvents were purchased from commercial sources and preserved under argon. More sensitive compounds were stored in a desiccator or in the glovebox if required. Reagents were used as received without further purification unless otherwise noted. All reactions were performed under nitrogen (or argon) and stirring unless otherwise noted. When needed, glassware was dried overnight in an oven (150 °C). Solvents indicated as dry were either purchased as such, distilled prior to use, or dried by a passage through a column of anhydrous alumina or copper using a Puresolv MD 5 from Innovative Technology Inc., based on the Grubbs' design. Molecular sieves were activated heated under vacuum in an oven at 200 °C for at least 24 h. Flash column chromatography was performed using Silicycle SiliaFlash® P60 230-400 mesh. Reactions were monitored using Merck Kieselgel 60F254 aluminum. TLC's were revealed by UV fluorescence (254 nm) then with either $KMnO_4$ or phosphomolybdic acid. NMR spectra were recorded on AV2 400 MHz, AV2 600 MHz or AV2 800 MHz Brüker spectrometers at room temperature. ^{1}H frequency is at 400.13 MHz, ^{13}C frequency is at 100.62 MHz. Chemical shifts (δ) were reported in parts per million (ppm) relative to residual solvent peaks rounded to the nearest 0.01 for proton and 0.1 for carbon (ref: $CDCl_3$ [^{1}H: 7.26, ^{13}C: 77.16 ppm]; Acetone-d_6 [^{1}H: 2.05, ^{13}C: 29.84 and 206.26 ppm]).). Coupling constants (*J*) were reported in Hz to the nearest 0.1 Hz. Peak multiplicity was indicated as follows: s (singlet), d (doublet), t (triplet), q (quartet), p (quintet), m (multiplet) and br (broad). Attribution of peaks was done using the multiplicities and integrals of the peaks. COSY, HSQC, HMBC and NOESY experiments were used when needed to confirm the attribution. IR spectra were recorded in a Jasco FT/IR-4X spectrometer outfitted with a PIKE technology MIRacleTM ATR accessory as neat films compressed onto a Zinc Selenide window. The spectra are reported in cm^{-1}. Abbreviations used are: w (weak), m (medium), s (strong) and br (broad). The accurate masses were measured by the mass spectrometry service of the EPFL by ESI-TOF using a QTOF Ultima from Waters. Melting points were determined using a Stuart SMP30. Specific optical rotations $[\alpha]_D$ were measured with a Jasco P - 2000 polarimeter (589 nm). Enantiomeric excesses were determined with a 1260 Infinity II SFC System from Agilent using chiral stationary phase columns by comparing the samples with the corresponding racemic samples, column and elution details specified in each entry.

List of Abbreviations:

TLC – thin-layer chromatography; FCC – flash column chromatography.

2) Experimental procedures and characterization data

a) Lactone 8 synthesis

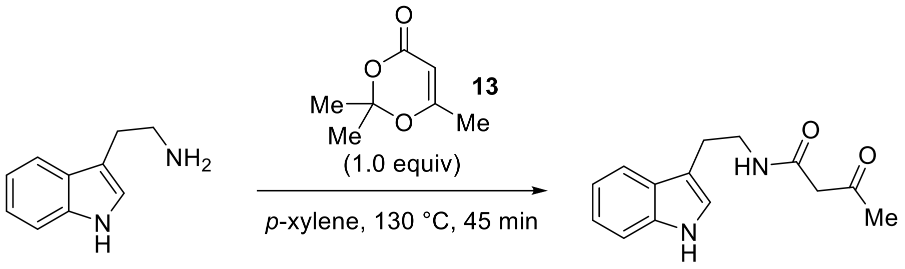

A solution of tryptamine **12** (4.0 g, 25 mmol, 1.0 equiv) and 2,2,6-trimethyl-4*H*-1,3-dioxin-4-one **13** (3.3 mL, 25 mmol, 1.0 equiv) in *p*-xylene (6.3 mL, 4.0 M) was stirred at 130 °C for 45 min. The solvent was removed under reduced pressure. The crude product was then purified by FCC (SiO_2, 100% EtOAc) to give a yellow solid that was triturated three times with Et_2O to afford the pure desired product **10** (5.1 g, 20.9 mmol, 84% yield) as a beige powder. All characterization data were in full agreement with those reported in the literature.[1]

N-(2-(1*H*-indol-3-yl)ethyl)-3-oxobutanamide (10)

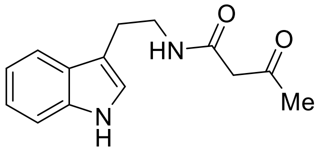

10

Beige powder.

Rf = 0.43 (EtOAc).

^{1}H NMR (400 MHz, $CDCl_3$) δ 8.12 (br s, 1H), 7.61 (d, *J* = 7.9 Hz, 1H), 7.37 (d, *J* = 8.1 Hz, 1H), 7.21 (t, *J* = 7.6 Hz, 1H), 7.13 (t, *J* = 7.5 Hz, 1H), 7.06 (d, *J* = 2.5 Hz, 1H), 6.89 (br s, 1H), 3.62 (q, *J* = 6.7 Hz, 2H), 3.35 (s, 2H), 2.99 (t, *J* = 6.8 Hz, 2H), 2.22 (s, 3H).

^{13}C NMR (101 MHz, $CDCl_3$) δ 204.7, 165.5, 136.5, 127.4, 122.3, 122.2, 119.6, 118.8, 113.0, 111.4, 49.9, 39.9, 31.2, 25.3.

HRMS (ESI/QTOF) m/z: $[M + Na]^+$ Calcd for $C_{14}H_{16}N_2NaO_2^+$ 267.1104; Found 267.1110.

IR (ν_{max}, cm^{-1}) 3395 (w), 3302 (m), 2923 (w), 1712 (m), 1644 (s), 1546 (m), 1456 (m), 1431 (m), 1357 (m), 1336 (m), 1227 (m), 1160 (m), 1094 (w), 909 (w), 742 (s).

M.p. = 78 – 79 °C.

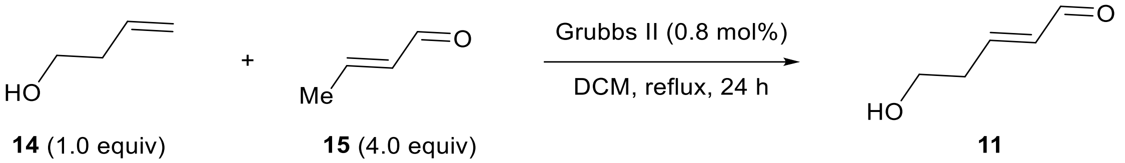

A solution of Grubbs II catalyst (204 mg, 240 µmol, 0.8 mol%) in dry and degassed DCM (3.0 mL) was added to a dry and degassed solution of but-3-en-1-ol **14** (2.6 mL, 30 mmol, 1.0 equiv) and crotonaldehyde **15** (9.9 mL, 120 mmol, 4.0 equiv) in DCM (12.0 mL, 2.5 M) at rt under Ar. The reaction mixture was reflux for 24 h. The crude mixture was purified by FCC (SiO_2, Et_2O/pentane 8/2 then 100% Et_2O to 9/1 Et_2O/acetone) to afford the desired conjugated aldehyde **11** (2.02 g, 20.2 mmol, 67% yield) as a colorless oil. All characterization data were in full agreement with those reported in the literature.[2]

(*E*)-5-hydroxypent-2-enal (11)

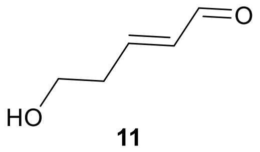

Colorless oil.

Rf = 0.3 (Et_2O).

^{1}H NMR (400 MHz, $CDCl_3$) δ 9.50 (d, *J* = 7.9 Hz, 1H), 6.90 (dt, *J* = 15.7, 6.8 Hz, 1H), 6.19 (ddt, *J* = 15.7, 7.8, 1.5 Hz, 1H), 3.83 (t, *J* = 6.2 Hz, 2H), 2.60 (qd, *J* = 6.3, 1.5 Hz, 2H), 2.14 (s, 1H).

^{13}C NMR (101 MHz, $CDCl_3$) δ 194.2, 155.1, 134.6, 60.8, 35.9.

HRMS (Sicrit plasma/LTQ-Orbitrap) m/z: $[M - H]^+$ Calcd for $C_5H_7O_2^+$ 99.0441; Found 99.0440.

IR (ν_{max}, cm^{-1}) 3374 (w), 2948 (w), 2882 (w), 2831 (w), 1675 (s), 1402 (w), 1137 (m), 1042 (m), 972 (s).

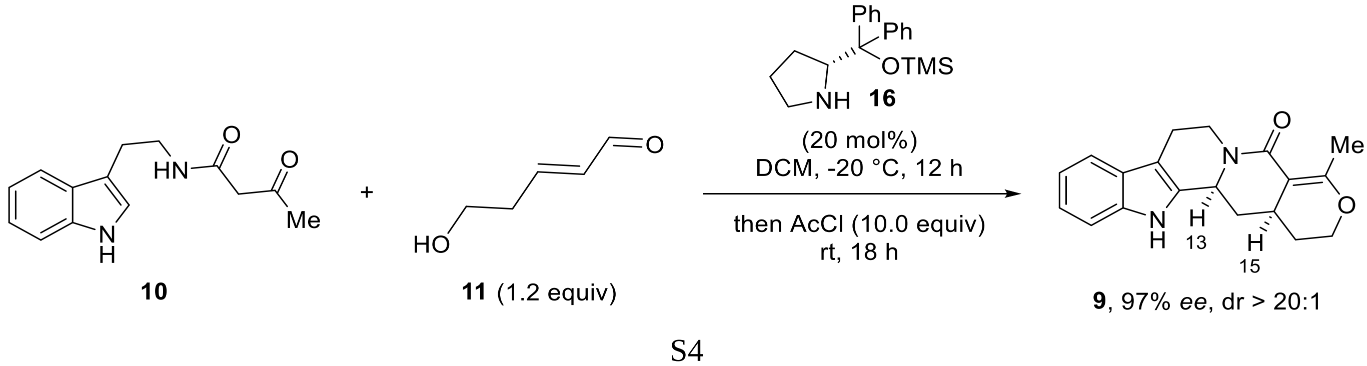

To a solution of β-ketoamide **10** (3.45 g, 14.1 mmol, 1.0 equiv) and freshly made (*R*)-Hayashi-Jørgensen organocatalyst **16** (920 mg, 2.8 mmol, 20 mol%) in dry DCM (14.1 mL, 1.0 M) was added the conjugated aldehyde **11** (1.7 g, 17.0 mmol, 1.2 equiv) at -20 °C. The reaction mixture was stirred at -20 °C for 12 h after which the reaction mixture completely solidified and TLC showed full conversion. The reaction mixture was allowed to warm to room temperature before AcCl (10.1 mL, 141 mmol, 10.0 equiv) dropwise addition to the reaction mixture. The orange mixture was stirred at rt for 18 h. A saturated aqueous solution of $NaHCO_3$ was then added carefully and the aqueous layer was extracted three times with DCM. The combined organic layers were washed with brine, dried over $MgSO_4$, filtered and evaporated *in vacuo*. The pentacyclic product **9** was obtained with 97% *ee* and a dr > 20:1 in favor of the H13/H15 *cis* isomer. The crude product was used in the subsequent step without further purification.

An analytical sample was obtained as a white solid by prep-TLC (3/7 acetone/hexane) for characterization and *ee* determination, that could be recrystallized from acetone/hexane by slow evaporation of the solvent. All characterization data were in full agreement with those reported in the literature.[1]

(13b*S*,14a*R*)-4-methyl-1,2,7,8,13,13b,14,14a-octahydro-5*H*-indolo[2,3-a]pyrano[3,4-g]quinolizin-5-one (9)

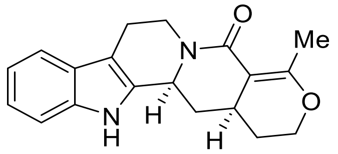

9

White crystal.

Rf = 0.28 (EtOAc).

^{1}H NMR (600 MHz, $CDCl_3$) δ 7.85 (s, 1H), 7.51 (d, *J* = 7.8 Hz, 1H), 7.32 (d, *J* = 8.0 Hz, 1H), 7.19 – 7.15 (m, 1H), 7.14 – 7.10 (m, 1H), 5.25 – 5.17 (m, 1H), 4.86 – 4.81 (m, 1H), 4.24 (ddd, *J* = 10.7, 4.0, 2.3 Hz, 1H), 3.93 (ddd, *J* = 12.6, 10.6, 2.0 Hz, 1H), 2.90-2.84 (m, 2H), 2.80 – 2.74 (m, 1H), 2.74 – 2.66 (m, 1H), 2.53 (ddd, *J* = 12.4, 4.7, 3.1 Hz, 1H), 2.33 (d, *J* = 1.5 Hz, 3H), 2.04 (ddt, *J* = 13.3, 6.0, 2.2 Hz, 1H), 1.64 – 1.56 (m, 1H), 1.54 (q, *J* = 12.3 Hz, 1H).

^{13}C NMR (151 MHz, $CDCl_3$) δ 165.0, 162.4, 136.4, 133.8, 127.1, 122.2, 120.0, 118.6, 111.0, 110.1, 103.8, 65.6, 53.6, 40.4, 35.6, 29.9, 29.2, 21.4, 20.6.

HRMS (ESI/QTOF) m/z: $[M + H]^+$ Calcd for $C_{19}H_{21}N_2O_2^+$ 309.1598; Found 309.1603.

IR (ν_{max}, cm^{-1}) 3257 (w), 2915 (w), 2848 (w), 1632 (m), 1570 (m), 1420 (m), 1301 (m), 1280 (m), 1254 (m), 1162 (m), 1066 (m), 906 (m), 821 (m), 728 (s), 696 (m), 647 (m).

M.p. = 255 – 257 °C (from white solid to yellow/brown and almost black when it melts).

$[\alpha]_D^{24}$ = -149 (*c* 0.24, $CHCl_3$).

SFC: IH column, 2 mL/min, 16% MeOH in supercritical CO_2, λ = 270.4 nm. Rt (major)= 3.8 min, Rt (minor) = 6.1 min.

SFC chromatogram of racemic compound 9:

**Data file:**	Run IH 16% MeOH 10 minutes.amx2023-10-13 18-24-24+02-00VG-2-099-A-rac.dx		
**Sequence Name:**	SFC-2023-10-13 18-22-08+02-00	**Project Name:**	Data
**Sample name:**	VG-2-099-A-rac	**Operator:**	SYSTEM
**Instrument:**	SFC	**Injection date:**	2023-10-13 18:25:05+02:00
**Inj. volume:**	2,000 µL	**Location:**	D1F-F1
**Acq. method:**	Run IH 16% MeOH 10 minutes.amx	**Type:**	Sample
**Processing method:**	*Manual Integration.pmx	**Column:**	IH-3
**Manually modified:**	Manual Integration		

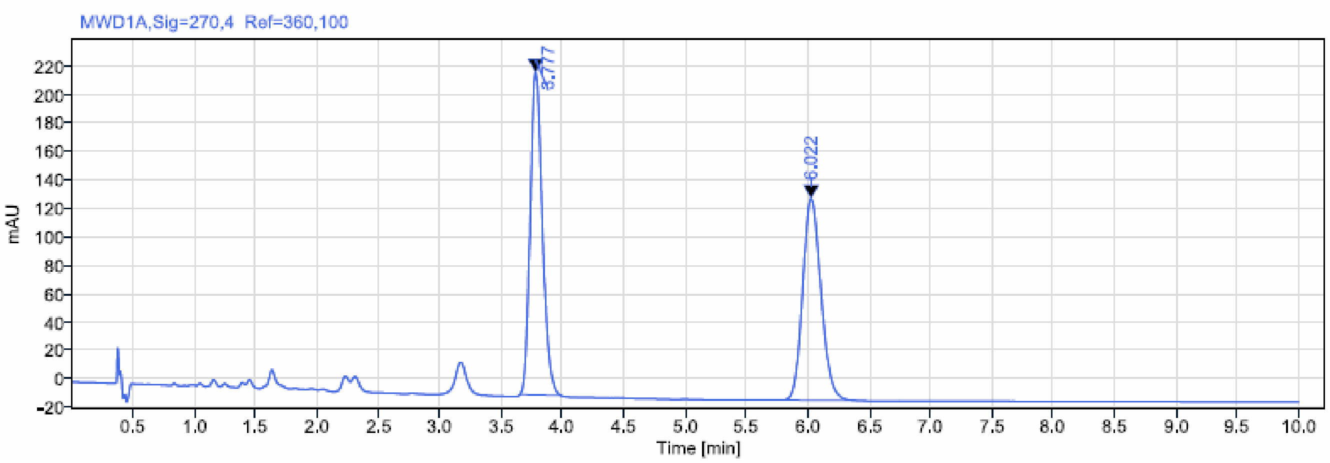

Signal: MWD1A,Sig=270,4 Ref=360,100

RT [min]	Type	Width [min]	Area	Height	Area%	Name
3,777	MM m	0,33	1556,51	226,46	51,41	
6,022	MM m	0,60	1471,33	141,64	48,59	
		**Sum**	**3027,83**			

SFC chromatogram of enantioenriched compound 9 (97% ee):

**Data file:**	Run IH 16% MeOH 10 minutes.amx2023-10-13 18-37-22+02-00VG-2-112-A-enantio.dx		
**Sequence Name:**	SFC-2023-10-13 18-23-11+02-00	**Project Name:**	Data
**Sample name:**	VG-2-112-A-enantio	**Operator:**	SYSTEM
**Instrument:**	SFC	**Injection date:**	2023-10-13 18:37:54+02:00
**Inj. volume:**	2,000 µL	**Location:**	D1F-F2
**Acq. method:**	Run IH 16% MeOH 10 minutes.amx	**Type:**	Sample
**Processing method:**	*Manual Integration.pmx	**Column:**	IH-3
**Manually modified:**	Manual Integration		

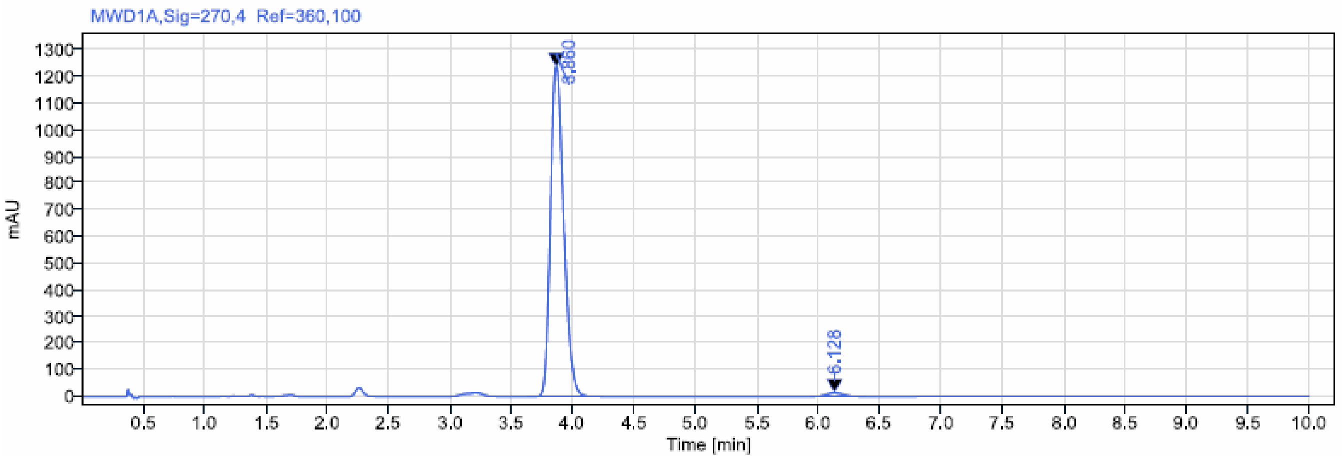

Signal: MWD1A,Sig=270,4 Ref=360,100

RT [min]	Type	Width [min]	Area	Height	Area%	Name
3,860	MM m	0,60	9261,30	1238,86	98,50	
6,128	MM m	0,44	141,04	13,81	1,50	
		**Sum**	**9402,34**			

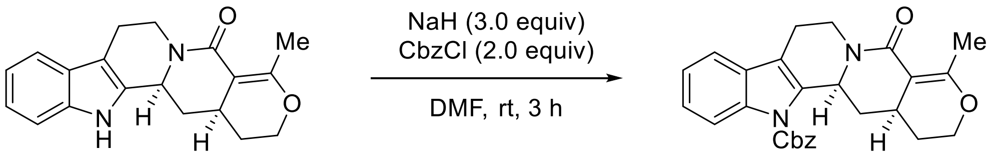

9 **17**

To the crude compound **9** (14.1 mmol) in dry DMF (140 mL, 0.1 M) at 0 °C was added NaH (1.7 g, 42.3 mmol, 60%wt in mineral oil, 3.0 equiv) portionwise under Ar. The reaction mixture was stirred at 0 °C for 1 h, before slow addition of benzyl chloroformate (4.0 mL, 28.2 mmol, 2.0 equiv). The mixture was then stirred at 0 °C for 30 minutes, then at rt for 3 h. The reaction was quenched with aqueous $NaHCO_3$ solution and water was added. The mixture was extracted with EtOAc three times. The combined organic layers were washed

with brine, dried over $MgSO_4$, filtered and evaporated *in vacuo*. The product was purified by FCC (SiO_2, from hexane to 1/1 hexane/EtOAc) to afford the *N*-Cbz indole **17** (4.5 g, 10.2 mmol, 72% over 2 steps) as a pale-yellow solid.

benzyl (13b*S*,14a*R*)-4-methyl-5-oxo-1,7,8,13b,14,14a-hexahydro-5*H*-indolo[2,3-a]pyrano[3,4-g]quinolizine-13(2*H*)-carboxylate (17)

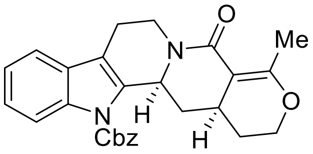

17

Pale-yellow solid.

Rf = 0.5 (1/1 EtOAc/hexane).

^{1}H NMR (600 MHz, $CDCl_3$) δ 8.08 (d, *J* = 8.1 Hz, 1H), 7.52 – 7.47 (m, 2H), 7.46 – 7.39 (m, 4H), 7.29 (td, *J* = 7.8, 1.5 Hz, 1H), 7.26 (td, *J* = 7.4, 1.2 Hz, 1H), 5.55 (d, *J* = 11.8 Hz, 1H), 5.34 (d, *J* = 11.8 Hz, 1H), 5.19 – 5.15 (m, 1H), 5.05 (dd, *J* = 10.8, 3.2 Hz, 1H), 4.20 (ddd, *J* = 10.6, 4.0, 2.1 Hz, 1H), 3.85 (ddd, *J* = 12.8, 10.6, 2.2 Hz, 1H), 2.81 – 2.71 (m, 2H), 2.70 – 2.65 (m, 1H), 2.51 (dt, *J* = 12.3, 3.3 Hz, 1H), 2.33 (d, *J* = 1.4 Hz, 3H), 2.33-2.27 (m, 1H), 1.76 – 1.70 (m, 1H), 1.39 (tdd, *J* = 12.9, 11.2, 4.0 Hz, 1H), 1.13 (td, *J* = 12.4, 10.8 Hz, 1H).

^{13}C NMR (151 MHz, $CDCl_3$) δ 165.1, 162.0, 151.5, 136.9, 135.6, 134.8, 129.2, 129.1, 129.1, 129.0, 125.0, 123.5, 119.8, 118.5, 115.8, 103.7, 69.3, 66.0, 55.5, 39.2, 36.1, 30.3, 28.8, 22.1, 20.7.

HRMS (ESI/QTOF) m/z: $[M + H]^+$ Calcd for $C_{27}H_{27}N_2O_4^+$ 443.1965; Found 443.1966.

IR (ν_{max}, cm^{-1}) 3051 (w), 2918 (w), 2879 (w), 1728 (m), 1641 (m), 1585 (m), 1455 (m), 1403 (m), 1386 (m), 1339 (m), 1308 (m), 1291 (m), 1281 (m), 1252 (m), 1211 (s), 1160 (m), 1116 (m), 1065 (m), 1024 (m), 827 (m), 732 (s), 698 (s).

M.p. = 171 – 173 °C.

$[\alpha]_D^{24}$ = -340 (*c* 0.3, $CHCl_3$).

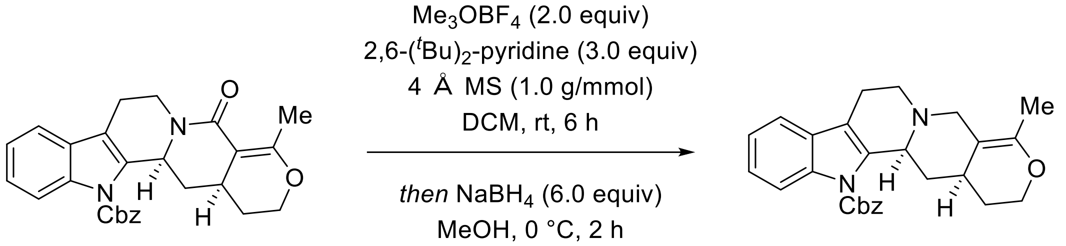

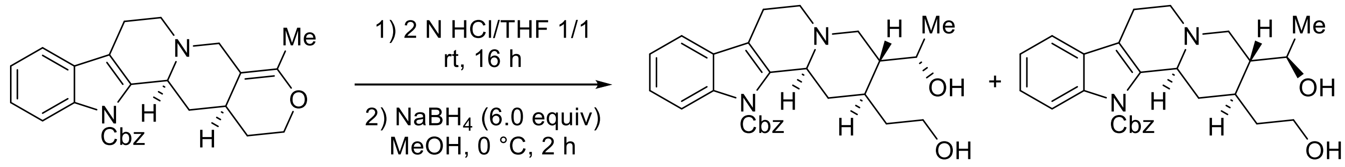

19a:19b, dr 3:1

A solution of amide **17** (4.2 g, 9.4 mmol, 1.0 equiv), 2,6-di-*tert*-butyl pyridine (6.1 mL, 28.2 mmol, 3.0 equiv) and 4 Å activated MS (9.4 g, 1.0 g/mmol) in DCM (94 mL, 0.1 M) was stirred at rt for 10 minutes under Ar. Meerwein's salt Me_3OBF_4 (2.8 g, 18.8 mmol, 2.0 equiv) was added at rt, and the solution was stirred at this temperature for 6 h and turned to an orange solution. DCM was removed under vacuum and dry MeOH (94 mL, 0.1 M) was added. The reaction mixture was cooled to 0 °C, and $NaBH_4$ (2.1 g, 56.4 mmol, 6.0 equiv) was added portionwise. The mixture was then stirred at 0 °C for 2 h. The reaction was quenched by addition of aqueous saturated solution of Na_2CO_3 and the mixture was extracted five times with EtOAc. The combined organic layers were washed with brine, dried over anhydrous $MgSO_4$, then filtered and concentrated under reduced pressure to give crude amine enol ether **18** that was used for the next step without further purification.

A solution of the crude amine enol ether **18** in a 1/1 mixture of 2 N aqueous HCl/THF was stirred at rt for 16 h. A saturated aqueous solution of Na_2CO_3 was added, and the mixture was extracted four times with EtOAc. The combined organic layers were washed with brine, dried over anhydrous $MgSO_4$, then filtered and concentrated under reduced pressure to give the crude hemiketal that was used for the next step without further purification.

The crude hemiketal was dissolved in dry MeOH (94 mL, 0.1 M) and cooled to 0 °C. $NaBH_4$ (2.1 g, 56.4 mmol, 6.0 equiv) was added portionwise. The mixture was then stirred at 0 °C for 2 h. The remaining $NaBH_4$ was quenched by addition of saturated aqueous solution of Na_2CO_3 and the mixture was extracted five times with EtOAc. The combined organic layers were washed with brine, dried over anhydrous $MgSO_4$, then filtered and concentrated under reduced pressure to give the crude diol. Trituration with 1/1 hexane/Et_2O to remove the remaining 2,6-di-*tert*-butyl pyridine give diol **S19a:S19b** (3.2 g, 7.2 mmol, 76% yield over 3 steps) as an

inseparable 3:1 mixture of diastereoisomers that was used without further purification. Crystals were obtained by slow evaporation from a 1/25/25 MeOH/acetone/hexane mixture.

N.B.: The crude diol could be purified by FCC (SiO_2, 1/5 MeOH/EtOAc + 2% NEt_3) but the poor solubility of ***S19a:S19b*** *made it stick on silica.*

benzyl (2*R*,3*R*,12b*S*)-3-(1-hydroxyethyl)-2-(2-hydroxyethyl)-1,3,4,6,7,12b-hexahydroindolo[2,3-a]quinolizine-12(2*H*)-carboxylate (S19)

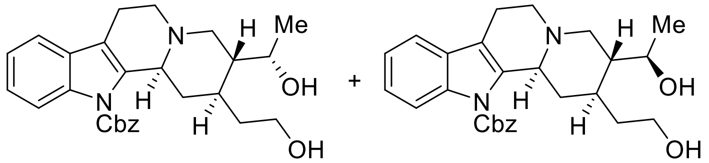

19a **19b**

19a:19b, dr 3:1

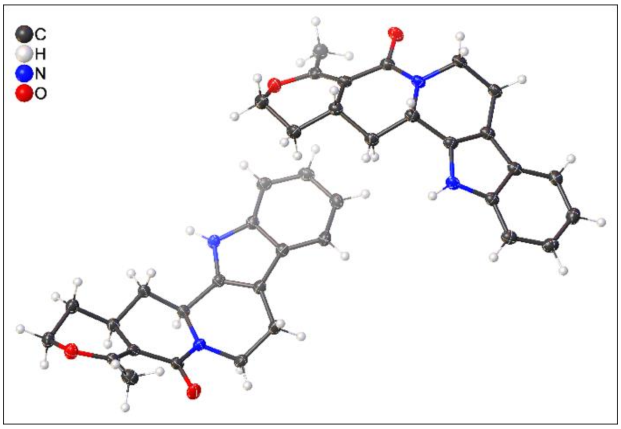

X-Ray structure of 19a (50% ellipsoid probability level)

White/beige solid.

Rf = 0.32 (1/7 MeOH/ EtOAc + 2% NEt_3).

^{1}H NMR (800 MHz, $CDCl_3$) (inseparable 3:1 mixture of diastereoisomers) δ 8.08 (d, *J* = 8.1 Hz, 1H), 7.50 (d, *J* = 7.0 Hz, 2H), 7.44 – 7.35 (m, 4H), 7.26 – 7.18 (m, 2H), 5.46 – 5.39 (m, 2H), 4.16 (qd, *J* = 6.6, 2.1 Hz, 0.75H), 4.11 (dt, *J* = 11.2, 5.5 Hz, 0.25H), 4.04-3.93 (m, 1H), 3.66 (dt, *J* = 10.6, 5.4 Hz, 0.75H), 3.63 – 3.59 (m, 0.25H), 3.53-3.50 (m, 1H), 3.25 (dd, *J* = 12.7, 4.0 Hz, 0.25H), 3.14 – 3.08 (m, 1.75H), 2.89 – 2.80 (m, 1H), 2.80 – 2.69 (m, 2.75H), 2.62 (t, *J* = 12.1 Hz, 0.25H), 2.14 (d, *J* = 12.8 Hz, 0.75H), 2.08 (d, *J* = 12.6 Hz, 0.25H), 1.88 – 1.78 (m, 1H), 1.73-1.69 (m, 0.25H), 1.69 – 1.63 (m, 0.75H), 1.58-1.52 (m, 1H), 1.43 – 1.35 (m, 0.25H), 1.35 – 1.26 (m, 1.75H), 1.21 (d, *J* = 6.5 Hz, 2.25H), 1.11 (d, *J* = 6.6 Hz, 0.75H).

^{13}C NMR (201 MHz, $CDCl_3$) (inseparable 3:1 mixture of diastereoisomers) δ 151.7 (major), 151.7 (minor), 136.7 (major), 136.7 (minor), 135.1 (major), 135.0 (minor), 129.6 (major), 129.6 (minor), 129.0, 129.0, 128.9, 128.9, 124.4 (minor), 124.4 (major), 123.3 (minor), 123.2 (major), 118.3 (minor), 118.2 (major), 116.9 (minor), 116.8 (major), 115.8 (major + minor), 69.1 (minor), 69.0 (major), 67.5 (minor), 65.8 (major), 60.7 (major), 60.1 (minor), 59.2 (minor), 59.0 (major), 54.6 (minor), 54.4 (major), 47.8 (major), 47.7 (minor), 43.4 (major + minor), 35.8 (minor), 35.4 (minor), 35.1 (major), 34.7 (major), 34.0 (minor), 34.0 (major), 34.0 (minor), 22.3 (major), 22.3 (minor), 20.3 (major), 17.8 (minor).

HRMS (ESI/QTOF) m/z: $[M + H]^+$ Calcd for $C_{27}H_{33}N_2O_4^+$ 449.2435; Found 449.2430.

IR (ν_{max}, cm^{-1}) 3339 (w), 2967 (w), 2923 (w), 1729 (s), 1611 (s), 1456 (s), 1390 (s), 1349 (s), 1313 (s), 1259 (m), 1214 (s), 1161 (s), 1116 (m), 1086 (m), 1047 (s), 1027 (s), 881 (w), 747 (s), 698 (s), 664 (m).

M.p. = 161 – 162 °C.

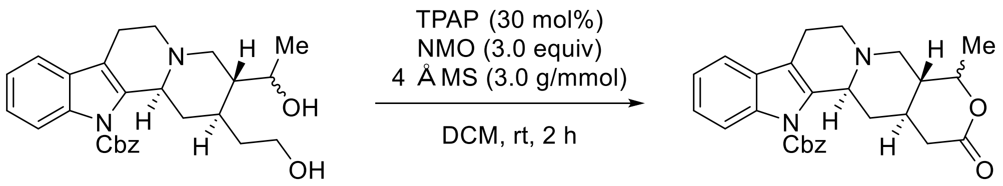

19a:19b, d.r. 3:1 **8a:8b**, d.r. 3:1

A solution of diol **S19a:S19b** (2.1 g, 4.7 mmol, 1.0 equiv), 4 Å activated MS (14.1 g, 3.0 g/mmol) and anhydrous NMO (1.65 g, 14.1 mmol, 3.0 equiv) in dry DCM (120 mL, 0.04 M) was stirred at rt for 5 minutes. Then dry TPAP (495 mg, 1.4 mmol, 30 mol%) was added and the reaction mixture was stirred at rt for 2 h. The solvent was evaporated under reduced pressure and the residue was purified by FCC (SiO_2, 3/7 EtOAc/hexane + 2% NEt_3 to EtOAc + 2% NEt_3) to afford a combined mixture of separable diastereoisomers (dr 3:1) of lactone **8a:8b** (1.5 g, 3.3 mmol, 57% yield over 4 steps) as a pale-yellow foam. Under these purification conditions, the two diastereomers were separated for characterization.

Note: *70% yield was obtained from FCC (1/5 MeOH/EtOAc + 2% NEt_3) purified diol **19**.*

benzyl (4*S*,4a*R*,13b*S*,14a*R*)-4-methyl-2-oxo-1,4a,5,7,8,13b,14,14a-octahydro-4*H*-indolo[2,3-a]pyrano[3,4-g]quinolizine-13(2*H*)-carboxylate (8a)

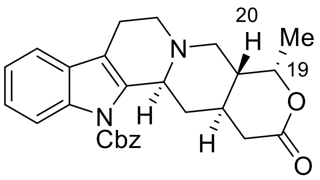

8a

Pale-yellow foam.

Rf = 0.32 (EtOAc + 2% NEt_3).

^{1}H NMR (600 MHz, $CDCl_3$) δ 8.12 (d, *J* = 8.2 Hz, 1H), 7.52 – 7.48 (m, 2H), 7.47 – 7.42 (m, 3H), 7.41 (d, *J* = 7.3 Hz, 1H), 7.28 (dt, *J* = 8.1, 4.4 Hz, 1H), 7.26 – 7.23 (m, 1H), 5.50 (d, *J* = 11.6 Hz, 1H), 5.31 (d, *J* = 11.6 Hz, 1H), 4.60 (p, *J* = 6.1 Hz, 1H), 3.95 (d, *J* = 10.6 Hz, 1H), 3.15 – 3.07 (m, 1H), 2.99 (dd, *J* = 12.4, 3.8 Hz, 1H), 2.84 – 2.69 (m, 3H), 2.56 (t, *J* = 11.9 Hz, 1H), 2.44 (dd, *J* = 18.1, 5.6 Hz, 1H), 2.13 – 2.06 (m, 1H), 2.04 (d, *J* = 12.5 Hz, 1H), 1.98 (dd, *J* = 18.2, 11.8 Hz, 1H), 1.57 – 1.46 (m, 1H), 1.22 (d, *J* = 6.8 Hz, 3H), 1.18 (q, *J* = 11.7 Hz, 1H).

^{13}C NMR (151 MHz, $CDCl_3$) δ 170.1, 151.4, 136.7, 136.0, 134.8, 129.4, 129.3, 129.3, 129.1, 124.6, 123.3, 118.3, 117.0, 115.8, 77.7, 69.2, 58.3, 56.1, 47.4, 37.0, 36.7, 34.5, 29.3, 22.5, 17.2.

HRMS (ESI/QTOF) m/z: $[M + H]^+$ Calcd for $C_{27}H_{29}N_2O_4^+$ 445.2122; Found 445.2127.

IR (ν_{max}, cm^{-1}) 2978 (w), 2914 (w), 2817 (w), 1724 (s), 1457 (m), 1391 (m), 1348 (m), 1315 (s), 1213 (s), 1162 (m), 1115 (m), 1075 (w), 1024 (m), 983 (w), 911 (w), 752 (m), 730 (m), 700 (m).

$[\alpha]_D^{24}$ = -188 (*c* 0.2, $CHCl_3$).

*N.B.: in agreement with the X-Ray structure of **19a**, the 6.1 Hz coupling constant between H(19) and H(20) (4.60 (p, J = 6.1 Hz, 1H)) shows that H(19)/H(20) are more likely cis.*

benzyl (4*R*,4a*R*,13b*S*,14a*R*)-4-methyl-2-oxo-1,4a,5,7,8,13b,14,14a-octahydro-4*H*-indolo[2,3-a]pyrano[3,4-g]quinolizine-13(2*H*)-carboxylate (8b)

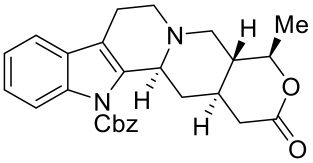

8b

Pale-yellow foam.

Rf = 0.51 (EtOAc + 2% NEt_3).

^{1}H NMR (600 MHz, $CDCl_3$) δ 8.11 (d, *J* = 8.2 Hz, 1H), 7.51 – 7.47 (m, 2H), 7.46 – 7.39 (m, 4H), 7.31 – 7.27 (m, 1H), 7.26 – 7.22 (m, 1H), 5.51 (d, *J* = 11.9 Hz, 1H), 5.31 (d, *J* = 12.0 Hz, 1H), 4.06-4.01 (m, 1H), 3.99 (d, *J* = 10.6 Hz, 1H), 3.14-3.09 (m, 2H), 2.84-2.70 (m, 4H), 2.50 (t, *J* = 11.8 Hz, 1H), 2.43 (dd, *J* = 18.1, 4.8 Hz, 1H), 2.05 – 1.99 (m, 1H), 1.98 (d, *J* = 12.1 Hz, 1H), 1.60 – 1.53 (m, 1H), 1.35 (d, *J* = 6.1 Hz, 3H), 1.17 (q, *J* = 11.7 Hz, 1H).

^{13}C NMR (151 MHz, $CDCl_3$) δ 170.0, 151.4, 136.7, 136.0, 134.9, 129.4, 129.3, 129.1, 124.6, 123.3, 118.3, 117.0, 115.8, 80.3, 69.2, 58.4, 56.6, 47.8, 40.9, 36.8, 35.5, 34.0, 22.5, 20.0.

HRMS (ESI/QTOF) m/z: $[M + H]^+$ Calcd for $C_{27}H_{29}N_2O_4^+$ 445.2122; Found 445.2127.

IR (ν_{max}, cm^{-1}) 2948 (w), 2906 (w), 1727 (s), 1456 (m), 1389 (m), 1348 (m), 1315 (m), 1262 (m), 1215 (m), 1196 (m), 1163 (m), 1115 (m), 1025 (m), 988 (w), 911 (w), 752 (m), 731 (m), 699 (m).

$[\alpha]_D^{24}$ = -156 (*c* 0.2, $CHCl_3$).

b) (-)-Ajmalicine and (+)-mayumbine total synthesis

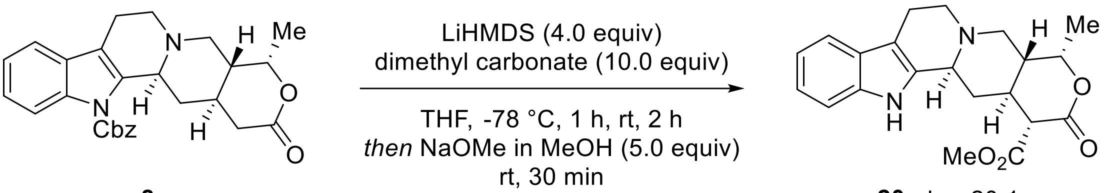

8a **20**, dr > 20:1

At -78 °C, LiHMDS (1.0 M in THF, 0.5 mL, 0.44 mmol, 4.0 equiv) was added dropwise to a solution of lactone **8a** (50 mg, 0.11 mmol, 1.0 equiv) in THF (1.1 mL). The reaction mixture was stirred at -78 °C for 1 h before dropwise addition of anhydrous dimethyl carbonate (95 µL, 1.1 mmol, 10.0 equiv). The reaction mixture was stirred at -78 °C for 1 h, then was let slowly warm up to rt and it was stirred for 2 more hours at this temperature. NaOMe (5.4 M in MeOH, 102 µL, 0.55 mmol, 5.0 equiv) was slowly added and the mixture was stirred at rt for 30 minutes. The mixture was then poured into $NaHCO_3$ aqueous solution, and it was extracted 4 times with DCM. The combined organic layers were washed with brine, dried over $MgSO_4$, and concentrated under reduced pressure. The residue was purified by FCC (SiO_2, EtOAc) to afford the desired malonate **20** (dr > 20:1) (30.8 mg, 83.6 µmol, 75% yield) as a white powder.

*N.B.: Due to solubility issues (insoluble or low solubility) of compound **20** in most of the solvents, the NMR spectra were measured in methanol-d$_4$. Hence, the indole-NH proton as well as the acidic malonate α-proton were not observed and the corresponding malonate α-carbon was observed as a triplet due to C-D coupling.*

Methyl (1*S*,4*S*,4a*R*,13b*S*,14a*S*)-4-methyl-2-oxo-1,2,4a,5,7,8,13,13b,14,14a-decahydro-4*H*-indolo[2,3-a]pyrano[3,4-g]quinolizine-1-carboxylate (20)

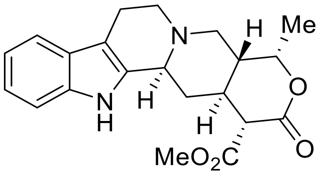

20

White powder.

Rf = 0.35 (EtOAc).

¹H NMR (600 MHz, CD_3OD) δ 7.39 (dt, *J* = 7.8, 1.0 Hz, 1H), 7.28 (dt, *J* = 8.1, 0.9 Hz, 1H), 7.05 (ddd, *J* = 8.2, 7.0, 1.2 Hz, 1H), 6.97 (ddd, *J* = 8.0, 7.0, 1.0 Hz, 1H), 4.82 – 4.76 (m, 1H), 3.85 (s, 3H), 3.42 – 3.38 (m,

1H), 3.18 – 3.14 (m, 1H), 3.05 (d, J = 7.4 Hz, 1H), 3.02 – 2.95 (m, 1H), 2.78 – 2.74 (m, 1H), 2.71 (td, J = 11.2, 4.5 Hz, 1H), 2.52 – 2.45 (m, 2H), 2.38 – 2.30 (m, 2H), 1.40 (d, J = 6.8 Hz, 3H), 1.39 – 1.35 (m, 1H) (The NH-indole and the acidic malonate α-proton were deuterated in CD_3OD).

^{13}C NMR (151 MHz, CD_3OD) δ 170.9, 169.0, 138.1, 134.8, 128.2, 122.1, 119.8, 118.6, 112.0, 108.0, 79.8, 60.5, 56.9, 55.0 (t, J = 19.2 Hz, O(O)C-**C**D-CO_2Me), 54.2, 53.2, 39.5, 35.0, 33.9, 22.5, 17.6.

HRMS (APCI/QTOF) m/z: $[M + Na]^+$ Calcd for $C_{21}H_{24}N_2NaO_4^+$ 391.1628; Found 391.1623.

IR (ν_{max}, cm^{-1}) 3324 (w), 2921 (w), 2849 (w), 2815 (w), 2766 (w), 1753 (m), 1707 (s), 1452 (w), 1438 (w), 1382 (m), 1374 (m), 1325 (m), 1273 (m), 1254 (m), 1192 (m), 1147 (s), 1087 (m), 1056 (m), 1015 (m), 979 (w), 901 (w), 829 (w), 741 (s), 726 (m), 674 (m), 648 (w), 609 (m).

Mp = 215 – 220 °C (decomposition).

$[\alpha]_D^{24}$ = -21 (c 0.1, MeOH).

N.B.: *NMR spectra of* ***20*** *have also been measured in $CDCl_3$ in presence of EtOAc (making the compound soluble). Coupling constant of 11.2 Hz between α-H malonate (3.22 (d, J = 11.2 Hz, 1H)) and H(15)* (2.48 (qd, J = 11.3, 4.1 Hz, 1H)) *shows the trans-conformation between those two protons.*

^{1}H NMR (400 MHz, $CDCl_3$ + 4.5 equiv of EtOAc) δ 7.80 (s, 1H), 7.49 – 7.46 (m, 1H), 7.31 (dt, J = 8.1, 1.0 Hz, 1H), 7.15 (ddd, J = 8.1, 7.1, 1.4 Hz, 1H), 7.10 (td, J = 7.4, 1.2 Hz, 1H), 4.78 – 4.71 (m, 1H), 3.88 (s, 3H), 3.34 (dd, J = 11.9, 1.3 Hz, 1H), 3.22 (d, J = 11.2 Hz, 1H), 3.11 (dd, J = 10.7, 5.8 Hz, 1H), 3.02 (dd, J = 10.2, 2.8 Hz, 1H), 3.01 – 2.95 (m, 1H), 2.79 – 2.73 (m, 1H), 2.69 (td, J = 11.1, 4.2 Hz, 1H), 2.48 (qd, J = 11.3, 4.1 Hz, 1H), 2.40 – 2.19 (m, 3H), 1.50 – 1.44 (m, 1H), 1.43 (d, J = 6.8 Hz, 3H).

^{13}C NMR (101 MHz, $CDCl_3$ + 4.5 equiv of EtOAc) δ 169.2, 166.1, 136.3, 127.3, 122.0, 119.8, 118.4, 111.0, 108.7, 78.1, 59.1, 56.3, 54.4, 53.3, 53.2, 39.1, 35.1, 32.8, 29.8, 21.8, 17.7.

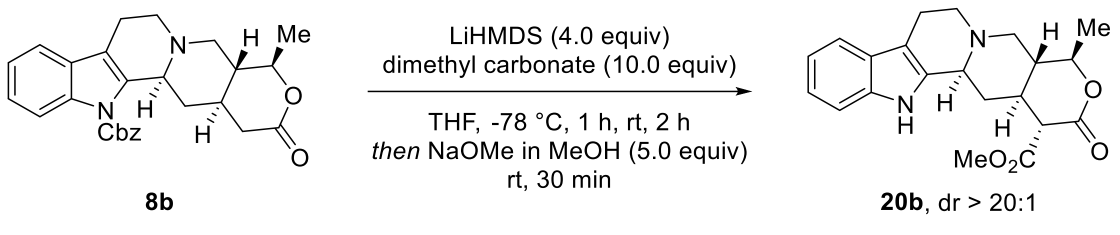

At -78 °C, LiHMDS (1.0 M in THF, 0.5 mL, 0.44 mmol, 4.0 equiv) was added dropwise to a solution of lactone **8b** (50 mg, 0.11 mmol, 1.0 equiv) in THF (1.1 mL). The reaction mixture was stirred at -78 °C for 1

h before dropwise addition of anhydrous dimethyl carbonate (95 µL, 1.1 mmol, 10.0 equiv). The reaction mixture was stirred at -78 °C for 1 h, then was let slowly warm up to rt over 30 minutes and it was stirred for 2 more hours at this temperature. NaOMe (5.4 M in MeOH, 102 µL, 0.55 mmol, 5.0 equiv) was slowly added and the mixture was stirred at rt for 30 minutes. The mixture was then poured into $NaHCO_3$ aqueous solution, and it was extracted 4 times with DCM. The combined organic layers were washed with brine, dried over $MgSO_4$, and concentrated under reduced pressure. The residue was purified by FCC (SiO_2, EtOAc) to afford the desired malonate **20b** (dr > 20:1) (31.0 mg, 84.2 µmol, 76% yield) as a beige powder.

Methyl (1*S*,4*R*,4a*R*,13b*S*,14a*S*)-4-methyl-2-oxo-1,2,4a,5,7,8,13,13b,14,14a-decahydro-4*H*-indolo[2,3-a]pyrano[3,4-g]quinolizine-1-carboxylate (20b)

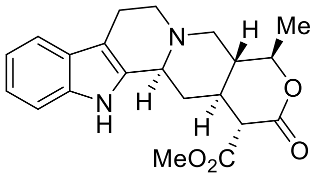

20b

Beige powder.

Rf = 0.65 (EtOAc).

^{1}H NMR (600 MHz, CD_2Cl_2) δ 7.87 (s, 1H), 7.45 (d, *J* = 7.8 Hz, 1H), 7.31 (d, *J* = 8.0 Hz, 1H), 7.12 (t, *J* = 7.3 Hz, 1H), 7.06 (t, *J* = 7.6 Hz, 1H), 4.33 (dq, *J* = 12.2, 6.6 Hz, 1H), 3.84 (s, 3H), 3.37 (d, *J* = 11.6 Hz, 1H), 3.25 (d, *J* = 12.1 Hz, 1H), 3.15 – 3.07 (m, 2H), 2.96 – 2.86 (m, 1H), 2.75 – 2.65 (m, 2H), 2.31 – 2.19 (m, 2H), 2.13 (d, *J* = 12.7 Hz, 1H), 1.85 – 1.77 (m, 1H), 1.42 (d, *J* = 6.4 Hz, 3H), 1.40 – 1.34 (m, 1H).

^{13}C NMR (151 MHz, CD_2Cl_2) δ 169.4, 166.2, 136.6, 134.2, 127.6, 121.9, 119.8, 118.4, 111.2, 108.8, 80.7, 59.1, 56.7, 54.9, 53.1, 42.8, 38.7, 34.8, 30.1, 22.2, 20.0.

HRMS (ESI/QTOF) m/z: $[M + H]^+$ Calcd for $C_{21}H_{25}N_2O_4^+$ 369.1809; Found 369.1800.

IR (ν_{max}, cm^{-1}) 3330 (w), 2951 (w), 2917 (w), 2898 (w), 2847 (w), 2813 (w), 2759 (w), 1751 (m), 1706 (s), 1453 (w), 1438 (w), 1387 (w), 1357 (w), 1322 (w), 1264 (m), 1216 (m), 1198 (m), 1147 (m), 1114 (m), 1084 (w), 1053 (m), 1037 (w), 1011 (w), 980 (w), 840 (w), 742 (s), 728 (w), 692 (w), 674 (w)

Mp = 248 – 250 °C (decomposition).

$[\alpha]_D^{24}$ = -13 (*c* 0.1, CH_2Cl_2)

N.B.: Coupling constant of 12.1 Hz between α-H malonate (3.25 (d, J = 12.1 Hz, 1H)) and H(15) shows the two protons are in a trans relationship.

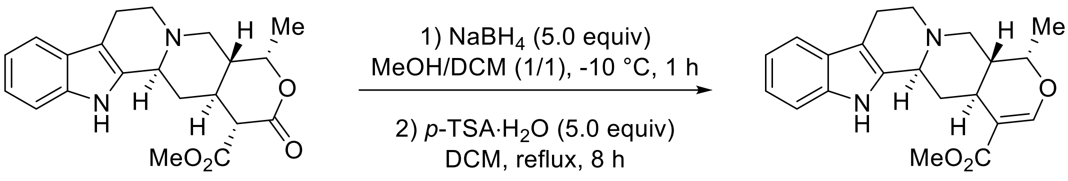

20 **1 (-)-Ajmalicine**

At -10 °C, using a cryostat, $NaBH_4$ (9.5 mg, 0.25 mmol, 5.0 equiv) was added portionwise to a stirred solution of **20** (18.4 mg, 50.0 µmol, 1.0 equiv) in a 1/1 mixture of dry DCM/MeOH (4.0 mL) and the mixture was stirred at -10 °C for 1 h. The solution was then directly poured into aqueous saturated Na_2CO_3 solution and it was extracted with DCM four times. The combined organic layers were washed with brine, dried over $MgSO_4$, filtered and the filtrate was concentrated under reduced pressure. The crude lactol (1.0 equiv) was then dissolved in DCM (2.5 mL). *p*-TSA·H_2O (47.5 mg, 0.25 mmol, 5.0 equiv) was added and the reaction mixture was heated to reflux for 8 h. The solution was poured into aqueous saturated Na_2CO_3 solution and it was extracted with DCM five times. The combined organic layers were washed with brine, dried over $MgSO_4$, filtered and the filtrate was concentrated under reduced pressure. The residue was purified by FCC (SiO_2, 1/2 EtOAc/hexane) to afford (-)-ajmalicine **1** (10.0 mg, 28.3 µmol, 57% yield over 2 steps) as a white solid. A sample was recrystallized by slow open-air evaporation from a 1/3/3 DCM/acetone/hexane mixture to afford (-)-ajmalicine hydrate as colorless crystals.

All characterization data were in full agreement with those reported in the literature.[3–7]

(-)-Ajmalicine (1)

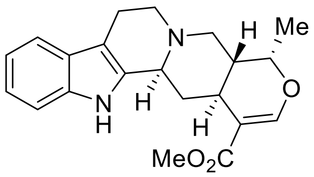

1 (-)-Ajmalicine

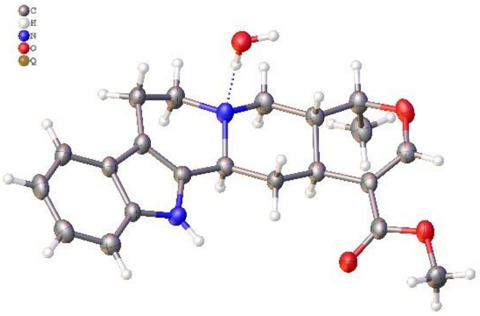

X-Ray structure of 1·H_2O (50% ellipsoid probability level)

Colorless crystals.

Rf = 0.5 (1/1 EtOAc/hexane).

^{1}H NMR (600 MHz, $CDCl_3$) δ 7.97 (s, 1H), 7.53 (d, *J* = 1.8 Hz, 1H), 7.46 (dd, *J* = 7.8, 1.1 Hz, 1H), 7.30 (dt, *J* = 8.0, 0.9 Hz, 1H), 7.14 (ddd, *J* = 8.2, 7.1, 1.2 Hz, 1H), 7.08 (ddd, *J* = 8.0, 7.1, 1.1 Hz, 1H), 4.43 (qd, *J* =

6.6, 3.9 Hz, 1H), 3.74 (s, 3H), 3.42 (d, *J* = 11.4 Hz, 1H), 3.21 (dt, *J* = 12.5, 3.1 Hz, 1H), 3.12 (dd, *J* = 11.2, 5.9 Hz, 1H), 3.06 – 2.97 (m, 1H), 2.99 (dd, *J* = 10.6, 2.9 Hz, 1H), 2.78 – 2.73 (m, 1H), 2.70 (td, *J* = 11.2, 4.3 Hz, 1H), 2.43 (tdd, *J* = 11.4, 3.5, 1.9 Hz, 1H), 2.26 (t, *J* = 10.9 Hz, 1H), 2.17 (t, *J* = 11.1 Hz, 1H), 1.33 (q, *J* = 11.8 Hz, 1H), 1.19 (d, *J* = 6.7 Hz, 3H).

^{13}C NMR (151 MHz, $CDCl_3$) δ 167.6, 154.8, 136.1, 134.4, 127.4, 121.6, 119.6, 118.2, 111.0, 108.1, 106.8, 73.8, 60.3, 57.0, 53.4, 51.1, 41.1, 33.0, 30.8, 21.9, 15.1.

HRMS (APCI/QTOF) m/z: $[M + H]^+$ Calcd for $C_{21}H_{25}N_2O_3^+$ 353.1860; Found 353.1858.

IR (ν_{max}, cm^{-1}) 3578 (w), 3208 (w), 2915 (m), 2849 (w), 1795 (w), 1685 (m), 1614 (s), 1499 (w), 1438 (m), 1380 (m), 1356 (w), 1322 (m), 1299 (s), 1283 (m), 1214 (w), 1190 (s), 1158 (m), 1106 (s), 1003 (w), 961 (w), 929 (w), 893 (w), 849 (m), 803 (m), 788 (m), 769 (m), 742 (s), 607 (w).

Mp = 255 – 260 °C (decomposition).

$[\alpha]_D^{25}$ = -61 (*c* 0.2, $CHCl_3$); lit.[3] $[\alpha]_D^{25}$ = −60.0 (*c* 0.05, $CHCl_3$).

Table S3: Chemical shifts of ^1H NMR spectra of natural,[5,6], synthetic (-)-ajmalicine in the literature[3], and our synthetic (-)-ajmalicine **1** ($CDCl_3$).

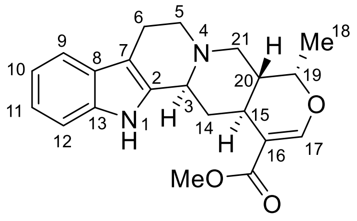

1 (-)-Ajmalicine

Proton	δH (ppm) natural[6], multi (J, Hz), 400 MHz	δH (ppm) synthetic,[3] multi (J, Hz), 500 MHz	Synthetic δH (ppm) synthetic, multi (J, Hz), 600 MHz
18 (Me)	1.18, 3H, d, 6	1.19, 3H, d, 6.6	1.19, 3H, d, 6.7
14b	1.32, 1H, q, 12	1.31, 1H, q, 11.6	1.33, 1H, q, 11.8
20	2.14, 1H, tt, 12, 3	2.15, 1H, tt, 11.2, 3.4	2.17, 1H, t, 11.1
21a	2.25, 1H, t, 12	2.26, 1H, t, 10.9	2.26, 1H, t, 10.9
15	2.45, 1H, tdd, 12, 3, 1.5	2.44, 1H, tdd, 11.4, 3.6, 1.9	2.43, 1H, tdd, 11.4, 3.5, 1.9
5a	2.68, 1H, td, 12, 4	2.69, 1H, t, 10.9	2.70, 1H, td, 11.2, 4.3
6a	2.75, 1H, brd, 16	2.77 – 2.72, 1H, m	2.78 – 2.73, 1H, m
21b	2.97, 1H, dd, 12, 3	2.98, 1H, dd, 10.8, 3.0	2.99, 1H, dd, 10.6, 2.9
6b	3.00, 1H, ddd, 16, 11, 6	3.06 – 2.97, 1H, m	3.06 – 2.97, 1H, m
5b	3.10, 1H, dd, 12, 6	3.11, 1H, dd, 11.0, 5.8	3.12, 1H, dd, 11.2, 5.9
14a	3.22, 1H, dt, 12, 3	3.20, 1H, dt, 12.5, 3.1	3.21, 1H, dt, 12.5, 3.1
3	3.42, 1H, brd, 12	3.41, 1H, dd, 11.3, 2.3	3.42, 1H, d, 11.4
OMe	3.74, 3H, s	3.74, 3H, s	3.74, 3H, s
19	4.43, 1H, qd, 6, 3	4.43, 1H, qd, 6.6, 3.9	4.43, 1H, qd, 6.6, 3.9
10	7.09, 1H	7.08, 1H, ddd, 8.1, 7.2, 1.1	7.08, 1H, ddd, 8.0, 7.1, 1.1
11	7.14, 1H	7.14, 1H, ddd, 8.2, 7.0, 1.3	7.14, 1H, ddd, 8.2, 7.1, 1.2
12	7.30, 1H	7.30, 1H, dd, 8.0, 0.9	7.30, 1H, dt, 8.0, 0.9
9	7.47, 1H	7.46, 1H, dd, 7.7, 1.1	7.46, 1H, dd, 7.8, 1.1
17	7.54, 1H, d, 1.5	7.53, 1H, d, 1.8	7.53, 1H, d, 1.8
NH	7.96, 1H, brs	7.90, 1H, s	7.97, 1H, s

Table S4: Chemical shifts of ^{13}C NMR spectra of synthetic (-)-ajmalicine in the literature,[3,5] and our synthetic (-)-ajmalicine **1** ($CDCl_3$).

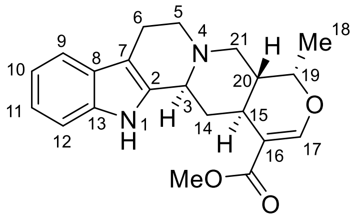

1 (-)-Ajmalicine

**Carbon**	**δC (ppm) synthetic,[5] 100 MHz**	**δC (ppm) synthetic,[3] 125 MHz**	**δC (ppm) our synthetic, 151 MHz**
18 (Me)	15.0	15.1	15.1
6	21.8	22.0	21.9
15	30.7	30.8	30.8
14	32.9	33.1	33.0
20	41.0	41.2	41.1
OMe	51.0	51.1	51.1
5	53.3	53.4	53.4
21	56.9	57.1	57.0
3	60.1	60.3	60.3
19	73.8	73.9	73.8
7	106.7	106.8	106.8
16	107.9	108.1	108.1
12	110.8	111.0	111.0
9	118.1	118.2	118.2
10	119.4	119.5	119.6
11	121.4	121.6	121.6
8	127.3	127.4	127.4
2	134.4	134.5	134.4
13	136.0	136.1	136.1
17	154.7	154.8	154.8
C=O	167.5	167.6	167.6

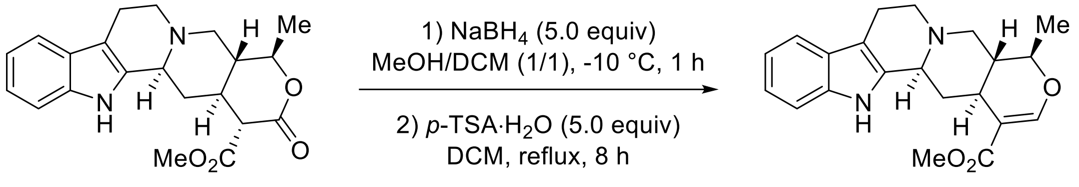

At -10 °C, using a cryostat, $NaBH_4$ (8.5 mg, 0.23 mmol, 5.0 equiv) was added portionwise to a stirred solution of **8b** (16.6 mg, 45.0 µmol, 1.0 equiv) in a 1/1 mixture of dry DCM/MeOH (3.6 mL) and the mixture was stirred at -10 °C for 1 h. The solution was then directly poured into aqueous saturated Na_2CO_3 solution and it was extracted with DCM four times. The combined organic layers were washed with brine, dried over $MgSO_4$, filtered and the filtrate was concentrated under reduced pressure. The crude lactol (1.0 equiv) was then dissolved in DCM (2.3 mL). *p*-TSA·H_2O (42.8 mg, 0.23 mmol, 5.0 equiv) was added and the reaction mixture was heated to reflux for 8 h. The solution was poured into aqueous saturated Na_2CO_3 solution and it was extracted with DCM five times. The combined organic layers were washed with brine, dried over $MgSO_4$, filtered and the filtrate was concentrated under reduced pressure. The residue was purified by FCC (SiO_2, 1/2 EtOAc/hexane) to afford (+)-mayumbine **2** (8.3 mg, 23.6 µmol, 53% yield over 2 steps) as a white solid.

All characterization data were in full agreement with those reported in the literature.[6,8–10]

(+)-Mayumbine (2)

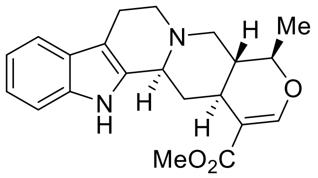

2 (+)-Mayumbine

White solid.

Rf = 0.35 (2/3 EtOAc/hexane).

^{1}H NMR (400 MHz, $CDCl_3$) δ 7.94 (s, 1H), 7.56 (d, *J* = 1.5 Hz, 1H), 7.47 (d, *J* = 7.7 Hz, 1H), 7.30 (d, *J* = 8.0 Hz, 1H), 7.14 (td, *J* = 7.5, 1.4 Hz, 1H), 7.08 (td, *J* = 7.4, 1.2 Hz, 1H), 3.88 (dq, *J* = 10.0, 6.3 Hz, 1H), 3.74 (s, 3H), 3.41 (d, *J* = 11.2 Hz, 1H), 3.19 – 3.10 (m, 3H), 3.09 – 2.97 (m, 1H), 2.79 – 2.68 (m, 2H), 2.40 (tt, *J* = 11.0, 2.7 Hz, 1H), 2.24 (t, *J* = 11.0 Hz, 1H), 1.77 (q, *J* = 9.8 Hz, 1H), 1.37 (d, *J* = 6.3 Hz, 3H), 1.31 – 1.26 (m, 1H).

^{13}C NMR (201 MHz, $CDCl_3$) δ 167.6, 156.2, 136.1, 134.6, 127.4, 121.6, 119.5, 118.2, 111.0, 108.1, 108.0, 75.7, 59.8, 56.4, 53.5, 51.1, 43.8, 36.7, 33.0, 21.9, 18.5.

HRMS (ESI/QTOF) m/z: $[M + H]^+$ Calcd for $C_{21}H_{25}N_2O_3^+$ 353.1860; Found 353.1855.

IR (ν_{max}, cm^{-1}) 3319 (w), 2948 (w), 2922 (m), 2851 (w), 1701 (m), 1686 (m), 1618 (m), 1451 (m), 1437 (m), 1375 (m), 1322 (m), 1290 (s), 1264 (m), 1187 (s), 1152 (m), 1101 (s), 1051 (m), 1036 (m), 1008 (w), 970 (w), 927 (w), 851 (w), 788 (m), 768 (m), 740 (s).

Mp = 205 – 210 °C (decomposition).

$[\alpha]_D^{25}$ = +57 (*c* 0.15, $CHCl_3$); lit.[8] $[\alpha]_D^{25}$ = +58.8 (*c* 0.17, $CHCl_3$).

Table S5: Chemical shifts of ^{1}H NMR spectra for the natural,[6] reported synthetic[9] and our synthetic (+)-mayumbine **2** ($CDCl_3$).

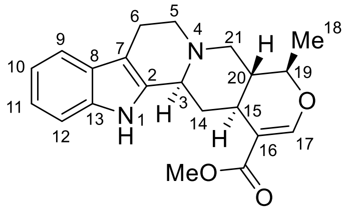

2 (+)-Mayumbine

Proton	δH (ppm) natural,[6] multi (J, Hz), 400 MHz	δH (ppm) synthetic,[9] multi (J, Hz), 400 MHz	Our δH (ppm) synthetic, multi (J, Hz), 400 MHz
14b	1.26, 1H, q, 12	1.27, 1H, q, 12.0	1.31 – 1.26, 1H, m
18 (Me)	1.36, 3H, d, 6	1.37, 3H, d, 6.4	1.37, 3H, d, 6.3
20	1.75, 1H, qd, 12, 3	1.76, 1H, qd, 10.8, 3.2	1.77, 1H, q, 9.8
21a	2.20, 1H, t, 12	2.23, 1H, t, 10.8	2.24, 1H, t, 11.0
15	2.38, 1H, tdd, 12, 3, 1.5	2.40, 1H, t, 10.8	2.40, 1H, tt, 11.0, 2.7
5a	2.68, 1H, td, 12, 4	2.77 – 2.66, 2H, m	2.79 – 2.68, 2H, m
6a	2.75, 1H, brd, 16, 4		
6b	3.02, 1H, ddd, 16, 11, 6	3.08 – 2.97, 1H, m	3.09 – 2.97, 1H, m
21b	3.11, 1H, dd, 12, 3	3.29 – 3.11, 3H, m	3.15 – 3.10, 3H, m
5b	3.12, 1H, dd, 12, 6		
14a	3.17, 1H, dt, 12, 3		
3	3.37, 1H, brd, 12	3.40, 1H, d, 10.8	3.41, 1H, d, 11.2
OMe	3.74, 3H, s	3.74, 3H, s	3.74, 3H, s
19	3.86, 1H, dq, 12, 6	3.92 – 3.84, 1H, m	3.88, 1H, dq, 10.0, 6.3
10	7.07, 1H	7.08, 1H, dd, 7.2, 6.8	7.08, 1H, td, 7.4, 1.2
11	7.12, 1H	7.14, 1H, dd, 7.2, 6.8	7.14, 1H, td, 7.5, 1.4
12	7.28, 1H	7.30, 1H, d, 8.0	7.30, 1H, d, 8.0
9	7.46, 1H	7.47, 1H, d, 7.6	7.47, 1H, d, 7.7
17	7.56, 1H, d, 1.5	7.56, 1H, d, 1.2	7.56, 1H, d, 1.5
NH	8.06, 1H, brs	7.98, 1H, s	7.94, 1H, s

Table S6: Chemical shifts of ^{13}C NMR spectra for the natural,[10] reported synthetic[9] and our synthetic (+)-mayumbine **2** ($CDCl_3$).

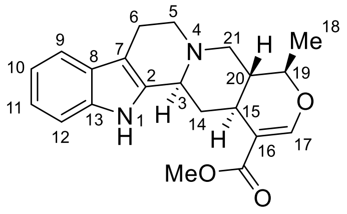

2 (+)-Mayumbine

Carbon	δC (ppm) natural[10]	δC (ppm) synthetic,[9] 150 MHz	δC (ppm) our synthetic, 201 MHz
18 (Me)	18.2	18.4	18.5
6	21.8	21.8	21.9
14	32.8	32.8	33.0
15	36.5	36.5	36.7
20	43.6	43.7	43.8
OMe	50.8	51.0	51.1
5	53.2	53.3	53.5
21	56.2	56.3	56.4
3	59.6	59.7	59.8
19	75.5	75.6	75.7
16	107.8	107.9	108.0
7	107.9	107.9	108.1
12	110.8	110.8	111.0
9	117.9	118.0	118.2
10	119.2	119.3	119.5
11	121.2	121.4	121.6
8	127.3	127.3	127.4
2	134.4	134.4	134.6
13	136.0	135.9	136.1
17	155.9	156.0	156.1
C=O	167.3	167.5	167.6

c) (-)-Roxburghine C total synthesis

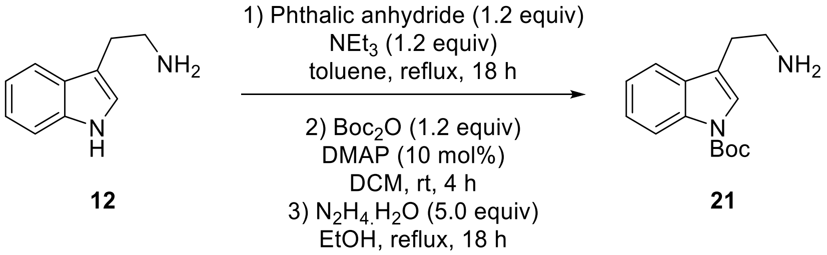

Tert-butyl 3-(2-aminoethyl)-1*H*-indole-1-carboxylate **21** (8.5 g, 32.6 mmol, 82%) was synthesized in 3 steps from tryptamine **12** (6.4 g, 40 mmol, 1.0 equiv) according to a reported procedure.[11] All characterization data were in full agreement with those reported in the literature.[12]

tert-butyl 3-(2-aminoethyl)-1*H*-indole-1-carboxylate (21)

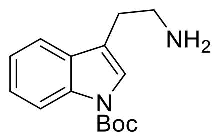

21

Yellow thick oil.

Rf = 0.5 (9/1 DCM/MeOH + 2% NEt_3).

^{1}H NMR (400 MHz, $CDCl_3$) δ 8.13 (d, *J* = 8.3 Hz, 1H), 7.54 (dt, *J* = 7.7, 1.0 Hz, 1H), 7.42 (s, 1H), 7.32 (ddd, *J* = 8.4, 7.1, 1.3 Hz, 1H), 7.23 (td, *J* = 7.6, 1.2 Hz, 1H), 3.05 (t, *J* = 6.7 Hz, 2H), 2.85 (t, *J* = 6.7 Hz, 2H), 1.67 (s, 9H), 1.51 (s, 2H).

^{13}C NMR (101 MHz, $CDCl_3$) δ 149.9, 135.8, 130.7, 124.5, 123.3, 122.5, 119.1, 118.4, 115.5, 83.6, 41.7, 29.3, 28.4.

HRMS (ESI/QTOF) m/z: $[M + H]^+$ Calcd for $C_{15}H_{21}N_2O_2^+$ 261.1598; Found 261.1601.

IR (ν_{max}, cm^{-1}) 2977 (w), 2931 (w), 1725 (s), 1452 (m), 1368 (s), 1253 (s), 1154 (s), 1087 (s), 1015 (m), 854 (m), 765 (m), 744 (s).

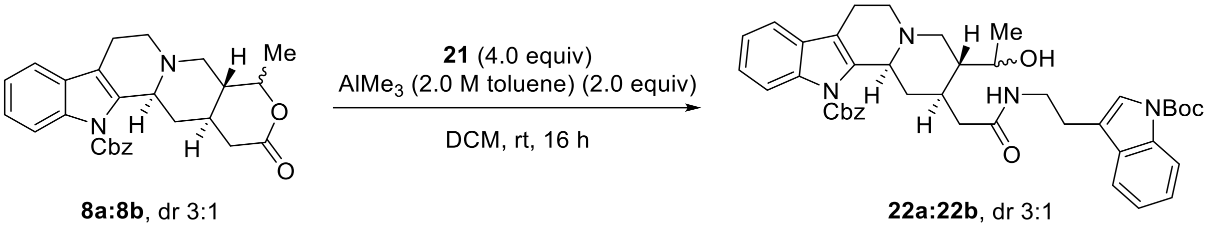

8a:8b, dr 3:1 **22a:22b**, dr 3:1

$AlMe_3$ (2.0 M in toluene, 2.9 mL, 5.8 mmol, 2.0 equiv) was added dropwise to a stirred solution of N_1-Boc tryptamine **21** (3.0 g, 11.7 mmol, 4.0 equiv) in dry DCM (15 mL) at rt under Ar. The deep yellow solution was then stirred at rt for 45 minutes and was then cooled to 0 °C. A solution of lactone **8** (1.3 g, 2.9 mmol, 1.0 equiv) in dry DCM (30 mL, 0.1 M) was then added dropwise to the **21**-Al complex solution, and the mixture was then stirred at rt for 16 h. The reaction mixture was then poured into aqueous Na_2CO_3 solution, and was extracted with DCM three times. The combined organic layers were washed with brine, dried over $MgSO_4$, filtered and concentrated under reduced pressure. The mixture was purified by FCC (SiO_2, 1/3 acetone/hexane + 2% NEt_3 to 7/3 acetone/hexane + 2% NEt_3) to give a combined mixture of two separable diastereoisomers (dr 3:1) of alcohol **22a:22b** (1.5 g, 2.1 mmol, 73% yield) as a light yellow foam. Under these purification conditions, the two diastereomers were separated for characterization.

benzyl (2*R*,3*R*,12b*S*)-2-(2-((2-(1-(*tert*-butoxycarbonyl)-1*H*-indol-3-yl)ethyl)amino)-2-oxoethyl)-3-((*S*)-1-hydroxyethyl)-1,3,4,6,7,12b-hexahydroindolo[2,3-a]quinolizine-12(2*H*)-carboxylate (22a)

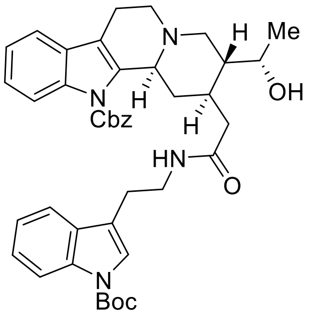

22a

Light yellow foam.

Rf = 0.35 (3/2 acetone/hexane + 2% NEt_3).

^{1}H NMR (600 MHz, $CDCl_3$) δ 8.13 – 8.09 (m, 1H), 8.09 (d, *J* = 8.0 Hz, 1H), 7.54 (d, *J* = 7.8 Hz, 1H), 7.48 – 7.45 (m, 2H), 7.44 (s, 1H), 7.39-7.36 (m, 3H), 7.35 – 7.30 (m, 2H), 7.25 – 7.20 (m, 3H), 5.77 (t, *J* = 5.9 Hz, 1H), 5.46 (d, *J* = 11.8 Hz, 1H), 5.32 (d, *J* = 11.8 Hz, 1H), 3.99 (d, *J* = 10.6 Hz, 1H), 3.82 (q, *J* = 7.1 Hz, 2H), 3.67 – 3.56 (m, 2H), 3.05-3.00 (m, 2H), 2.92 (t, *J* = 6.9 Hz, 2H), 2.80-2.74 (m, 2H), 2.73 – 2.65 (m, 2H), 2.15 – 2.02 (m, 3H), 1.96 – 1.91 (m, 1H), 1.66 (s, 9H), 1.55 – 1.50 (m, 1H), 1.32 (q, *J* = 11.7 Hz, 1H), 1.16 (d, *J* = 6.3 Hz, 3H).

^{13}C NMR (151 MHz, $CDCl_3$) δ 173.1, 151.6, 149.9, 136.81, 136.8, 135.7, 134.9, 130.5, 129.6, 129.2, 129.0, 129.0, 124.8, 124.4, 123.3, 123.2, 122.8, 119.0, 118.2, 117.7, 116.8, 115.7, 115.5, 83.9, 69.0, 66.2, 58.8, 54.7, 47.5, 44.8, 41.5, 39.4, 35.4, 33.3, 28.4, 25.3, 22.4, 19.5.

HRMS (ESI/QTOF) m/z: $[M + H]^+$ Calcd for $C_{42}H_{49}N_4O_6^+$ 705.3647; Found 705.3662.

IR (ν_{max}, cm^{-1}) 3293 (w), 2971 (w), 2925 (w), 2854 (w), 1728 (s), 1645 (m), 1547 (w), 1455 (s), 1370 (s), 1353 (s), 1309 (s), 1255 (s), 1215 (s), 1159 (s), 1090 (m), 1017 (m), 858 (w), 747 (s), 699 (m).

$[\alpha]_D^{24}$ = -50 (*c* 0.4, $CHCl_3$).

benzyl (2*R*,3*R*,12b*S*)-2-(2-((2-(1-(*tert*-butoxycarbonyl)-1*H*-indol-3-yl)ethyl)amino)-2-oxoethyl)-3-((R)-1-hydroxyethyl)-1,3,4,6,7,12b-hexahydroindolo[2,3-a]quinolizine-12(2*H*)-carboxylate (22b)

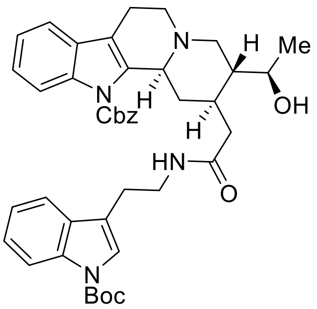

22b

Light yellow foam.

Rf = 0.24 (6/4 acetone/hexane + 2% NEt_3).

^{1}H NMR (400 MHz, $CDCl_3$) δ 8.13 – 8.08 (m, 1H), 8.09 – 8.05 (m, 1H), 7.52 (dt, *J* = 7.7, 1.0 Hz, 1H), 7.48 – 7.44 (m, 2H), 7.42 (s, 1H), 7.40 – 7.32 (m, 4H), 7.32 – 7.27 (m, 1H), 7.25 – 7.18 (m, 3H), 5.84 (t, *J* = 5.9 Hz, 1H), 5.42 (d, *J* = 11.8 Hz, 1H), 5.36 (d, *J* = 11.8 Hz, 1H), 4.03 (d, *J* = 10.6 Hz, 1H), 3.82 (p, *J* = 6.3 Hz, 1H), 3.63-3.48 (m, 2H), 3.15 (dd, *J* = 12.8, 3.8 Hz, 1H), 3.10 – 3.01 (m, 1H), 2.92 – 2.85 (m, 2H), 2.78 – 2.66 (m, 3H), 2.59 (t, *J* = 12.1 Hz, 1H), 2.31 (dd, *J* = 14.2, 6.1 Hz, 1H), 2.12 (dd, *J* = 14.2, 4.2 Hz, 1H), 2.00 (dt, *J* = 12.7, 3.0 Hz, 1H), 1.86 – 1.76 (m, 1H), 1.76 – 1.68 (m, 1H), 1.65 (s, 9H), 1.46 – 1.32 (m, 1H), 1.14 (d, *J* = 6.4 Hz, 3H).

^{13}C NMR (101 MHz, $CDCl_3$) δ 172.2, 151.5, 149.9, 136.6, 136.4, 135.6, 135.0, 130.5, 129.5, 129.1, 128.9, 128.9, 124.7, 124.4, 123.3, 123.2, 122.7, 119.1, 118.2, 117.8, 116.6, 115.7, 115.5, 83.9, 69.0, 68.9, 58.6, 56.0, 46.9, 42.8, 41.3, 39.2, 37.3, 34.6, 28.4, 25.3, 22.2, 20.1.

HRMS (ESI/QTOF) m/z: $[M + H]^+$ Calcd for $C_{42}H_{49}N_4O_6^+$ 705.3647; Found 705.3662.

IR (ν_{max}, cm^{-1}) 3309 (w), 2972 (w), 2926 (w), 1728 (s), 1649 (m), 1455 (s), 1380 (s), 1371 (s), 1352 (s), 1309 (s), 1254 (s), 1214 (s), 1157 (s), 1090 (m), 1016 (m), 745 (s), 698 (m), 665 (m).

$[\alpha]_D^{24}$ = -3.6 (*c* 0.2, $CHCl_3$).

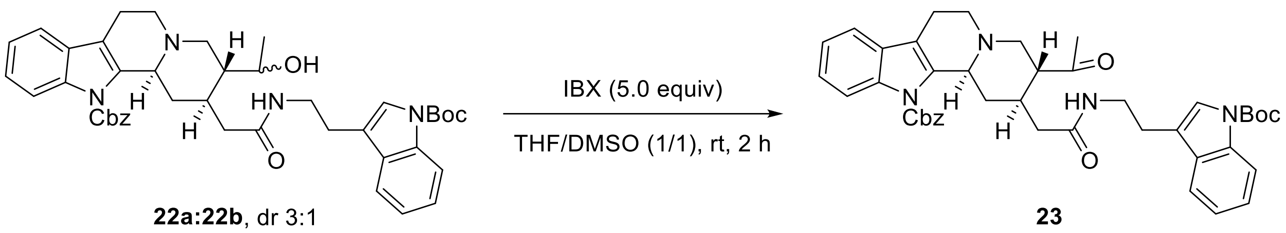

22a:22b, dr 3:1 **23**

To a solution of alcohol **22** (1.5 g, 2.1 mmol, 1.0 equiv) in a 1/1 mixture of THF/DMSO (43 mL, 0.05 M) was added freshly prepared IBX* (3.0 g, 10.5 mmol, 5.0 equiv) portionwise at rt under Ar. The solution was stirred at rt for 2 h. The solution was poured into aqueous Na_2CO_3 solution and diluted with water. The mixture was extracted with EtOAc four times. The combined organic layers were washed with brine, dried over $MgSO_4$, filtered and the filtrate was concentrated under reduced pressure. The crude product was used for the next step without further purification. An analytical sample of **23** was obtained as a brown foam by prep-TLC (1/1 acetone/hexane) for characterization

*Fresh IBX was prepared according to reported procedure.[13]

benzyl (2*R*,3*R*,12b*S*)-3-acetyl-2-(2-((2-(1-(*tert*-butoxycarbonyl)-1*H*-indol-3-yl)ethyl)amino)-2-oxoethyl)-1,3,4,6,7,12b-hexahydroindolo[2,3-a]quinolizine-12(2*H*)-carboxylate (23)

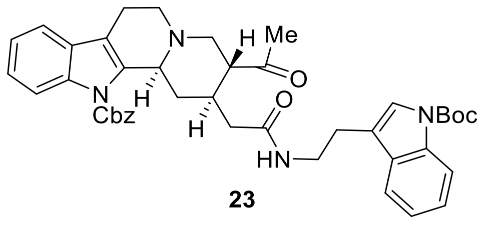

23

Brown foam.

Rf = 0.48 (1/1 acetone/hexane).

^{1}H NMR (400 MHz, $CDCl_3$) δ 8.16 – 8.10 (m, 1H), 8.10 – 8.06 (m, 1H), 7.54 (dt, *J* = 7.7, 1.1 Hz, 1H), 7.48 – 7.43 (m, 3H), 7.41 – 7.32 (m, 4H), 7.30 (ddd, *J* = 8.4, 7.2, 1.3 Hz, 1H), 7.26 – 7.18 (m, 3H), 5.60 (t, *J* = 5.8

Hz, 1H), 5.45 (d, J = 11.9 Hz, 1H), 5.37 (d, J = 11.9 Hz, 1H), 4.23 (d, J = 10.8 Hz, 1H), 3.62 – 3.45 (m, 2H), 3.24 (dd, J = 12.6, 3.6 Hz, 1H), 3.17 – 3.08 (m, 1H), 3.01 – 2.92 (m, 1H), 2.92 – 2.84 (m, 3H), 2.83 – 2.72 (m, 3H), 2.19 (s, 3H), 2.24 – 2.12 (m, 1H), 2.06 (dt, J = 12.9, 3.0 Hz, 1H), 1.99 – 1.94 (m, 2H), 1.65 (s, 9H), 1.46 – 1.33 (m, 1H).

^{13}C NMR (101 MHz, $CDCl_3$) δ 211.1, 171.1, 151.4, 149.8, 136.4, 136.4, 135.7, 135.1, 130.5, 129.4, 129.1, 128.9, 124.7, 124.5, 123.4, 123.2, 122.7, 119.1, 118.2, 117.8, 116.1, 115.9, 115.5, 83.7, 68.9, 57.4, 51.0, 45.5, 41.3, 39.2, 35.4, 31.5, 29.8, 28.4, 25.4, 22.2.

HRMS (ESI/QTOF) m/z: $[M + H]^+$ Calcd for $C_{42}H_{47}N_4O_6^+$ 703.3490; Found 703.3487.

IR (ν_{max}, cm^{-1}) 2977 (w), 2925 (w), 2852 (w), 1728 (s), 1653 (m), 1455 (s), 1370 (s), 1352 (s), 1309 (s), 1255 (s), 1216 (m), 1156 (s), 1090 (m), 1016 (m), 909 (m), 745 (s), 731 (s), 699 (m).

$[\alpha]_D^{24}$ = -3.1 (c 0.2, $CHCl_3$).

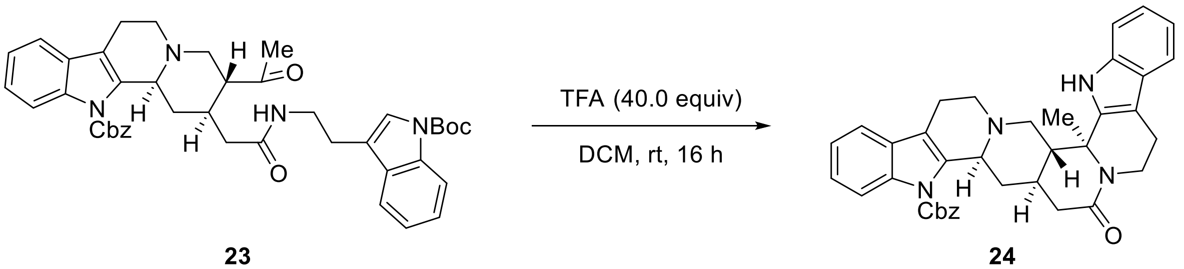

23 **24**

To a solution of the crude ketone **23** (1.1 g, 1.6 mmol, 1.0 equiv) in DCM (25 mL) was added TFA (5.0 mL, 64.0 mmol, 40.0 equiv) at rt and it was stirred at this temperature for 16 h. The mixture was then poured into saturated aqueous solution of Na_2CO_3 and it was extracted three times with EtOAc. The combined organic layers were washed with brine, dried over anhydrous $MgSO_4$, then filtered and concentrated under reduced pressure. The crude product was purified by FCC (SiO_2, 4/6 acetone/hexane) to give the octacyclic product **24** (dr > 20:1) (640 mg, 1.1 mmol, 68% yield over 2 steps) as a beige solid.

benzyl (5b*S*,6a*R*,16b*S*,16c*R*)-16b-methyl-8-oxo-6,6a,7,8,10,11,16,16b,16c,17,19,20-dodecahydroindolo[2',3':3,4]pyrido[2,1-a]indolo[2',3':3,4]pyrido[2,1-g][2,7]naphthyridine-5(5b*H*)-carboxylate (24)

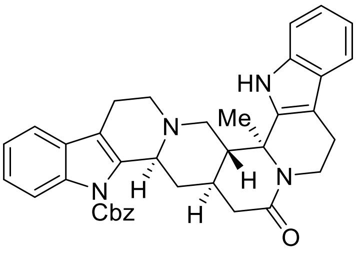

24, dr > 20:1

Beige solid.

Rf = 0.43 (4/6 acetone/hexane).

^{1}H NMR (400 MHz, $CDCl_3$) δ 8.25 (s, 1H), 8.14 (d, *J* = 8.0 Hz, 1H), 7.53 (d, *J* = 7.7 Hz, 1H), 7.51 – 7.47 (m, 2H), 7.47 – 7.36 (m, 5H), 7.31 – 7.26 (m, 1H), 7.26 – 7.19 (m, 2H), 7.15 (td, *J* = 7.5, 1.0 Hz, 1H), 5.49 (d, *J* = 11.6 Hz, 1H), 5.32 (d, *J* = 11.7 Hz, 1H), 5.20 – 5.12 (m, 1H), 4.12 (d, *J* = 10.6 Hz, 1H), 3.67 (dd, *J* = 12.1, 4.0 Hz, 1H), 3.13 – 3.04 (m, 2H), 2.91 – 2.63 (m, 6H), 2.50 (dd, *J* = 18.1, 6.3 Hz, 1H), 2.11 – 1.97 (m, 3H), 1.74 – 1.62 (m, 1H), 1.52 (s, 3H), 1.31 – 1.20 (m, 1H).

^{13}C NMR (101 MHz, $CDCl_3$) δ 168.9, 151.4, 137.4, 136.5, 136.2, 136.1, 134.8, 129.3, 129.2, 129.2, 129.0, 126.5, 124.6, 123.3, 122.4, 120.0, 118.6, 118.2, 116.6, 115.8, 111.1, 111.0, 69.2, 60.2, 57.5, 56.6, 46.8, 43.3, 38.4, 37.8, 34.8, 30.4, 22.3, 21.9, 21.6.

HRMS (ESI/QTOF) m/z: $[M + H]^+$ Calcd for $C_{37}H_{37}N_4O_3^+$ 585.2860; Found 585.2872.

IR (ν_{max}, cm^{-1}) 3265 (w), 2919 (w), 2848 (w), 1729 (s), 1610 (s), 1456 (s), 1393 (s), 1347 (m), 1315 (s), 1262 (m), 1240 (m), 1214 (m), 1162 (m), 1115 (m), 1025 (m), 741 (s), 699 (m).

M.p. = 195 – 197 °C.

$[\alpha]_D^{24}$ = -30 (*c* 0.2, $CHCl_3$).

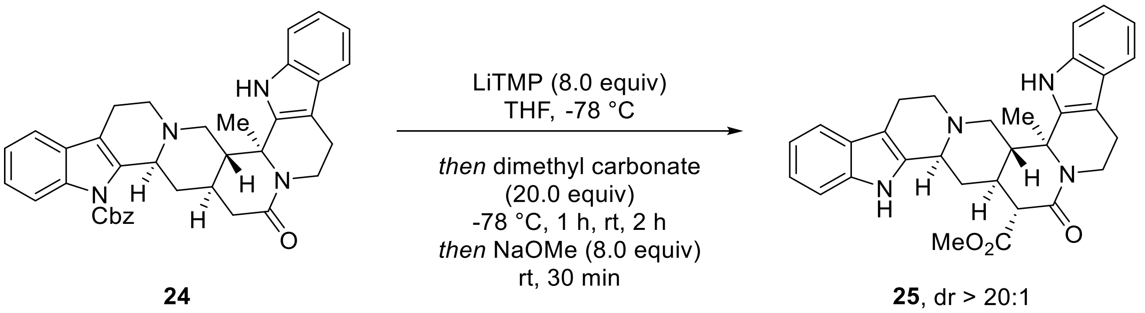

At -78 °C, a solution of lactam **24** (600 mg, 1.0 mmol, 1.0 equiv) in THF (7.0 mL) was added dropwise to a freshly prepared LiTMP solution in THF (TMP (1.7 mL, 10.2 mmol, 10.0 equiv) in THF (14 mL) with BuLi (2.5 M in hexanes, 3.3 mL, 8.2 mmol, 8.0 equiv) at -78 °C for 45 minutes). The reaction was stirred at -78 °C for 1 h before dropwise addition of anhydrous dimethyl carbonate (1.7 mL, 20.4 mmol, 20.0 equiv). The reaction mixture was stirred at -78 °C for 1 h, then was let slowly warm up to rt over 30 minutes and it was stirred for 2 more hours at this temperature. NaOMe (5.4 M in MeOH, 1.5 mL, 8.2 mmol, 8.0 equiv) was slowly added and the mixture was stirred at rt for 30 minutes. The mixture was then poured into $NaHCO_3$ aqueous solution, basified with aqueous Na_2CO_3 solution and extracted 3 times with EtOAc. The combined organic layers were washed with brine, dried over $MgSO_4$, and concentrated under reduced pressure. The residue was purified by FCC (SiO_2, hexane to EtOAc/hexane 7/3) to afford the desired *β*-amidoester **25** (dr > 20:1) (390 mg, 0.77 mmol, 75% yield) as a white solid. Crystals were obtained by slow evaporation from a 1/3/3 DCM/acetone/hexane mixture.

methyl (5b*S*,6a*S*,7*S*,16b*S*,16c*R*)-16b-methyl-8-oxo-5,5b,6,6a,7,8,10,11,16,16b,16c,17,19,20-tetradecahydroindolo[2',3':3,4]pyrido[2,1-a]indolo[2',3':3,4]pyrido[2,1-g][2,7]naphthyridine-7-carboxylate (25)

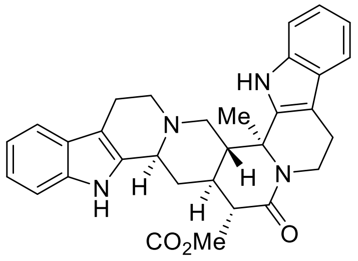

25

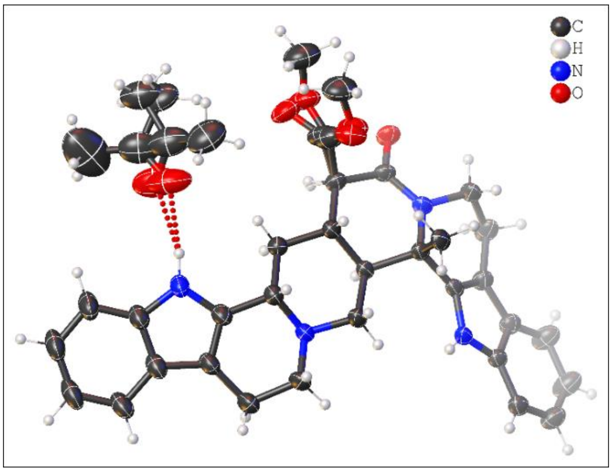

X-Ray structure of 25 (50% ellipsoid probability level)

White solid.

Rf = 0.32 (6/4 EtOAc/hexane).

^{1}H NMR (400 MHz, $CDCl_3$) δ 7.79 (s, 1H), 7.75 (s, 1H), 7.53 (d, *J* = 7.8 Hz, 1H), 7.46 (d, *J* = 7.7 Hz, 1H), 7.39 (d, *J* = 8.1 Hz, 1H), 7.31 (d, *J* = 8.0 Hz, 1H), 7.23 (ddd, *J* = 8.2, 7.1, 1.1 Hz, 1H), 7.18 – 7.13 (m, 2H),

7.09 (td, J = 7.5, 1.1 Hz, 1H), 5.11 (dt, J = 12.3, 3.2 Hz, 1H), 3.88 (s, 3H), 3.59 (dd, J = 10.4, 4.0 Hz, 1H), 3.42 (dd, J = 11.0, 2.5 Hz, 1H), 3.30 (d, J = 10.8 Hz, 1H), 3.17 – 3.10 (m, 1H), 2.98-2.87 (m, 2H), 2.80 – 2.71 (m, 5H), 2.53 (qd, J = 11.4, 4.2 Hz, 1H), 2.31 – 2.18 (m, 2H), 1.74 (s, 3H), 1.48 (q, J = 11.8 Hz, 1H).

^{13}C NMR (101 MHz, $CDCl_3$) δ 171.3, 165.1, 136.4, 136.3, 136.1, 133.5, 127.2, 126.5, 122.8, 121.9, 120.3, 119.8, 118.8, 118.3, 111.5, 111.1, 111.0, 108.7, 60.2, 58.7, 57.2, 55.8, 53.6, 53.0, 46.0, 38.4, 36.1, 34.2, 22.5, 22.0, 21.8.

HRMS (ESI/QTOF) m/z: $[M + H]^+$ Calcd for $C_{31}H_{33}N_4O_3^+$ 509.2547; Found 509.2555.

IR (ν_{max}, cm^{-1}) 3355 (w), 2922 (w), 2849 (w), 1735 (w), 1721 (w), 1612 (m), 1453 (m), 1425 (m), 1349 (w), 1296 (m), 1260 (m), 1234 (m), 1155 (m), 737 (s), 700 (m), 678 (m).

M.p. = 326 – 328 °C (decomposition).

$[\alpha]_D^{24}$ = -250 (c 0.2, $CHCl_3$).

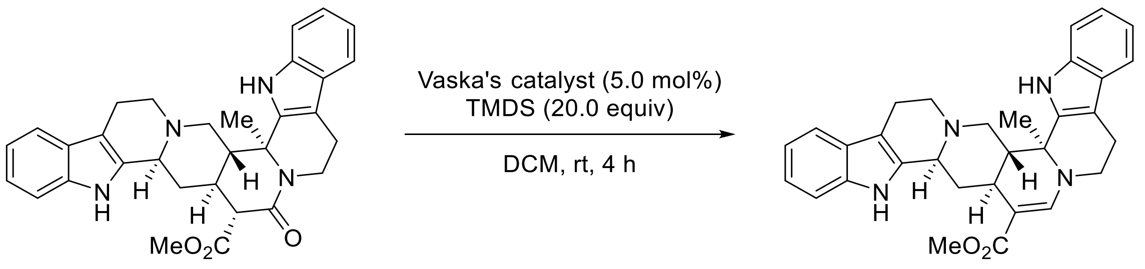

25 **3 (-)-Roxburghine C**

To a flask purged with argon containing **25** (220 mg, 0.43 mmol, 1.0 equiv) and Vaska’s catalyst (17 mg, 22 µmol, 5.0 mol%) was added dry and degassed DCM (8.6 mL). The mixture was stirred until the solution was homogeneous. TMDS (1.5 mL, 8.6 mmol, 20.0 equiv) was slowly added to the reaction mixture and it was stirred at rt for 4 h under argon. The mixture was then concentrated under reduced pressure. The residue was purified by FCC (SiO_2, 3/7 acetone/hexane) to afford (-)-roxburghine C **3** (191 mg, 0.39 mmol, 91% yield) as a white solid. All characterization data were in full agreement with those reported in the literature.[14,15]

(-)-Roxburghine C (3)

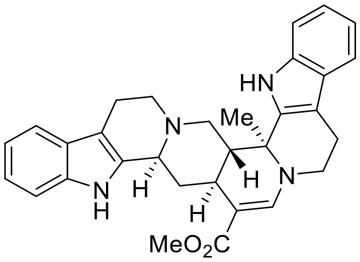

3 (-)-Roxburghine C

White solid.

Rf = 0.35 (3/7 acetone/hexane).

^{1}H NMR (600 MHz, Acetone-d_6) δ 10.20 (s, 1H), 10.19 (s, 1H), 7.59 (s, 1H), 7.50 (d, *J* = 7.8 Hz, 1H), 7.39 (dt, *J* = 8.1, 0.9 Hz, 1H), 7.34 (d, *J* = 7.8 Hz, 1H), 7.32 (dt, *J* = 8.1, 0.9 Hz, 1H), 7.12 (ddd, *J* = 8.2, 7.0, 1.2 Hz, 1H), 7.04 (ddd, *J* = 8.0, 7.1, 1.0 Hz, 1H), 7.00 (ddd, *J* = 8.1, 7.0, 1.2 Hz, 1H), 6.94 (ddd, *J* = 7.9, 7.0, 1.1 Hz, 1H), 3.68 (ddd, *J* = 12.5, 4.8, 1.7 Hz, 1H), 3.63 (s, 3H), 3.65 – 3.60 (m, 1H), 3.54 (dd, *J* = 10.6, 3.2 Hz, 1H), 3.48 (dt, *J* = 12.5, 3.1 Hz, 1H), 3.40 (dq, *J* = 11.4, 2.3 Hz, 1H), 3.02 (dd, *J* = 10.3, 5.7 Hz, 1H), 2.93 – 2.80 (m, 2H), 2.77 (dddd, *J* = 14.0, 11.1, 5.7, 2.3 Hz, 1H), 2.67 – 2.64 (m, 1H), 2.64 – 2.61 (m, 2H), 2.61 – 2.58 (m, 1H), 1.83 (td, *J* = 11.2, 3.1 Hz, 1H), 1.51 (s, 3H), 1.08 (dt, *J* = 12.6, 11.4 Hz, 1H).

^{13}C NMR (151 MHz, Acetone-d_6) δ 167.7, 147.0, 137.6, 137.5, 137.4, 136.6, 128.3, 127.5, 122.4, 121.3, 119.9, 119.4, 119.0, 118.3, 112.0, 111.8, 110.1, 107.9, 96.3, 60.8, 58.6, 58.1, 54.2, 50.8, 50.2, 50.1, 36.2, 35.4, 23.4, 22.8, 18.7.

HRMS (ESI/QTOF) m/z: $[M + H]^+$ Calcd for $C_{31}H_{33}N_4O_2^+$ 493.2598; Found 493.2607.

IR (ν_{max}, cm^{-1}) 3308 (w), 2920 (w), 2849 (w), 1694 (m), 1659 (m), 1606 (s), 1454 (m), 1437 (m), 1410 (m), 1349 (m), 1317 (s), 1294 (m), 1216 (s), 1181 (m), 1162 (m), 1110 (s), 1039 (w), 1008 (w), 741 (s).

M.p. = 247 – 250 °C (deep color change to reddish at 232 °C); lit.[14] M.p. = 245 – 250 °C.

$[\alpha]_D^{20}$ = -242 (*c* 0.255, MeOH); lit.[14] $[\alpha]_D^{20}$ = -221° (*c* 0.253, MeOH).

Table S1: Chemical shifts of ^{1}H NMR spectra for the natural[14] and the synthetic (-)-roxburghine C **3** (acetone-d_6).

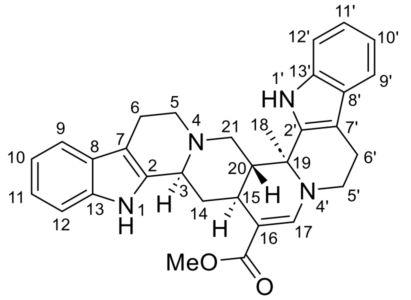

S13, **(-)-Roxburghine C**

**Proton**	**δH (ppm) natural,[14] multi (*J*, Hz)**	**Our δH (ppm) synthetic, multi (*J*, Hz)**
18 (Me)	1.68	1.51, s
3	1.2 - 3.8 (15 H)	3.40, dq (11.4, 2.3)
5		2.64 – 2.67, m + 3.02, dd (10.3, 5.7)
6		2.58 – 2.61, m + 2.77, dddd (14.0, 11.1, 5.7, 2.3)
5'		3.60 – 3.65, m + 3.68 ddd (12.5, 4.8, 1.7)
6'		2.80 – 2.93, m
14		1.08, dt (12.6, 11.4) + 3.48, dt (12.5, 3.1)
15		2.58 – 2.61, m
20		1.83, td (11.2, 3.1)
21		2.61 – 2.64, m + 3.54, dd (10.6, 3.2)
OMe	3.73	3.63, s
17	7.55	7.59, s
10	6.8 - 7.5 (8H indole)	6.94, ddd (7.9, 7.0, 1.1)
11		7.00, ddd (8.1, 7.0, 1.2)
10'		7.04, ddd (8.0, 7.1, 1.0)
11'		7.12, ddd (8.2, 7.0, 1.2)
12		7.32, dt (8.1, 0.9)
9		7.34, d (7.8)
12'		7.39, dt (8.1, 0.9)
9'		7.50, d (7.8)
1	8.15	10.20, s
1'	8.28	10.19, s

Table S2: Chemical shifts of ^{13}C NMR spectra for the natural[15] and the synthetic (-)-roxburghine C (**S13**) (acetone-d_6). **It seems not evident to assign precisely the two carbons at 50.1 and 50.2 ppm.*

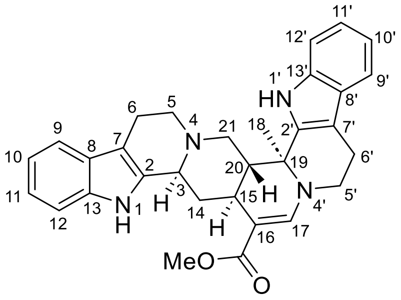

S13, (-)-Roxburghine C

Carbon	δC (ppm) natural[15]	Our δC (ppm) synthetic
Me (18)	18.7	18.7
6	23.3 or 22.7	22.8
6'	22.7 or 23.3	23.4
14	35.3	35.4
15	36.1	36.2
20	50.0	50.1 or 50.2*
OMe	50.1	50.2 or 50.1*
5'	50.7	50.8
5	54.0	54.2
21	57.9	58.1
19	58.5	58.6
3	60.6	60.8
16	96.2	96.3
7	109.9	107.9
7'	107.7	110.1
12	111.7 or 111.9	111.8
12'	111.7 or 111.9	112.0
9	118.1 or 118.8	118.3
9'	118.1 or 118.8	119.0
10	119.2 or 119.7	119.4
10'	119.2 or 119.7	119.9
11	121.1 or 122.2	121.3
11'	121.1 or 122.2	122.4

8'	127.3 or 128.1	127.5
8	127.3 or 128.1	128.3
2	136.5	136.6
2'	137.2	137.4
13'	137.3	137.5
13	137.4	137.6
17	146.9	147.0
C=O	167.7	167.7

d) Pictet-Spengler reaction selectivity

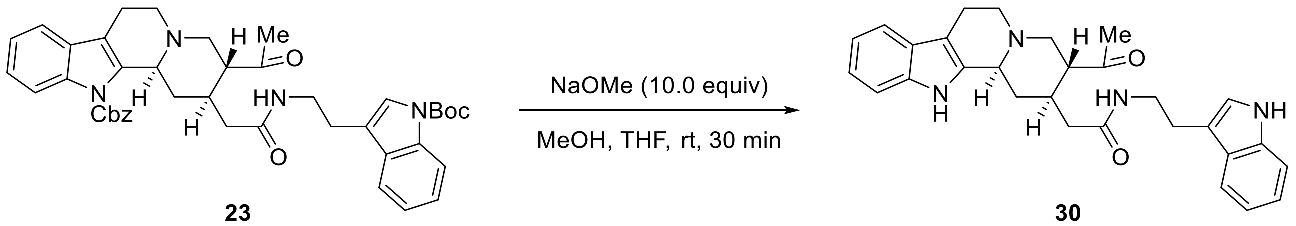

23 30

To a solution of **23** (90 mg, 0.13 mmol, 1.0 equiv) in THF (2.6 mL) was added NaOMe (5.4 M in MeOH, 240 µL, 1.3 mmol, 10.0 equiv) at rt and the reaction mixture was stirred at this temperature for 30 minutes. The mixture was then poured into saturated aqueous solution of Na_2CO_3 and it was extracted four times with EtOAc. The combined organic layers were washed with brine, dried over anhydrous $MgSO_4$, then filtered and concentrated under reduced pressure. The crude product was purified by FCC (SiO_2, 1/15 MeOH/DCM) to give the desired product **30** (60 mg, 0.13 mmol, quantitative yield) as a light brown foam.

N-(2-(1*H*-indol-3-yl)ethyl)-2-((2*R*,3*R*,12b*S*)-3-acetyl-1,2,3,4,6,7,12,12b-octahydroindolo[2,3-a]quinolizin-2-yl)acetamide (30)

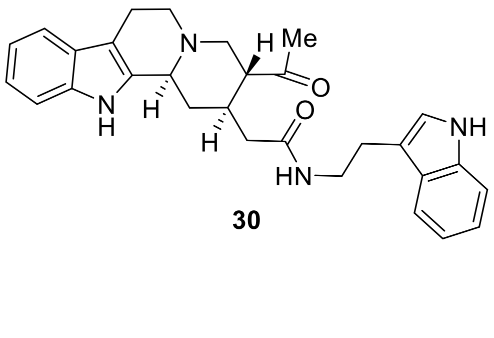

30

Light-brown foam.

Rf = 0.3 (1/15 MeOH/DCM).

^{1}H NMR (400 MHz, $CDCl_3$) δ 8.25 (s, 1H), 8.22 (s, 1H), 7.56 (d, *J* = 7.8 Hz, 1H), 7.46 (d, *J* = 7.5 Hz, 1H), 7.24 – 7.19 (m, 2H), 7.17 – 7.11 (m, 2H), 7.11 – 7.06 (m, 2H), 6.93 (d, *J* = 2.3 Hz, 1H), 5.73 (t, *J* = 5.8 Hz, 1H), 3.60 (dq, *J* = 12.8, 6.4 Hz, 1H), 3.48 (dq, *J* = 12.7, 6.4 Hz, 1H), 3.19 – 3.09 (m, 2H), 3.07 – 3.00 (m, 1H), 3.00 – 2.89 (m, 1H), 2.92 (t, *J* = 6.6 Hz, 2H), 2.79 – 2.67 (m, 2H), 2.59 (td, *J* = 10.9, 4.3 Hz, 1H), 2.28 (dd, *J* = 13.4, 9.5 Hz, 2H), 2.16 (s, 3H), 2.20 – 2.06 (m, 2H), 1.79 (dd, *J* = 14.1, 9.1 Hz, 1H), 1.25 (q, *J* = 12.0 Hz, 1H).

^{13}C NMR (101 MHz, $CDCl_3$) δ 210.2, 171.5, 136.5, 136.2, 134.5, 127.4, 127.2, 122.6, 122.2, 121.5, 119.5, 119.5, 118.7, 118.2, 112.7, 111.5, 111.2, 107.8, 59.0, 57.3, 55.2, 52.6, 41.0, 39.8, 34.6, 34.5, 29.9, 25.4, 21.9.

HRMS (Nanochip-based ESI/LTQ-Orbitrap) m/z: $[M + H]^+$ Calcd for $C_{29}H_{33}N_4O_2^+$ 469.2598; Found 469.2597.

IR (ν_{max}, cm^{-1}) 3402 (w), 3285 (w), 2918 (w), 2846 (w), 2803 (w), 2757 (w), 1701 (m), 1648 (m), 1525 (w), 1454 (m), 1437 (w), 1353 (m), 1321 (w), 1299 (w), 1230 (w), 1162 (w), 1103 (w), 1009 (w), 908 (w), 739 (s).

$[\alpha]_D{}^{24}$ = -16 (*c* 0.2, $CHCl_3$).

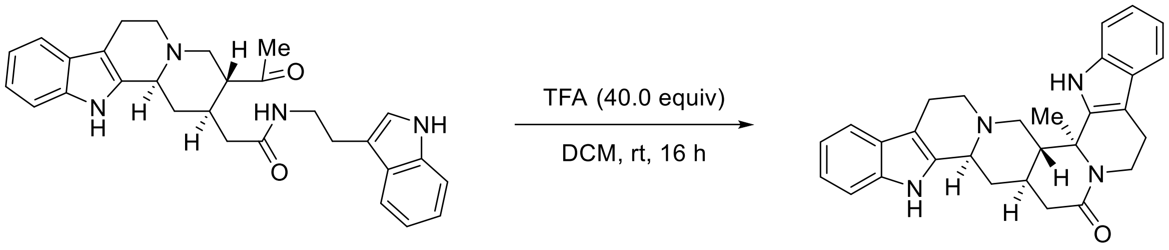

30 **33a**, dr > 20:1

To a solution of the ketone **30** (5.0 mg, 10.7 µmol, 1.0 equiv) in DCM (200 µL) was added TFA (40 µL, 0.54 mmol, 40.0 equiv) at rt and it was stirred at this temperature for 16 h. The mixture was then poured into saturated aqueous solution of Na_2CO_3 and it was extracted three times with EtOAc. The combined organic layers were washed with brine, dried over anhydrous $MgSO_4$, then filtered and concentrated under reduced pressure. The crude product was purified by prep-TLC (SiO_2, 1/15 MeOH/DCM) to give the octacyclic product **33a** (dr > 20:1) (3.1 mg, 6.9 µmol, 65% yield) as a white powder.

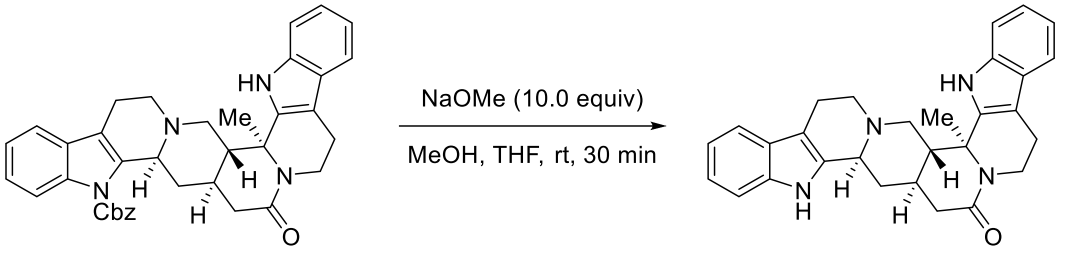

24 **33a**

To a solution of **24** (11.7 mg, 20.0 µmol, 1.0 equiv) in THF (400 µL) was added NaOMe (5.4 M in MeOH, 37.0 µL, 0.2 mmol, 10.0 equiv) at rt and it was stirred at this temperature for 30 minutes. The mixture was then poured into saturated aqueous solution of Na_2CO_3 and it was extracted three times with EtOAc. The combined organic layers were washed with brine, dried over anhydrous $MgSO_4$, then filtered and concentrated under reduced pressure. The crude product was purified by prep-TLC (SiO_2, 1/15 MeOH/DCM) to give the octacyclic product diastereoisomer **33a** (8.4 mg, 18.6 µmol, 93% yield) as a white powder.

(5b*S*,6a*R*,16b*S*,16c*R*)-16b-methyl-5b,6,6a,7,10,11,16,16b,16c,17,19,20-dodecahydroindolo[2',3':3,4]pyrido[2,1-a]indolo[2',3':3,4]pyrido[2,1-g][2,7]naphthyridin-8(5*H*)-one (33a)

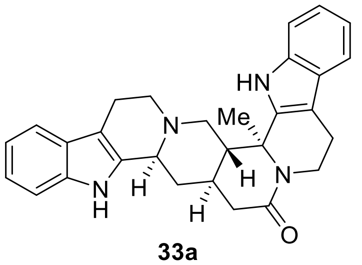

33a

White powder.

Rf = 0.3 (1/15 MeOH/DCM).

^{1}H NMR (600 MHz, Acetone-d_6) δ 9.99 (s, 1H), 9.92 (s, 1H), 7.47 (d, *J* = 7.8 Hz, 1H), 7.37 (t, *J* = 7.9 Hz, 2H), 7.30 (d, *J* = 8.1 Hz, 1H), 7.10 (t, *J* = 7.6 Hz, 1H), 7.03 (td, *J* = 7.6, 2.8 Hz, 2H), 6.96 (t, *J* = 7.4 Hz, 1H), 5.08 (ddd, *J* = 12.6, 4.3, 2.0 Hz, 1H), 3.80 (dd, *J* = 11.2, 3.9 Hz, 1H), 3.41 (d, *J* = 11.6 Hz, 1H), 3.19 – 3.13 (m, 1H), 2.88 – 2.78 (m, 2H), 2.76 (t, *J* = 11.0 Hz, 1H), 2.72 – 2.65 (m, 4H), 2.63 (dd, *J* = 18.0, 5.4 Hz, 1H), 2.49 (dt, *J* = 12.4, 3.5 Hz, 1H), 2.28 – 2.21 (m, 1H), 2.15 – 2.08 (m, 1H), 2.04 – 1.97 (m, 1H), 1.70 (s, 3H), 1.34 – 1.28 (m, 1H).

^{13}C NMR (151 MHz, Acetone-d_6) δ 168.4, 139.2, 137.5, 136.1, 128.2, 127.6, 122.4, 121.5, 119.9, 119.5, 118.9, 118.4, 112.1, 111.8, 110.8, 108.1, 61.2, 60.1, 57.9, 54.1, 48.2, 39.0, 38.0, 37.9, 31.1, 22.8, 22.6, 22.2.

HRMS (ESI/QTOF) m/z: $[M + H]^+$ Calcd for $C_{29}H_{31}N_4O^+$ 451.2492; Found 451.2495.

IR (ν_{max}, cm^{-1}) 3312 (w), 2921 (m), 2848 (w), 1613 (s), 1456 (m), 1442 (m), 1429 (m), 1411 (m), 1348 (m), 1302 (m), 1260 (m), 1234 (w), 1164 (w), 1091 (m), 1065 (m), 1049 (m), 1026 (m), 1011 (m), 801 (m), 742 (s), 694 (m).

M.p. > 250 °C (decomposition).

$[\alpha]_D^{24}$ = -113 (*c* 0.1, $CHCl_3$).

The observed 1D NOE-diff for **33a** showed the Me-18 at α-position

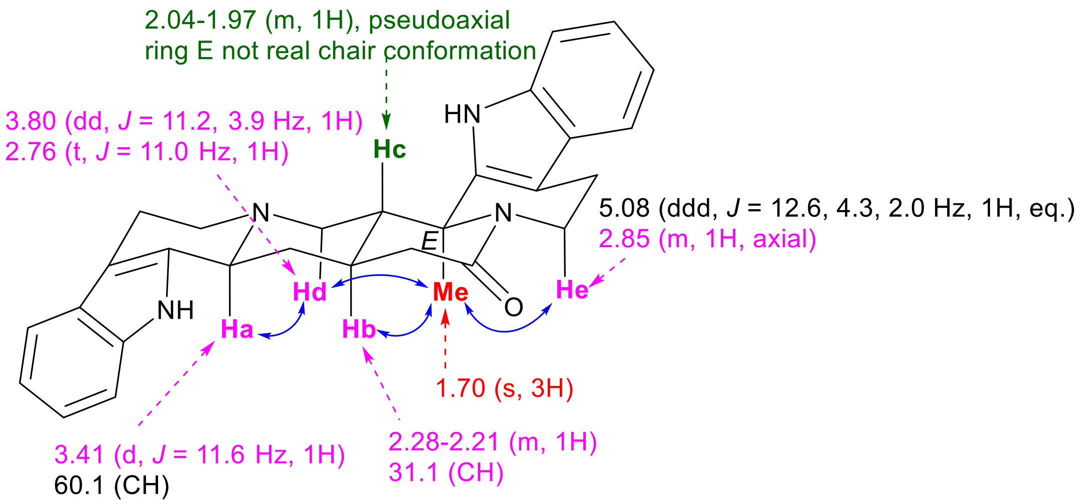

N.B: *^{1}H NMR and ^{13}C NMR in $CDCl_3$ were also measured.*

^{1}H NMR (600 MHz, $CDCl_3$) δ 7.77 (s, 1H), 7.76 (s, 1H), 7.52 (d, *J* = 7.8 Hz, 1H), 7.47 (d, *J* = 7.9 Hz, 1H), 7.39 (d, *J* = 8.1 Hz, 1H), 7.32 (d, *J* = 8.0 Hz, 1H), 7.22 (t, *J* = 7.7 Hz, 1H), 7.17 – 7.13 (m, 2H), 7.10 (t, *J* = 7.4 Hz, 1H), 5.15 (dd, *J* = 12.8, 4.0 Hz, 1H), 3.60 (d, *J* = 10.4 Hz, 1H), 3.44 (d, *J* = 11.2 Hz, 1H), 3.22 – 3.15 (m, 1H), 3.03 – 2.94 (m, 1H), 2.90 (td, *J* = 12.2, 3.4 Hz, 1H), 2.85 – 2.71 (m, 6H), 2.30 (d, *J* = 12.6 Hz, 1H), 2.27-2.16 (m, 3H), 1.65 (s, 3H), 1.48 – 1.40 (m, 1H).

^{13}C NMR (151 MHz, $CDCl_3$) δ 168.7, 137.2, 136.3, 136.1, 133.8, 127.3, 126.6, 122.7, 121.9, 120.2, 119.8, 118.8, 118.4, 111.4, 111.1, 111.0, 108.6, 60.2, 59.0, 57.6, 53.7, 46.8, 38.4, 37.7, 37.2, 30.5, 22.5, 21.9, 21.8.

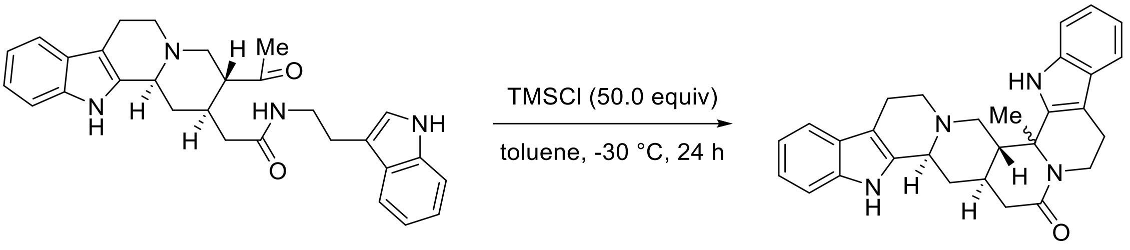

30 **33a:33b**, dr 1:1.2

To a solution of the ketone **30** (5.0 mg, 10.7 µmol, 1.0 equiv) in toluene (500 µL) was slowly added TMSCl (68 µL, 534 µmol, 50.0 equiv) at -30 °C using a cryostat, and the mixture was stirred at this temperature for 24 h. The mixture was then poured into saturated aqueous solution of Na_2CO_3 and it was extracted three times with EtOAc. The combined organic layers were washed with brine, dried over anhydrous $MgSO_4$, then filtered and concentrated under reduced pressure. The crude product was purified by prep-TLC (SiO_2, 1/15 MeOH/DCM) to give a 1:1.2 mixture of inseparable diastereoisomers of octacyclic product **33a:33b** (4.7 mg, 10.6 µmol, 97% yield) as a white powder.

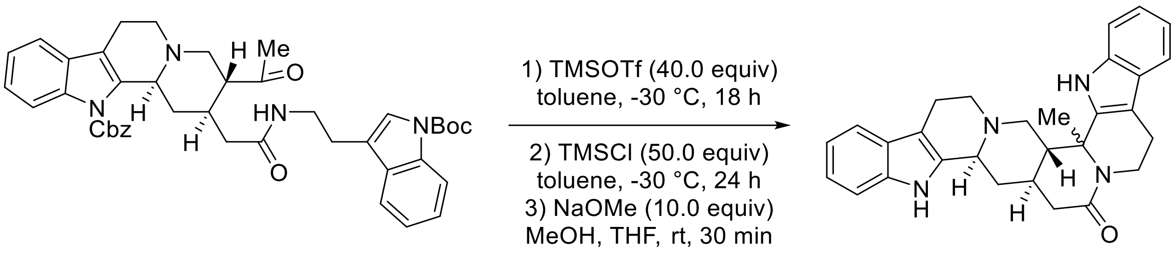

23 **33a:33b**, dr 1:1.2

To a solution of the ketone **23** (6.5 mg, 9.3 µmol, 1.0 equiv) in toluene (500 µL) was slowly added TMSOTf (67 µL, 370 µmol, 40.0 equiv) at -30 °C using a cryostat, and the mixture was stirred at this temperature for 18 h. The mixture was quenched at -30 °C with a saturated aqueous solution of Na_2CO_3 and it was extracted three times with EtOAc. The combined organic layers were washed with brine, dried over anhydrous Na_2SO_4, then filtered and concentrated under reduced pressure. The crude ketone product **29** was used without further purification in the next step.

To a solution of the crude ketone **29** (1.0 equiv) in toluene (500 µL) was slowly added TMSCl (59 µL, 463 µmol, 50.0 equiv) at -30 °C using a cryostat, and the mixture was stirred at this temperature for 24 h. The mixture was then poured into saturated aqueous solution of Na_2CO_3 and it was extracted three times with EtOAc. The combined organic layers were washed with brine, dried over anhydrous Na_2SO_4, then filtered and concentrated under reduced pressure. A 1:1.2 mixture of inseparable diastereoisomers of crude octacyclic

product **24:24b** (5.1 mg, 8.8 µmol, 95% yield) was obtained and used without further purification in the next step.

To a solution of the 1:1.2 mixture of inseparable diastereoisomers of crude octacyclic product **24:24b** (1.0 equiv) in THF (500 µL) was added NaOMe (5.4 M in MeOH, 16.3 µL, 88 µmol, 10.0 equiv) at rt and it was stirred at this temperature for 30 minutes. The mixture was then poured into saturated aqueous solution of Na_2CO_3 and it was extracted three times with EtOAc. The combined organic layers were washed with brine, dried over anhydrous $MgSO_4$, then filtered and concentrated under reduced pressure. The crude product was purified by prep-TLC (SiO_2, 1/15 MeOH/DCM) to give a 1:1.2 mixture of inseparable diastereoisomers of octacyclic product **33a:33b** (3.4 mg, 7.5 µmol, 81% yield over 3 steps) as a white powder.

(5b*S*,6a*R*,16c*R*)-16b-methyl-5b,6,6a,7,10,11,16,16b,16c,17,19,20-dodecahydroindolo[2',3':3,4]pyrido[2,1-a]indolo[2',3':3,4]pyrido[2,1-g][2,7]naphthyridin-8(5*H*)-one (33a:33b)

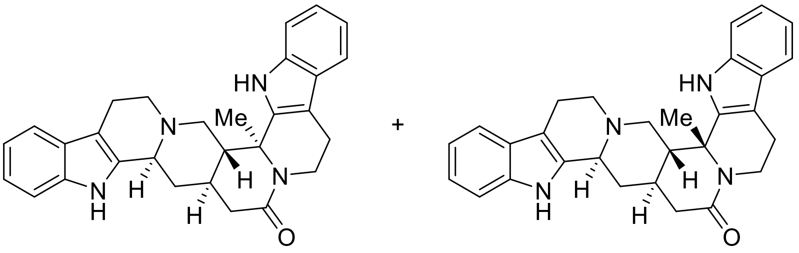

33a **33b**

33a:33b, dr 1:1.2

White powder.

Rf = 0.3 (1/15 MeOH/DCM).

^{1}H NMR (800 MHz, $CDCl_3$) (inseparable 1:1.2 mixture of diastereoisomers) δ 7.83 (s, 0.54H), 7.81 (s, 0.92H), 7.78 (s, 0.54H), 7.53 – 7.50 (m, 1H), 7.49 (d, *J* = 9.0 Hz, 0.54H), 7.47 (d, *J* = 7.9 Hz, 0.46H), 7.39 (d, *J* = 8.1 Hz, 1H), 7.31 (t, *J* = 8.9 Hz, 1H), 7.22 (t, *J* = 7.7 Hz, 1H), 7.17 – 7.12 (m, 2H), 7.12 – 7.08 (m, 1H), 5.16 (dd, *J* = 12.8, 4.6 Hz, 0.46H), 4.99 (dd, *J* = 13.2, 5.2 Hz, 0.54H), 3.59 (d, *J* = 10.1 Hz, 1H), 3.42 (d, *J* = 11.6 Hz, 0.46H), 3.33 (d, *J* = 11.7 Hz, 0.54H), 3.29 (dd, *J* = 10.3, 5.6 Hz, 0.54H), 3.19-3.16 (m, 0.46H), 3.16 – 3.11 (m, 0.54H), 3.08 (m, 0.54H), 3.01 – 2.95 (m, 1H), 2.92 – 2.72 (m, 4.84H), 2.68 – 2.62 (m, 1.08H), 2.33 – 2.28 (m, 1H), 2.27– 2.20 (m, 1.54H), 2.20 – 2.16 (m, 0.92H), 1.80 (d, *J* = 1.9 Hz, 1.62H), 1.84 – 1.77 (m, 0.54H), 1.65 (d, *J* = 2.0 Hz, 1.38H), 1.46 – 1.36 (m, 1H).

^{13}C NMR (201 MHz, $CDCl_3$) (inseparable 1:1.2 mixture of diastereoisomers) δ 170.5 (b), 168.7 (a), 137.2 (a), 136.3 (b), 136.3 (a), 136.1 (a), 136.0 (b), 135.0 (b), 133.9 (b), 133.8 (a), 127.3 (a), 127.2 (b), 126.9 (b), 126.6

(a), 122.8 (b), 122.6 (a), 121.9 (a), 121.8 (b), 120.2 (a), 120.1 (b), 119.8 (a+b), 118.8 (a), 118.6 (b), 118.3 (a), 118.3 (b), 113.4 (b), 111.4 (a), 111.1 (a+b), 111.0 (b), 111.0 (a), 108.6 (a), 108.4 (b), 61.8 (b), 60.2 (a), 59.2 (b), 59.0 (a), 57.9 (b), 57.6 (a), 53.9 (b), 53.7 (a), 49.6 (b), 46.8 (a), 40.5 (b), 38.5 (b), 38.4 (a), 37.8 (a), 37.2 (a), 36.8 (b), 31.6 (b), 30.4 (a), 30.0 (b), 22.5 (a), 22.0 (a), 21.9 (b), 21.8 (a), 21.6 (b).

HRMS (ESI/QTOF) m/z: $[M + H]^+$ Calcd for $C_{29}H_{31}N_4O^+$ 451.2492; Found 451.2495.

IR (ν_{max}, cm^{-1}) 3417 (w), 3286 (w), 2921 (w), 2849 (w), 1614 (m), 1452 (m), 1438 (m), 1406 (m), 1345 (w), 1319 (w), 1297 (m), 1261 (w), 1235 (w), 1145 (w), 1107 (w), 1055 (w), 1027 (w), 1009 (w), 907 (m), 801 (w), 730 (s).

3) References

(1) Zhang, W.; Bah, J.; Wohlfarth, A.; Franzén, J. A Stereodivergent Strategy for the Preparation of Corynantheine and Ipecac Alkaloids, Their Epimers, and Analogues: Efficient Total Synthesis of (−)-Dihydrocorynantheol, (−)-Corynantheol, (−)-Protoemetinol, (−)-Corynantheal, (−)-Protoemetine, and Related. *Chem. Eur. J.* **2011**, *17* (49), 13814–13824. https://doi.org/10.1002/chem.201102012.

(2) Zhao, H.; Gao, K.; Ma, H.; Yip, T. C.; Dai, W.-M. Synthesis of the C19–C30 Bis-THF Fragment of Iriomoteolide-13a via Stepwise SN2 Cyclization and Intramolecular Syn-Oxypalladation. *Org. Chem. Front.* **2021**, *8* (23), 6491–6498. https://doi.org/10.1039/D1QO01049B.

(3) Younai, A.; Zeng, B.-S.; Meltzer, H. Y.; Scheidt, K. A. Enantioselective Syntheses of Heteroyohimbine Natural Products: A Unified Approach through Cooperative Catalysis. *Angewandte Chemie International Edition* **2015**, *54* (23), 6900–6904. https://doi.org/10.1002/anie.201502011.

(4) Rose, H. A. Crystallographic Data. 92. Ajmalicine, Ajmalicine Hydrate, and Py-Tetrahydrosepentinol. *Anal. Chem.* **1955**, *27* (3), 469–470. https://doi.org/10.1021/ac60099a050.

(5) Stahl, R.; Borschberg, H.-J. A Reinvestigation of the Oxidative Rearrangement of Yohimbane-Type Alkaloids. Part A. Formation of Pseudoindoxyl (= 1,2-Dihydro-3H-Indol-3-One) Derivatives. *Helvetica Chimica Acta* **1994**, *77* (5), 1331–1345. https://doi.org/10.1002/hlca.19940770514.

(6) Lounasmaa, M.; Kan, S.-K. A 400 Mhz 1H NMR Study of the Eight Basic Heteroyohimbine Alkaloids. *Tetrahedron* **1980**, *36* (11), 1607–1611. https://doi.org/10.1016/S0040-4020(01)83129-X.

(7) Dubost, J.-P.; Léger, J.-M.; Goursolle, M.; Colleter, J.-C.; Carpy, A. Structure de la Raubasine Hydratée. *Acta Crystallogr., Sect. C: Cryst. Struct. Commun.* **1984**, *C40*, 152–154. https://doi.org/10.1107/S0108270184003383.

(8) Hanessian, S.; Faucher, A. M. General Stereocontrolled Strategy for the Heteroyohimbine Alkaloids: The Total Synthesis of (-)-Ajmalicine and (+)-19-Epiajmalicine. *J. Org. Chem.* **1991**, *56* (9), 2947–2949. https://doi.org/10.1021/jo00009a001.

(9) Wang, X.; Xia, D.; Qin, W.; Zhou, R.; Zhou, X.; Zhou, Q.; Liu, W.; Dai, X.; Wang, H.; Wang, S.; Tan, L.; Zhang, D.; Song, H.; Liu, X.-Y.; Qin, Y. A Radical Cascade Enabling Collective Syntheses of Natural Products. *Chem* **2017**, *2* (6), 803–816. https://doi.org/10.1016/j.chempr.2017.04.007.

(10) Uusvuori, R.; Lounasmaa, M. 13-C NMR Data of 3-Isoajmalicine and 19-Epiajmalicine. *Planta Med* **1981**, *41* (04), 406–407. https://doi.org/10.1055/s-2007-971737.

(11) Liu, C.; Sun, Z.; Xie, F.; Liang, G.; Yang, L.; Li, Y.; Cheng, M.; Lin, B.; Liu, Y. Gold(I)-Catalyzed Pathway-Switchable Tandem Cycloisomerizations to Indolizino[8,7-b]Indole and Indolo[2,3-a]Quinolizine Derivatives. *Chem. Commun.* **2019**, *55* (96), 14418–14421. https://doi.org/10.1039/C9CC05667J.

(12) Jacquemard, U.; Bénéteau, V.; Lefoix, M.; Routier, S.; Mérour, J.-Y.; Coudert, G. Mild and Selective Deprotection of Carbamates with Bu4NF. *Tetrahedron* **2004**, *60* (44), 10039–10047. https://doi.org/10.1016/j.tet.2004.07.071.

(13) Frigerio, M.; Santagostino, M.; Sputore, S. A User-Friendly Entry to 2-Iodoxybenzoic Acid (IBX). *J. Org. Chem.* **1999**, *64* (12), 4537–4538. https://doi.org/10.1021/jo9824596.

(14) Merlini, L.; Mondelli, R.; Nasini, G.; Hesse, M. The Structure of Roxburghines A–E, New Indole Alkaloids from an *Uncaria* Sp. *Tetrahedron* **1970**, *26* (10), 2259–2279. https://doi.org/10.1016/S0040-4020(01)92806-6.

(15) Merlini, L.; Mondelli, R.; Nasini, G.; Wehrli, F. W.; Hagaman, E. W.; Wenkert, E. 13C-NMR. Analysis of the Roxburghines. *Helvetica Chimica Acta* **1976**, *59* (6), 2254–2260. https://doi.org/10.1002/hlca.19760590641.

4) Copies of NMR spectra

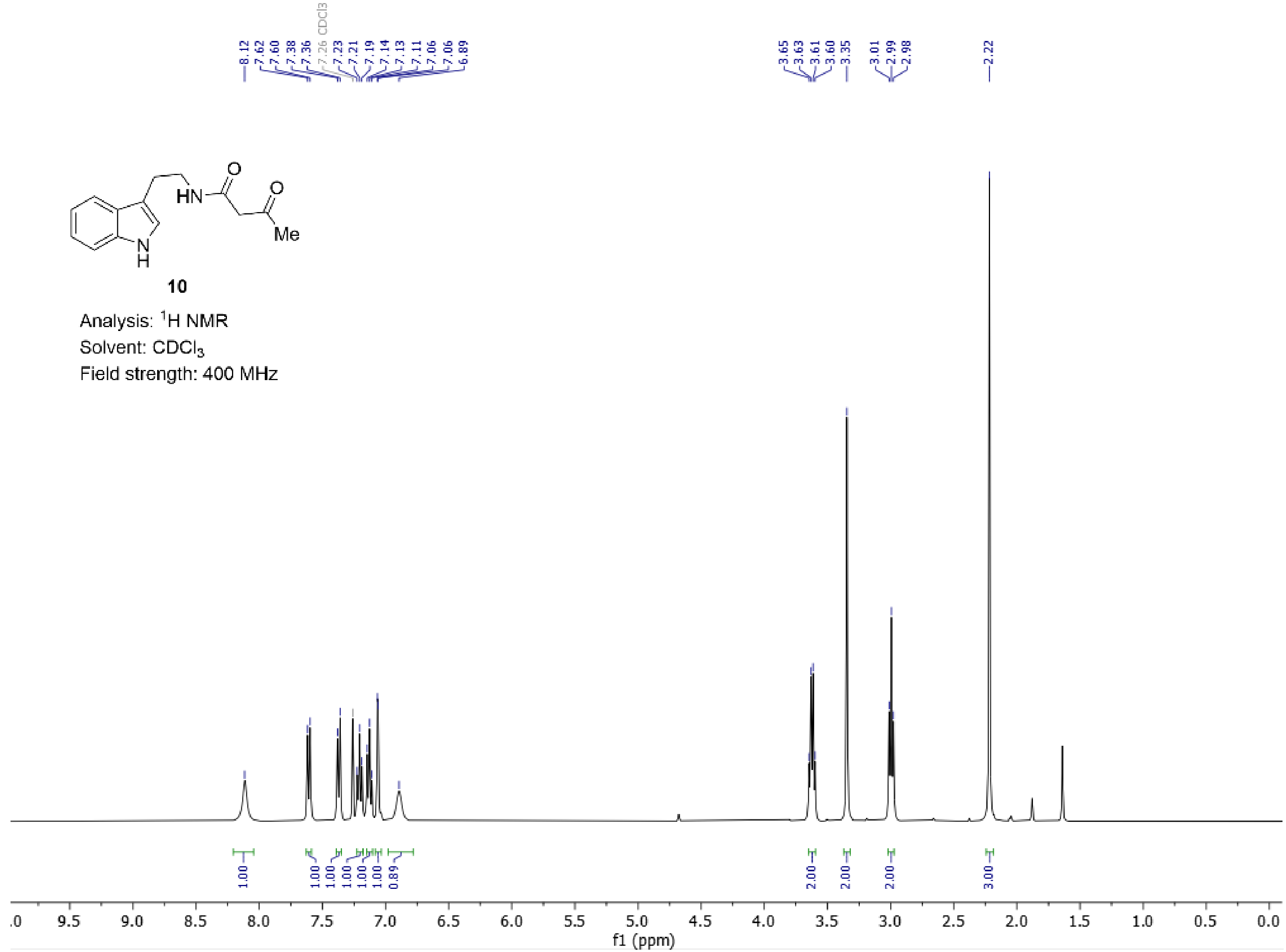

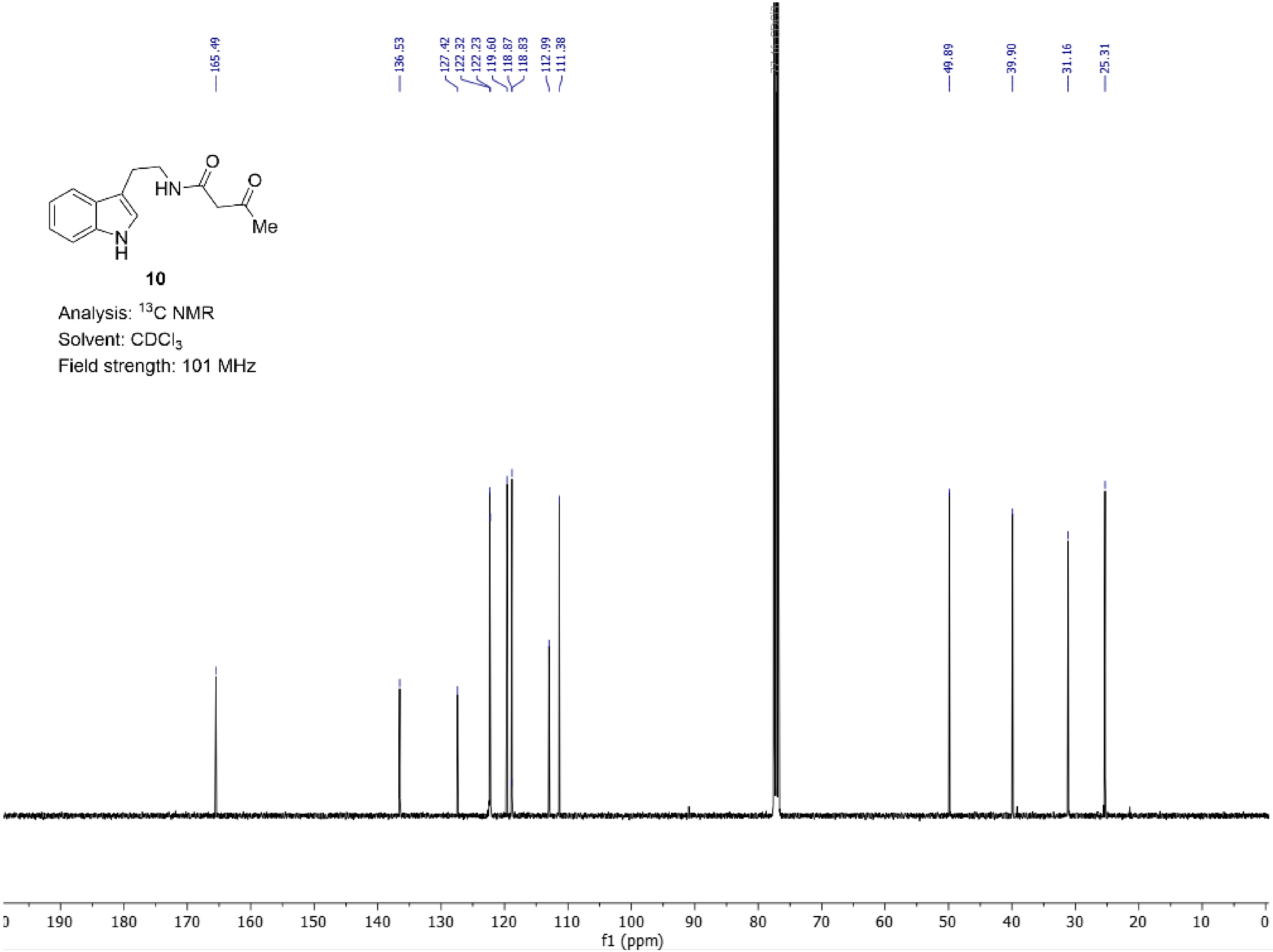

10

Analysis: ^{13}C NMR

Solvent: $CDCl_3$

Field strength: 101 MHz

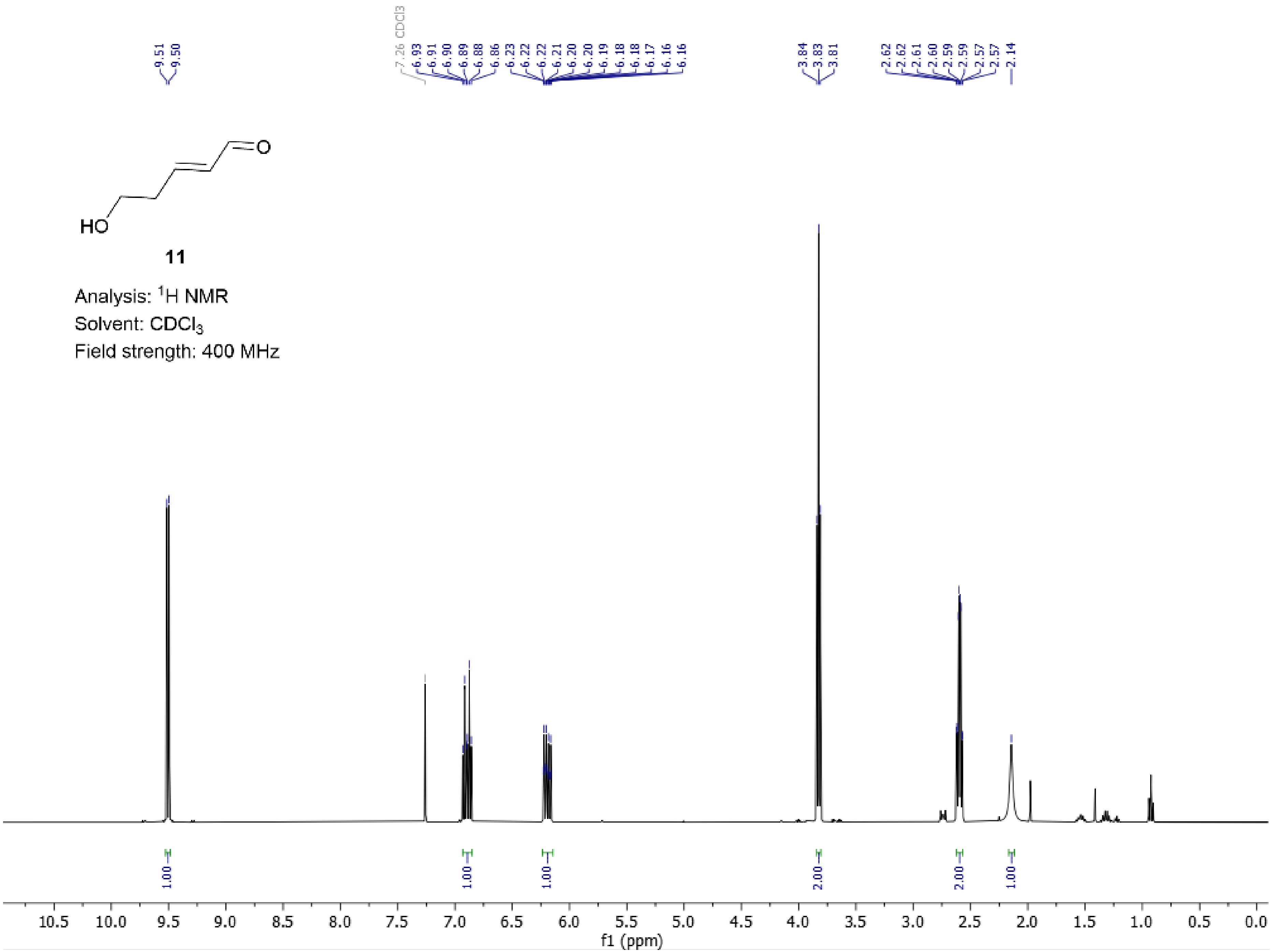

9.51
9.50
7.26 CDCl3
6.93
6.91
6.90
6.89
6.88
6.86
6.23
6.22
6.22
6.21
6.20
6.20
6.19
6.18
6.18
6.17
6.16
6.16
3.84
3.83
3.81
2.62
2.62
2.61
2.60
2.59
2.59
2.57
2.57
2.14
HO
O
11
Analysis: ^{1}H NMR
Solvent: $CDCl_3$
Field strength: 400 MHz
1.00
1.00
1.00
2.00
2.00
1.00
10.5
10.0
9.5
9.0
8.5
8.0
7.5
7.0
6.5
6.0
5.5
5.0
4.5
4.0
3.5
3.0
2.5
2.0
1.5
1.0
0.5
0.0
f1 (ppm)

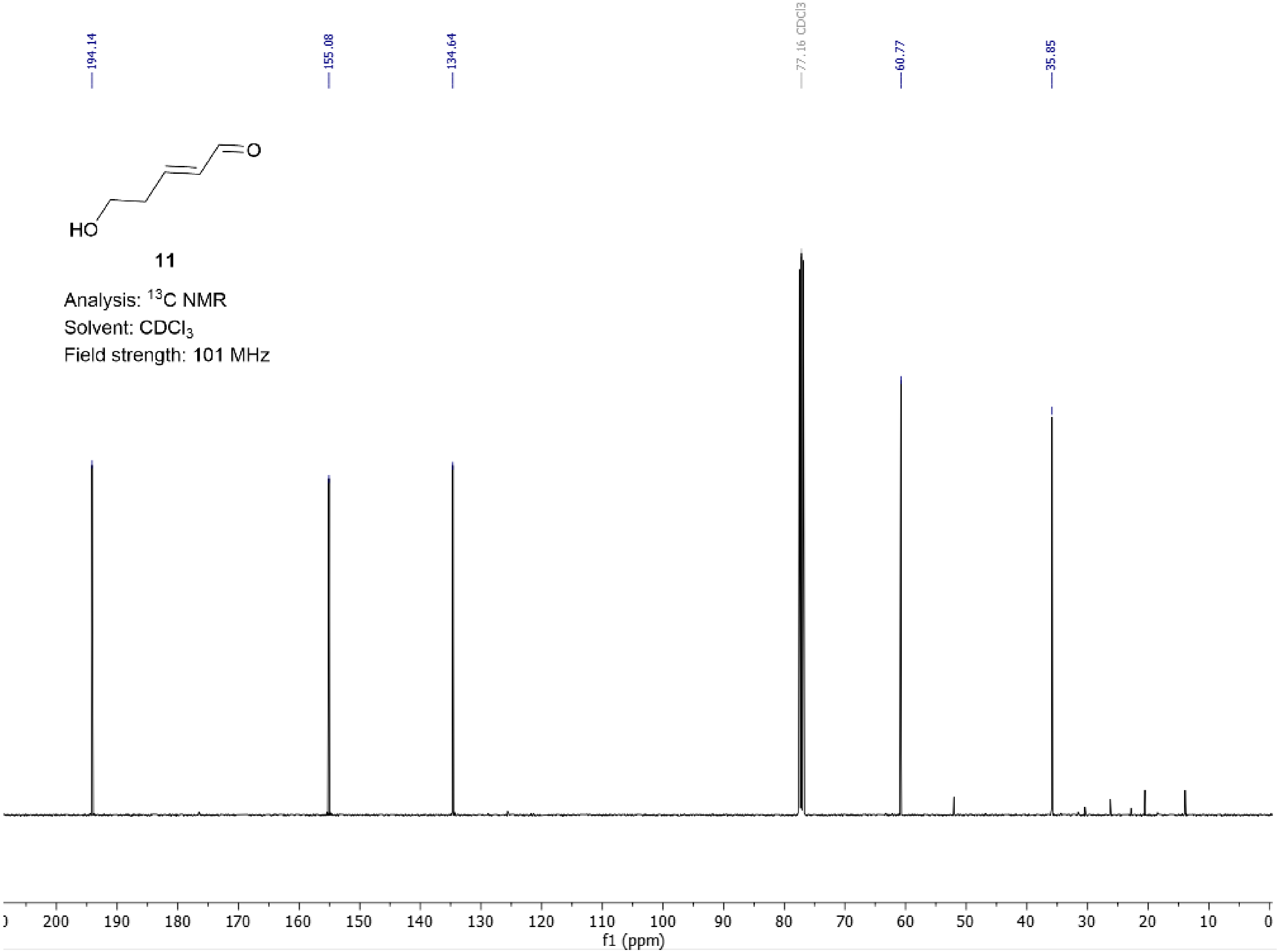

194.14
155.08
134.64
77.16 CDCl3
60.77
35.85
HO
O
11
Analysis: 13C NMR
Solvent: CDCl3
Field strength: 101 MHz
200
190
180
170
160
150
140
130
120
110
100
90
80
70
60
50
40
30
20
10
0
f1 (ppm)

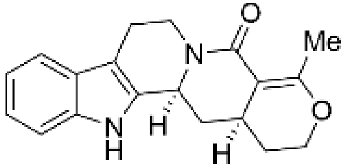

9

Analysis: ^{1}H NMR
Solvent: $CDCl_3$
Field strength: 600 MHz

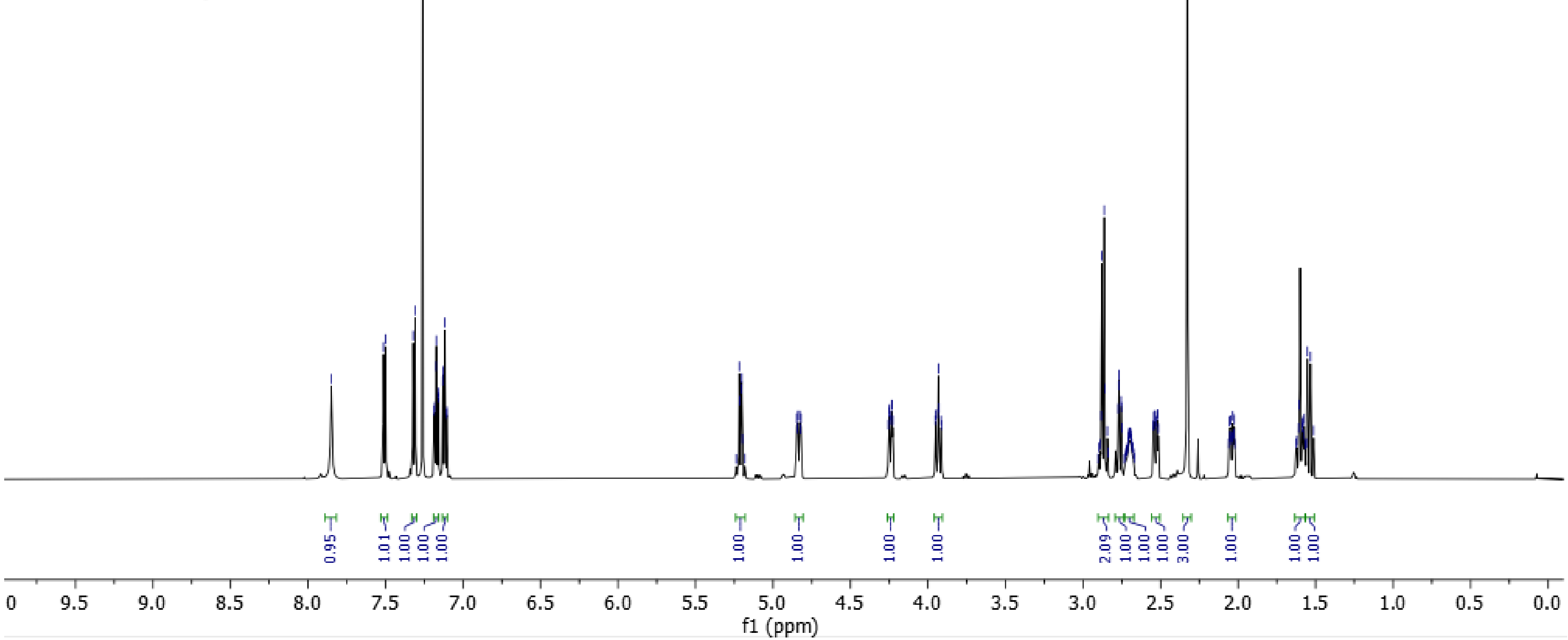

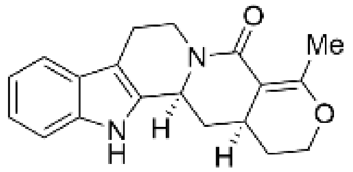

9

Analysis: ^{13}C NMR
Solvent: $CDCl_3$
Field strength: 151 MHz

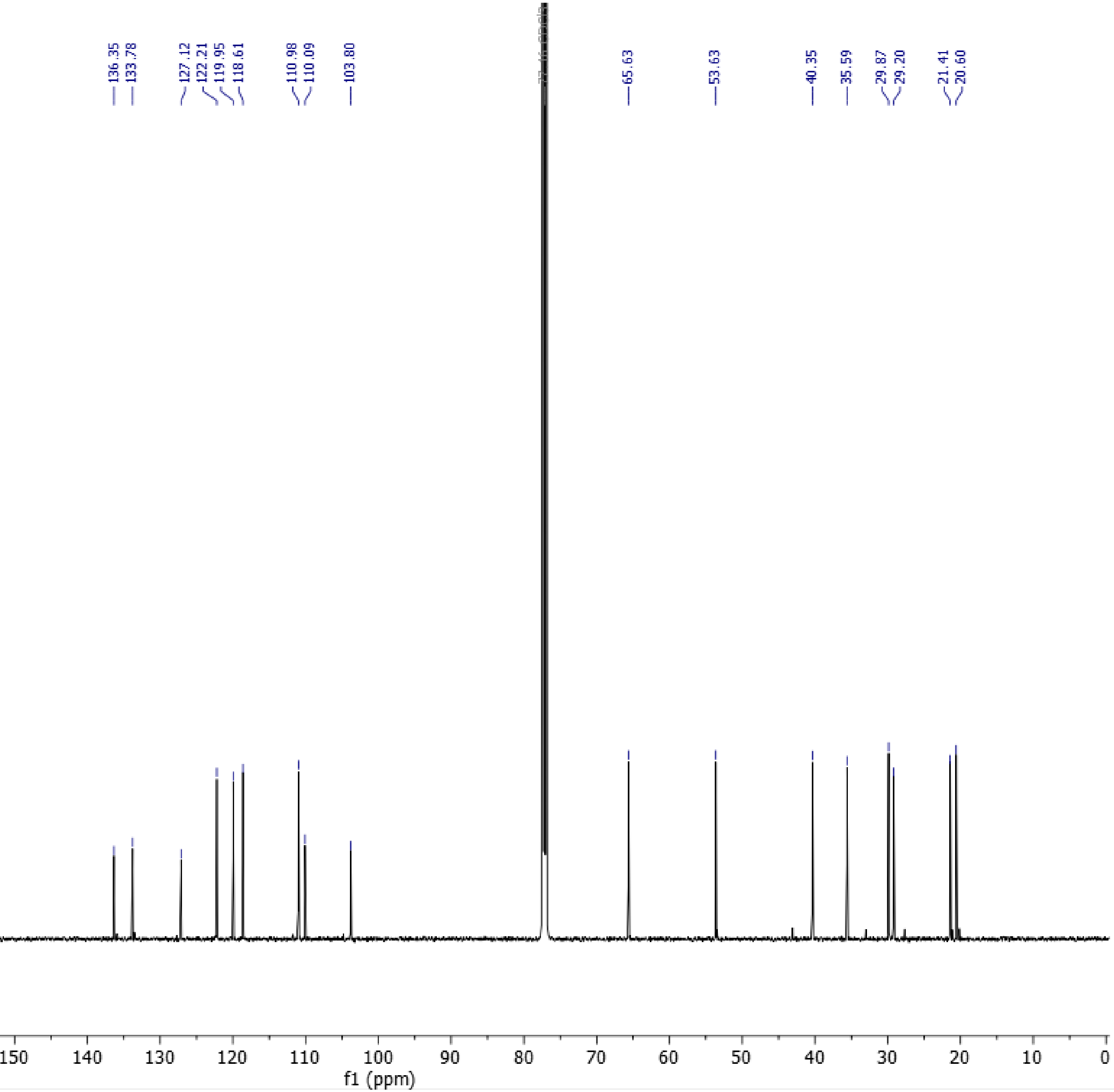

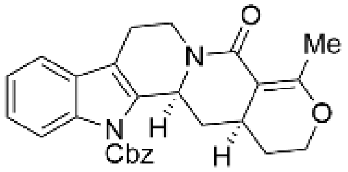

17

Analysis: ^{1}H NMR
Solvent: $CDCl_3$
Field strength: 600 MHz

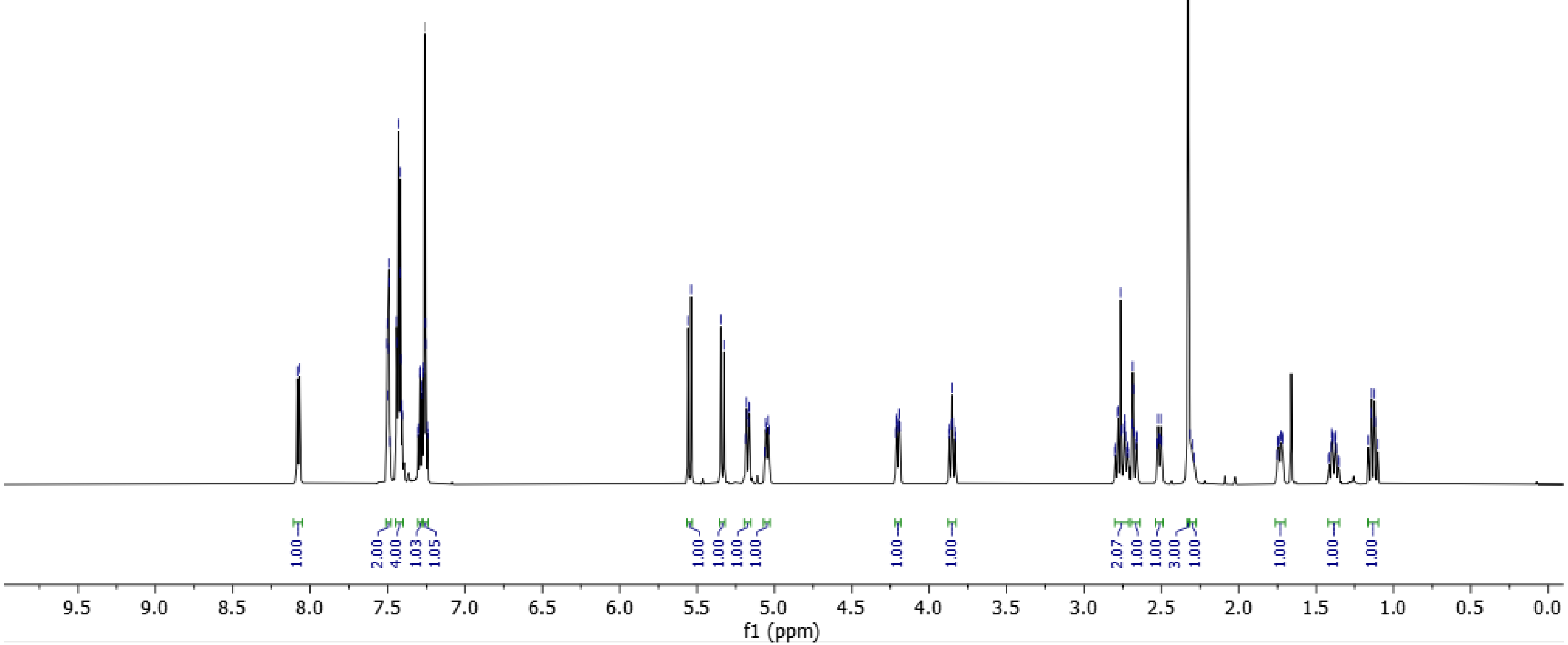

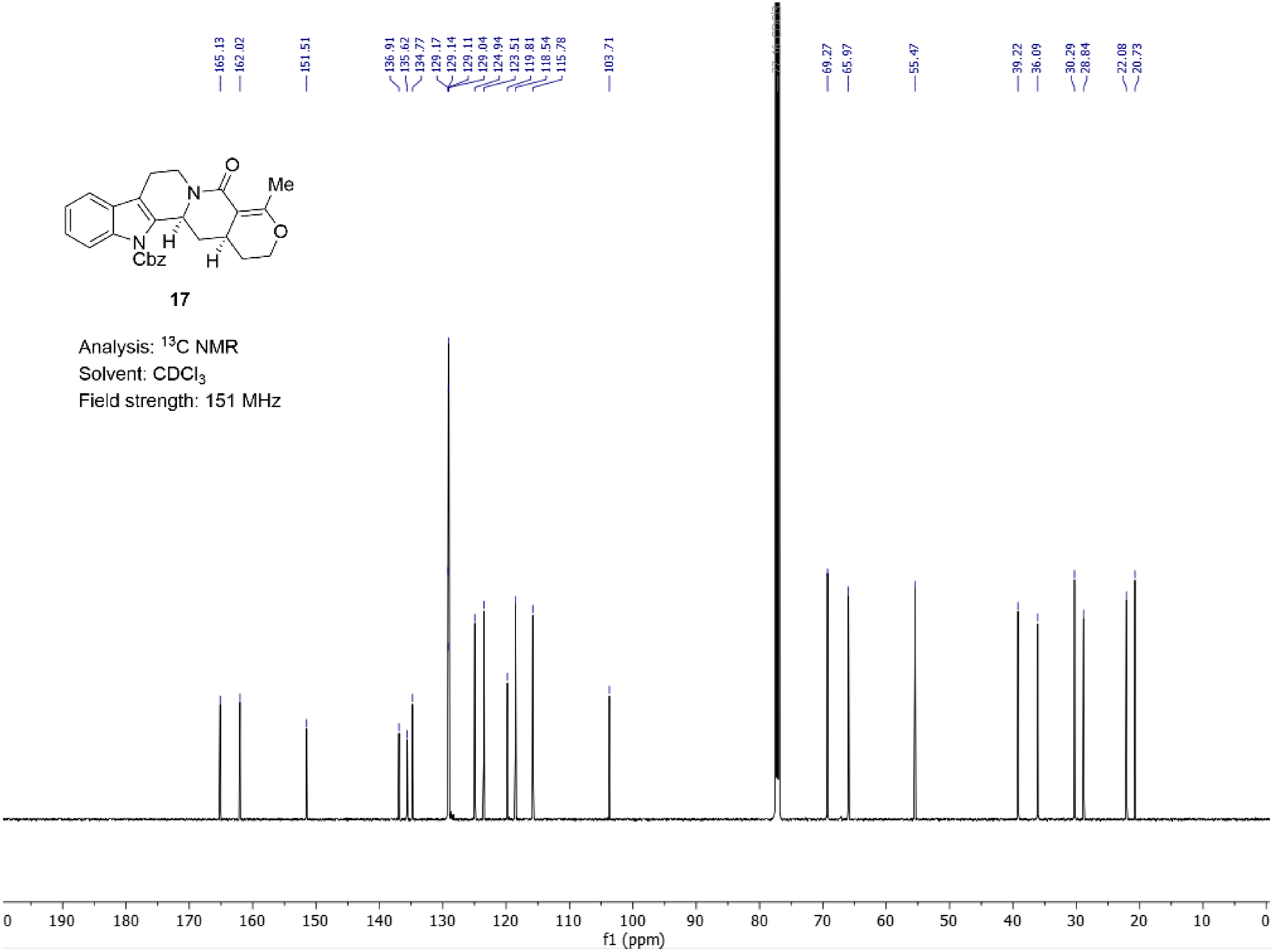

165.13
162.02
151.51
136.91
135.62
134.77
129.17
129.14
129.11
129.04
124.94
123.51
119.81
118.54
115.78
103.71
69.27
65.97
55.47
39.22
36.09
30.29
28.84
22.08
20.73
Me
O
N
H
H
Cbz
17
Analysis: ^{13}C NMR
Solvent: $CDCl_3$
Field strength: 151 MHz
f1 (ppm)

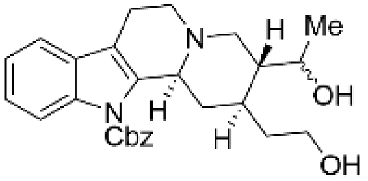

19a:19b, dr 3:1

Analysis: ^{1}H NMR

Solvent: $CDCl_3$

Field strength: 800 MHz

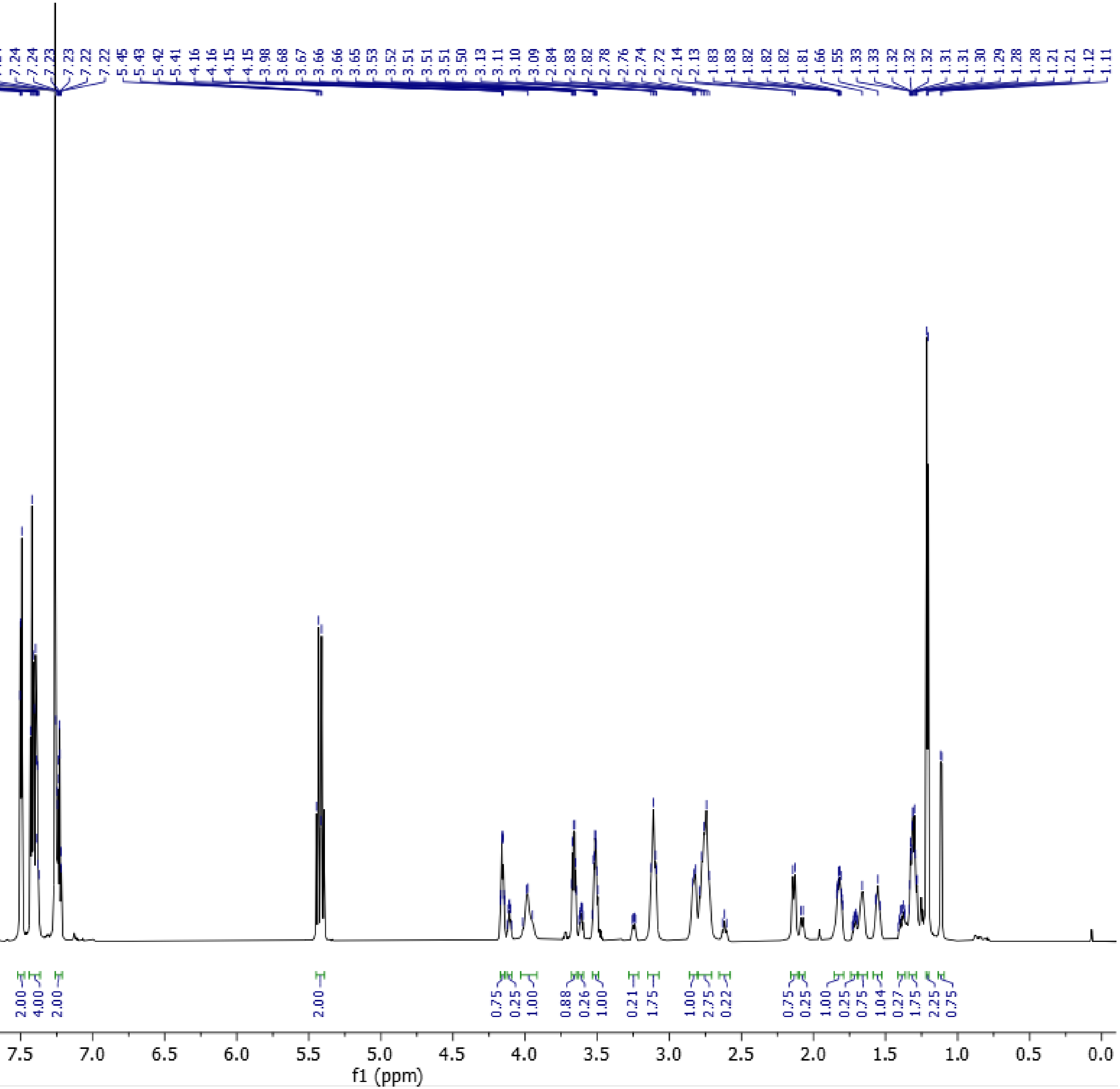

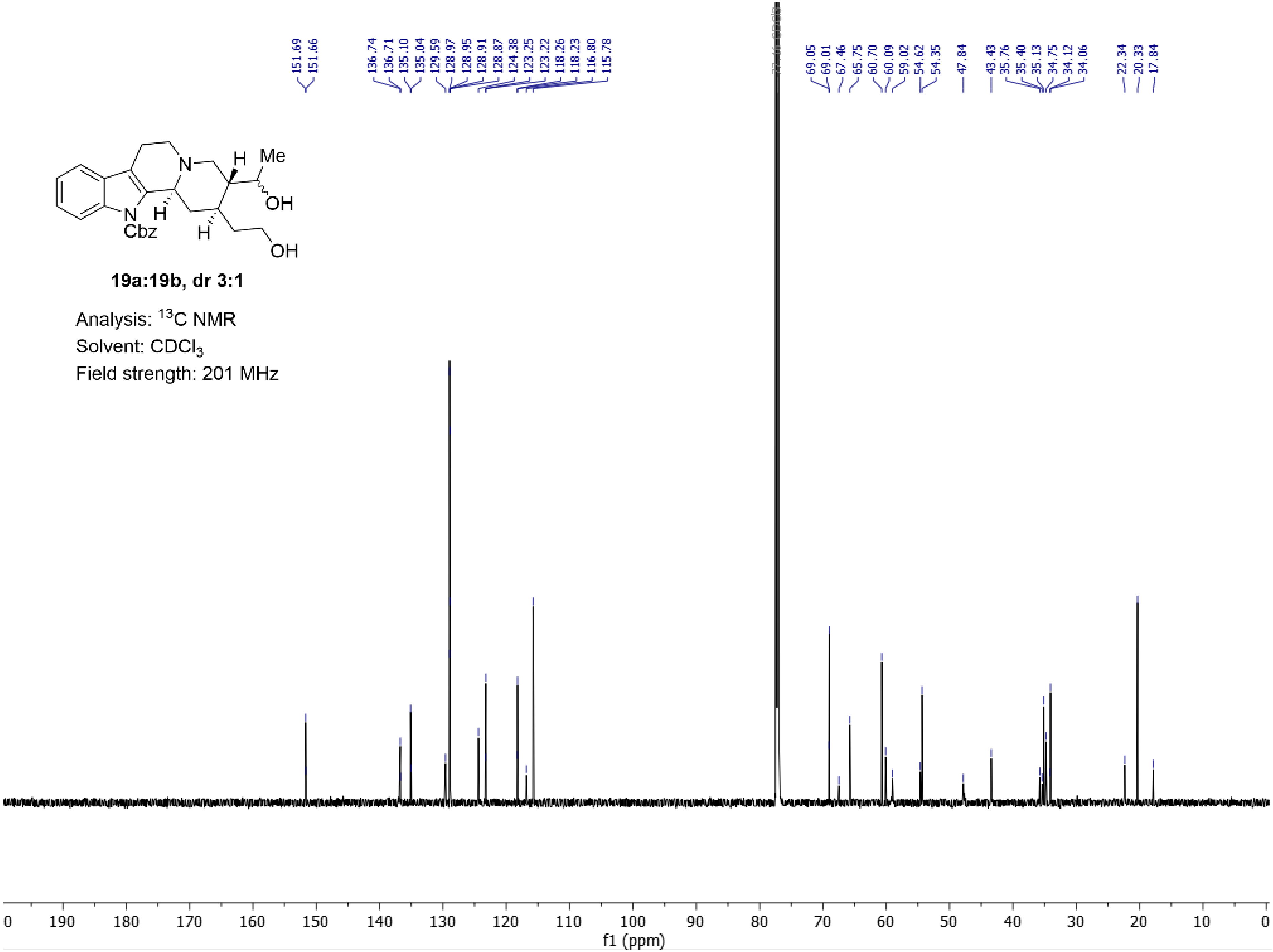
19a:19b, dr 3:1
Analysis: 13C NMR
Solvent: CDCl3
Field strength: 201 MHz
151.69
151.66
136.74
136.71
135.10
135.04
129.59
128.97
128.95
128.91
128.87
124.38
123.25
123.22
118.26
118.23
116.80
115.78
69.05
69.01
67.46
65.75
60.70
60.09
59.02
54.62
54.35
47.84
43.43
35.76
35.40
35.13
34.75
34.12
34.06
22.34
20.33
17.84
f1 (ppm)

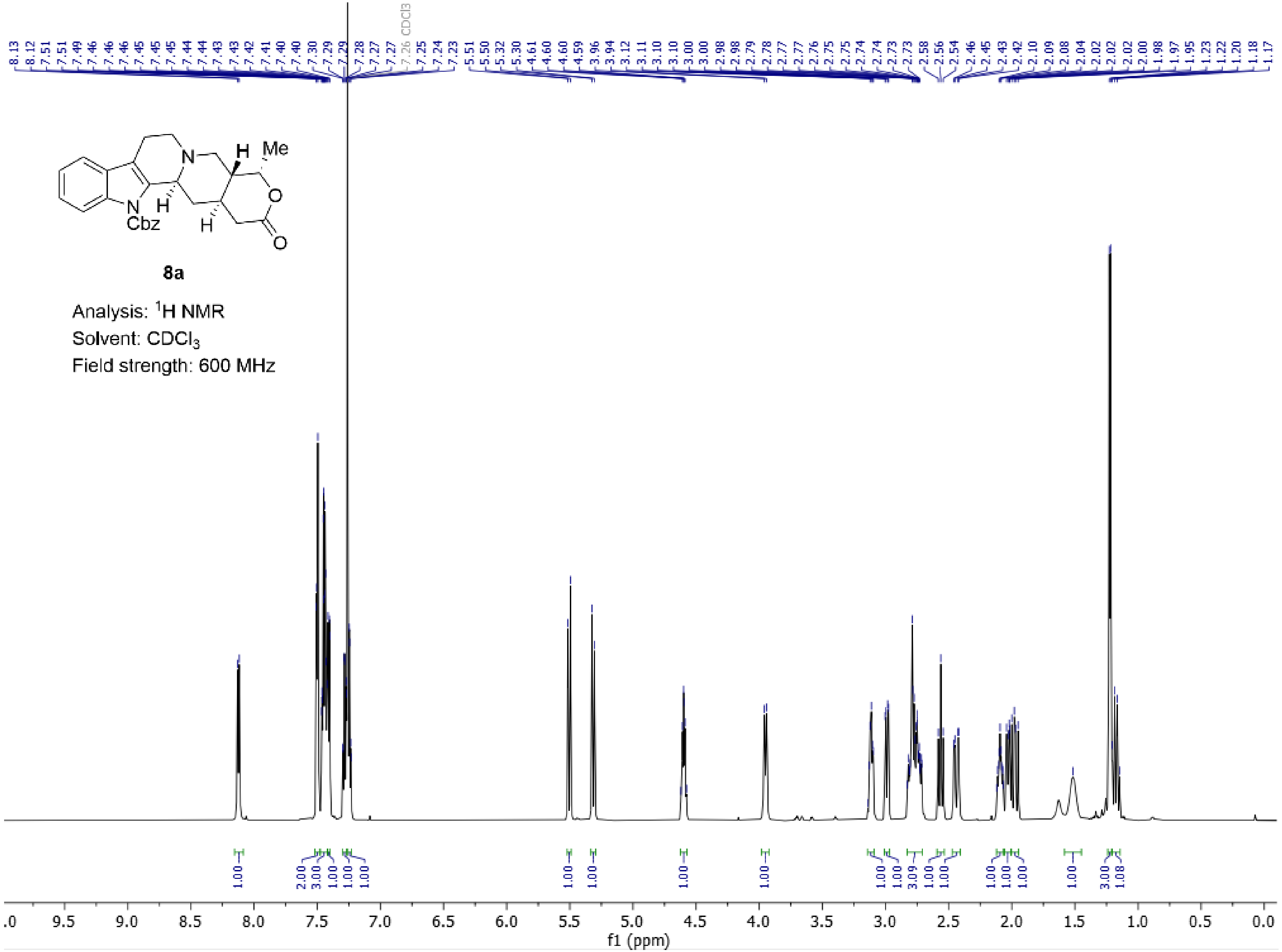

8.13 8.12 7.51 7.51 7.49 7.46 7.46 7.46 7.45 7.45 7.45 7.44 7.44 7.43 7.43 7.42 7.41 7.40 7.40 7.30 7.29 7.29 7.28 7.27 7.27 7.26 CDCl3 7.25 7.24 7.23 5.51 5.50 5.32 5.30 4.61 4.60 4.59 3.96 3.94 3.12 3.11 3.10 3.10 3.00 3.00 2.98 2.98 2.79 2.78 2.77 2.77 2.76 2.75 2.75 2.74 2.74 2.73 2.73 2.58 2.56 2.54 2.46 2.45 2.43 2.42 2.10 2.09 2.08 2.04 2.02 2.02 2.02 2.00 1.98 1.97 1.95 1.23 1.22 1.20 1.18 1.17
Me
N
O
H
Cbz
8a
Analysis: ^{1}H NMR
Solvent: $CDCl_3$
Field strength: 600 MHz
1.00 2.00 3.00 1.00 1.00 1.00 1.00 1.00 1.00 1.00 1.00 1.00 3.09 1.00 1.00 1.00 1.00 1.00 1.00 3.00 1.08
f1 (ppm)

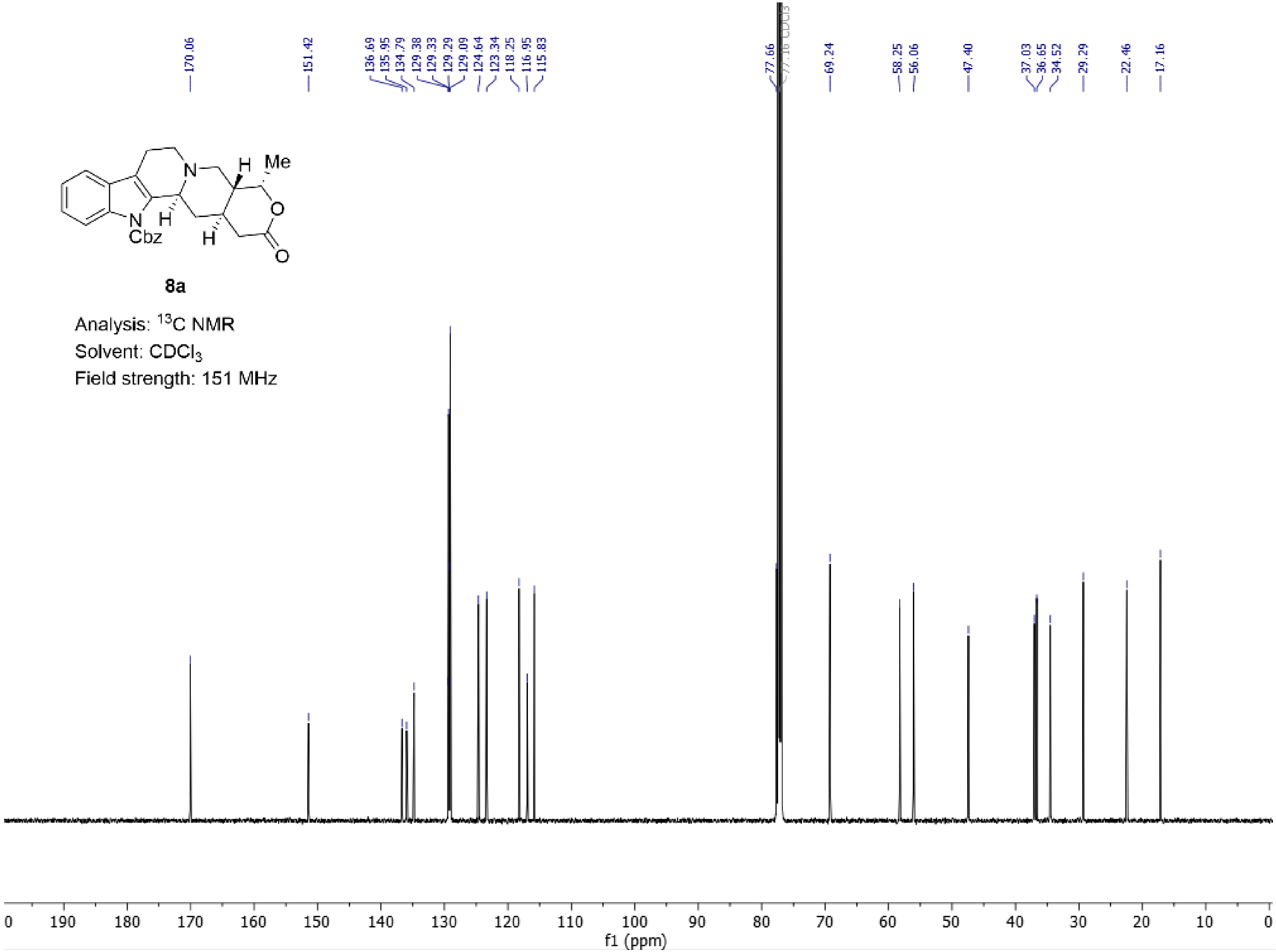

8a

Analysis: ^{13}C NMR
Solvent: $CDCl_3$
Field strength: 151 MHz

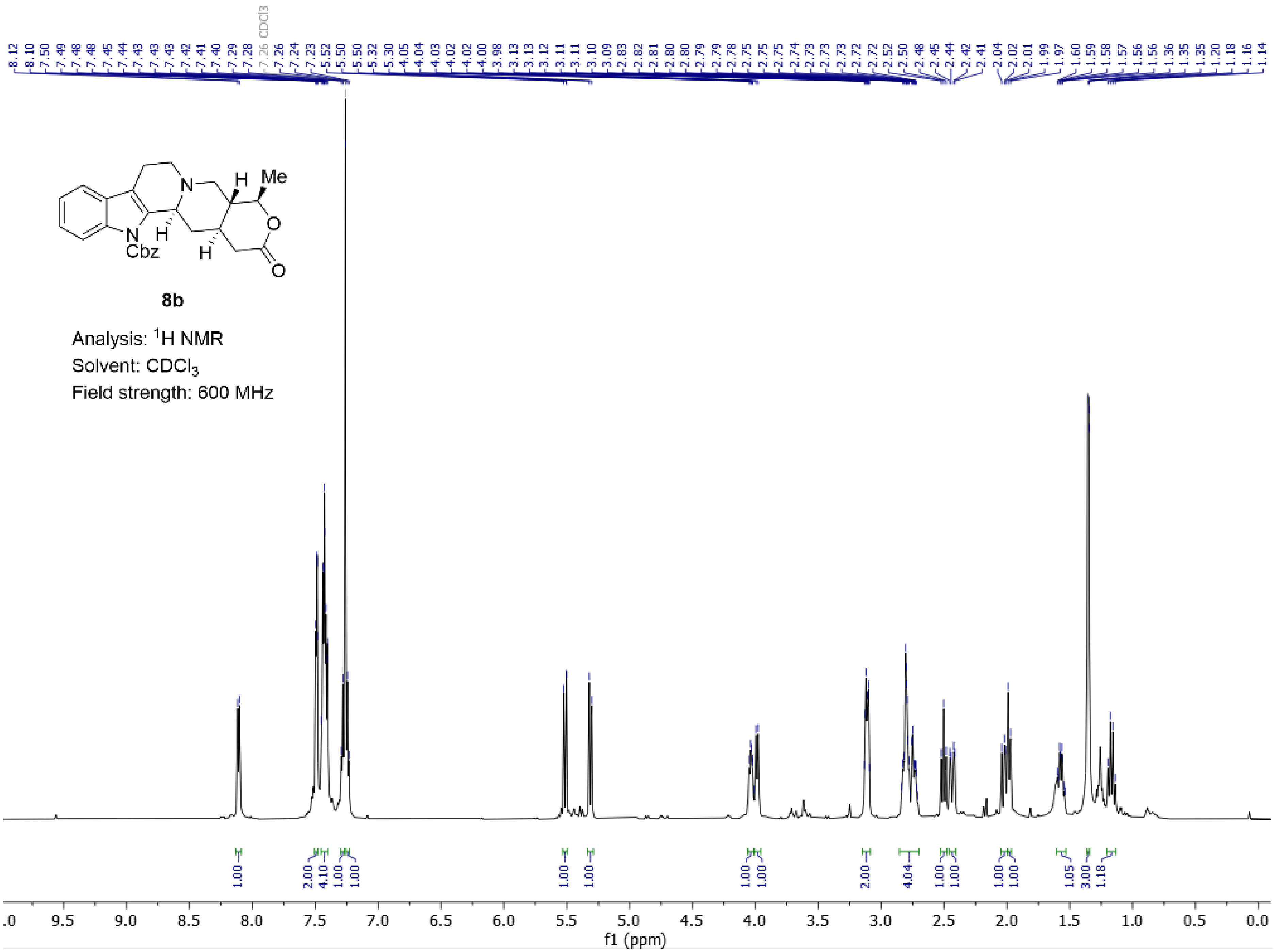
8.12 8.10 7.50 7.49 7.48 7.48 7.45 7.44 7.43 7.43 7.43 7.42 7.41 7.40 7.29 7.28 7.26 CDCl3 7.26 7.24 7.23 5.52 5.50 5.50 5.32 5.30 4.05 4.04 4.03 4.02 4.02 4.00 3.98 3.13 3.13 3.12 3.11 3.11 3.10 3.09 2.83 2.82 2.81 2.80 2.80 2.79 2.79 2.78 2.75 2.75 2.75 2.74 2.73 2.73 2.73 2.72 2.72 2.52 2.50 2.48 2.45 2.44 2.44 2.42 2.41 2.04 2.02 2.01 1.99 1.97 1.60 1.59 1.58 1.57 1.56 1.56 1.36 1.35 1.35 1.20 1.18 1.16 1.14
N
H
Me
O
N
H
Cbz
H
O
8b
Analysis: 1H NMR
Solvent: CDCl3
Field strength: 600 MHz
1.00 2.00 4.10 1.00 1.00 1.00 1.00 1.00 1.00 2.00 4.04 1.00 1.00 1.00 1.00 1.05 3.00 1.18
.0 9.5 9.0 8.5 8.0 7.5 7.0 6.5 6.0 5.5 5.0 4.5 4.0 3.5 3.0 2.5 2.0 1.5 1.0 0.5 0.0
f1 (ppm)

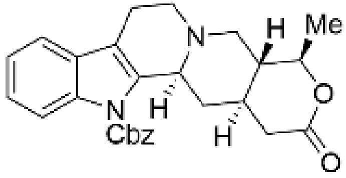

8b

Analysis: ^{13}C NMR
Solvent: $CDCl_3$
Field strength: 151 MHz

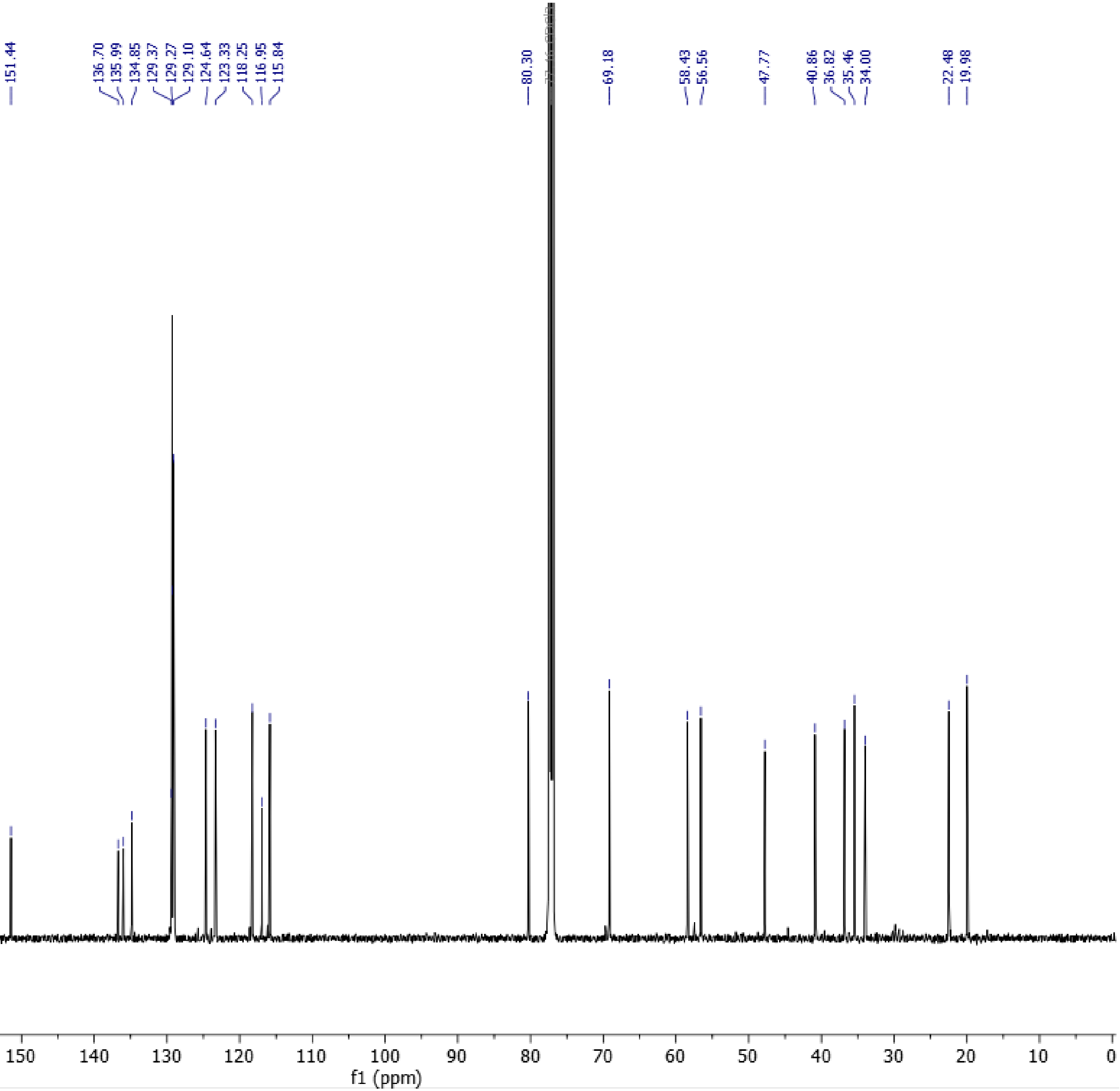

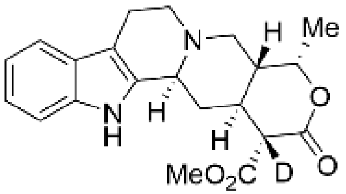

20a

Analysis: ^{1}H NMR
Solvent: Methanol-d_4
Field strength: 600 MHz

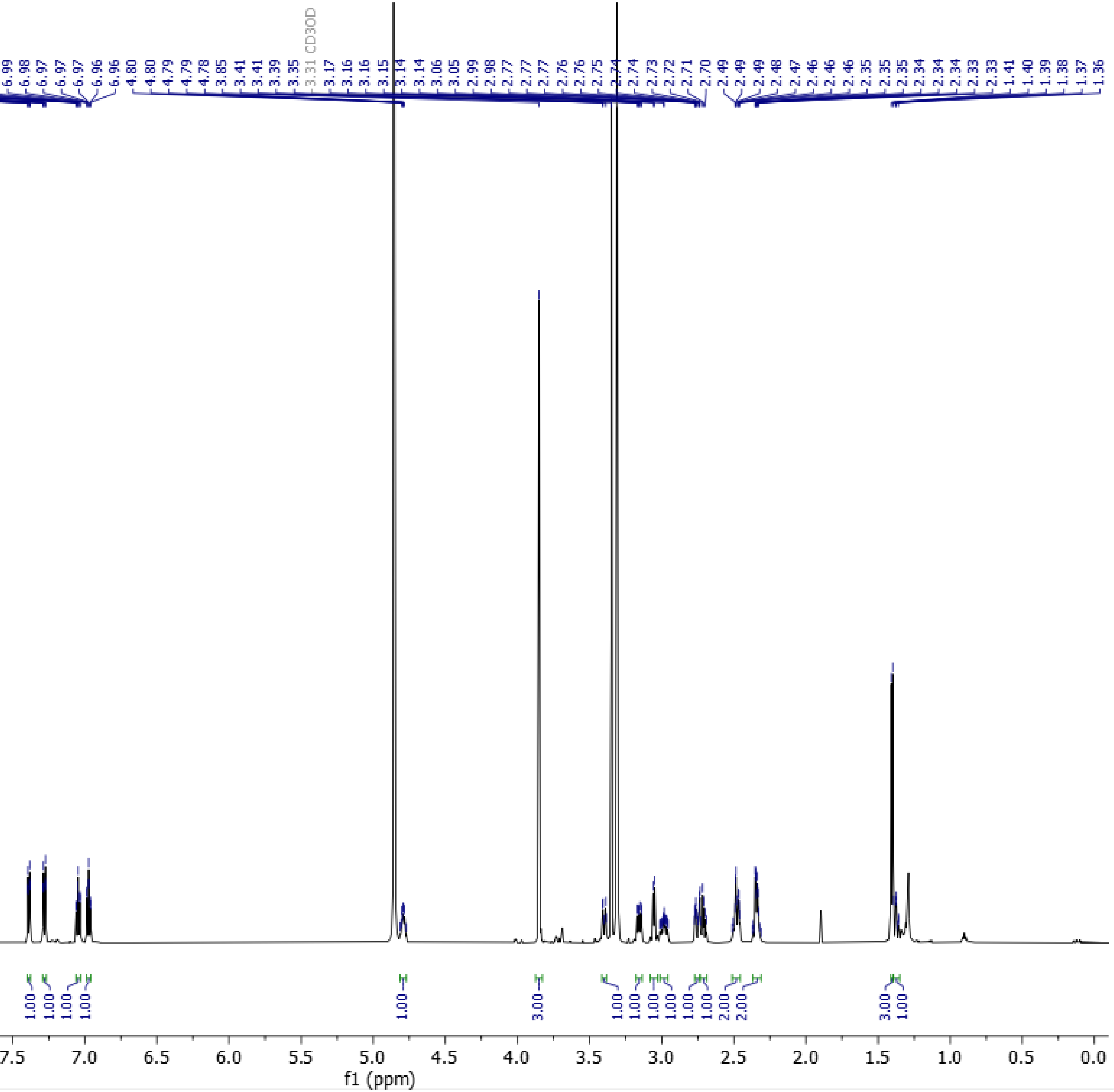

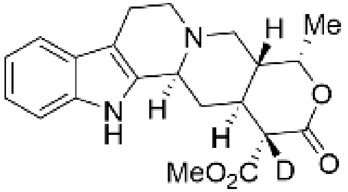

20a

Analysis: ^{13}C NMR
Solvent: Methanol-d_4
Field strength: 151 MHz

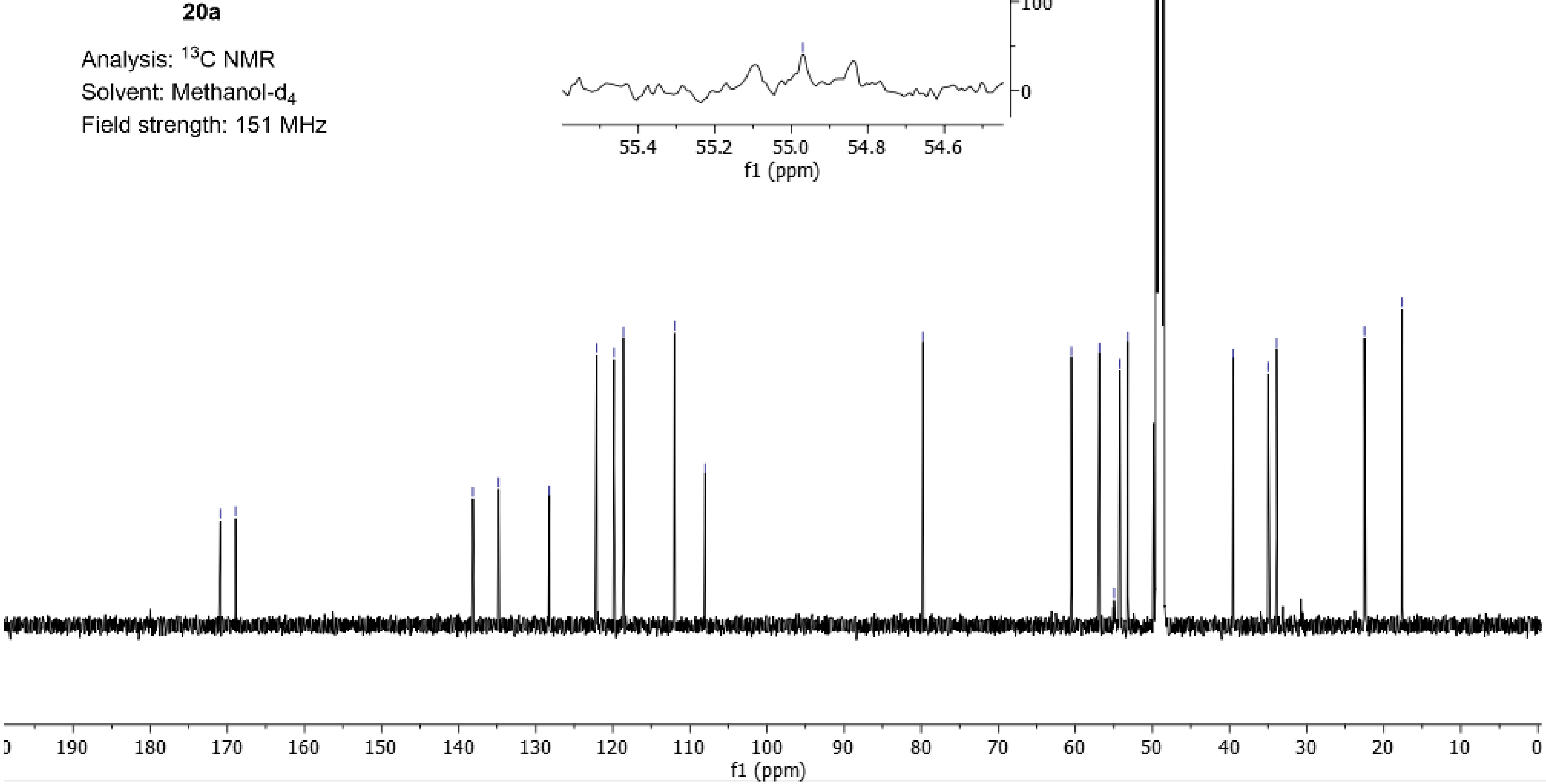

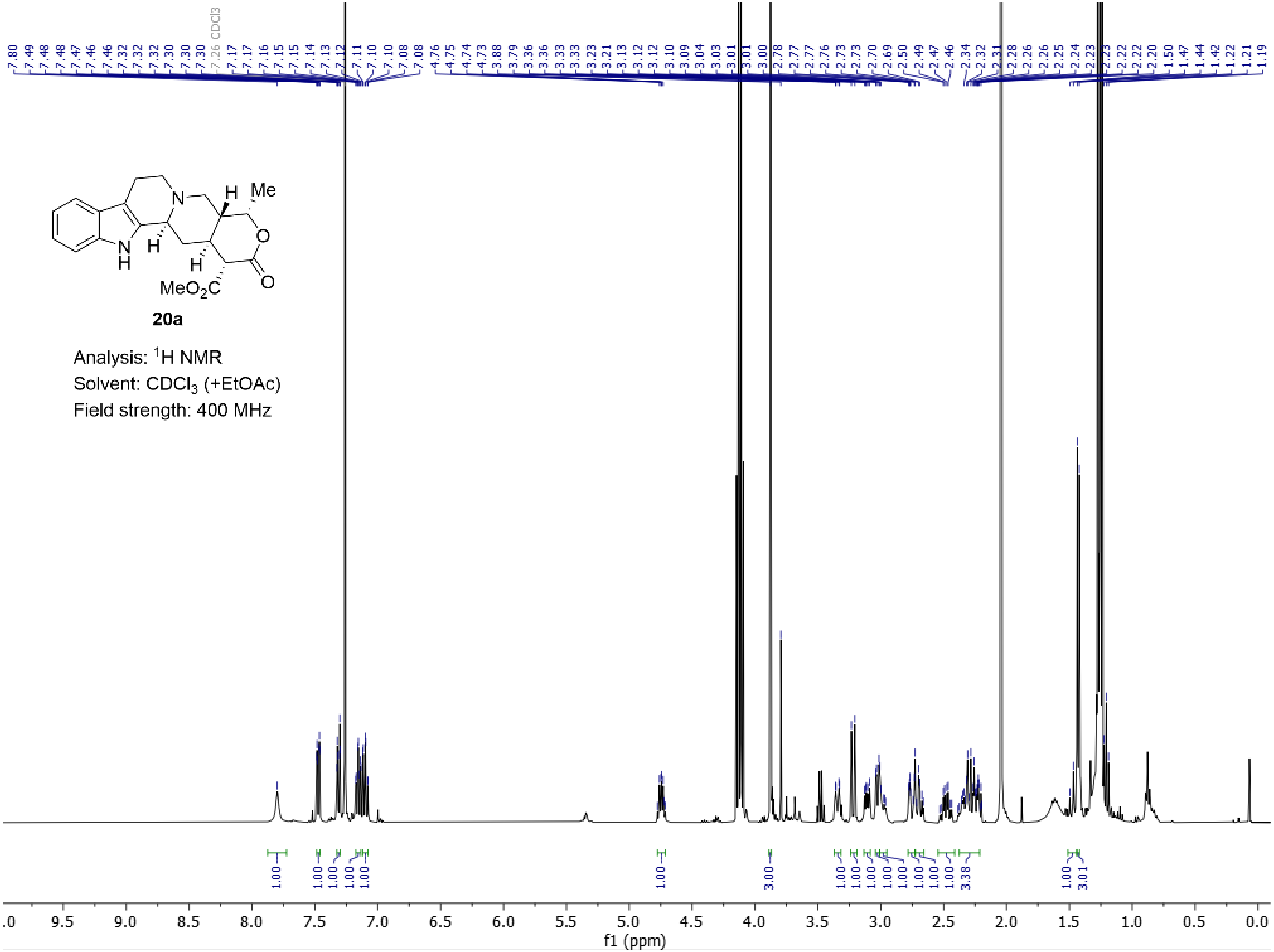

H
Me
N
O
NH
H
H
O
MeO2C
20a
Analysis: 1H NMR
Solvent: CDCl3 (+EtOAc)
Field strength: 400 MHz
f1 (ppm)

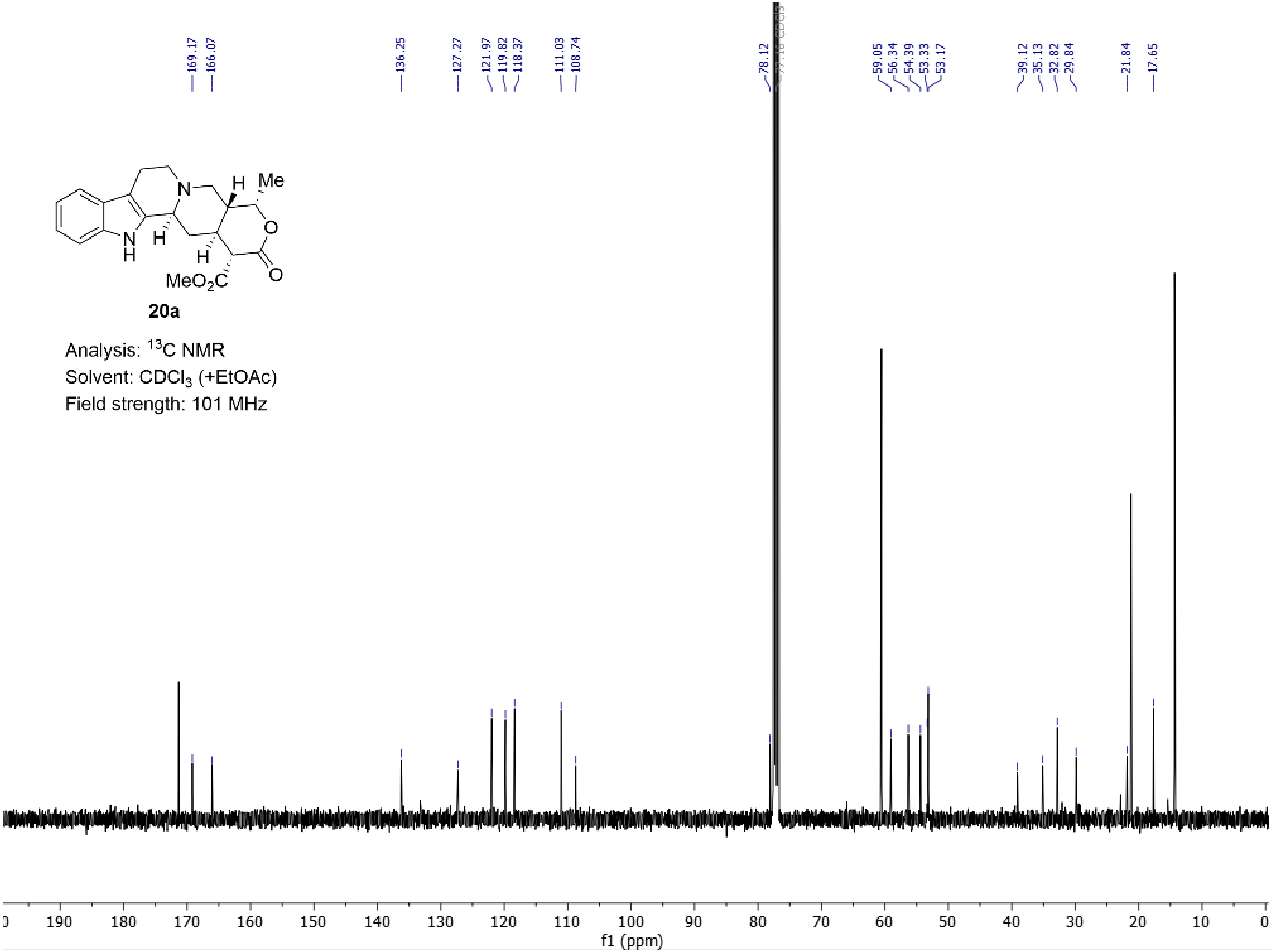

20a

Analysis: ^{13}C NMR

Solvent: $CDCl_3$ (+EtOAc)

Field strength: 101 MHz

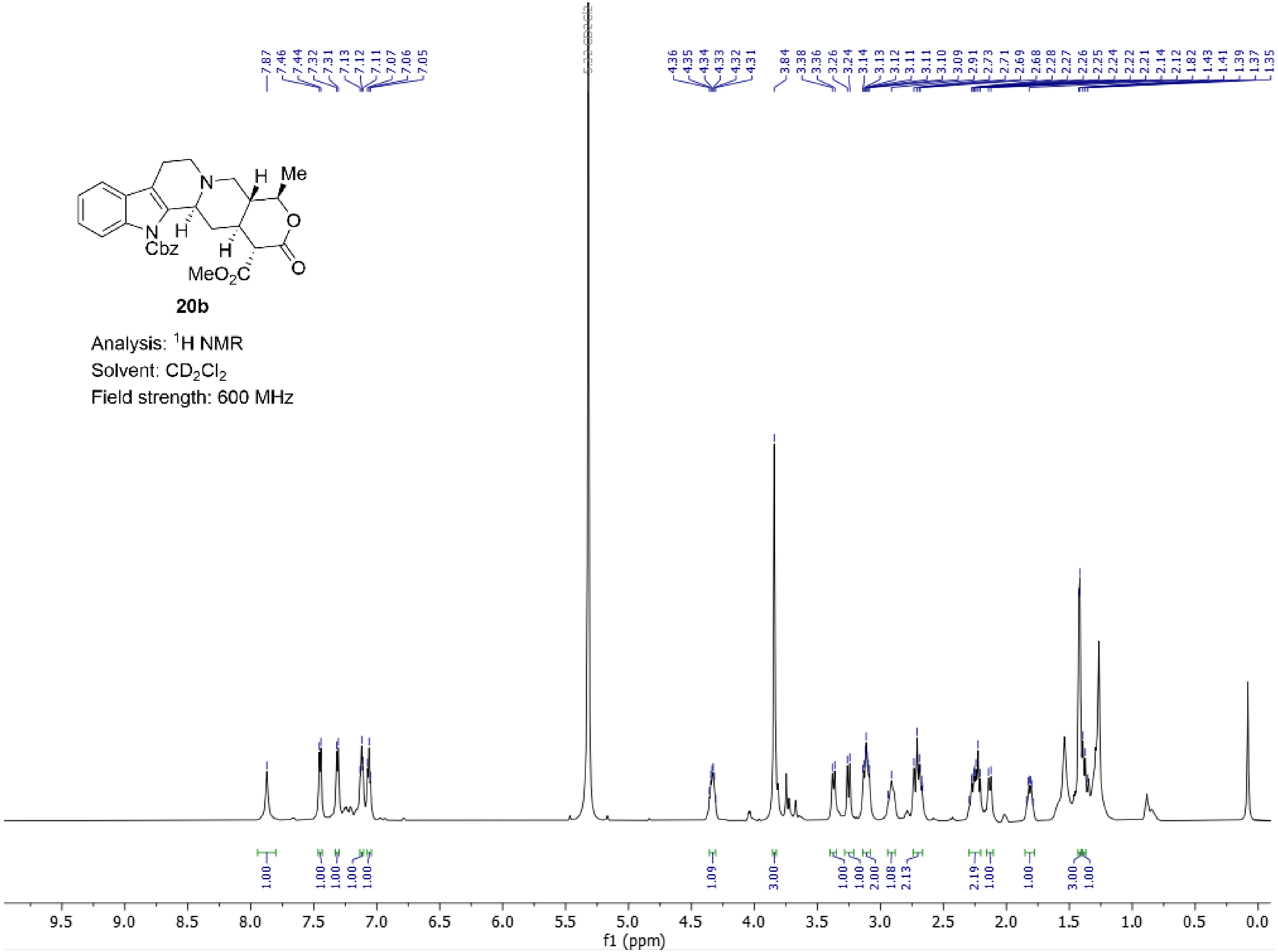
7.87
7.46
7.44
7.32
7.31
7.13
7.12
7.11
7.07
7.06
7.05
4.36
4.35
4.34
4.33
4.32
4.31
3.84
3.38
3.36
3.26
3.24
3.14
3.13
3.12
3.11
3.11
3.10
3.09
2.91
2.73
2.71
2.69
2.68
2.28
2.27
2.26
2.25
2.24
2.22
2.21
2.14
2.12
1.82
1.43
1.41
1.39
1.37
1.35
H
Me
N
O
N
Cbz
H
H
MeO2C
O
20b
Analysis: 1H NMR
Solvent: CD2Cl2
Field strength: 600 MHz
1.00
1.00
1.00
1.00
1.00
1.09
3.00
1.00
1.00
2.00
1.08
2.13
2.19
1.00
1.00
3.00
1.00
9.5
9.0
8.5
8.0
7.5
7.0
6.5
6.0
5.5
5.0
4.5
4.0
3.5
3.0
2.5
2.0
1.5
1.0
0.5
0.0
f1 (ppm)

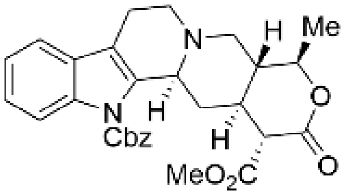

20b

Analysis: ^{13}C NMR
Solvent: CD_2Cl_2
Field strength: 151 MHz

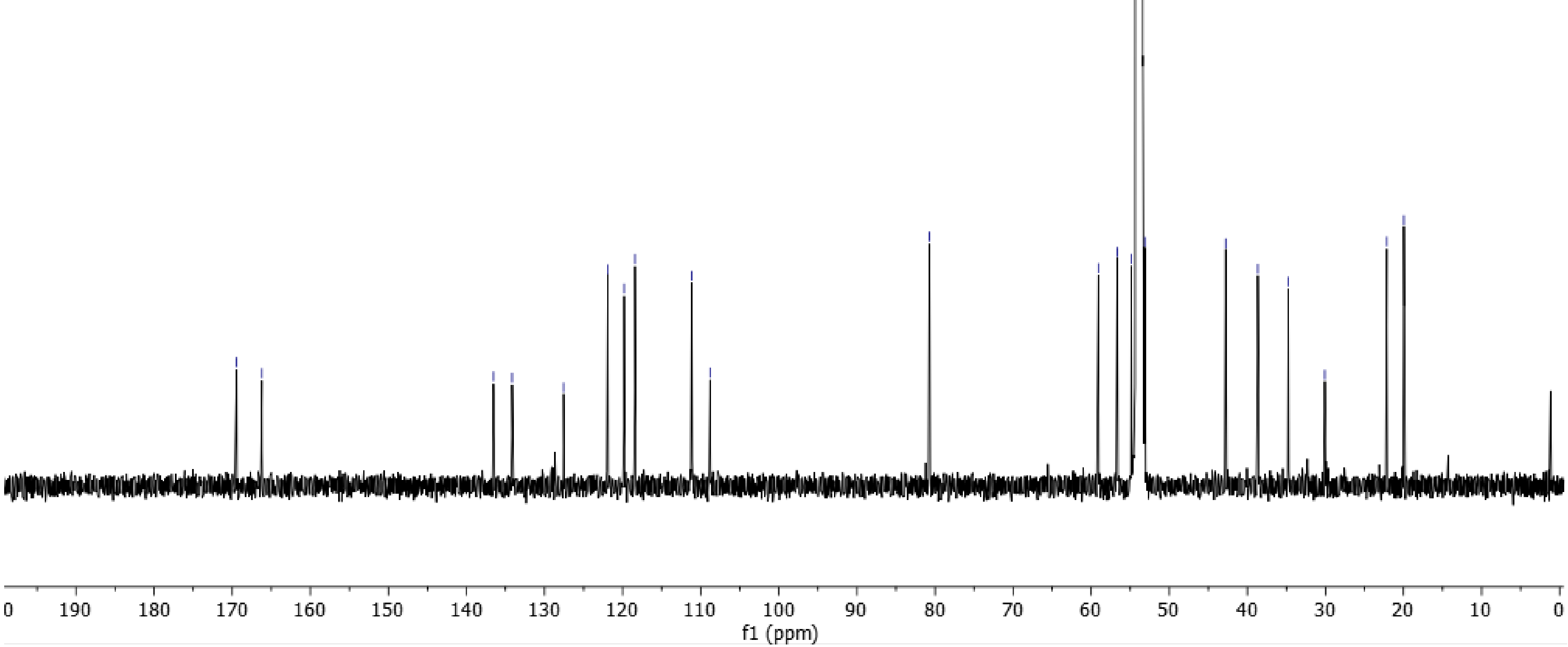

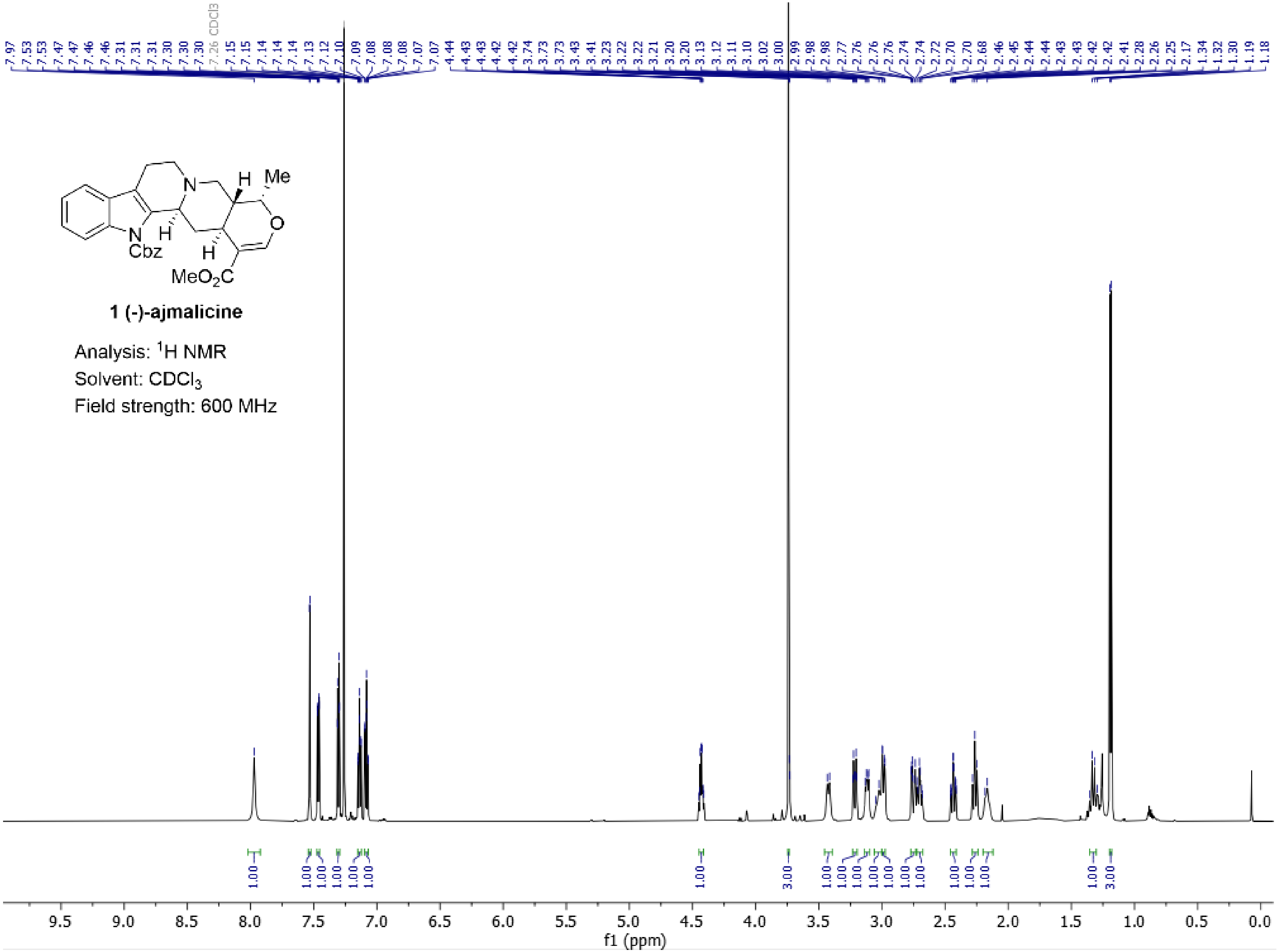
7.97 7.53 7.53 7.47 7.47 7.46 7.46 7.31 7.31 7.31 7.30 7.30 7.26 CDCl3 7.15 7.15 7.14 7.14 7.13 7.12 7.10 7.09 7.08 7.08 7.07 7.07 4.44 4.43 4.43 4.42 4.42 3.74 3.73 3.43 3.41 3.23 3.22 3.21 3.21 3.20 3.20 3.13 3.12 3.11 3.10 3.02 3.00 2.99 2.98 2.98 2.77 2.76 2.76 2.74 2.74 2.72 2.70 2.70 2.68 2.46 2.45 2.44 2.44 2.43 2.43 2.42 2.42 2.41 2.28 2.26 2.25 2.17 1.34 1.32 1.30 1.19 1.18
N
H
Me
O
H
Cbz
H
MeO2C
1 (-)-ajmalicine
Analysis: 1H NMR
Solvent: CDCl3
Field strength: 600 MHz
1.00
1.00
1.00
1.00
1.00
1.00
1.00
3.00
1.00
1.00
1.00
1.00
1.00
1.00
1.00
1.00
1.00
1.00
1.00
3.00
9.5 9.0 8.5 8.0 7.5 7.0 6.5 6.0 5.5 5.0 4.5 4.0 3.5 3.0 2.5 2.0 1.5 1.0 0.5 0.0
f1 (ppm)

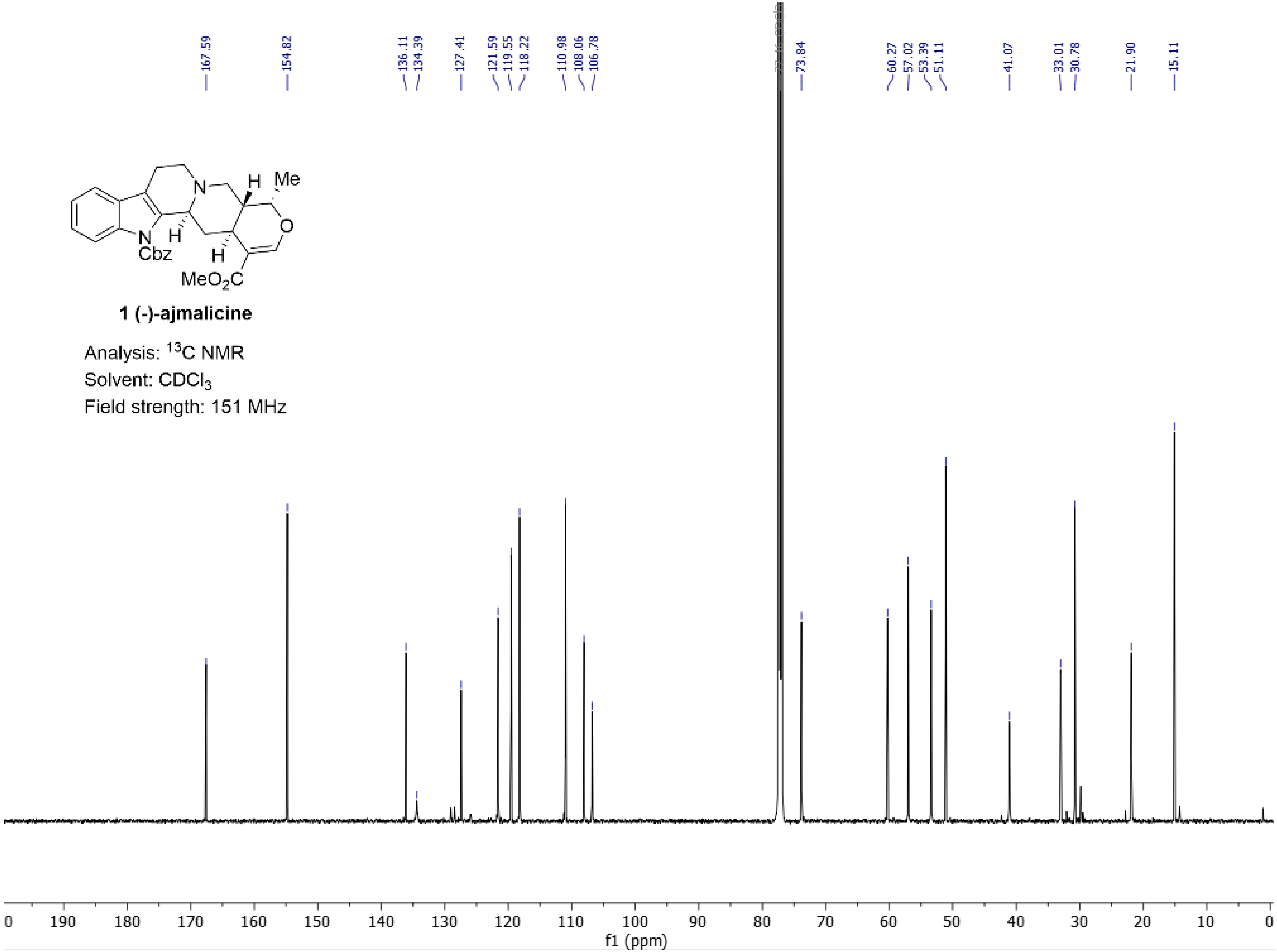

1 (-)-ajmalicine

Analysis: ^{13}C NMR

Solvent: $CDCl_3$

Field strength: 151 MHz

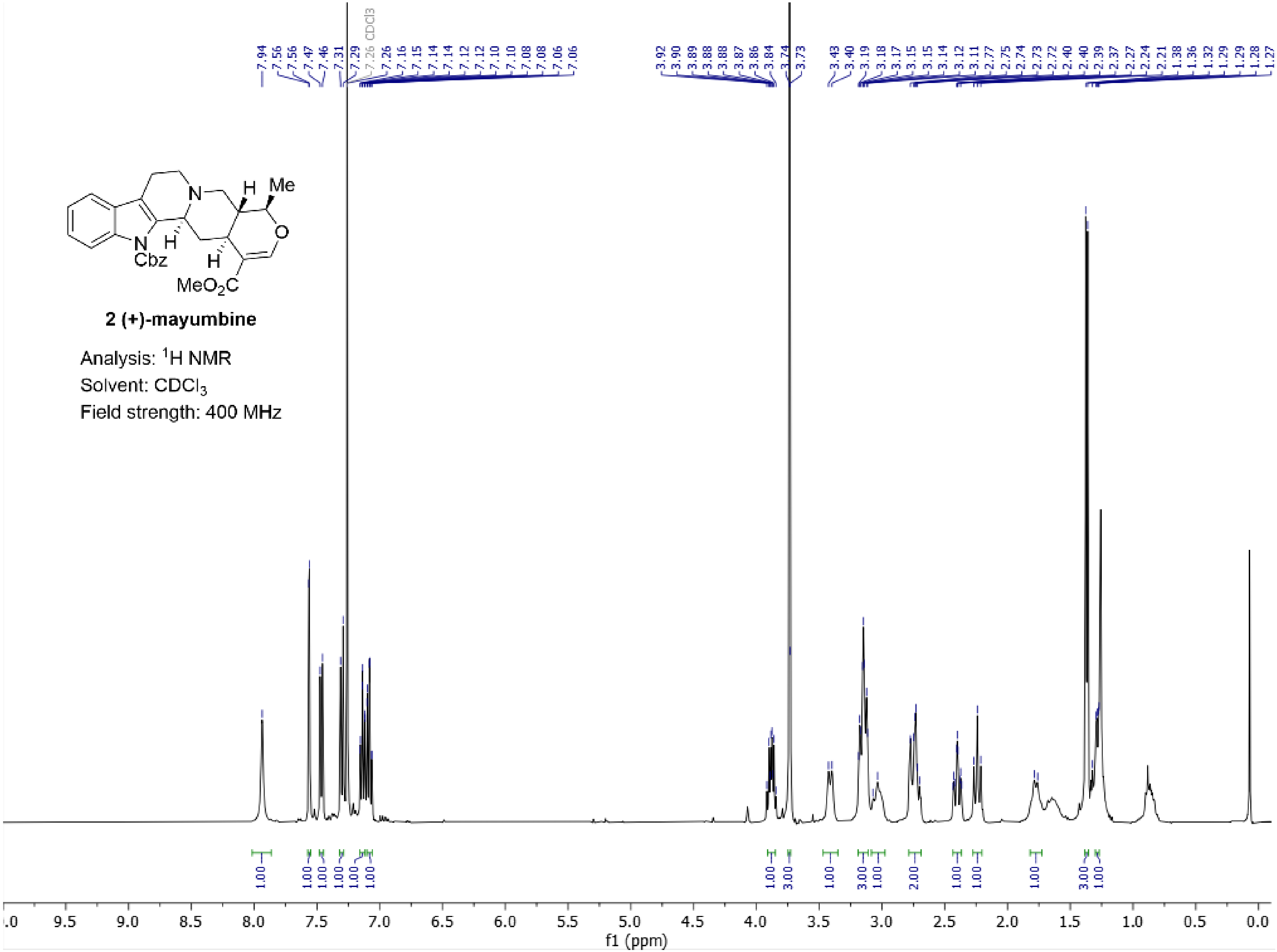

2 (+)-mayumbine
Analysis: 1H NMR
Solvent: CDCl3
Field strength: 400 MHz
N
H
Me
O
Cbz
MeO2C
7.94 7.56 7.56 7.47 7.46 7.31 7.29 7.26 CDCl3 7.26 7.16 7.15 7.14 7.14 7.12 7.12 7.10 7.10 7.08 7.08 7.06 7.06
3.92 3.90 3.89 3.88 3.88 3.87 3.86 3.84 3.74 3.73
3.43 3.40 3.19 3.18 3.17 3.15 3.15 3.14 3.12 3.11 2.77 2.75 2.74 2.73 2.72 2.40 2.40 2.39 2.37 2.27 2.24 2.21 1.38 1.36 1.32 1.29 1.29 1.28 1.27
f1 (ppm)

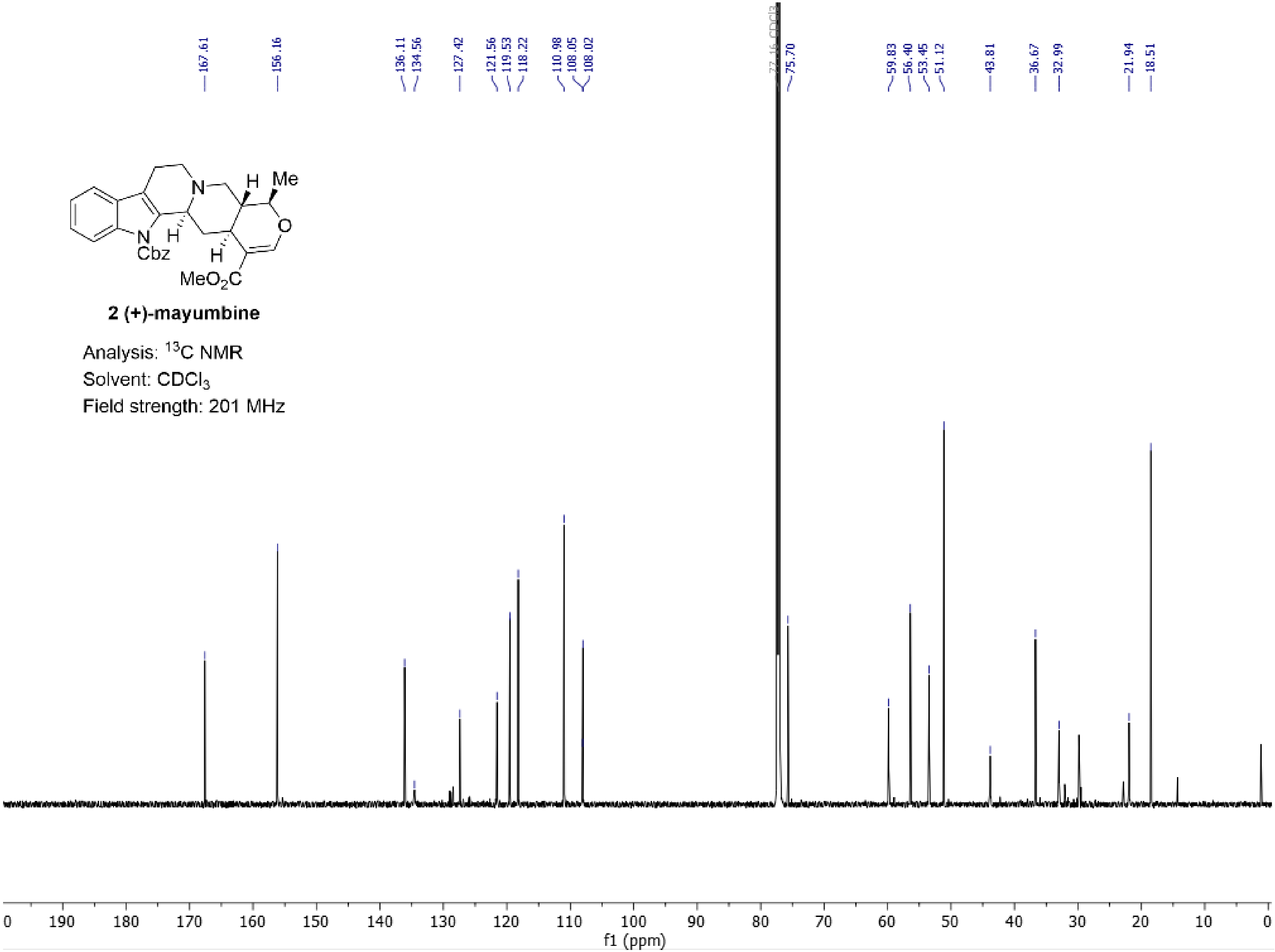

167.61
156.16
136.11
134.56
127.42
121.56
119.53
118.22
110.98
108.05
108.02
77.16 CDCl3
75.70
59.83
56.40
53.45
51.12
43.81
36.67
32.99
21.94
18.51
H
Me
N
O
N
H
H
Cbz
MeO_2C
2 (+)-mayumbine
Analysis: ^{13}C NMR
Solvent: $CDCl_3$
Field strength: 201 MHz
0
190
180
170
160
150
140
130
120
110
100
90
80
70
60
50
40
30
20
10
0
f1 (ppm)

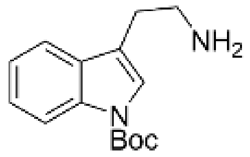

21

Analysis: ^{1}H NMR
Solvent: $CDCl_3$
Field strength: 400 MHz

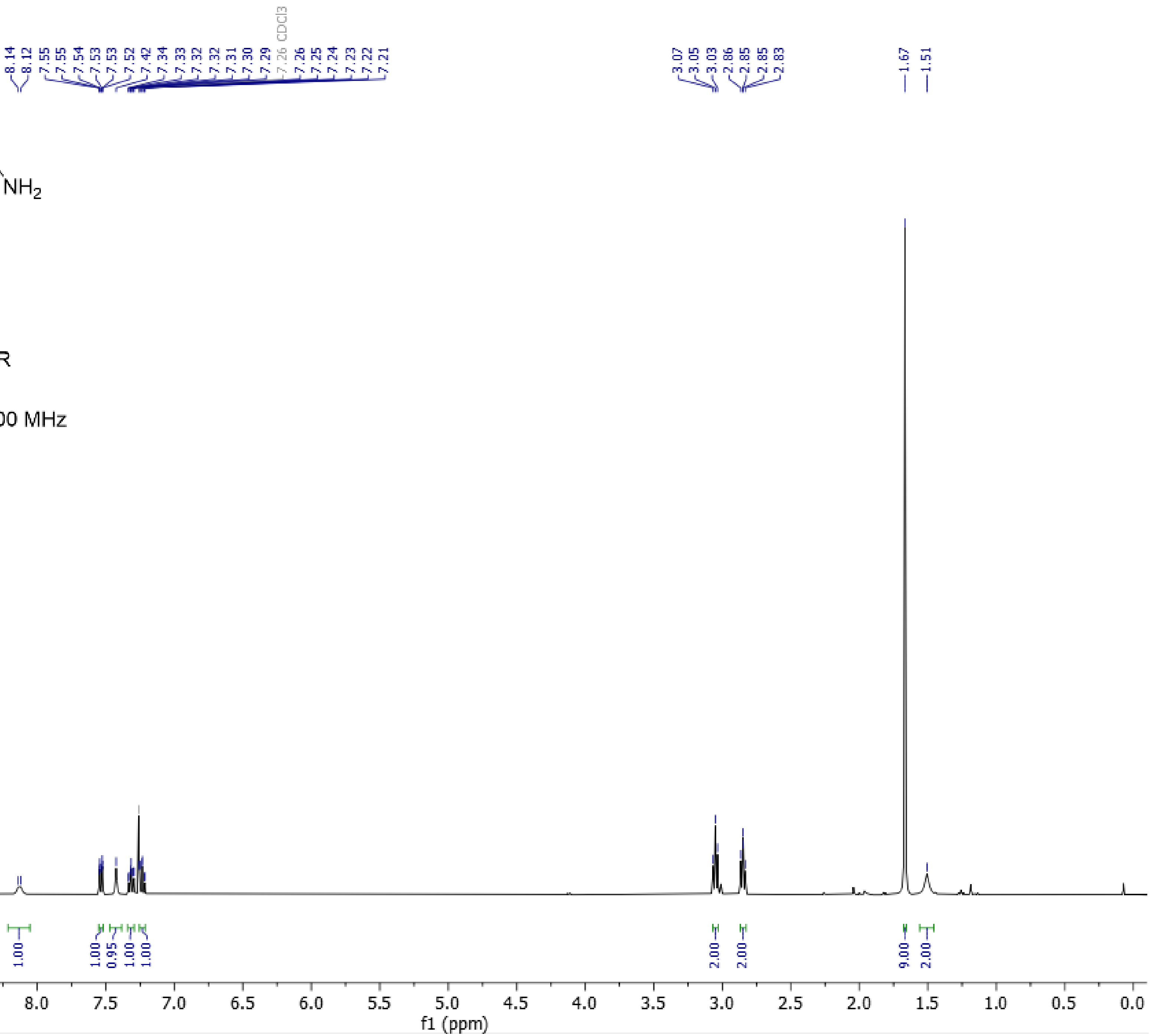

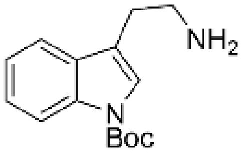

21

Analysis: ^{13}C NMR
Solvent: $CDCl_3$
Field strength: 101 MHz

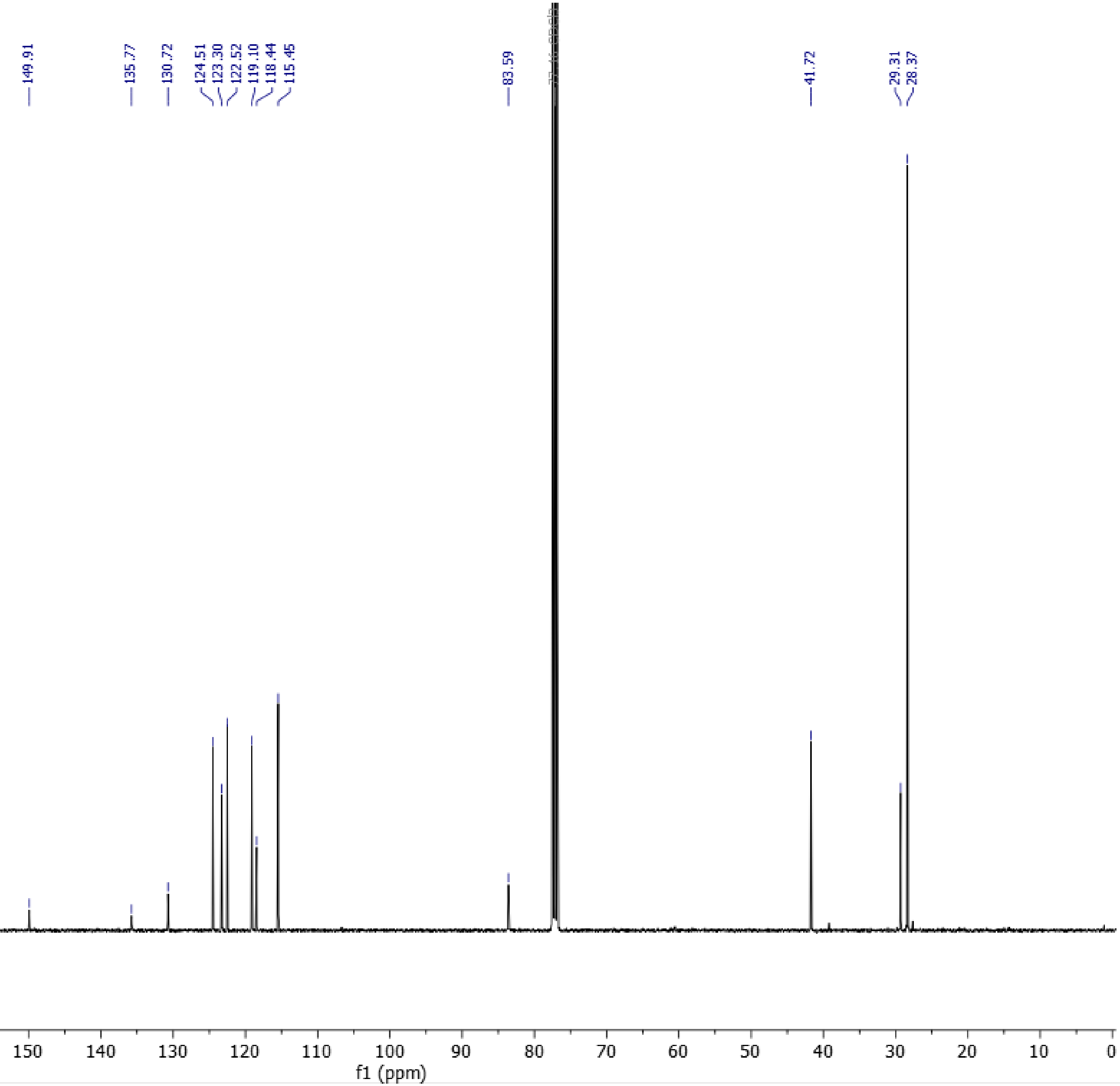

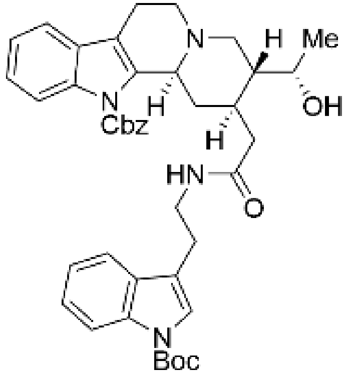

22a

Analysis: ^{1}H NMR
Solvent: $CDCl_3$
Field strength: 600 MHz

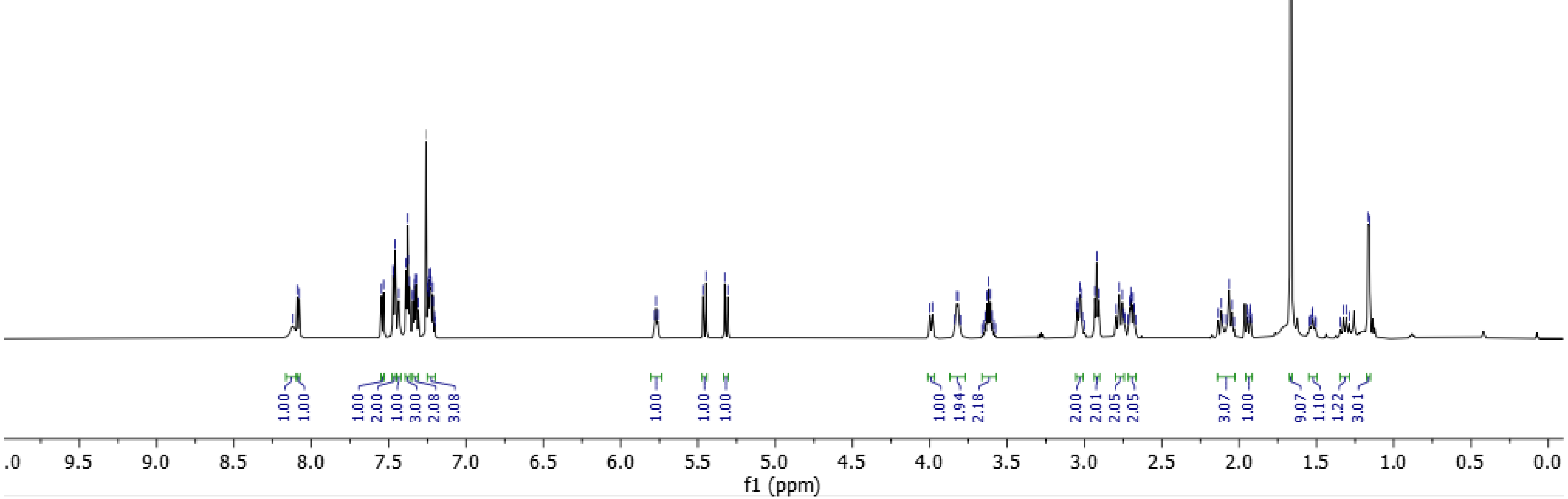

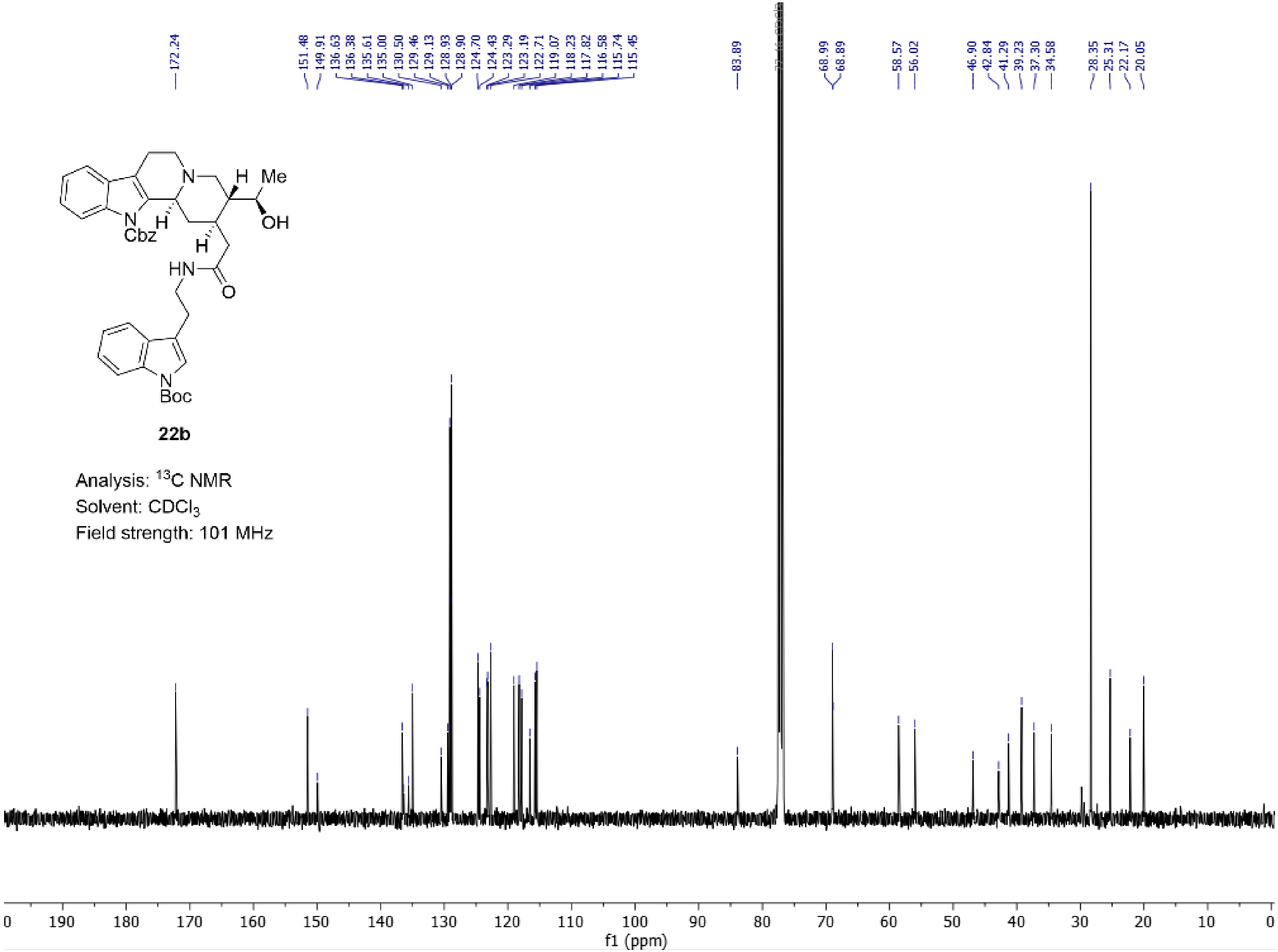

22b

Analysis: ^{13}C NMR

Solvent: $CDCl_3$

Field strength: 101 MHz

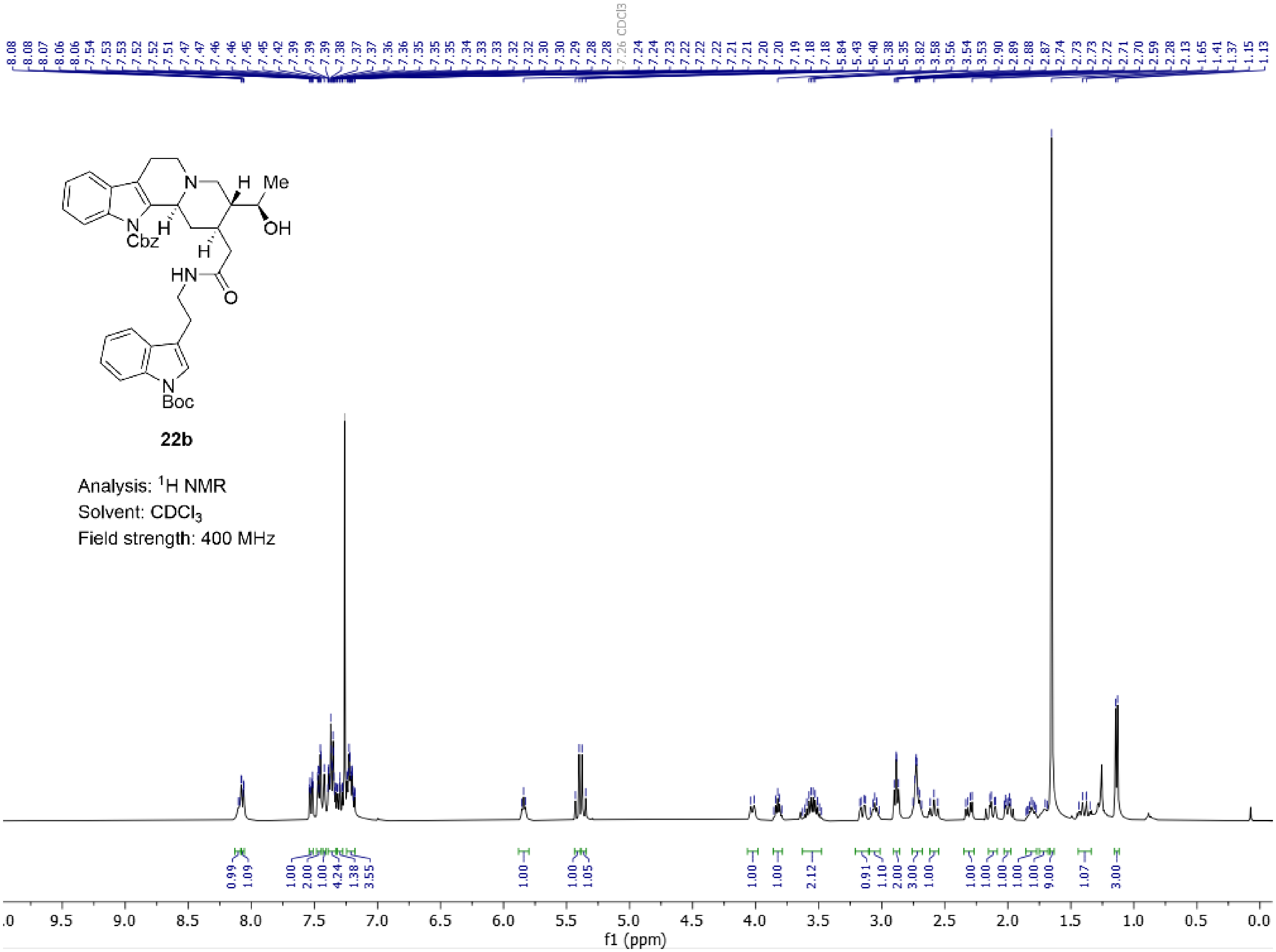

22b
Analysis: ^{1}H NMR
Solvent: $CDCl_3$
Field strength: 400 MHz
f1 (ppm)

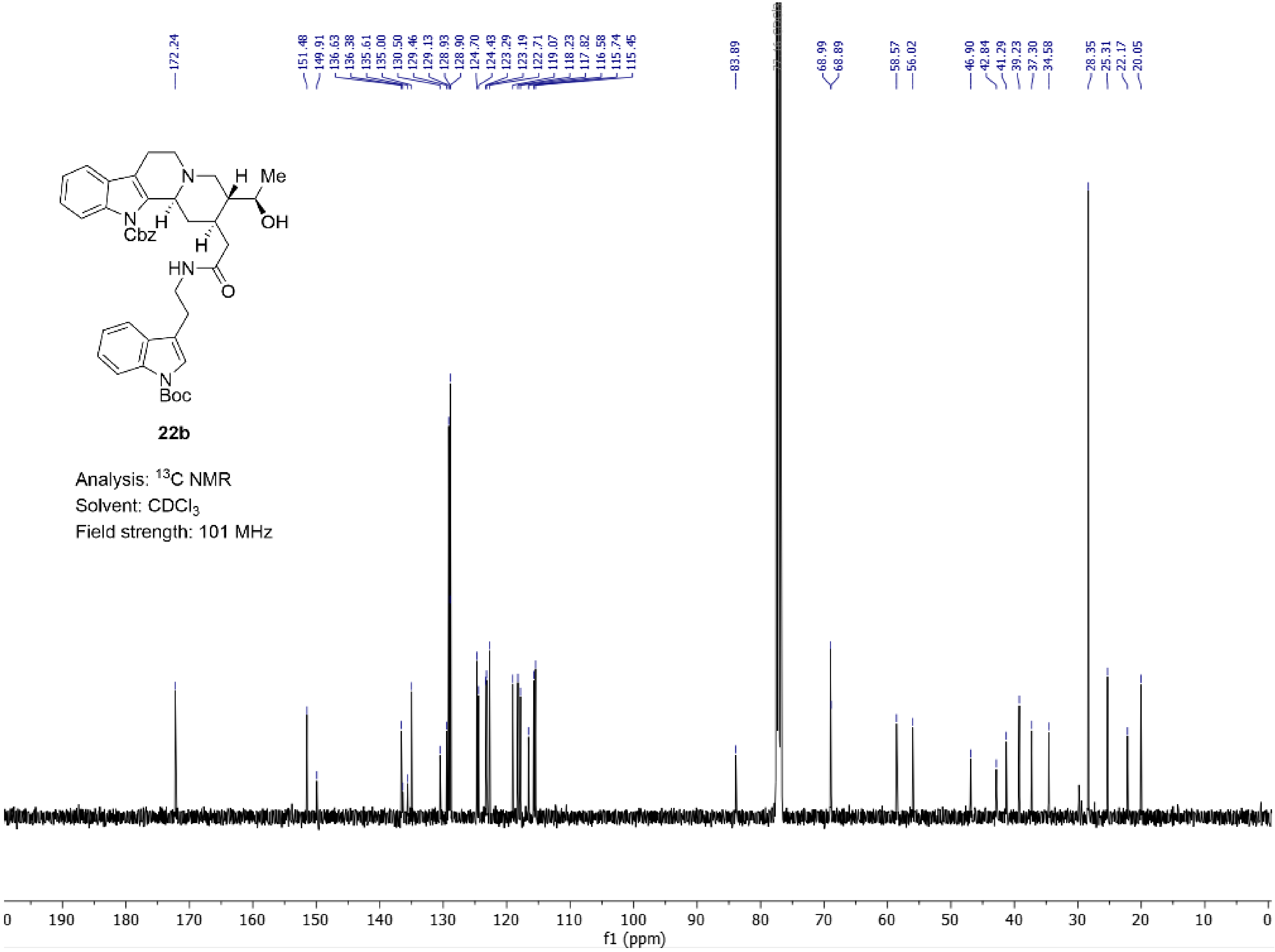
172.24
151.48
149.91
136.63
136.38
135.61
135.00
130.50
129.46
129.13
128.93
128.90
124.70
124.43
123.29
123.19
122.71
119.07
118.23
117.82
116.58
115.74
115.45
83.89
68.99
68.89
58.57
56.02
46.90
42.84
41.29
39.23
37.30
34.58
28.35
25.31
22.17
20.05
N
H
Me
OH
Cbz
HN
O
Boc
22b
Analysis: ^{13}C NMR
Solvent: $CDCl_3$
Field strength: 101 MHz
0 190 180 170 160 150 140 130 120 110 100 90 80 70 60 50 40 30 20 10 0
f1 (ppm)

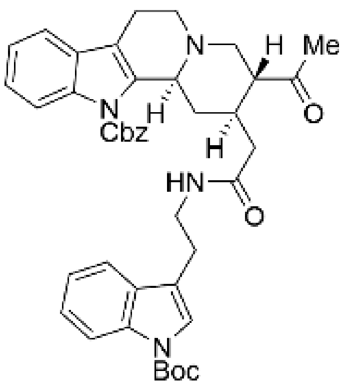

23

Analysis: ^{1}H NMR
Solvent: $CDCl_3$
Field strength: 400 MHz

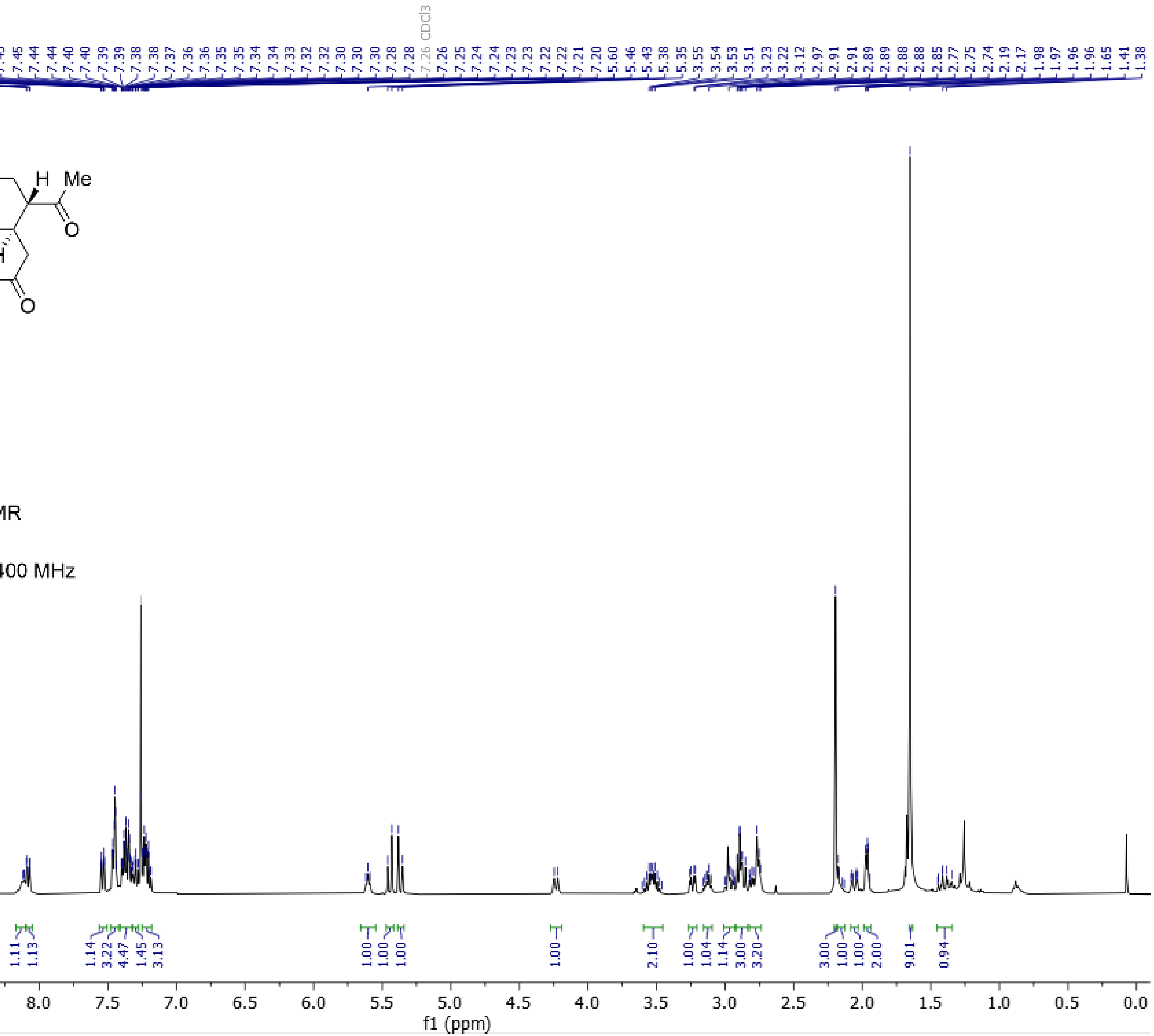

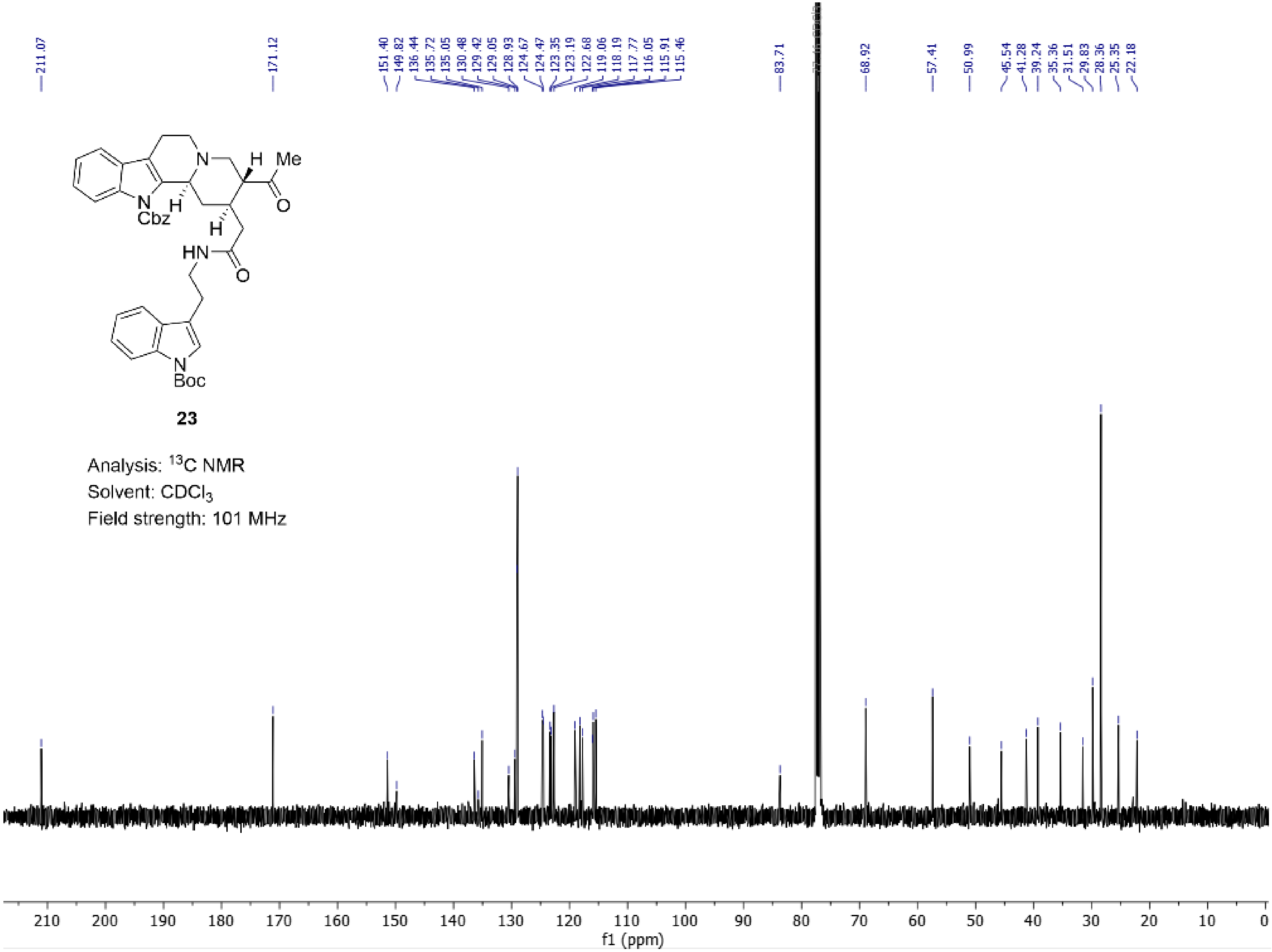
211.07
171.12
151.40
149.82
136.44
135.72
135.05
130.48
129.42
129.05
128.93
124.67
124.47
123.35
123.19
122.68
119.06
118.19
117.77
116.05
115.91
115.46
83.71
68.92
57.41
50.99
45.54
41.28
39.24
35.36
31.51
29.83
28.36
25.35
22.18
H
Me
N
O
N
Cbz
H
H
HN
O
N
Boc
23
Analysis: ^{13}C NMR
Solvent: $CDCl_3$
Field strength: 101 MHz
210
200
190
180
170
160
150
140
130
120
110
100
90
80
70
60
50
40
30
20
10
0
f1 (ppm)

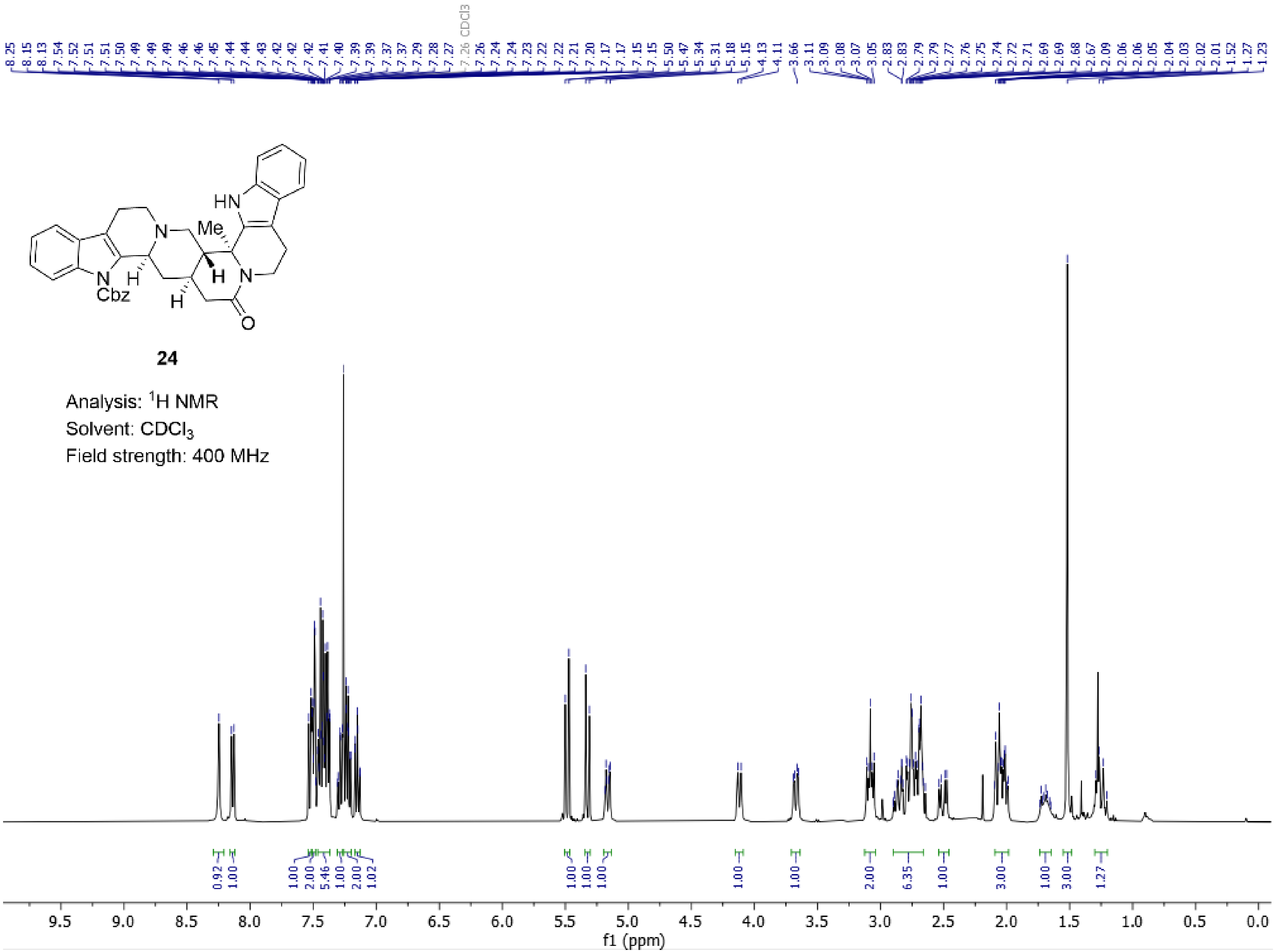
8.25 8.15 8.13 7.54 7.52 7.51 7.51 7.50 7.49 7.49 7.49 7.46 7.46 7.45 7.44 7.44 7.43 7.42 7.42 7.42 7.41 7.40 7.39 7.39 7.37 7.37 7.29 7.28 7.27 7.26 CDCl3 7.26 7.24 7.24 7.23 7.22 7.22 7.21 7.20 7.17 7.17 7.15 7.15 5.50 5.47 5.34 5.31 5.18 5.15 4.13 4.11 3.66 3.11 3.09 3.08 3.07 3.05 2.83 2.83 2.79 2.79 2.77 2.76 2.75 2.74 2.72 2.71 2.69 2.69 2.68 2.67 2.09 2.06 2.05 2.04 2.03 2.02 2.01 1.52 1.27 1.23
HN
Me
N
N
H
H
H
Cbz
O
24
Analysis: ^{1}H NMR
Solvent: $CDCl_3$
Field strength: 400 MHz
0.92 1.00 1.00 2.00 5.46 1.00 2.00 1.02 1.00 1.00 1.00 1.00 1.00 2.00 6.35 1.00 3.00 1.00 3.00 1.27
9.5 9.0 8.5 8.0 7.5 7.0 6.5 6.0 5.5 5.0 4.5 4.0 3.5 3.0 2.5 2.0 1.5 1.0 0.5 0.0
f1 (ppm)

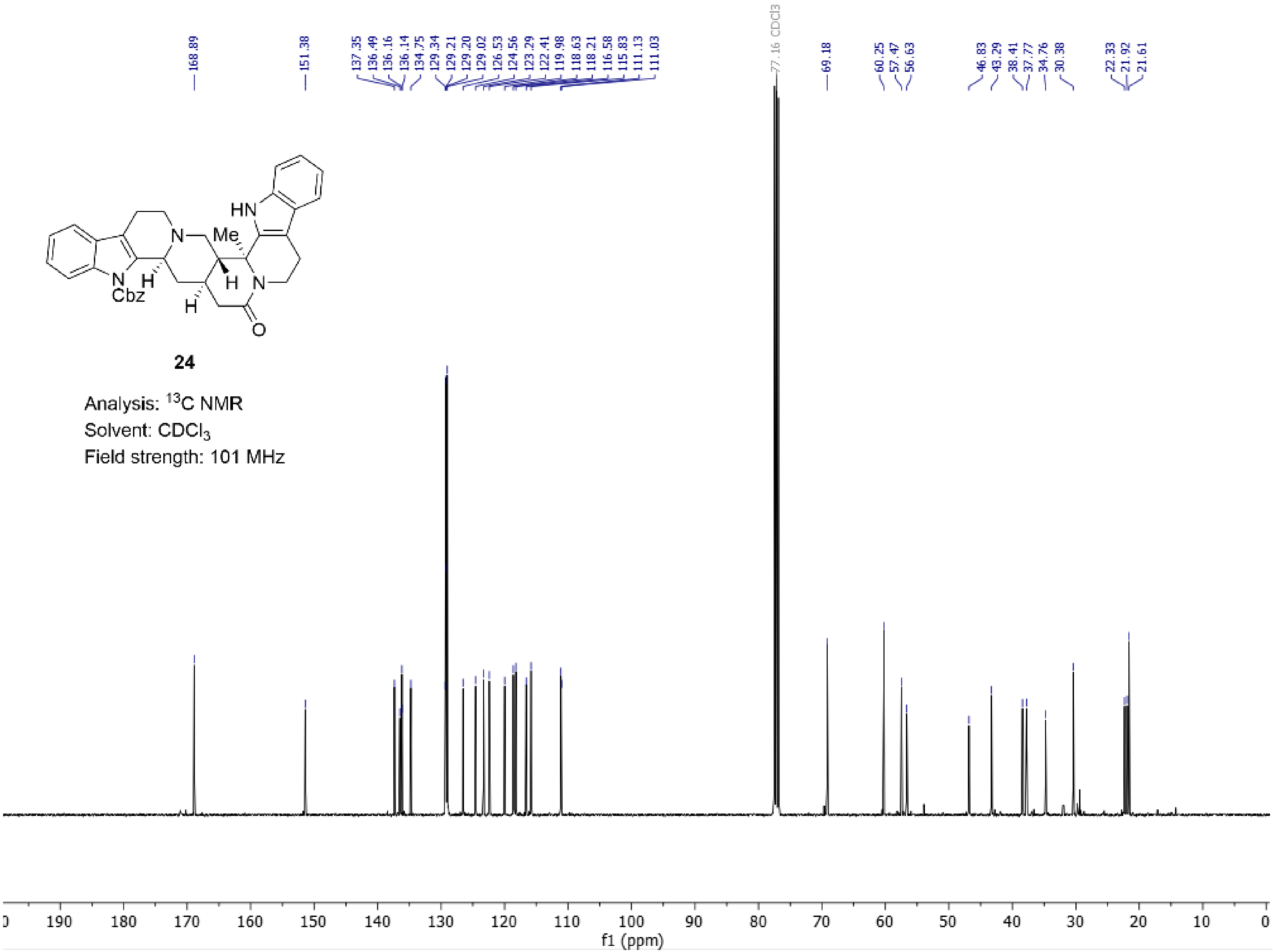
168.89
151.38
137.35
136.49
136.16
136.14
134.75
129.34
129.21
129.20
129.02
126.53
124.56
123.29
122.41
119.98
118.63
118.21
116.58
115.83
111.13
111.03
77.16 CDCl3
69.18
60.25
57.47
56.63
46.83
43.29
38.41
37.77
34.76
30.38
22.33
21.92
21.61
HN
N
Me
H
N
Cbz
H
N
H
O
24
Analysis: ^{13}C NMR
Solvent: $CDCl_3$
Field strength: 101 MHz
190
180
170
160
150
140
130
120
110
100
90
80
70
60
50
40
30
20
10
0
f1 (ppm)

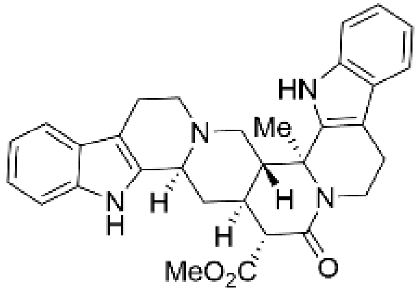

25

Analysis: ^{1}H NMR
Solvent: $CDCl_3$
Field strength: 400 MHz

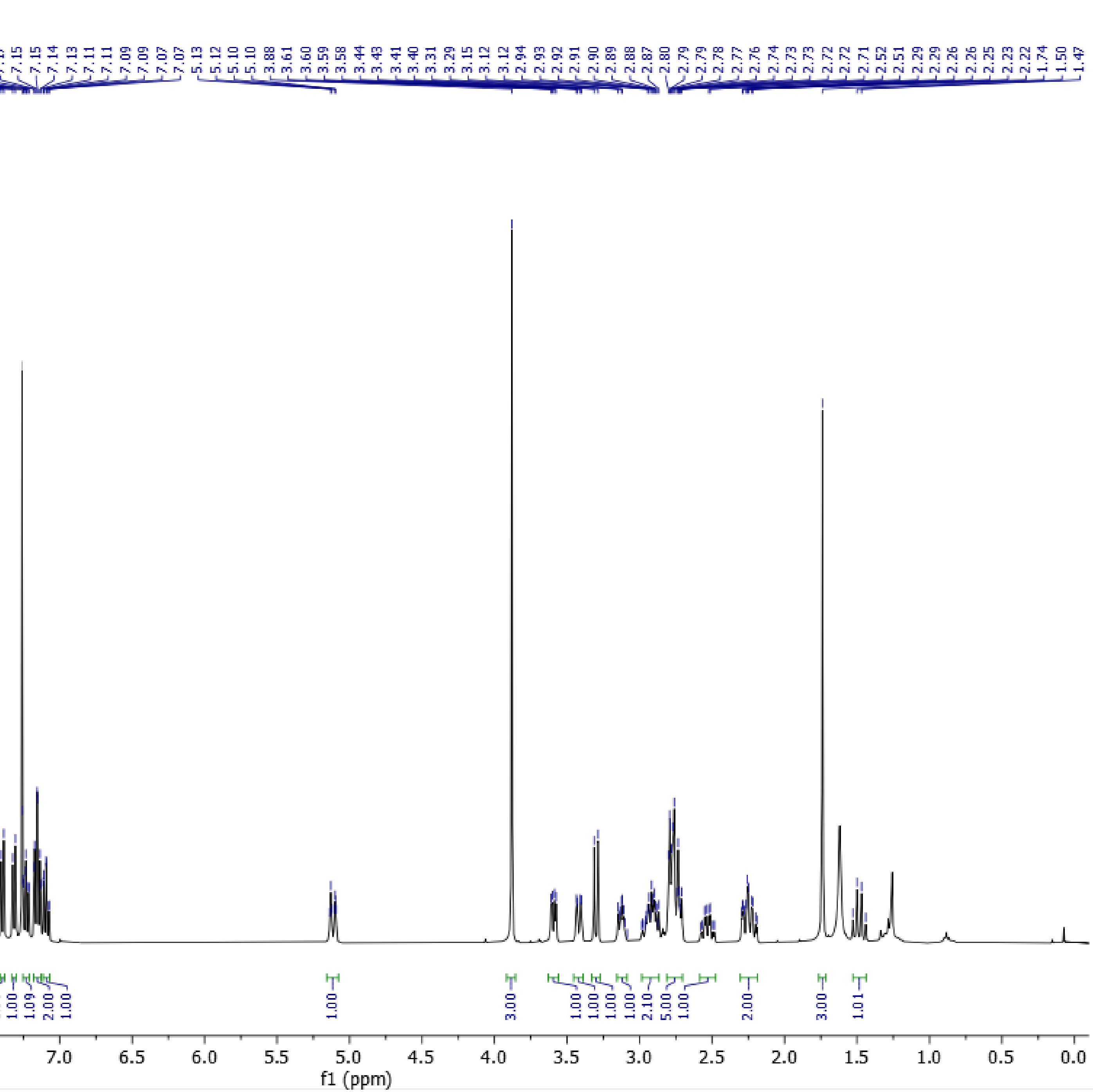

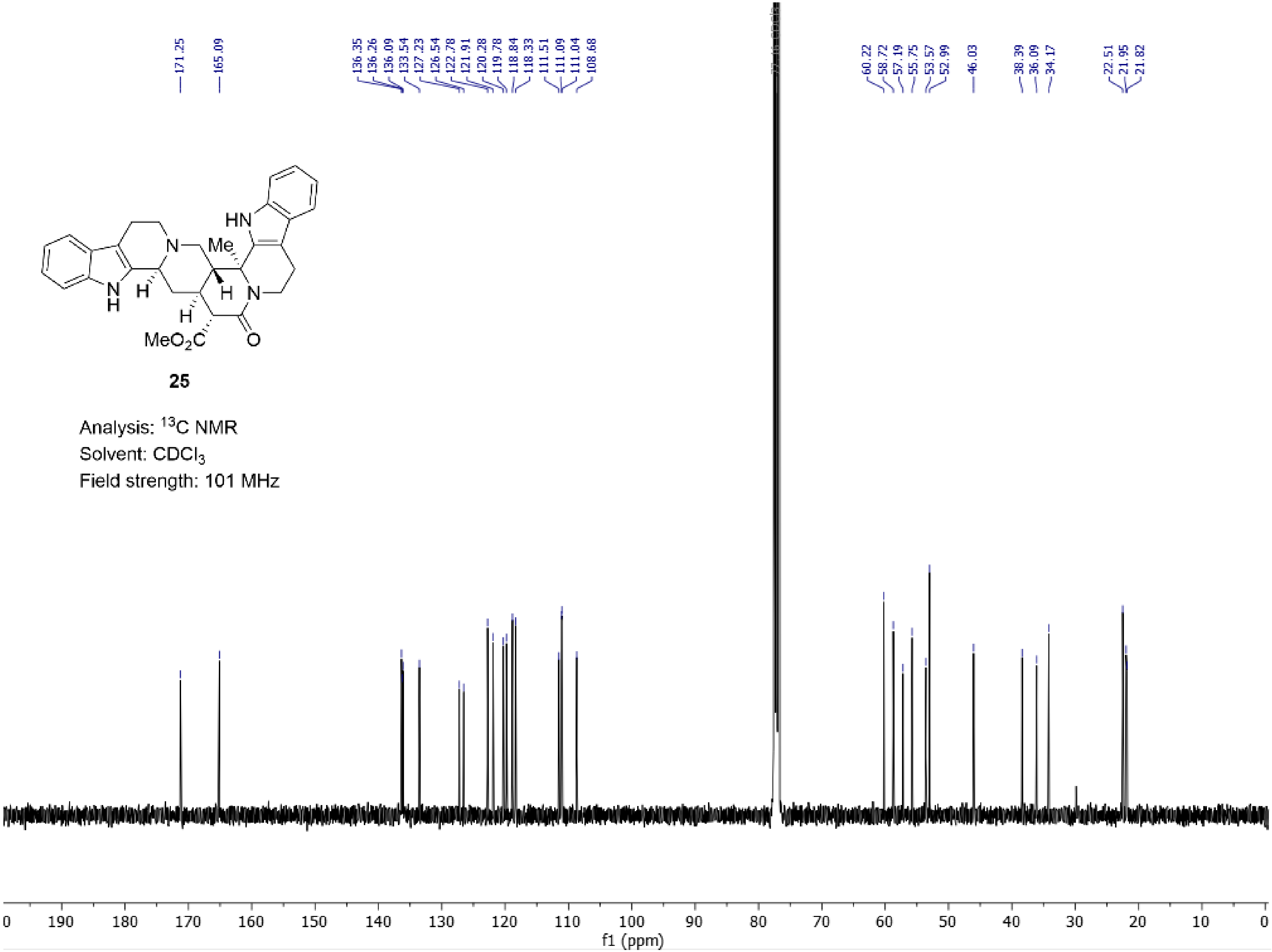
171.25
165.09
136.35
136.26
136.09
133.54
127.23
126.54
122.78
121.91
120.28
119.78
118.84
118.33
111.51
111.09
111.04
108.68
60.22
58.72
57.19
55.75
53.57
52.99
46.03
38.39
36.09
34.17
22.51
21.95
21.82
HN
N
Me
H
N
H
H
MeO2C
O
25
Analysis: 13C NMR
Solvent: CDCl3
Field strength: 101 MHz
f1 (ppm)

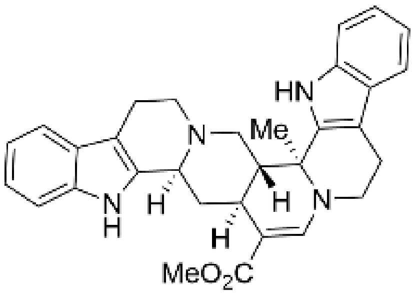

3 (-)-roxburghine C

Analysis: ^{1}H NMR
Solvent: Acetone-d_6
Field strength: 600 MHz

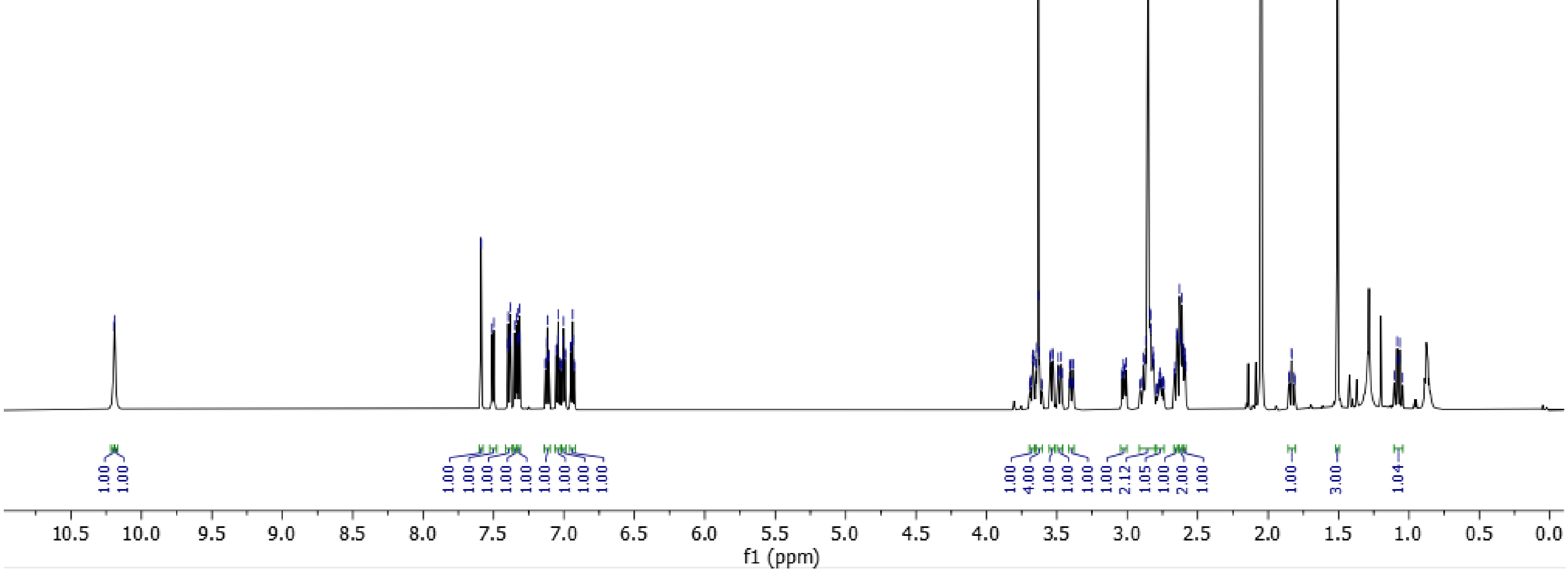

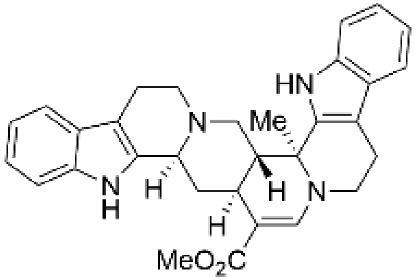

3 (-)-roxburghine C

Analysis: ^{13}C NMR
Solvent: Acetone-d_6
Field strength: 151 MHz

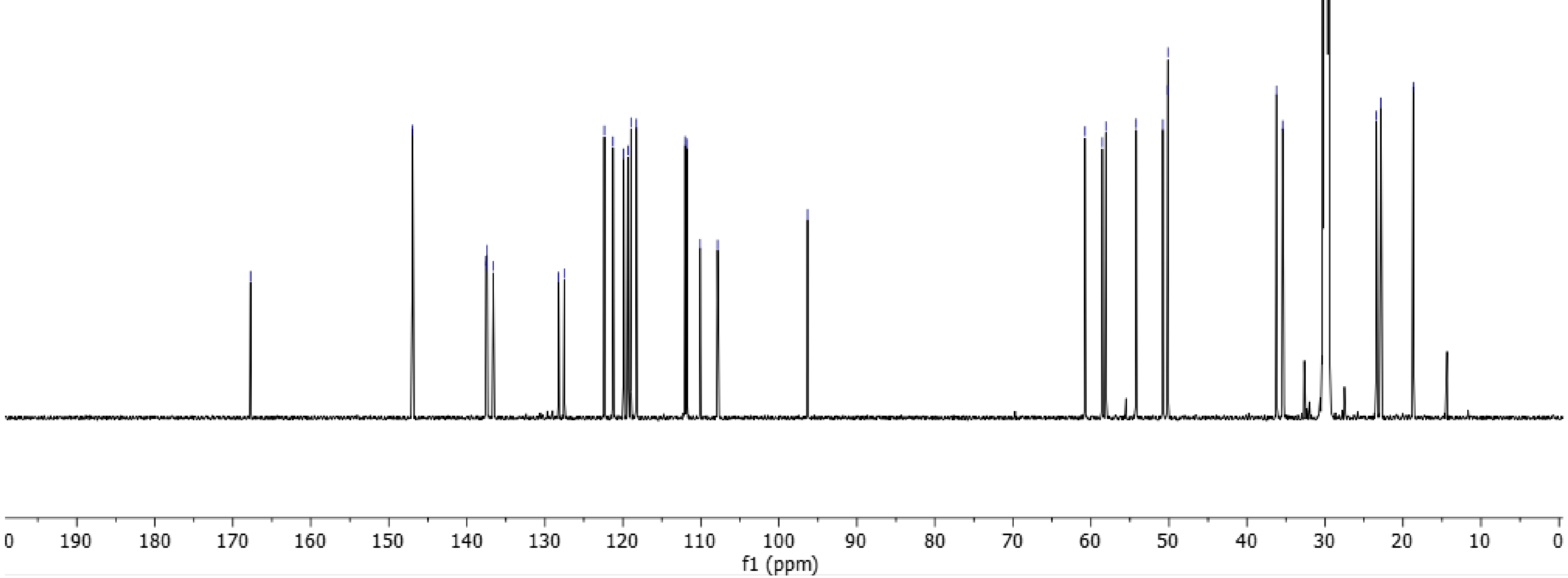

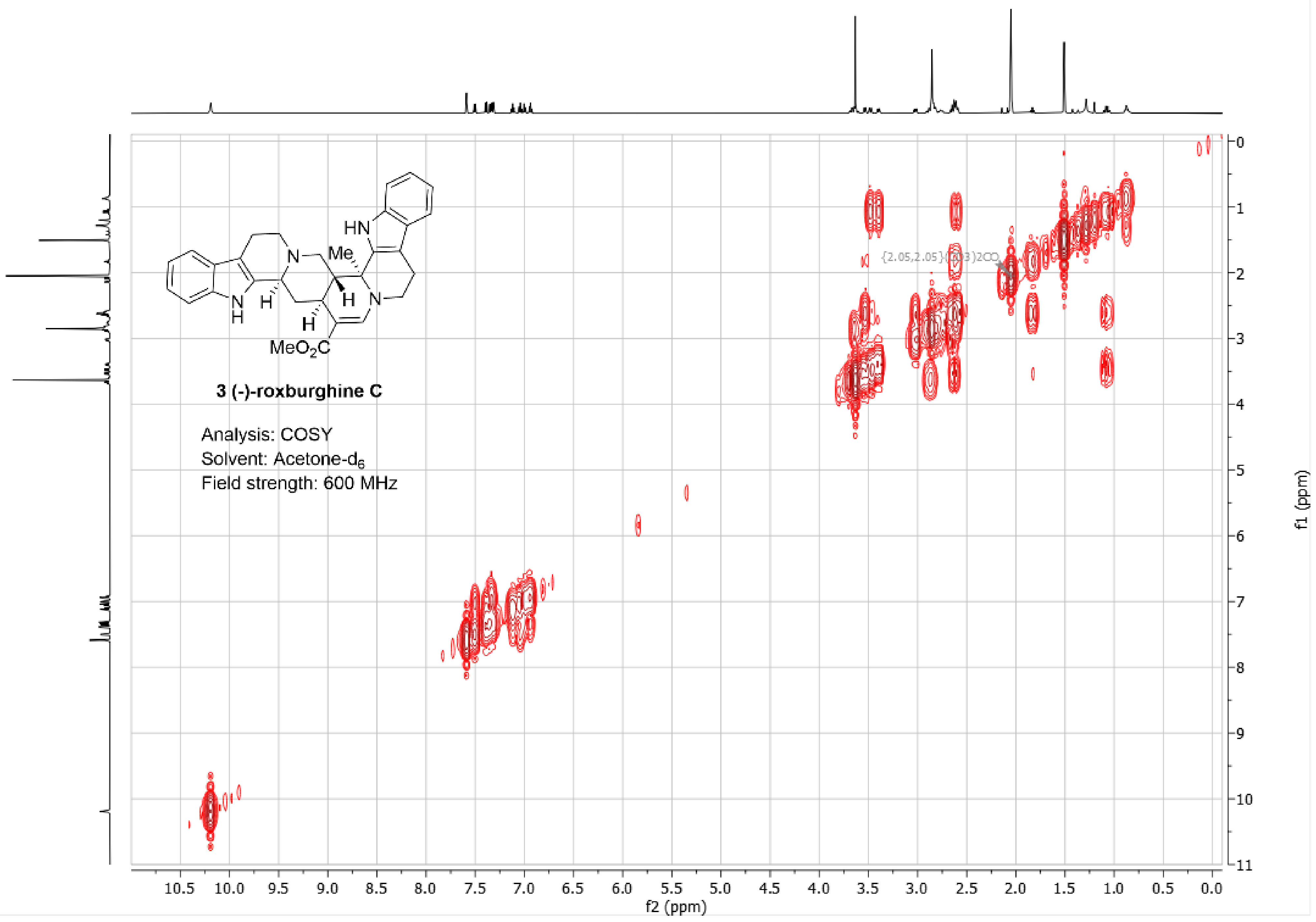

3 (-)-roxburghine C
Analysis: COSY
Solvent: Acetone-d6
Field strength: 600 MHz
f2 (ppm)
f1 (ppm)

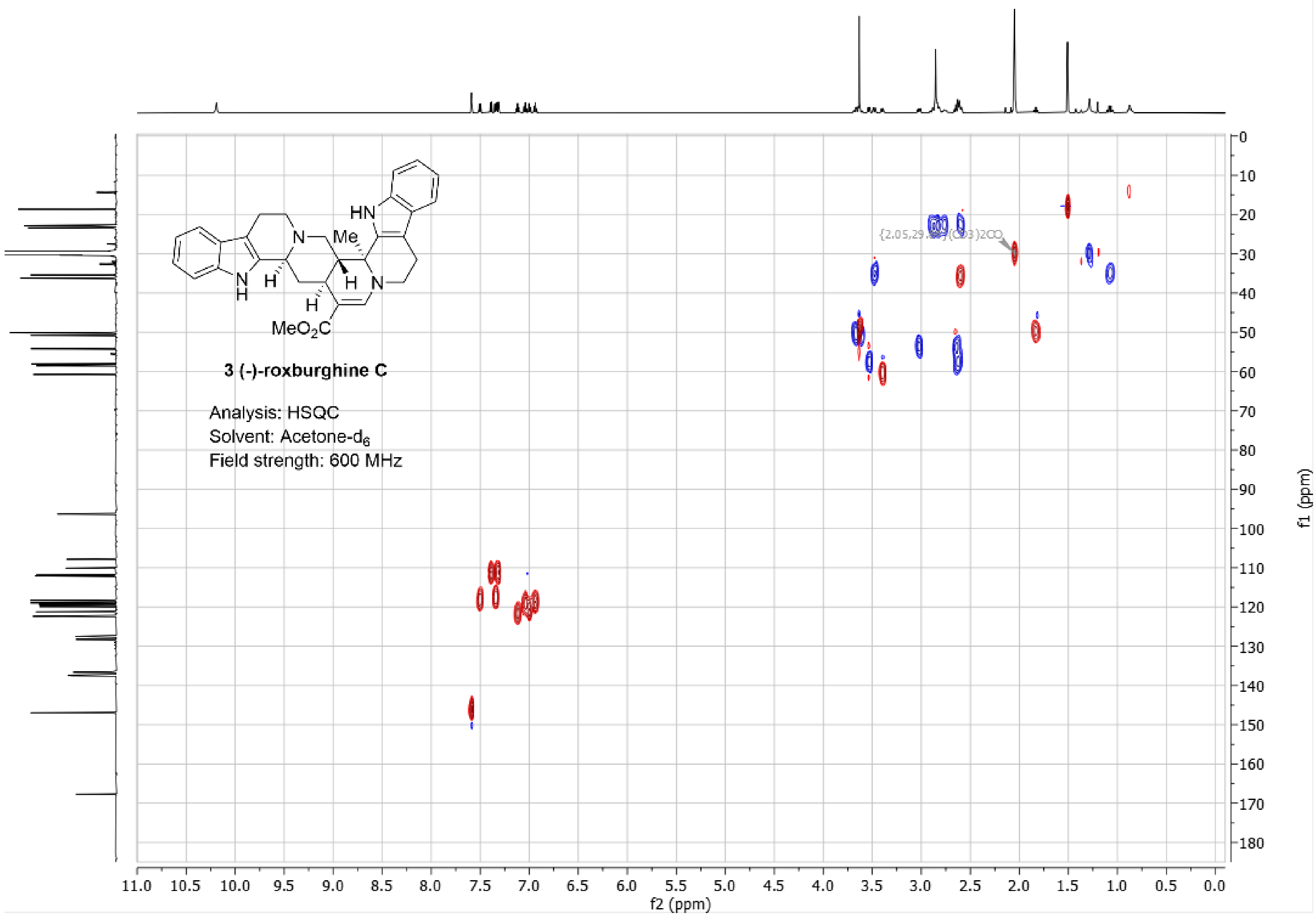

HN
N
Me
H
H
N
H
H
N
MeO2C
3 (-)-roxburghine C
Analysis: HSQC
Solvent: Acetone-d6
Field strength: 600 MHz
f2 (ppm)
f1 (ppm)

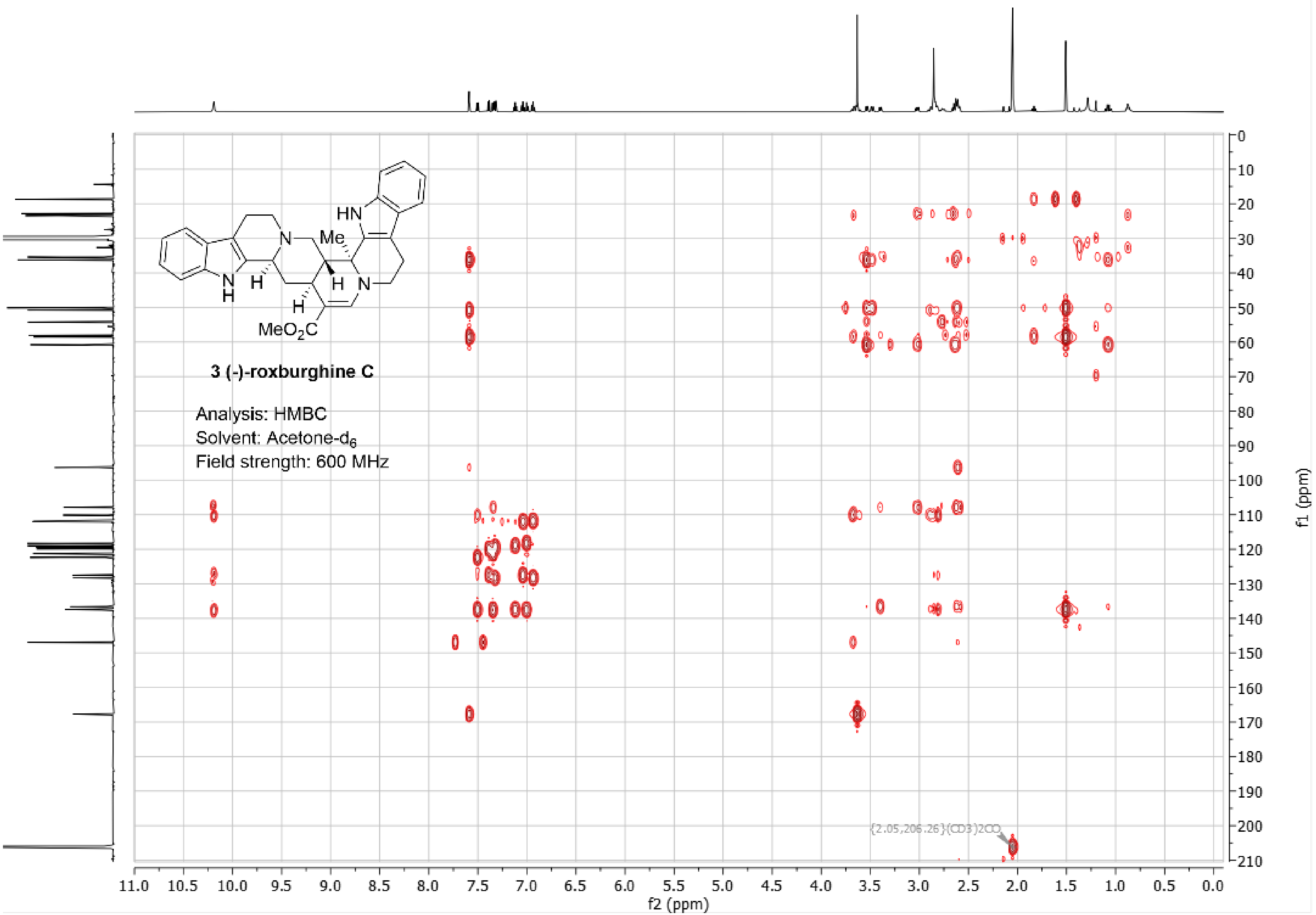
3 (-)-roxburghine C
Analysis: HMBC
Solvent: Acetone-d6
Field strength: 600 MHz
{2.05,206.26}(CD3)2CO
f2 (ppm)
f1 (ppm)

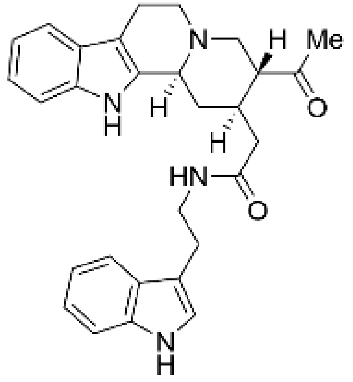

30

Analysis: ^{1}H NMR
Solvent: $CDCl_3$
Field strength: 400 MHz

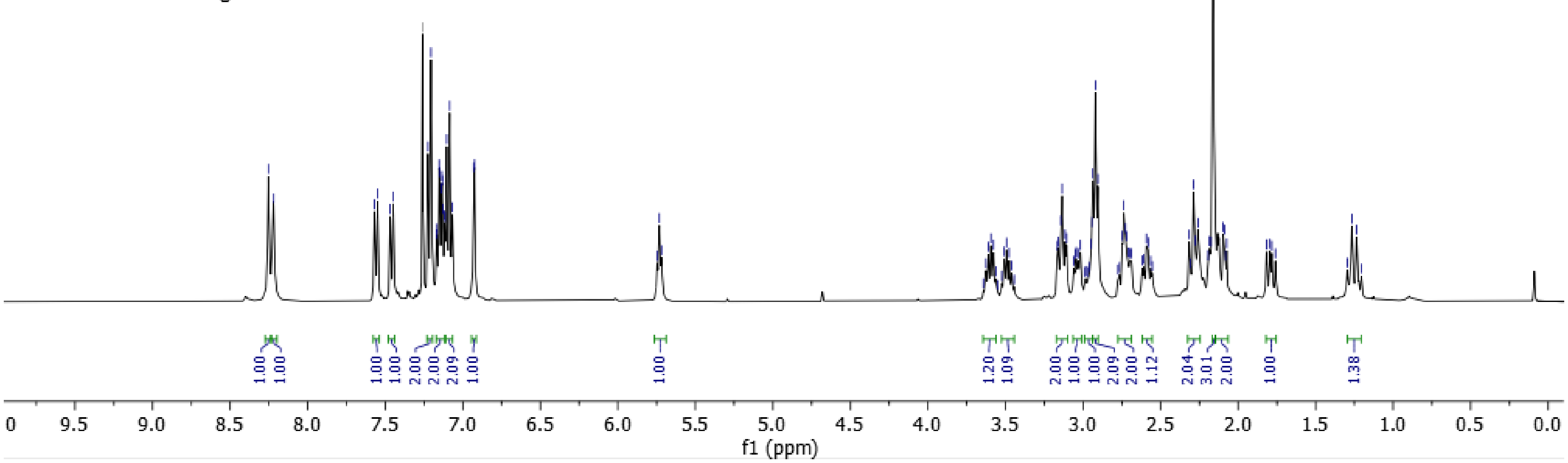

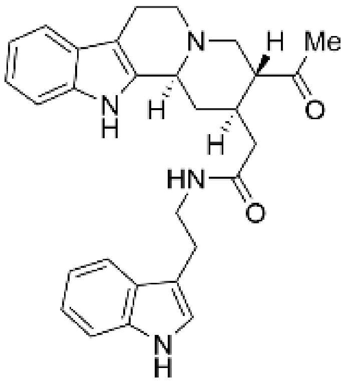

30

Analysis: ^{13}C NMR
Solvent: $CDCl_3$
Field strength: 101 MHz

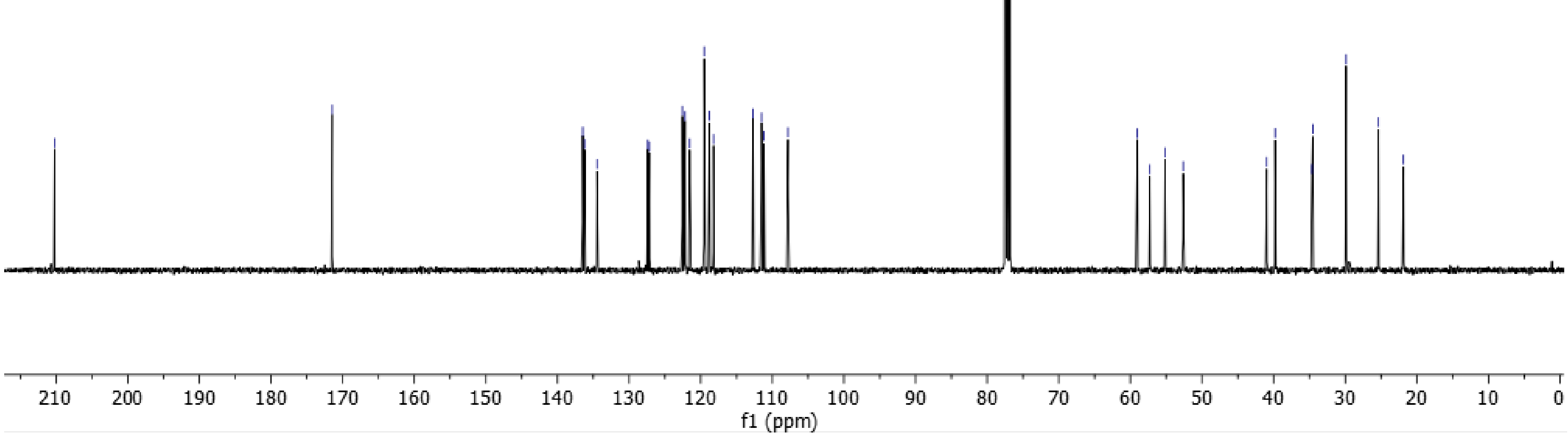

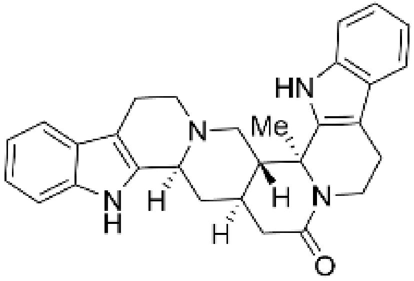

33a

Analysis: ^{1}H NMR
Solvent: Acetone-d_6
Field strength: 600 MHz

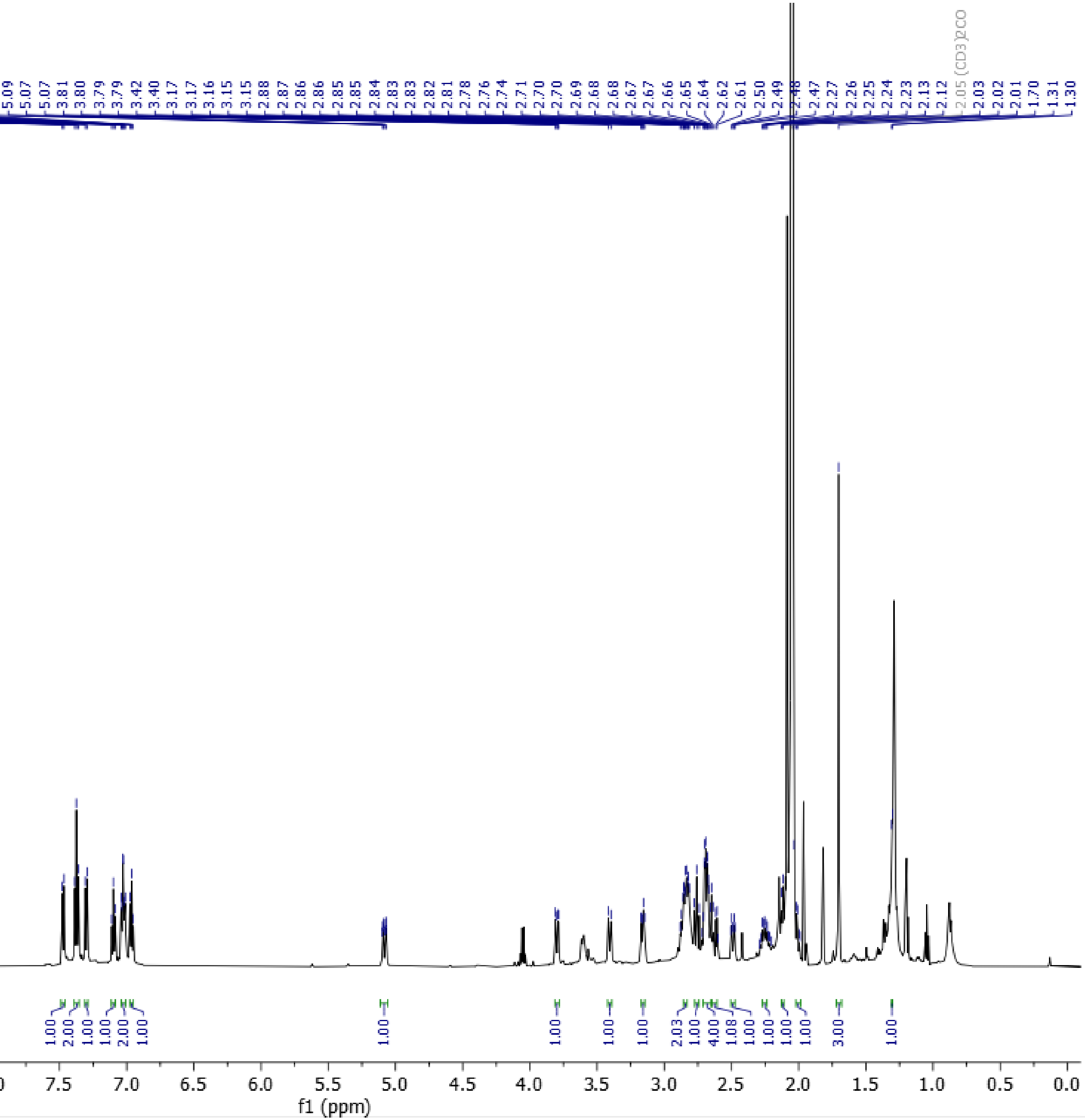

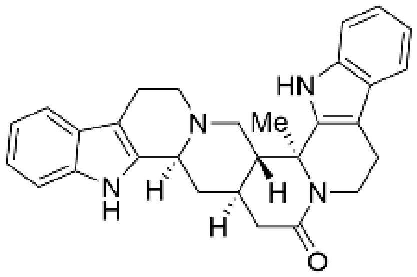

33a

Analysis: 1D NOE
Solvent: Acetone-d_6
Field strength: 400 MHz

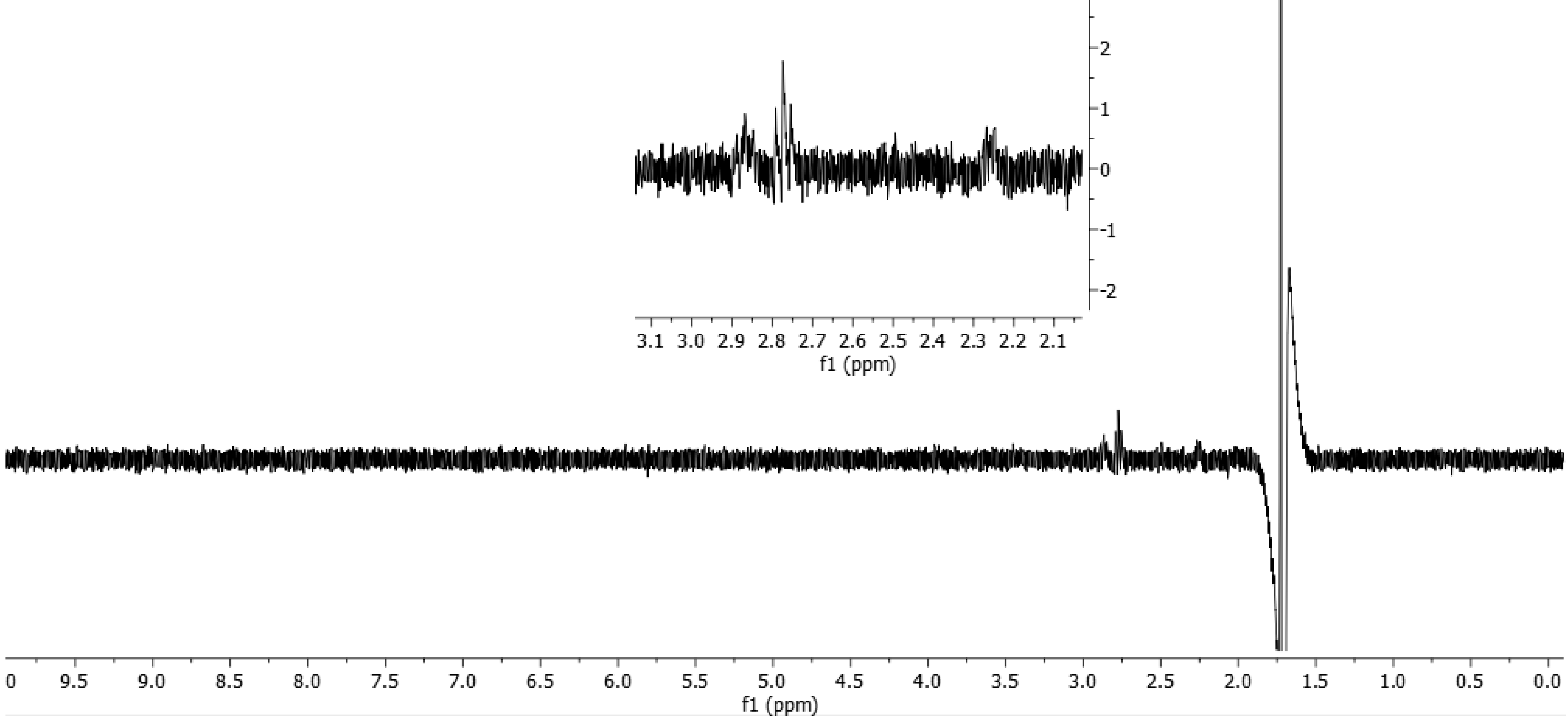

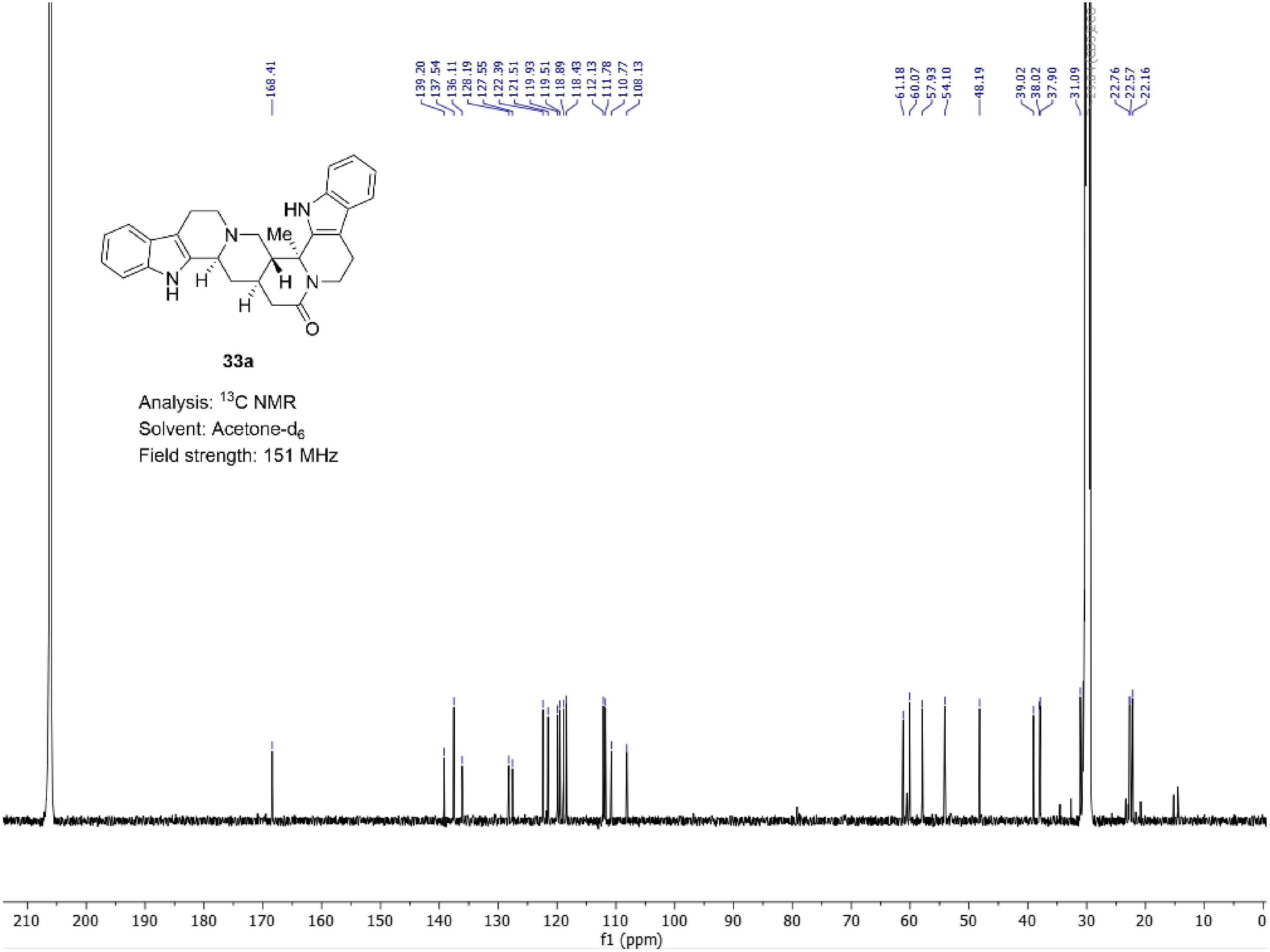

168.41
139.20
137.54
136.11
128.19
127.55
122.39
121.51
119.93
119.51
118.89
118.43
112.13
111.78
110.77
108.13
61.18
60.07
57.93
54.10
48.19
39.02
38.02
37.90
31.09
22.76
22.57
22.16
HN
N
Me
H
N
H
H
N
H
O
33a
Analysis: ¹³C NMR
Solvent: Acetone-d6
Field strength: 151 MHz
210
200
190
180
170
160
150
140
130
120
110
100
90
80
70
60
50
40
30
20
10
0
f1 (ppm)

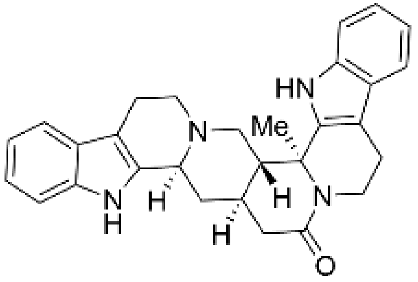

33a

Analysis: ^{1}H NMR
Solvent: $CDCl_3$
Field strength: 600 MHz

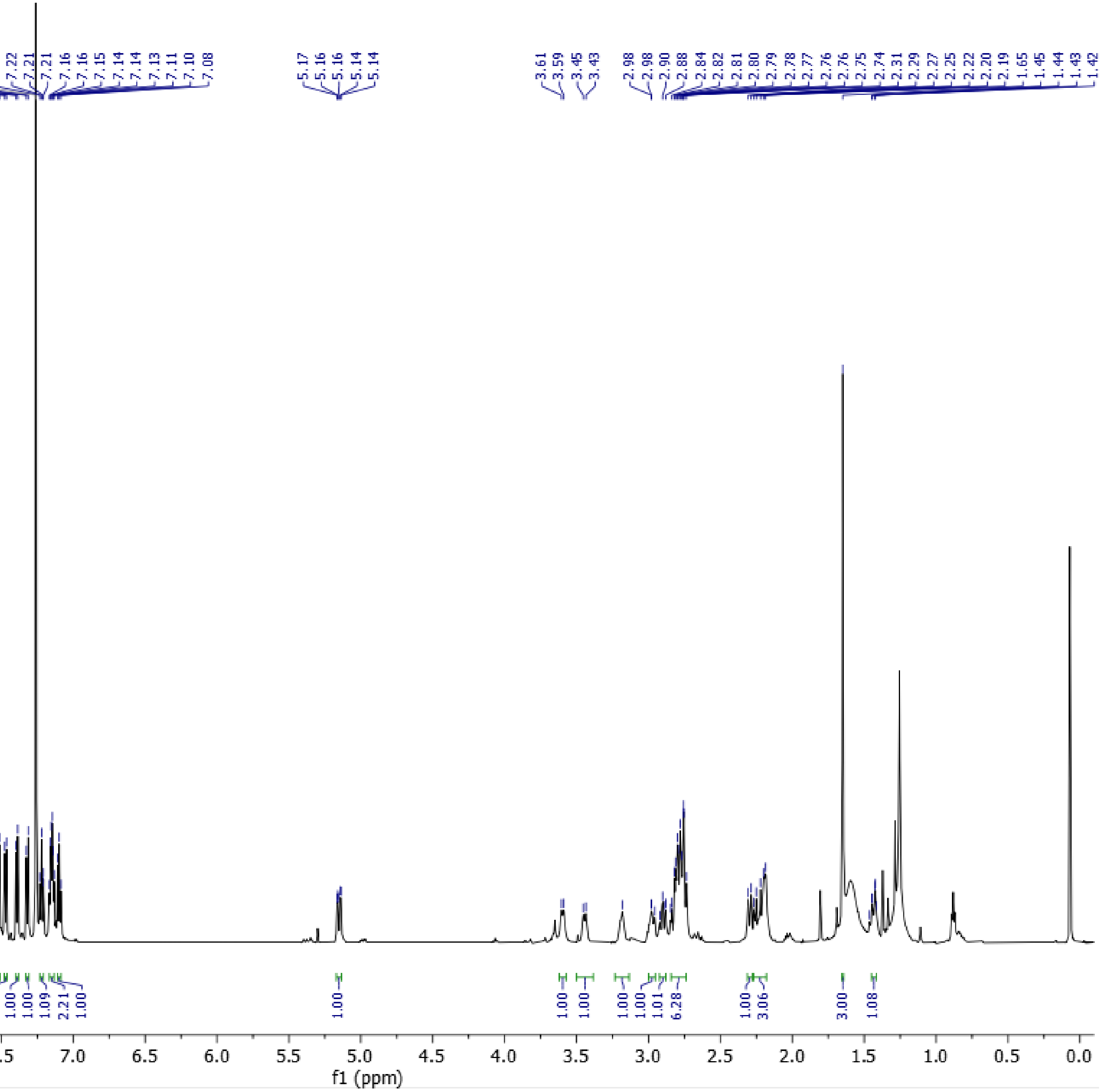

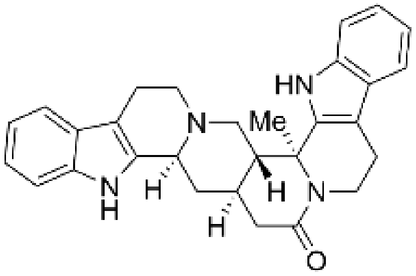

33a

Analysis: ^{13}C NMR
Solvent: $CDCl_3$
Field strength: 151 MHz

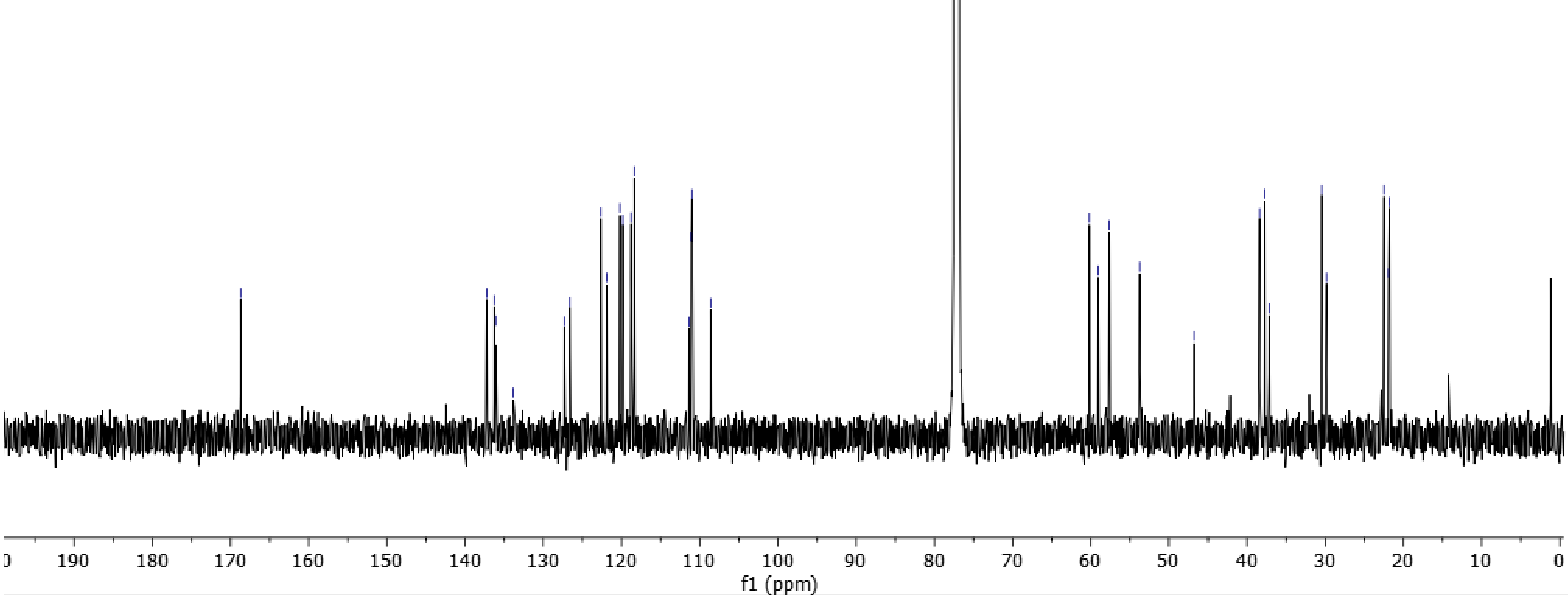

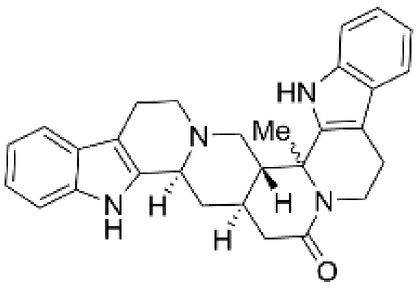

33a:33b, dr 1:1.2

Analysis: ^{1}H NMR
Solvent: $CDCl_3$
Field strength: 800 MHz

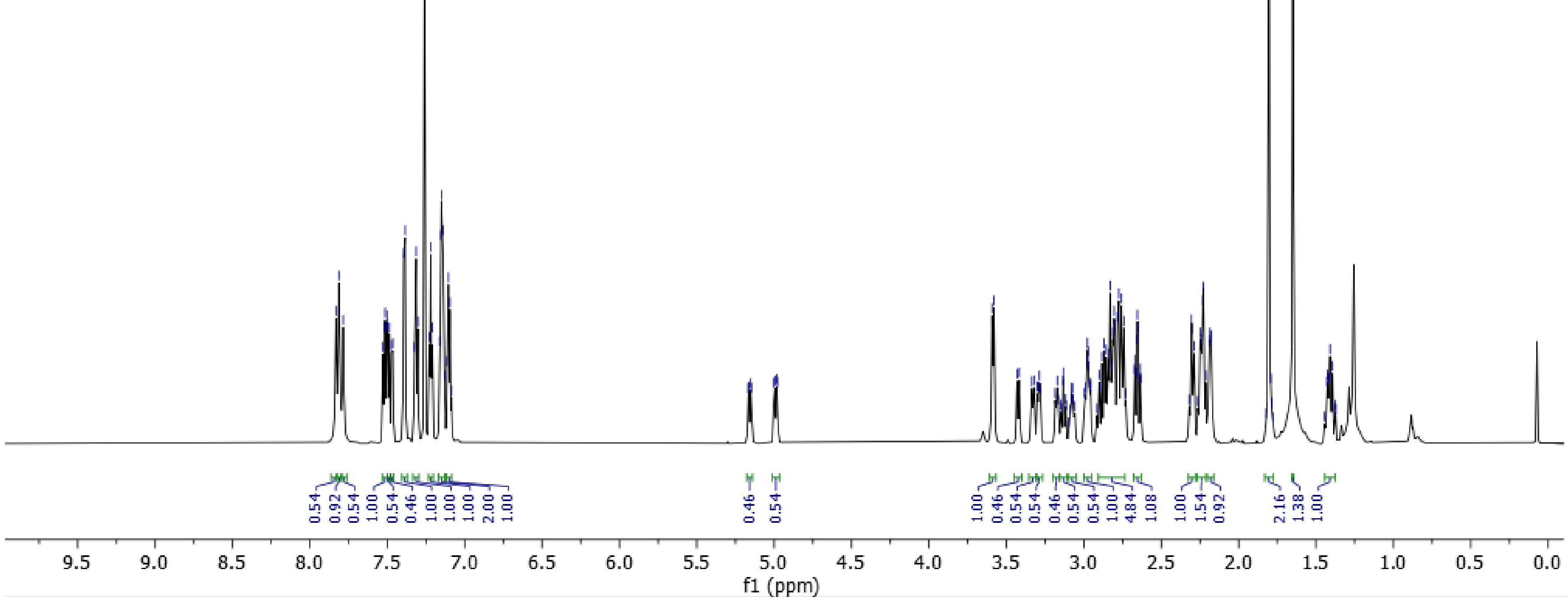

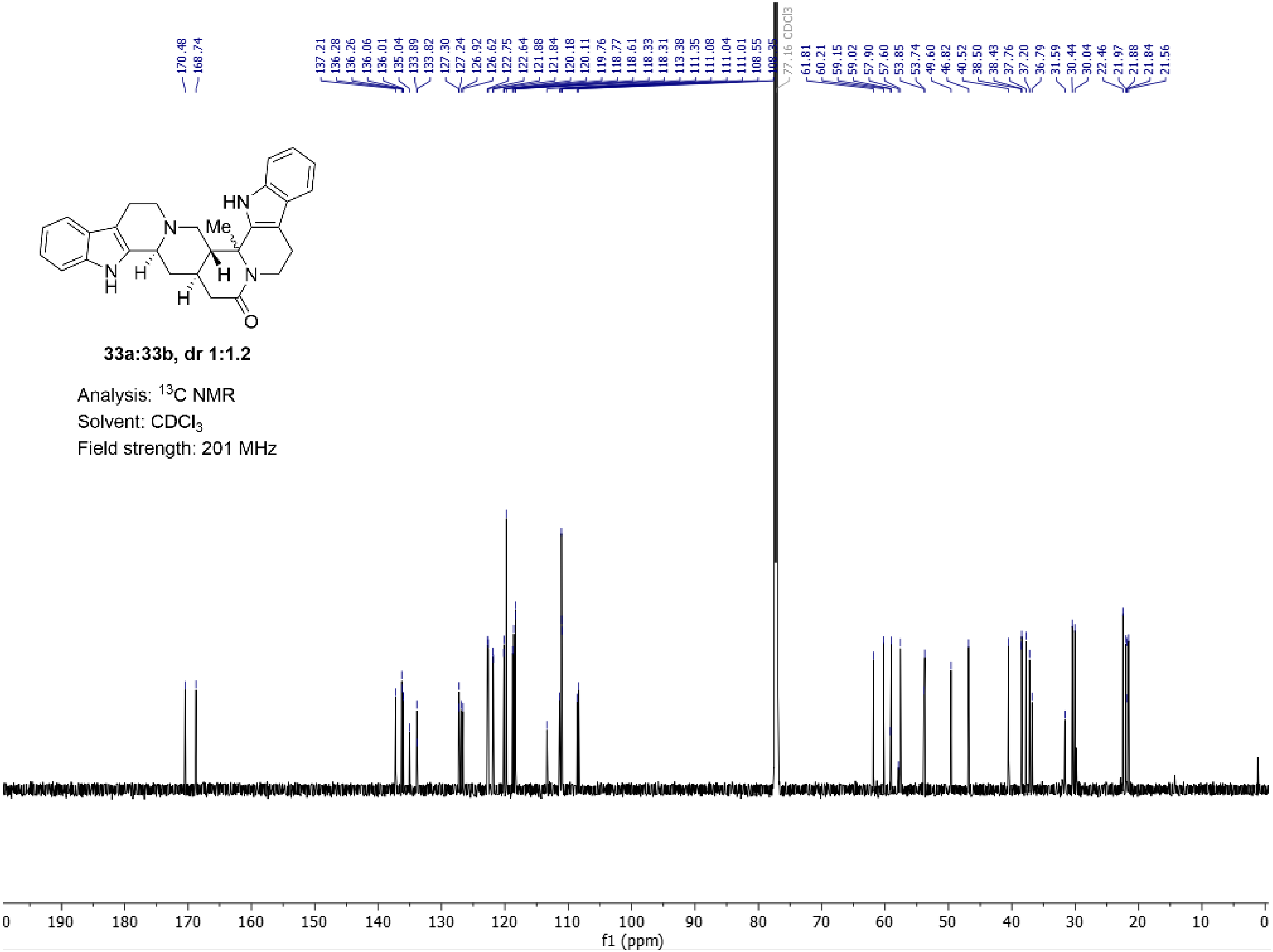

33a:33b, dr 1:1.2

Analysis: ^{13}C NMR
Solvent: $CDCl_3$
Field strength: 201 MHz

5) X-Ray crystallographic data

X-ray crystallographic data of **19a** (CCDC 2351173): **VG-2-080.**

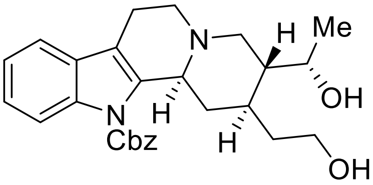

19a

R_1=3.76%

Solved by: **Farzaneh Fadaei Tirani**

Crystal Data and Experimental

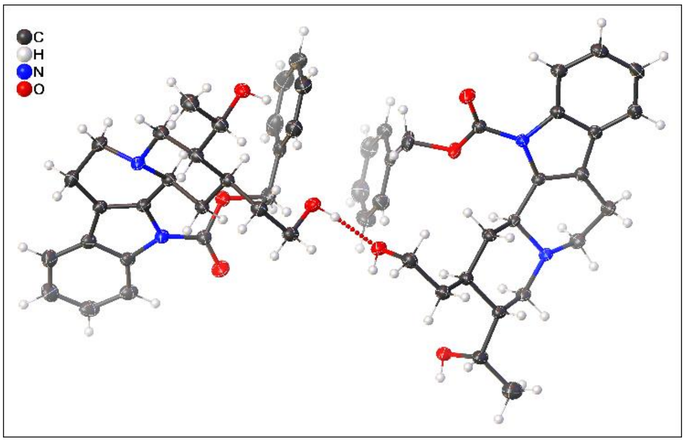

*X-Ray tructure of **19a** with a 50% ellipsoid probability level*

**Compound**	**VG-2-080**
Formula	$C_{27}H_{32}N_2O_4$
$D_{calc.}$/ g cm^{-3}	1.267
μ/mm^{-1}	0.682
Formula Weight	448.54
Colour	colourless
Shape	prism-shaped
Size/mm^3	0.13×0.06×0.02
T/K	140.00(10)
Crystal System	orthorhombic
Flack Parameter	-0.26(17)
Space Group	$P2_12_12_1$
a/Å	9.37705(8)
b/Å	10.11981(11)
c/Å	49.5764(7)
α/°	90
β/°	90
γ/°	90
V/Å^3	4704.50(10)
Z	8
Z'	2
Wavelength/Å	1.54184
Radiation type	Cu$K\alpha$
Θ_{min}/°	1.782
Θ_{max}/°	75.606
Measured Refl's.	50125
Indep't Refl's	9569
Refl's I≥2σ(I)	8476
R_{int}	0.0397
Parameters	632
Restraints	138
Largest Peak/e Å^{-3}	0.269
Deepest Hole/e Å^{-3}	-0.205
GooF	1.047
wR_2 (all data)	0.0924
wR_2	0.0892
R_1 (all data)	0.0451
R_1	0.0376
CCDC number	2351173

Experimental. Single colourless prism-shaped crystals of **vg-2-080** were used as supplied. A suitable crystal with dimensions 0.13 × 0.06 × 0.02 mm^3 was selected and mounted on an XtaLAB Synergy R, DW system, HyPix-Arc 150 diffractometer. The crystal was kept at a steady T = 140.00(10) K during data collection. The structure was solved with the ShelXT 2018/2 (Sheldrick, 2015) solution program using dual methods and by using Olex2 1.5 (Dolomanov et al., 2009) as the graphical interface. The model was refined with ShelXL 2019/3 (Sheldrick, 2015) using full matrix least squares minimisation on $\boldsymbol{F}^2$.

Crystal Data. $C_{27}H_{32}N_2O_4$, M_r = 448.54, orthorhombic, $P2_12_12_1$ (No. 19), a = 9.37705(8) Å, b = 10.11981(11) Å, c = 49.5764(7) Å, $\alpha = \beta = \gamma = 90°$, V = 4704.50(10) Å^3, T = 140.00(10) K, Z = 8, Z' = 2, μ(Cu K$_\alpha$) = 0.682, 50125 reflections measured, 9569 unique (R_{int} = 0.0397) which were used in all calculations. The final wR_2 was 0.0924 (all data) and R_1 was 0.0376 (I≥2σ(I)).

Structure Quality Indicators

**Reflections:**	d min (CuKα) 2Θ=151.2° 0.80	I/σ(I) 28.1	Rint m=5.25 3.97%	Full 135.4° 99.3	
**Refinement:**	Shift 0.001	Max Peak 0.3	Min Peak -0.2	GooF 1.047	Hooft -.26(17)

A colourless prism-shaped crystal with dimensions 0.13 × 0.06 × 0.02 mm^3 was mounted. Data were collected using an XtaLAB Synergy R, DW system, HyPix-Arc 150 diffractometer operating at T = 140.00(10) K.

Data were measured using ω scans with Cu K_α radiation. The diffraction pattern was indexed and the total number of runs and images was based on the strategy calculation from the program CrysAlisPro 1.171.42.100a (Rigaku OD, 2023). The maximum resolution achieved was Θ = 75.606° (0.80 Å).

The unit cell was refined using CrysAlisPro 1.171.42.100a (Rigaku OD, 2023) on 22447 reflections, 45% of the observed reflections.

Data reduction, scaling and absorption corrections were performed using CrysAlisPro 1.171.42.100a (Rigaku OD, 2023). The final completeness is 99.30 % out to 75.606° in Θ. A Gaussian absorption correction was performed using CrysAlisPro 1.171.43.100a (Rigaku Oxford Diffraction, 2024) Numerical absorption correction based on Gaussian integration over a multifaceted crystal model. Empirical absorption correction using spherical harmonics as implemented in SCALE3 ABSPACK scaling algorithm. The absorption coefficient μ of this material is 0.682 mm^{-1} at this wavelength (λ = 1.54184Å) and the minimum and maximum transmissions are 0.851 and 1.000.

The structure was solved in the space group $P2_12_12_1$ (# 19) by the ShelXT 2018/2 (Sheldrick, 2015) structure solution program using dual methods and refined by full matrix least squares minimisation on $\boldsymbol{F}^2$ using version 2019/3 of ShelXL 2019/3 (Sheldrick, 2015). All non-hydrogen atoms were refined anisotropically. Hydrogen atom positions were calculated geometrically and refined using the riding model.

The value of Z' is 2. This means that there are two independent molecules in the asymmetric unit. The moiety formula is C27 H32 N2 O4.

The Flack parameter was refined to -0.26(17). Determination of absolute structure using Bayesian statistics on Bijvoet differences using the Olex2 results in None. The chiral atoms in this structure are: C10(S), C13(R), C14(R), C18(S), C37(S), C40(R), C45A(S), C45B(R). Note: The Flack parameter is used to determine chirality of the crystal studied, the value should be near 0, a value of 1 means that the stereochemistry is wrong and the model should be inverted. A value of 0.5 means that the crystal consists of a racemic mixture of the two enantiomers.

Citations

CrysAlis^{Pro} Software System, Rigaku Oxford Diffraction, (2024).

Sheldrick, G.M., ShelXT-Integrated space-group and crystal-structure determination, *Acta Cryst.*, (2015), **A71**, 3-8.

Sheldrick, G.M., Crystal structure refinement with ShelXL, *Acta Cryst.*, (2015), **C71**, 3-8.

O.V. Dolomanov and L.J. Bourhis and R.J. Gildea and J.A.K. Howard and H. Puschmann, **Olex2**: A complete structure solution, refinement and analysis program, *J. Appl. Cryst.*, (2009), **42**, 339-341.

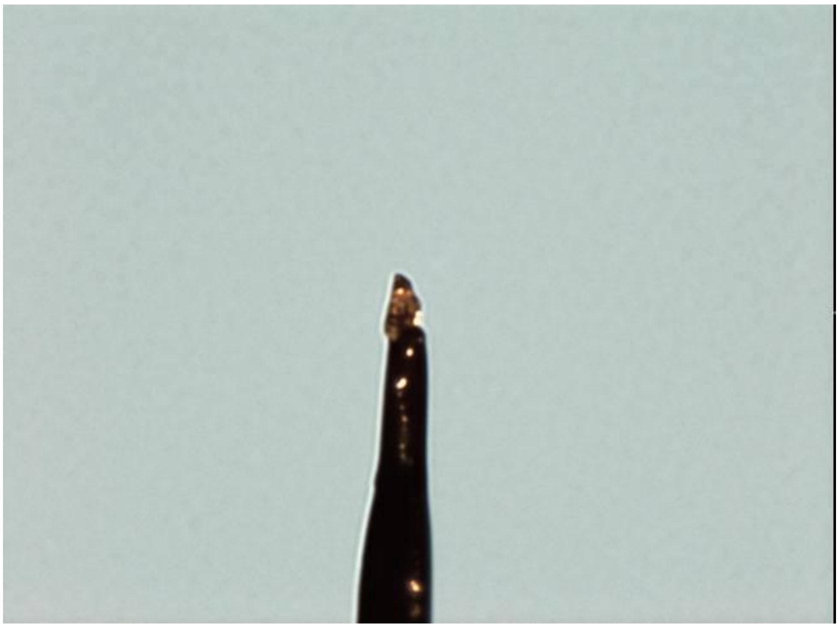

Figure 1 Image of the Crystal on the Diffractometer.

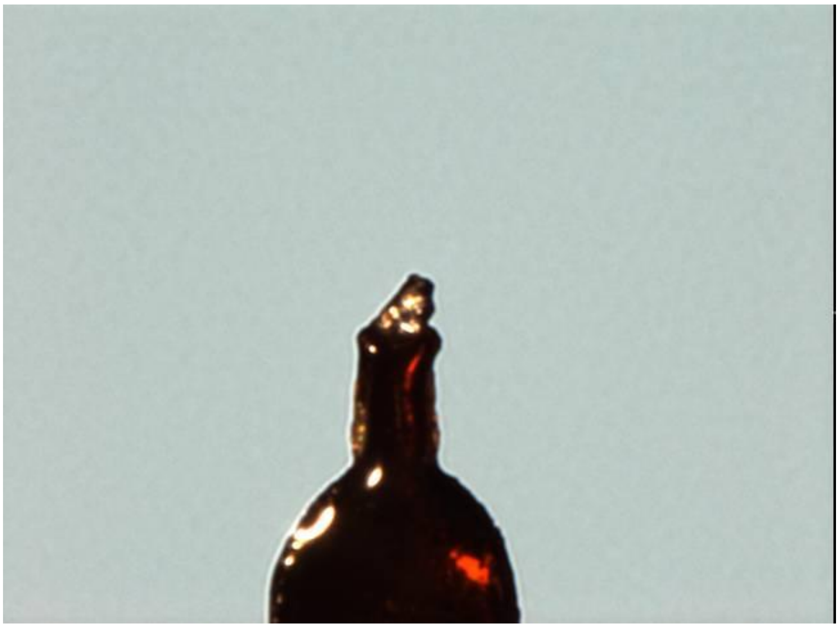

Figure 2 Image of the Crystal on the Diffractometer.

Data Plots: Diffraction Data

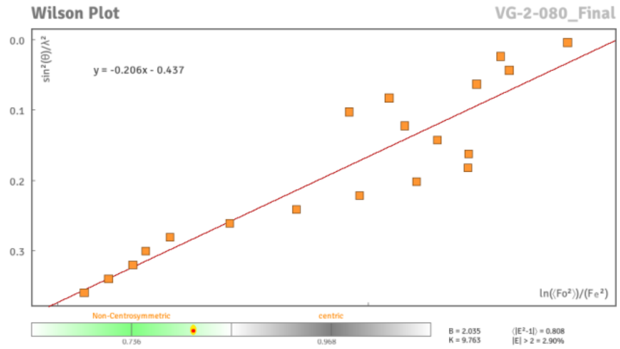

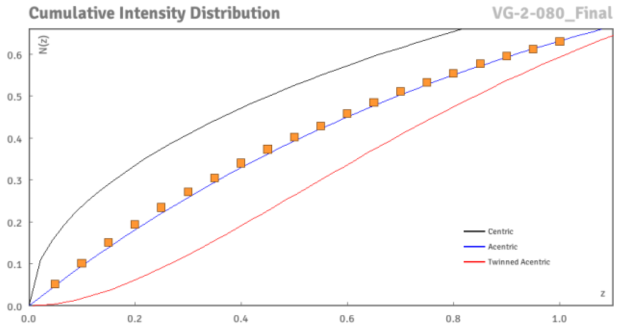

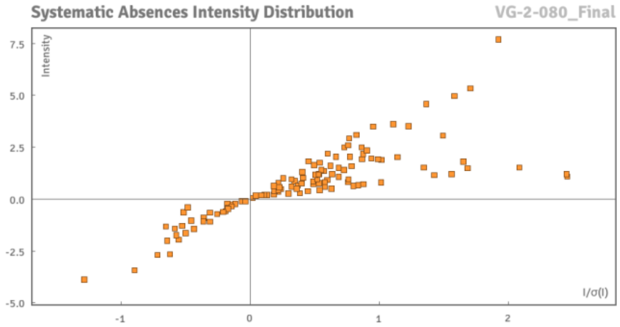

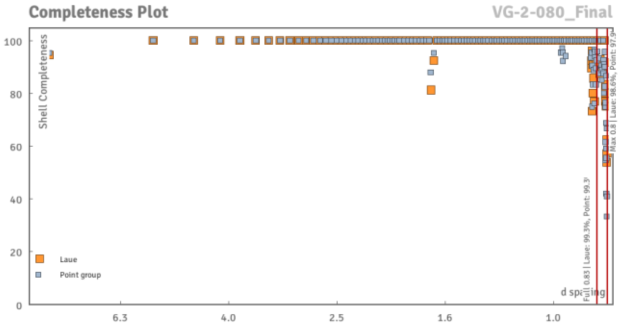

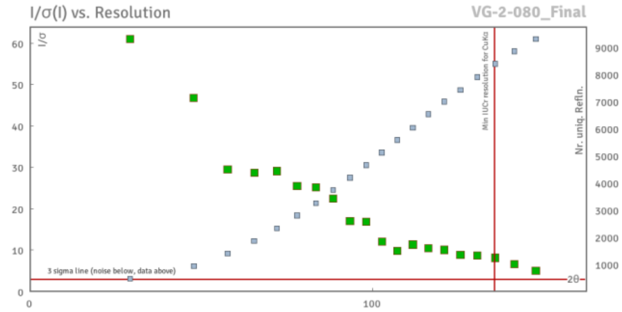

Data Plots: Refinement and Data

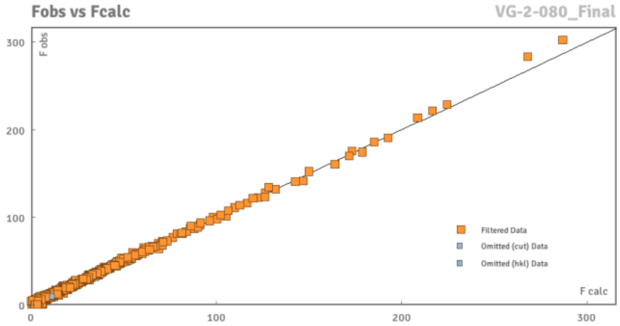

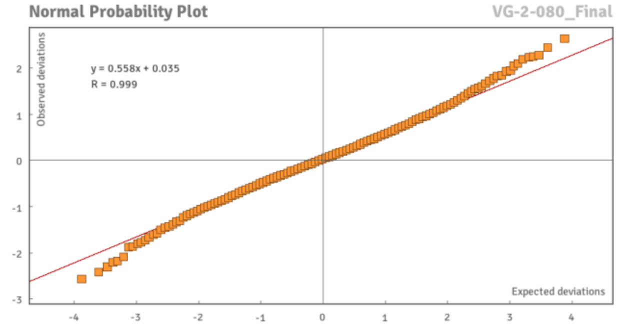

Reflection Statistics

Total reflections (after filtering)	50263	Unique reflections	9569
Completeness	0.979	Mean I/σ	19.92
hkl_{max} collected	(7, 12, 62)	hkl_{min} collected	(-11, -12, -62)
hkl_{max} used	(11, 12, 62)	hkl_{min} used	(-11, 0, 0)
Lim d_{max} collected	100.0	Lim d_{min} collected	0.77
d_{max} used	24.79	d_{min} used	0.8
Friedel pairs	4921	Friedel pairs merged	0
Inconsistent equivalents	0	R_{int}	0.0397
R_{sigma}	0.0356	Intensity transformed	0
Omitted reflections	23	Omitted by user (OMIT hkl)	0
Multiplicity	(10645, 5852, 2115, 982, 668, 500, 301, 273, 218, 201, 101, 86, 41, 15, 4, 4, 3)	Maximum multiplicity	33
Removed systematic absences	138	Filtered off (Shel/OMIT)	0

Table 1: Fractional Atomic Coordinates (×10^4) and Equivalent Isotropic Displacement Parameters (Å^2×10^3) for **vg-2-080**. U_{eq} is defined as 1/3 of the trace of the orthogonalised U_{ij}.

Atom	x	y	z	U_{eq}
O1	3360.9(18)	7338(2)	5524.0(4)	37.3(5)
O2	5432.9(17)	6237.2(18)	5577.7(4)	26.6(4)
O3	5809.1(18)	6359.1(18)	6418.4(4)	27.5(4)
O4	10738.8(18)	6422.1(18)	6330.2(4)	27.2(4)
N1	5479(2)	8233(2)	5383.9(4)	21.6(4)
N2	9209(2)	8691(2)	5638.5(4)	22.4(4)
C1	5019(3)	8975(3)	5159.1(5)	23.7(5)
C2	3660(3)	9130(3)	5052.3(5)	29.4(6)
C3	3528(3)	9930(3)	4827.5(6)	36.4(7)
C4	4715(3)	10542(3)	4711.3(6)	40.2(7)
C5	6069(3)	10352(3)	4815.7(6)	34.6(6)
C6	6237(3)	9555(3)	5042.1(5)	25.1(5)
C7	7453(3)	9157(3)	5202.6(5)	22.1(5)
C8	8994(3)	9529(3)	5176.6(5)	27.8(6)
C9	9870(3)	8689(3)	5370.7(5)	27.0(5)
C10	7850(2)	7959(2)	5644.6(5)	20.9(5)
C11	6979(2)	8391(2)	5406.0(5)	21.0(5)
C12	7138(2)	8285(2)	5912.8(5)	21.3(5)
C13	8044(2)	7838(2)	6153.2(5)	21.2(5)
C14	9575(2)	8375(3)	6127.8(5)	23.9(5)
C15	10165(2)	8141(3)	5844.4(5)	26.3(6)
C16	7340(3)	8240(3)	6420.6(5)	25.6(5)
C17	5829(3)	7757(3)	6456.3(5)	25.4(5)
C18	10567(3)	7844(3)	6349.7(6)	27.9(6)
C19	12033(3)	8445(3)	6340.0(6)	35.2(7)
C20	4633(2)	7267(3)	5501.8(5)	24.0(5)
C21	4693(3)	5197(3)	5726.5(6)	32.8(6)
C22	5725(3)	4069(3)	5746.9(5)	27.7(6)
C23	6674(3)	4006(3)	5959.3(6)	33.9(6)
C24	7660(3)	2995(3)	5975.9(7)	45.7(8)
C25	7699(4)	2034(3)	5777.1(9)	53.3(10)

Atom	x	y	z	U_{eq}
C26	6749(4)	2096(3)	5566.2(8)	52.9(10)
C27	5767(3)	3106(3)	5549.9(6)	39.6(7)
O5	5695.2(17)	733.8(19)	6826.6(4)	28.1(4)
O6	3704.5(17)	1294.4(19)	6600.0(3)	26.8(4)
O7	3201.8(18)	5444.5(18)	6571.2(4)	24.9(4)
O8A	-1662(8)	5111(12)	6582.6(16)	23.9(11)
O8B	-1460(40)	5190(60)	6575(8)	29(4)
N3	3491(2)	294(2)	7002.3(4)	19.1(4)
N4	-292.1(19)	1545(2)	7017.8(4)	20.9(4)
C28	3853(2)	-778(2)	7170.1(5)	20.0(5)
C29	5178(3)	-1334(3)	7229.9(5)	23.4(5)
C30	5192(3)	-2402(3)	7405.7(5)	28.3(6)
C31	3940(3)	-2914(3)	7516.1(5)	28.2(6)
C32	2628(3)	-2370(3)	7452.4(5)	24.8(5)
C33	2585(3)	-1294(2)	7277.9(5)	20.7(5)
C34	1431(2)	-499(2)	7173.3(5)	21.4(5)
C35	-146(3)	-647(3)	7215.5(6)	27.2(6)
C36	-905(2)	220(3)	7010.0(6)	26.0(5)
C37	1150(2)	1599(2)	6900.0(5)	19.8(5)
C38	1980(2)	452(2)	7013.9(5)	19.6(5)
C39	1784(2)	2944(2)	6972.7(5)	21.7(5)
C40	881(2)	4091(2)	6866.0(5)	21.5(5)
C41	-672(2)	3919(3)	6963.1(5)	22.9(5)
C42	-1199(2)	2539(3)	6890.4(5)	23.4(5)
C43	1522(3)	5413(3)	6949.4(5)	25.2(5)
C44	3044(3)	5643(3)	6856.0(5)	25.3(5)
C45A	-1677(6)	5039(5)	6872.6(11)	25.8(10)
C45B	-1800(40)	4830(30)	6848(6)	34(2)
C46A	-3179(3)	4890(4)	6974.5(7)	31.9(9)
C46B	-2073(17)	5937(16)	7041(3)	38(3)
C47	4423(2)	793(2)	6807.6(5)	20.3(5)
C48	4547(3)	1934(3)	6389.2(6)	33.0(6)
C49	3637(3)	1934(3)	6140.9(5)	24.5(5)
C50	3810(3)	960(3)	5946.2(6)	31.5(6)
C51	2941(3)	933(3)	5721.3(6)	39.3(7)
C52	1889(3)	1877(3)	5689.4(6)	40.4(7)
C53	1719(3)	2852(3)	5880.2(6)	38.9(7)
C54	2594(3)	2882(3)	6105.3(6)	31.3(6)

Table 2: Anisotropic Displacement Parameters (×10[4]) for **vg-2-080**. The anisotropic displacement factor exponent takes the form: $-2\pi^2[h^2a^{*2} \times U_{11}+ ... +2hka^* \times b^* \times U_{12}]$

Atom	U_{11}	U_{22}	U_{33}	U_{23}	U_{13}	U_{12}
O1	16.9(9)	45.2(12)	49.7(13)	10.0(10)	1.8(8)	-0.1(8)
O2	20.2(9)	24.1(9)	35.5(10)	5.1(8)	1.4(7)	-3.2(7)
O3	19.5(8)	27.2(9)	35.9(10)	0.5(8)	3.7(7)	-0.1(7)
O4	18.8(8)	27.9(9)	34.8(10)	5.6(8)	-2.4(7)	0.4(7)
N1	16.9(10)	25.4(11)	22.6(11)	2.3(8)	-2.1(8)	-0.3(8)
N2	14.7(9)	27.5(11)	25.1(11)	2.8(9)	-1.1(8)	-1.0(8)
C1	24.4(12)	26.0(12)	20.8(12)	-0.5(10)	0.2(9)	4.2(10)
C2	25.6(13)	36.8(15)	25.8(13)	-3.1(11)	-2.4(10)	6.2(12)
C3	31.1(15)	49.6(18)	28.5(14)	2.0(13)	-6.5(12)	11.8(13)
C4	43.4(17)	48.3(19)	28.8(15)	12.7(14)	-4.0(12)	8.6(15)
C5	32.9(14)	41.9(16)	28.9(14)	7.9(13)	2.8(11)	2.4(13)
C6	26.3(12)	28.5(13)	20.5(12)	-1.1(10)	0.6(10)	2.5(11)
C7	21.3(12)	24.5(12)	20.7(12)	-1.1(10)	0.8(9)	1.2(10)

Atom	U_{11}	U_{22}	U_{33}	U_{23}	U_{13}	U_{12}
C8	25.0(13)	33.9(14)	24.5(13)	1.6(11)	4.5(10)	-3.1(11)
C9	18.3(12)	32.6(14)	30.0(14)	-1.5(11)	4.4(10)	-1.2(11)
C10	15.9(11)	21.5(12)	25.3(13)	0.8(10)	-0.9(9)	-0.1(9)
C11	16.9(11)	21.8(12)	24.3(12)	-2.5(10)	1.1(9)	0.6(9)
C12	16.4(11)	22.3(12)	25.2(13)	1.4(10)	-1.4(9)	-0.8(9)
C13	17.4(11)	20.2(11)	26.0(13)	1.5(10)	-3.5(9)	-1.6(9)
C14	19.1(12)	22.0(12)	30.7(14)	2.6(10)	-5.5(10)	-2.5(10)
C15	14.4(11)	29.1(13)	35.3(15)	4.3(11)	-3.0(10)	0.0(10)
C16	25.6(13)	25.3(13)	25.9(13)	-0.8(11)	-4.9(10)	0.1(10)
C17	24.6(13)	27.9(13)	23.8(12)	-0.6(11)	-0.9(10)	5.0(11)
C18	24.4(13)	27.1(13)	32.1(14)	2.2(11)	-7.5(10)	-0.2(11)
C19	24.8(14)	35.7(16)	45.0(17)	6.1(14)	-11.2(12)	-4.1(12)
C20	20.0(12)	26.6(13)	25.4(13)	1.7(11)	-1.8(9)	-0.2(10)
C21	27.4(13)	28.1(14)	43.0(17)	7.9(13)	6.0(12)	-5.8(11)
C22	29.7(13)	23.1(12)	30.2(14)	4.0(11)	6.8(11)	-5.7(11)
C23	34.3(14)	33.5(15)	33.9(15)	0.9(12)	4.3(12)	-0.6(12)
C24	35.2(16)	47.3(19)	55(2)	17.8(17)	6.4(14)	6.0(15)
C25	45.9(19)	27.2(16)	87(3)	12.3(18)	31.8(19)	4.4(15)
C26	60(2)	29.5(16)	69(3)	-14.8(17)	31(2)	-8.3(16)
C27	46.1(17)	33.7(16)	38.8(17)	-6.4(13)	10.0(14)	-13.6(14)
O5	14.6(8)	37.7(10)	31.9(10)	5.3(9)	-0.9(7)	-0.9(7)
O6	17.6(8)	38.4(10)	24.3(9)	11.0(8)	0.0(7)	-0.7(8)
O7	18.8(8)	28.4(9)	27.6(9)	0.8(8)	0.1(7)	1.4(7)
O8A	14(2)	31(2)	26.7(16)	7.9(14)	0.4(14)	4.1(19)
O8B	26(7)	32(6)	29(6)	8(5)	0(6)	7(7)
N3	14.8(9)	20.4(10)	22.1(10)	1.8(8)	-0.2(7)	-0.3(8)
N4	11.8(9)	23.9(10)	26.9(11)	3.4(9)	0.4(8)	-0.5(8)
C28	20.6(11)	20.1(11)	19.3(11)	-1.9(9)	-3.1(9)	1.3(10)
C29	20.4(12)	22.1(12)	27.8(13)	-2.2(10)	-3.1(10)	1.3(10)
C30	28.3(13)	26.3(13)	30.5(14)	0.3(11)	-6.5(10)	6.9(11)
C31	38.2(15)	21.4(12)	24.9(13)	3.5(11)	-2.1(11)	5.6(11)
C32	28.4(13)	22.7(12)	23.3(12)	0.3(10)	1.7(10)	0.1(10)
C33	23.7(12)	17.5(11)	20.8(12)	-2.9(9)	0.0(9)	0.5(10)
C34	19.1(12)	22.1(12)	23.1(12)	-0.8(10)	-1.0(9)	-1.2(10)
C35	18.5(12)	25.8(13)	37.3(15)	4.4(11)	3.1(10)	-2.8(10)
C36	15.4(11)	28.1(13)	34.5(14)	1.2(11)	-1.5(10)	-3.5(10)
C37	13.9(10)	23.5(12)	22.0(11)	2.3(10)	0.9(9)	-0.7(9)
C38	13.5(11)	23.0(12)	22.2(12)	0.0(10)	-1.1(9)	-0.5(9)
C39	15.5(11)	22.7(12)	27.0(13)	3.3(10)	-2.6(9)	0.0(10)
C40	18.6(11)	22.9(12)	23.0(12)	1.8(10)	-0.1(9)	1.3(10)
C41	16.7(11)	28.6(13)	23.5(12)	2.3(10)	0.3(9)	4.3(10)
C42	14.0(11)	29.8(13)	26.3(12)	2.4(11)	-2.5(9)	1.9(10)
C43	24.4(13)	23.3(12)	27.9(13)	0.8(11)	0.8(10)	0.7(10)
C44	25.2(13)	22.3(12)	28.4(13)	0.0(11)	-2.6(10)	-2.1(10)
C45A	23.5(18)	28(2)	25.7(17)	3.1(15)	-0.1(13)	6.5(17)
C45B	32(3)	36(4)	34(3)	9(3)	10(3)	12(3)
C46A	25.5(16)	35.6(18)	34.7(18)	6.5(15)	3.6(13)	10.2(14)
C46B	40(5)	37(5)	37(5)	14(4)	20(4)	23(4)
C47	16.5(12)	20.8(11)	23.5(12)	-0.2(10)	-1.2(9)	-1.2(9)
C48	23.2(13)	46.6(17)	29.1(15)	14.1(13)	2.0(11)	-8.7(12)
C49	20.4(12)	29.0(13)	24.0(13)	7.3(11)	4.8(10)	-3.5(10)
C50	30.3(13)	27.9(14)	36.3(15)	2.8(12)	9.0(12)	1.1(12)
C51	48.5(18)	37.9(16)	31.4(16)	-7.3(13)	6.7(13)	-9.7(14)
C52	40.4(16)	50.7(19)	30.1(16)	4.9(14)	-7.6(13)	-10.2(15)
C53	31.5(14)	43.5(17)	41.7(17)	8.0(14)	-7.1(13)	6.9(13)
C54	34.0(14)	31.5(14)	28.5(14)	-2.0(12)	2.2(11)	3.2(12)

Table 3: Bond Lengths in Å for **vg-2-080.**

Atom	Atom	Length/Å
O1	C20	1.200(3)
O2	C20	1.338(3)
O2	C21	1.460(3)
O3	C17	1.427(3)
O4	C18	1.451(3)
N1	C1	1.411(3)
N1	C11	1.420(3)
N1	C20	1.388(3)
N2	C9	1.465(3)
N2	C10	1.475(3)
N2	C15	1.468(3)
C1	C2	1.388(4)
C1	C6	1.409(4)
C2	C3	1.383(4)
C3	C4	1.398(4)
C4	C5	1.384(4)
C5	C6	1.391(4)
C6	C7	1.448(3)
C7	C8	1.499(3)
C7	C11	1.347(4)
C8	C9	1.524(4)
C10	C11	1.502(3)
C10	C12	1.524(3)
C12	C13	1.531(3)
C13	C14	1.541(3)
C13	C16	1.536(3)
C14	C15	1.528(4)
C14	C18	1.538(3)
C16	C17	1.509(3)
C18	C19	1.504(4)
C21	C22	1.500(4)
C22	C23	1.380(4)
C22	C27	1.381(4)
C23	C24	1.382(4)
C24	C25	1.385(5)
C25	C26	1.375(6)
C26	C27	1.378(5)
O5	C47	1.198(3)
O6	C47	1.331(3)
O6	C48	1.461(3)
O7	C44	1.434(3)
O8A	C45A	1.439(6)
O8B	C45B	1.44(2)
N3	C28	1.408(3)
N3	C38	1.427(3)
N3	C47	1.396(3)
N4	C36	1.459(3)
N4	C37	1.474(3)
N4	C42	1.461(3)
C28	C29	1.397(3)
C28	C33	1.405(3)
C29	C30	1.389(4)
C30	C31	1.395(4)
C31	C32	1.384(4)
C32	C33	1.391(4)
C33	C34	1.445(3)
C34	C35	1.501(3)
C34	C38	1.348(3)
C35	C36	1.521(4)
C37	C38	1.507(3)
C37	C39	1.528(3)
C39	C40	1.532(3)
C40	C41	1.543(3)
C40	C43	1.523(3)
C41	C42	1.525(4)
C41	C45A	1.541(5)
C41	C45B	1.52(2)
C43	C44	1.519(3)
C45A	C46A	1.503(7)
C45B	C46B	1.49(2)
C48	C49	1.497(4)
C49	C50	1.389(4)
C49	C54	1.381(4)
C50	C51	1.381(4)
C51	C52	1.383(5)
C52	C53	1.376(5)
C53	C54	1.386(4)

Table 4: Bond Angles in ° for **vg-2-080**.

Atom	Atom	Atom	Angle/°
C20	O2	C21	115.91(19)
C1	N1	C11	107.7(2)
C20	N1	C1	122.2(2)
C20	N1	C11	127.8(2)
C9	N2	C10	112.5(2)
C9	N2	C15	111.82(19)
C15	N2	C10	108.88(19)
C2	C1	N1	129.9(2)
C2	C1	C6	122.7(2)
C6	C1	N1	107.4(2)
C3	C2	C1	117.1(3)
C2	C3	C4	121.3(3)
C5	C4	C3	121.0(3)
C4	C5	C6	119.1(3)

Atom	Atom	Atom	Angle/˚
C1	C6	C7	107.2(2)
C5	C6	C1	118.8(2)
C5	C6	C7	133.9(2)
C6	C7	C8	130.0(2)
C11	C7	C6	108.2(2)
C11	C7	C8	121.8(2)
C7	C8	C9	109.0(2)
N2	C9	C8	110.1(2)
N2	C10	C11	107.9(2)
N2	C10	C12	106.73(19)
C11	C10	C12	112.71(19)
N1	C11	C10	124.5(2)
C7	C11	N1	109.5(2)
C7	C11	C10	125.2(2)
C10	C12	C13	111.87(19)
C12	C13	C14	110.4(2)
C12	C13	C16	110.82(19)
C16	C13	C14	112.2(2)
C15	C14	C13	111.0(2)
C15	C14	C18	112.6(2)
C18	C14	C13	112.5(2)
N2	C15	C14	111.1(2)
C17	C16	C13	114.8(2)
O3	C17	C16	108.5(2)
O4	C18	C14	111.5(2)
O4	C18	C19	107.3(2)
C19	C18	C14	112.9(2)
O1	C20	O2	125.3(2)
O1	C20	N1	124.3(2)
O2	C20	N1	110.3(2)
O2	C21	C22	106.0(2)
C23	C22	C21	120.2(3)
C27	C22	C21	120.5(3)
C27	C22	C23	119.3(3)
C22	C23	C24	120.8(3)
C23	C24	C25	119.7(3)
C26	C25	C24	119.5(3)
C25	C26	C27	120.8(3)
C26	C27	C22	120.1(3)
C47	O6	C48	116.66(18)
C28	N3	C38	107.56(19)
C47	N3	C28	122.40(19)
C47	N3	C38	127.5(2)
C36	N4	C37	112.6(2)
C36	N4	C42	113.08(19)
C42	N4	C37	109.72(19)
C29	C28	N3	130.6(2)
C29	C28	C33	121.5(2)
C33	C28	N3	107.9(2)
C30	C29	C28	117.1(2)
C29	C30	C31	121.8(2)
C32	C31	C30	120.7(2)
C31	C32	C33	118.6(2)
C28	C33	C34	106.9(2)
C32	C33	C28	120.2(2)
C32	C33	C34	132.9(2)
C33	C34	C35	129.2(2)
C38	C34	C33	108.8(2)
C38	C34	C35	122.0(2)
C34	C35	C36	108.0(2)
N4	C36	C35	109.2(2)
N4	C37	C38	107.25(19)
N4	C37	C39	107.3(2)
C38	C37	C39	113.36(19)
N3	C38	C37	125.7(2)
C34	C38	N3	108.9(2)
C34	C38	C37	124.9(2)
C37	C39	C40	112.25(19)
C39	C40	C41	109.2(2)
C43	C40	C39	110.7(2)
C43	C40	C41	112.7(2)
C42	C41	C40	109.6(2)
C42	C41	C45A	114.0(3)
C45A	C41	C40	113.8(3)
C45B	C41	C40	118.3(16)
C45B	C41	C42	104.0(14)
N4	C42	C41	109.85(19)
C44	C43	C40	115.0(2)
O7	C44	C43	112.0(2)
O8A	C45A	C41	108.8(6)
O8A	C45A	C46A	110.5(5)
C46A	C45A	C41	113.7(4)
O8B	C45B	C41	110(3)
O8B	C45B	C46B	117(4)
C46B	C45B	C41	109.5(17)
O5	C47	O6	125.7(2)
O5	C47	N3	123.4(2)
O6	C47	N3	110.84(18)
O6	C48	C49	106.28(19)
C50	C49	C48	120.3(3)
C54	C49	C48	120.6(3)
C54	C49	C50	119.1(3)
C51	C50	C49	120.4(3)
C50	C51	C52	120.0(3)
C53	C52	C51	120.0(3)
C52	C53	C54	120.0(3)
C49	C54	C53	120.5(3)

Table 5: Torsion Angles in ˚ for **vg-2-080**.

Atom	Atom	Atom	Atom	Angle/˚
O2	C21	C22	C23	88.2(3)
O2	C21	C22	C27	-89.8(3)
N1	C1	C2	C3	179.9(3)
N1	C1	C6	C5	-179.6(2)
N1	C1	C6	C7	-0.4(3)
N2	C10	C11	N1	-160.1(2)

Atom	Atom	Atom	Atom	Angle/°
N2	C10	C11	C7	9.1(3)
N2	C10	C12	C13	-61.6(3)
C1	N1	C11	C7	-1.7(3)
C1	N1	C11	C10	168.9(2)
C1	N1	C20	O1	-36.2(4)
C1	N1	C20	O2	141.7(2)
C1	C2	C3	C4	0.8(5)
C1	C6	C7	C8	-177.2(3)
C1	C6	C7	C11	-0.6(3)
C2	C1	C6	C5	2.2(4)
C2	C1	C6	C7	-178.6(3)
C2	C3	C4	C5	0.8(5)
C3	C4	C5	C6	-0.9(5)
C4	C5	C6	C1	-0.5(4)
C4	C5	C6	C7	-179.4(3)
C5	C6	C7	C8	1.7(5)
C5	C6	C7	C11	178.3(3)
C6	C1	C2	C3	-2.3(4)
C6	C7	C8	C9	-172.1(3)
C6	C7	C11	N1	1.4(3)
C6	C7	C11	C10	-169.1(2)
C7	C8	C9	N2	-47.7(3)
C8	C7	C11	N1	178.4(2)
C8	C7	C11	C10	7.8(4)
C9	N2	C10	C11	-46.8(3)
C9	N2	C10	C12	-168.2(2)
C9	N2	C15	C14	169.8(2)
C10	N2	C9	C8	69.4(3)
C10	N2	C15	C14	-65.2(3)
C10	C12	C13	C14	52.2(3)
C10	C12	C13	C16	177.1(2)
C11	N1	C1	C2	179.3(3)
C11	N1	C1	C6	1.3(3)
C11	N1	C20	O1	163.3(3)
C11	N1	C20	O2	-18.7(4)
C11	C7	C8	C9	11.7(4)
C11	C10	C12	C13	-180.0(2)
C12	C10	C11	N1	-42.4(3)
C12	C10	C11	C7	126.7(3)
C12	C13	C14	C15	-47.2(3)
C12	C13	C14	C18	-174.5(2)
C12	C13	C16	C17	55.4(3)
C13	C14	C15	N2	54.4(3)
C13	C14	C18	O4	63.7(3)
C13	C14	C18	C19	-175.4(2)
C13	C16	C17	O3	54.2(3)
C14	C13	C16	C17	179.3(2)
C15	N2	C9	C8	-167.7(2)
C15	N2	C10	C11	-171.4(2)
C15	N2	C10	C12	67.2(2)
C15	C14	C18	O4	-62.6(3)
C15	C14	C18	C19	58.3(3)
C16	C13	C14	C15	-171.4(2)
C16	C13	C14	C18	61.4(3)
C18	C14	C15	N2	-178.5(2)
C20	O2	C21	C22	170.6(2)
C20	N1	C1	C2	15.4(4)
C20	N1	C1	C6	-162.6(2)
C20	N1	C11	C7	161.0(2)
C20	N1	C11	C10	-28.4(4)
C21	O2	C20	O1	-7.3(4)
C21	O2	C20	N1	174.8(2)
C21	C22	C23	C24	-177.9(3)
C21	C22	C27	C26	177.9(3)
C22	C23	C24	C25	0.1(5)
C23	C22	C27	C26	0.0(4)
C23	C24	C25	C26	-0.3(5)
C24	C25	C26	C27	0.3(5)
C25	C26	C27	C22	-0.2(5)
C27	C22	C23	C24	0.1(4)
O6	C48	C49	C50	-97.2(3)
O6	C48	C49	C54	81.2(3)
N3	C28	C29	C30	179.9(2)
N3	C28	C33	C32	-180.0(2)
N3	C28	C33	C34	-0.5(3)
N4	C37	C38	N3	-165.2(2)
N4	C37	C38	C34	5.7(3)
N4	C37	C39	C40	-59.0(3)
C28	N3	C38	C34	-1.9(3)
C28	N3	C38	C37	170.1(2)
C28	N3	C47	O5	-28.4(4)
C28	N3	C47	O6	149.8(2)
C28	C29	C30	C31	0.8(4)
C28	C33	C34	C35	177.6(3)
C28	C33	C34	C38	-0.7(3)
C29	C28	C33	C32	1.2(4)
C29	C28	C33	C34	-179.4(2)
C29	C30	C31	C32	0.2(4)
C30	C31	C32	C33	-0.6(4)
C31	C32	C33	C28	-0.1(4)
C31	C32	C33	C34	-179.3(3)
C32	C33	C34	C35	-3.1(5)
C32	C33	C34	C38	178.7(3)
C33	C28	C29	C30	-1.5(4)
C33	C34	C35	C36	-166.4(2)
C33	C34	C38	N3	1.6(3)
C33	C34	C38	C37	-170.6(2)
C34	C35	C36	N4	-50.0(3)
C35	C34	C38	N3	-176.8(2)
C35	C34	C38	C37	11.1(4)
C36	N4	C37	C38	-46.3(3)
C36	N4	C37	C39	-168.4(2)
C36	N4	C42	C41	166.8(2)
C37	N4	C36	C35	72.1(3)
C37	N4	C42	C41	-66.5(3)
C37	C39	C40	C41	53.6(3)
C37	C39	C40	C43	178.3(2)
C38	N3	C28	C29	-179.8(2)
C38	N3	C28	C33	1.5(3)
C38	N3	C47	O5	172.0(2)
C38	N3	C47	O6	-9.8(3)
C38	C34	C35	C36	11.6(4)
C38	C37	C39	C40	-177.2(2)
C39	C37	C38	N3	-47.0(3)

Atom	Atom	Atom	Atom	Angle/°
C39	C37	C38	C34	123.9(3)
C39	C40	C41	C42	-52.3(3)
C39	C40	C41	C45A	178.7(3)
C39	C40	C41	C45B	-171.2(12)
C39	C40	C43	C44	58.1(3)
C40	C41	C42	N4	59.4(3)
C40	C41	C45A	O8A	58.2(6)
C40	C41	C45A	C46A	-178.2(3)
C40	C41	C45B	O8B	32(4)
C40	C41	C45B	C46B	-98(2)
C40	C43	C44	O7	52.7(3)
C41	C40	C43	C44	-179.3(2)
C42	N4	C36	C35	-162.8(2)
C42	N4	C37	C38	-173.2(2)
C42	N4	C37	C39	64.7(2)
C42	C41	C45A	O8A	-68.5(6)
C42	C41	C45A	C46A	55.1(4)
C42	C41	C45B	O8B	-90(4)
C42	C41	C45B	C46B	140(2)
C43	C40	C41	C42	-175.8(2)
C43	C40	C41	C45A	55.3(3)
C43	C40	C41	C45B	65.3(12)
C45A	C41	C42	N4	-171.8(3)

Atom	Atom	Atom	Atom	Angle/°
C45B	C41	C42	N4	-173.2(13)
C47	O6	C48	C49	158.6(2)
C47	N3	C28	C29	17.1(4)
C47	N3	C28	C33	-161.6(2)
C47	N3	C38	C34	160.1(2)
C47	N3	C38	C37	-27.8(4)
C48	O6	C47	O5	-5.9(4)
C48	O6	C47	N3	175.9(2)
C48	C49	C50	C51	177.9(2)
C48	C49	C54	C53	-177.6(3)
C49	C50	C51	C52	-0.2(4)
C50	C49	C54	C53	0.8(4)
C50	C51	C52	C53	0.7(5)
C51	C52	C53	C54	-0.4(5)
C52	C53	C54	C49	-0.3(4)
C54	C49	C50	C51	-0.6(4)

Table 6: Hydrogen Fractional Atomic Coordinates (×10⁴) and Equivalent Isotropic Displacement Parameters (Å²×10³) for **vg-2-080**. U_{eq} is defined as 1/3 of the trace of the orthogonalised U_{ij}.

Atom	x	y	z	U_{eq}
H3	5027.5	6053.9	6472.95	41
H4	10039.02	6046.41	6403.07	41
H2	2856.7	8704.53	5130.17	35
H3A	2611.97	10067.37	4750.54	44
H4A	4591.4	11095.37	4558.31	48
H5	6871.69	10761.27	4733.96	41
H8C	9119.91	10476.81	5219.45	33
H8D	9320.11	9377.67	4989.18	33
H9A	9929.34	7772.1	5302.19	32
H9B	10850.31	9046.82	5383.11	32
H10	8039.41	6988.51	5633.37	25
H12A	6196.32	7845.03	5921.07	26
H12B	6980.04	9250.54	5924.37	26
H13	8095.63	6851.25	6148.68	25
H14	9517.51	9353.45	6153.21	29
H15A	11115.82	8560.08	5828.61	32
H15B	10281.28	7180.55	5813.76	32
H16A	7925.9	7894.88	6571.06	31
H16B	7343.58	9216.24	6433.5	31
H17A	5482.2	7979.78	6639.39	31
H17B	5195.58	8188.99	6322.98	31
H18	10132.57	8053.42	6528.91	33
H19A	11952.06	9409.71	6331.83	53
H19B	12537.44	8124.99	6179.61	53
H19C	12565.41	8192.28	6502.08	53
H21A	3820.65	4920.65	5629.28	39
H21B	4420.08	5512.84	5908.29	39

Atom	x	y	z	U_{eq}
H23	6649.63	4665.85	6095.56	41
H24	8308.86	2959.29	6122.97	55
H25	8375.68	1337.24	5786.41	64
H26	6770.69	1435.64	5430.12	63
H27	5117.86	3139.48	5402.9	47
H7	2481.43	5742.57	6490.92	37
H8A	-2457.1	5382.12	6527.45	36
H8B	-2051.58	5746.04	6518.61	43
H29	6033.46	-997.02	7153.58	28
H30	6078.16	-2795.6	7452.2	34
H31	3987.77	-3643.97	7636.28	34
H32	1774.73	-2724.13	7526.33	30
H35A	-406.24	-371.39	7400.7	33
H35B	-430.59	-1581.55	7191.13	33
H36A	-793.11	-160	6827.25	31
H36B	-1935.61	261.98	7052.29	31
H37	1086.3	1512.79	6699.45	24
H39A	2757.62	3008.53	6896.59	26
H39B	1860.84	3015.44	7171.29	26
H40	883.84	4047.06	6664.46	26
H41	-641.49	3956.75	7164.57	28
H41A	-684.75	4005.94	7164	28
H42A	-2195.8	2427.88	6952.43	28
H42B	-1180.17	2423.5	6692.16	28
H43A	914.17	6129.48	6876.79	30
H43B	1495.13	5475.73	7148.6	30
H44A	3330.91	6557.47	6901.84	30
H44B	3687.99	5031.22	6953.25	30
H45A	-1289.46	5889.6	6944.18	31
H45B	-2704.61	4304	6839.36	41
H46A	-3719.68	5694.73	6934.25	48
H46B	-3162.99	4745.08	7169.91	48
H46C	-3631.77	4133.43	6885.74	48
H46D	-1191.02	6440.43	7067.83	57
H46E	-2392.04	5576.29	7214.39	57
H46F	-2811.42	6519.99	6967.82	57
H48A	5441.48	1439.19	6356.9	40
H48B	4791.02	2849.41	6442.16	40
H50	4530.07	307.99	5967.92	38
H51	3066.31	265.19	5588.66	47
H52	1283.88	1853.42	5535.72	49
H53	1000.22	3504.4	5857.7	47
H54	2476.37	3558.9	6236.04	38

Table 7: Hydrogen Bond information for **vg-2-080**.

D	H	A	d(D-H)/Å	d(H-A)/Å	d(D-A)/Å	D-H-A/deg
O3	H3	O7	0.84	1.88	2.722(2)	175.2
O4	H4	O8A[1]	0.84	2.06	2.898(7)	179.4
O4	H4	O8B[1]	0.84	1.86	2.70(4)	177.6
C2	H2	O1	0.95	2.44	2.973(4)	115.4
C12	H12A	O2	0.99	2.46	3.100(3)	121.9
O7	H7	O4[2]	0.84	1.94	2.782(2)	175.8
O8A	H8A	O3[2]	0.84	1.98	2.807(9)	169.0
O8B	H8B	O3[2]	0.84	2.16	2.93(5)	152.5
C29	H29	O5	0.95	2.41	2.935(3)	114.7

D	H	A	d(D-H)/Å	d(H-A)/Å	d(D-A)/Å	D-H-A/deg
C36	H36B	O5[2]	0.99	2.53	3.356(3)	140.4
C39	H39A	O6	0.99	2.44	3.073(3)	121.2

– – – –

[1]1+x,+y,+z; [2]-1+x,+y,+z

Table 8: Atomic Occupancies for all atoms that are not fully occupied in **vg-2-080**.

Atom	Occupancy
O8A	0.818(6)
H8A	0.818(6)
O8B	0.182(6)
H8B	0.182(6)
H41	0.818(6)
H41A	0.182(6)
C45A	0.818(6)
H45A	0.818(6)
C45B	0.182(6)
H45B	0.182(6)
C46A	0.818(6)
H46A	0.818(6)
H46B	0.818(6)
H46C	0.818(6)
C46B	0.182(6)
H46D	0.182(6)
H46E	0.182(6)
H46F	0.182(6)

X-ray crystallographic data of **25** (CCDC 2351174): **VG-2-122.**

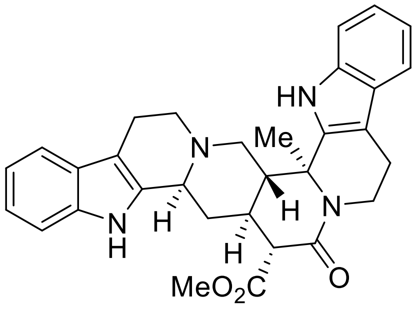

25

R₁=5.00%

Solved by: **Farzaneh Fadaei Tirani**

Crystal Data and Experimental

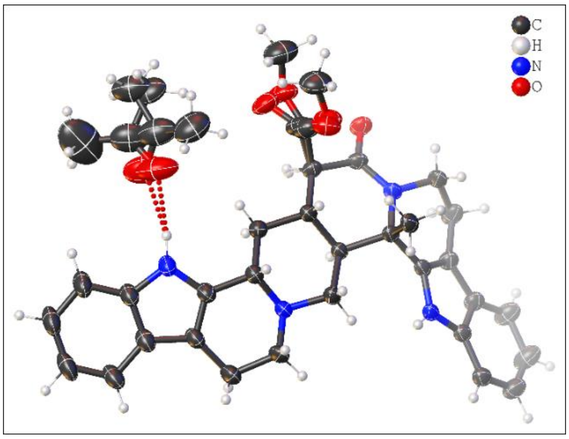

*X-Ray tructure of **25** with a 50% ellipsoid probability level*

Experimental. Single colourless block-shaped crystals of **vg-2-122** were used as supplied. A suitable crystal with dimensions 0.14 × 0.07 × 0.04 mm³ was selected and mounted on a XtaLAB Synergy R, DW system, HyPix-Arc 150 diffractometer. The crystal was kept at a steady T = 139.99(10) K during data collection. The structure was solved with the ShelXT 2018/2 (Sheldrick, 2015) solution program using dual methods and by using Olex2 1.5 (Dolomanov et al., 2009) as the graphical interface. The model was refined with ShelXL 2019/3 (Sheldrick, 2015) using full matrix least squares minimisation on F^2.

Crystal Data. $C_{37}H_{44}N_4O_5$, M_r = 624.76, monoclinic, $P2_1$ (No. 4), a = 11.06938(19) Å, b = 8.15997(12) Å, c = 18.1993(3) Å, β = 92.9559(15)°, α = γ = 90°, V = 1641.68(4) Å³, T = 139.99(10) K, Z = 2, Z' = 1, μ(Cu K_α) = 0.678, 26888 reflections measured, 6453 unique (R_{int} = 0.0236) which were used in all calculations. The final wR_2 was 0.1442 (all data) and R_1 was 0.0500 (I≥2σ(I)).

**Compound**	**VG-2-122**
Formula	$C_{37}H_{44}N_4O_5$
$D_{calc.}$/ g cm^{-3}	1.264
μ/mm^{-1}	0.678
Formula Weight	624.76
Colour	colourless
Shape	block-shaped
Size/mm³	0.14×0.07×0.04
T/K	139.99(10)
Crystal System	monoclinic
Flack Parameter	0.6(3)
Space Group	$P2_1$
a/Å	11.06938(19)
b/Å	8.15997(12)
c/Å	18.1993(3)
α/°	90
β/°	92.9559(15)
γ/°	90
V/Å³	1641.68(4)
Z	2
Z'	1
Wavelength/Å	1.54184
Radiation type	Cu$K\alpha$
Θ_{min}/°	2.431
Θ_{max}/°	74.637
Measured Refl's.	26888
Indep't Refl's	6453
Refl's I≥2σ(I)	5785
R_{int}	0.0236
Parameters	461
Restraints	210
Largest Peak/e Å^{-3}	0.633
Deepest Hole/e Å^{-3}	-0.228
GooF	1.056
wR_2 (all data)	0.1442
wR_2	0.1392
R_1 (all data)	0.0557
R_1	0.0500
CCDC number	2351174

Structure Quality Indicators

**Reflections:**	d min (CuKα) 2Θ=149.3° 0.80	I/σ(I) 41.7	Rint m=4.17 2.36%	Full 135.4° 98% to 149.3° 99.9
**Refinement:**	Shift 0.000	Max Peak 0.6	Min Peak -0.2	GooF 1.056

A colourless block-shaped crystal with dimensions 0.14 × 0.07 × 0.04 mm^3 was mounted. Data were collected using an XtaLAB Synergy R, DW system, HyPix-Arc 150 diffractometer operating at T = 139.99(10) K.

Data were measured using ω scans with Cu K_α radiation. The diffraction pattern was indexed and the total number of runs and images was based on the strategy calculation from the program CrysAlisPro system (CCD 43.95a 64-bit (release 03-11-2023)). The maximum resolution achieved was Θ = 74.637° (0.80 Å).

The unit cell was refined using CrysAlisPro 1.171.43.95a (Rigaku OD, 2023) on 13473 reflections, 50% of the observed reflections.

Data reduction, scaling and absorption corrections were performed using CrysAlisPro 1.171.43.95a (Rigaku OD, 2023). The final completeness is 99.90 % out to 74.637° in Θ. A Gaussian absorption correction was performed using CrysAlisPro 1.171.43.95a (Rigaku Oxford Diffraction, 2023) Numerical absorption correction based on Gaussian integration over a multifaceted crystal model. Empirical absorption correction using spherical harmonics as implemented in SCALE3 ABSPACK scaling algorithm. The absorption coefficient μ of this material is 0.678 mm^{-1} at this wavelength (λ = 1.54184Å) and the minimum and maximum transmissions are 0.878 and 1.000.

The structure was solved in the space group $P2_1$ (# 4) by the ShelXT 2018/2 (Sheldrick, 2015) structure solution program using dual methods and refined by full matrix least squares minimisation on $\boldsymbol{F}^2$ using version 2019/3 of ShelXL 2019/3 (Sheldrick, 2015). All non-hydrogen atoms were refined anisotropically. Hydrogen atom positions were calculated geometrically and refined using the riding model.

_refine_special_details: Refined as a 2-component inversion twin.

There is a single formula unit in the asymmetric unit, which is represented by the reported sum formula. In other words: Z is 2 and Z' is 1. The moiety formula is C31 H32 N4 O3, C3 H6 O1, 1[C3H6O].

The Flack parameter was refined to 0.6(3). Determination of absolute structure using Bayesian statistics on Bijvoet differences using the Olex2 results in None. The chiral atoms in this structure are: C1(S), C4(S), C6(S), C18(R). Note: The Flack parameter is used to determine chirality of the crystal studied, the value should be near 0, a value of 1 means that the stereochemistry is wrong, and the model should be inverted. A value of 0.5 means that the crystal consists of a racemic mixture of the two enantiomers.

Citations

CrysAlis^{Pro} Software System, Rigaku Oxford Diffraction, (2023).

Sheldrick, G.M., ShelXT-Integrated space-group and crystal-structure determination, *Acta Cryst.*, (2015), **A71**, 3-8.

Sheldrick, G.M., Crystal structure refinement with ShelXL, *Acta Cryst.*, (2015), **C71**, 3-8.

O.V. Dolomanov and L.J. Bourhis and R.J. Gildea and J.A.K. Howard and H. Puschmann, **Olex2**: A complete structure solution, refinement and analysis program, *J. Appl. Cryst.*, (2009), **42**, 339-341.

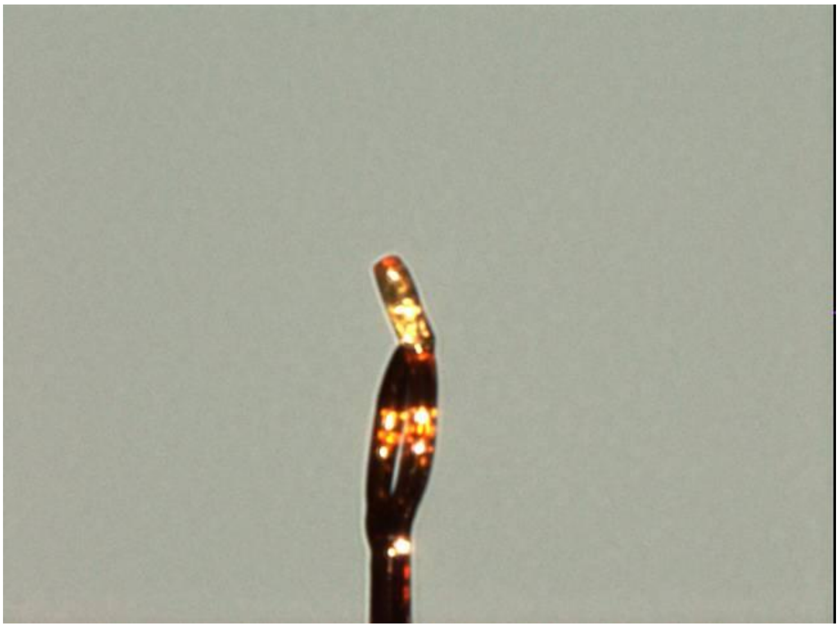

Figure 3 Image of the Crystal on the Diffractometer.

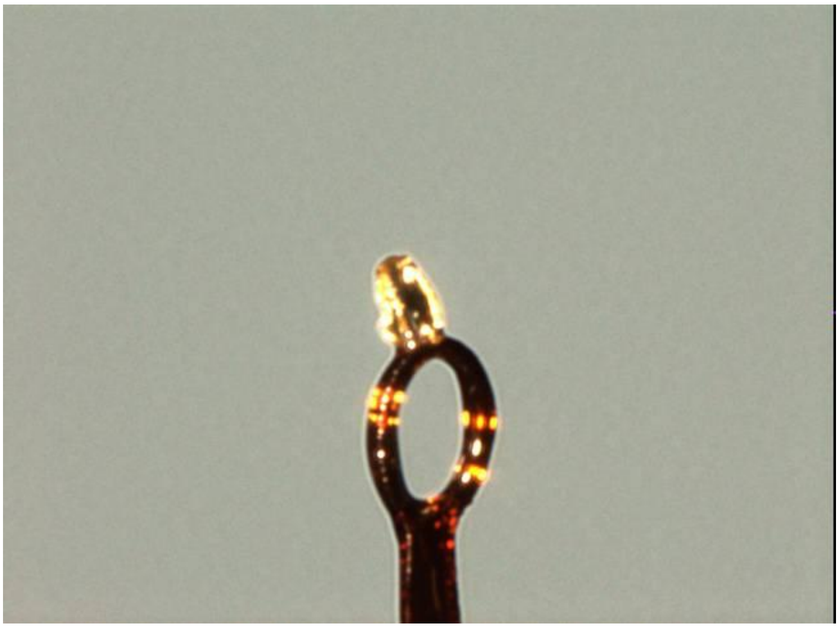

Figure 4 Image of the Crystal on the Diffractometer.

Data Plots: Diffraction Data

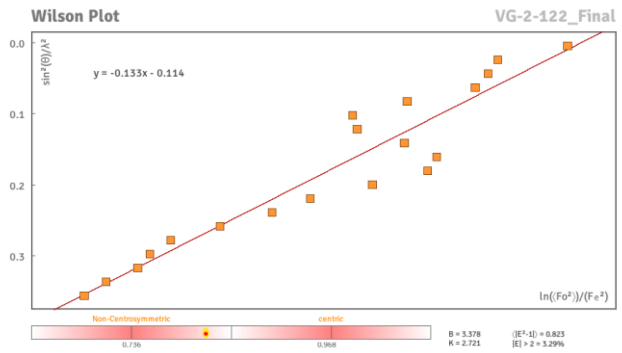

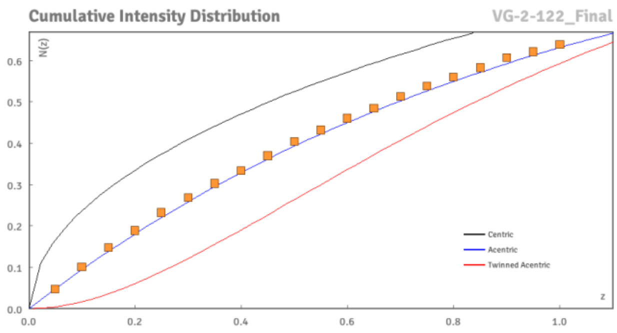

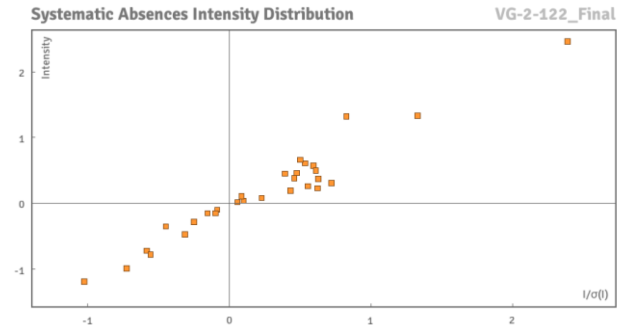

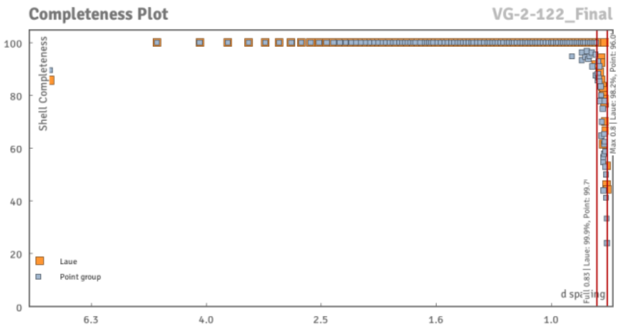

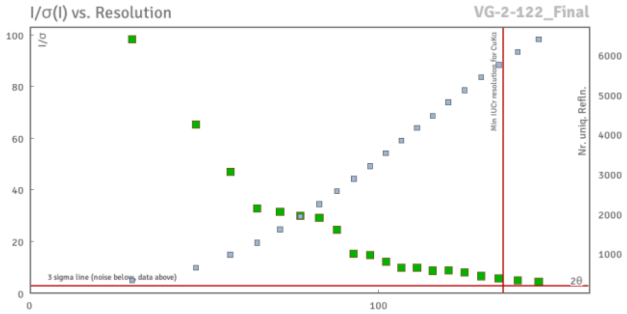

Data Plots: Refinement and Data

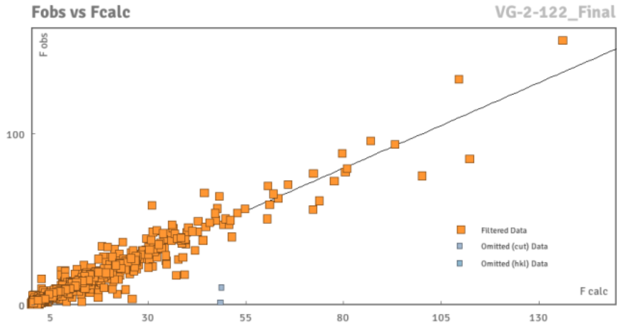

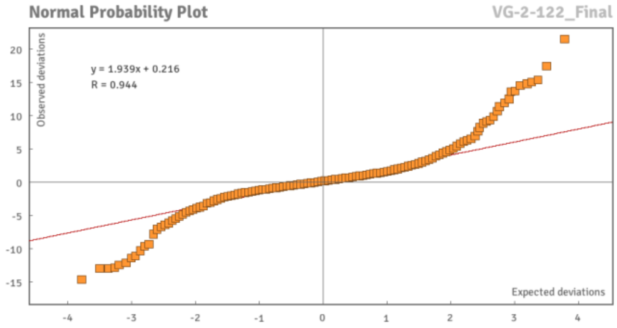

Reflection Statistics

Total reflections (after filtering)	26920	Unique reflections	6453
Completeness	0.96	Mean I/σ	24.16

hkl_{max} collected	(13, 10, 22)	hkl_{min} collected	(-13, -9, -19)
hkl_{max} used	(13, 10, 22)	hkl_{min} used	(-13, -9, 0)
Lim d_{max} collected	100.0	Lim d_{min} collected	0.77
d_{max} used	18.18	d_{min} used	0.8
Friedel pairs	3528	Friedel pairs merged	0
Inconsistent equivalents	1	R_{int}	0.0236
R_{sigma}	0.024	Intensity transformed	0
Omitted reflections	30	Omitted by user (OMIT hkl)	0
Multiplicity	(2362, 3001, 1891, 964, 475, 252, 154, 99, 68, 64, 67, 43, 21, 18, 8, 6, 1, 1, 1)	Maximum multiplicity	19
Removed systematic absences	32	Filtered off (Shel/OMIT)	0

Table 9: Fractional Atomic Coordinates (×10⁴) and Equivalent Isotropic Displacement Parameters (Å²×10³) for **vg-2-122**. U_{eq} is defined as 1/3 of the trace of the orthogonalised U_{ij}.

Atom	x	y	z	U_{eq}
O1	4400(2)	8952.9	8720.0(12)	42.8(5)
O2A	2150(9)	9593(11)	7408(5)	73(2)
O2B	1474(14)	7265(15)	8129(9)	56(2)
O3A	1655(11)	7639(13)	8196(8)	62.1(19)
O3B	2125(11)	9677(14)	7717(6)	63.1(19)
O4A	1518(11)	8294(14)	5396(7)	102(3)
O4B	1740(17)	8660(20)	5148(11)	110(4)
N1	4656(2)	6213(4)	8806.0(13)	34.1(5)
N2	2535(3)	5243(5)	4969.0(14)	45.5(7)
N3	4268(3)	3132(4)	6471.0(13)	39.2(6)
N4	5799(2)	1917(4)	8669.9(12)	31.8(5)
C1	4496(3)	4508(4)	8515.0(15)	30.6(6)
C2	4245(3)	7579(4)	8458.9(16)	33.5(6)
C3	3553(3)	7441(4)	7714.8(17)	37.3(7)
C4	3403(3)	5719(4)	7390.1(15)	32.4(6)
C5	3270(3)	5792(5)	6551.1(16)	37.3(7)
C6	3192(3)	4085(5)	6221.7(16)	37.2(6)
C7	3115(3)	4071(5)	5400.1(17)	41.2(7)
C8	2695(3)	4853(6)	4239.5(17)	47.9(8)
C9	2323(4)	5694(7)	3601.8(19)	62.0(11)
C10	2600(4)	4998(8)	2929.5(19)	65.3(12)
C11	3233(4)	3524(7)	2910.0(18)	61.6(11)
C12	3606(3)	2704(7)	3541.5(18)	55.6(10)
C13	3356(3)	3384(6)	4221.4(17)	46.7(8)
C14	3637(3)	2925(5)	4973.9(17)	44.1(8)
C15	4399(4)	1582(6)	5304.8(18)	54.8(10)
C16	4241(4)	1484(5)	6130.7(17)	47.4(8)
C17	4321(3)	2939(4)	7274.7(16)	37.8(7)
C18	4442(3)	4609(4)	7655.0(15)	30.4(6)
C19	5604(3)	3572(4)	8812.2(14)	30.7(6)
C20	6866(3)	1454(4)	9042.9(16)	33.0(6)
C21	7419(3)	-83(5)	9084.0(16)	38.5(7)
C22	8477(3)	-206(5)	9521(2)	47.8(8)
C23	8962(3)	1148(5)	9911(2)	56.7(10)
C24	8409(3)	2662(5)	9863(2)	51.0(8)
C25	7345(3)	2835(4)	9421.6(17)	36.7(6)
C26	6527(3)	4149(4)	9266.5(17)	36.8(6)
C27	6505(4)	5832(5)	9596(2)	47.4(8)
C28	5208(3)	6395(4)	9554.9(16)	43.1(8)
C29	3355(3)	3752(5)	8823.4(16)	36.8(6)
C30A	2401(11)	8374(17)	7758(11)	57(2)

Atom	x	y	z	U_{eq}
C30B	2282(16)	8120(30)	7832(17)	60(2)
C31A	474(7)	8300(12)	8209(5)	79(2)
C31B	918(11)	10336(16)	7886(8)	85(3)
C32A	1215(10)	9667(14)	5155(7)	89(2)
C32B	927(12)	9246(17)	5576(9)	86(3)
C33A	718(10)	10766(14)	5702(6)	68(2)
C33B	183(13)	8300(20)	6075(9)	104(4)
C34A	1043(14)	10201(19)	4417(8)	146(4)
C34B	650(20)	11070(20)	5415(12)	97(4)

Table 10: Anisotropic Displacement Parameters (×10[4]) for **vg-2-122**. The anisotropic displacement factor exponent takes the form: $-2\pi^2[h^2a^{*2} \times U_{11}+ ... +2hka^* \times b^* \times U_{12}]$

Atom	U_{11}	U_{22}	U_{33}	U_{23}	U_{13}	U_{12}
O1	54.5(13)	25.1(10)	48.1(13)	-3.6(9)	-4.1(10)	0.6(9)
O2A	77(3)	50(3)	92(5)	12(4)	-2(4)	26(3)
O2B	55(5)	48(5)	66(5)	-17(4)	10(4)	6(4)
O3A	61(4)	55(4)	70(3)	-16(3)	-1(3)	16(3)
O3B	66(3)	43(3)	80(4)	-14(4)	-7(4)	19(3)
O4A	93(5)	80(5)	133(7)	-28(4)	-6(4)	35(5)
O4B	102(6)	78(7)	149(8)	-22(6)	7(6)	-6(5)
N1	47.6(14)	24.3(12)	30.2(12)	-5.5(9)	1.2(10)	1.0(10)
N2	48.4(15)	59.7(18)	28.5(13)	2.7(12)	4.0(11)	17.5(14)
N3	54.0(15)	37.6(14)	26.0(12)	-3.8(10)	3.0(10)	12.2(12)
N4	48.2(14)	22.1(11)	24.7(11)	-2.6(9)	-0.6(10)	1.1(10)
C1	41.5(15)	23.7(13)	26.7(13)	-0.3(10)	3.6(11)	0.6(11)
C2	38.5(15)	22.9(13)	39.3(15)	-2.9(12)	4.9(12)	0.4(11)
C3	47.4(17)	26.2(15)	38.0(16)	0.0(12)	0.3(13)	4.4(13)
C4	38.0(15)	30.7(14)	28.6(13)	-0.1(11)	3.4(11)	3.6(11)
C5	44.6(16)	36.1(16)	31.3(15)	0.6(12)	3.1(12)	8.5(13)
C6	43.6(16)	37.7(16)	30.3(14)	-0.6(12)	2.1(12)	5.4(13)
C7	40.0(16)	52.2(19)	31.8(15)	-2.9(14)	4.1(12)	9.3(15)
C8	44.5(17)	68(2)	31.5(16)	-1.7(16)	2.4(13)	12.3(17)
C9	62(2)	92(3)	31.7(17)	6.5(18)	2.6(16)	22(2)
C10	60(2)	107(4)	28.3(16)	7(2)	1.4(15)	12(2)
C11	54(2)	103(4)	27.1(16)	-6.2(19)	2.6(14)	3(2)
C12	50.6(19)	83(3)	33.3(16)	-9.2(18)	3.6(14)	10(2)
C13	40.7(16)	68(2)	32.1(16)	-2.8(15)	3.2(12)	5.1(16)
C14	47.8(17)	55(2)	28.9(15)	-7.1(14)	1.5(12)	8.4(15)
C15	72(2)	58(2)	33.2(16)	-10.8(16)	-1.0(16)	19(2)
C16	69(2)	40.1(18)	33.0(16)	-11.6(13)	-1.0(15)	14.5(16)
C17	55.1(18)	30.2(15)	28.0(14)	-1.8(11)	2.3(12)	4.2(13)
C18	40.2(14)	26.1(13)	25.1(13)	0.1(11)	4.0(10)	1.7(11)
C19	42.6(15)	24.9(14)	25.0(13)	-0.5(10)	6.0(11)	-1.0(11)
C20	43.0(15)	26.4(14)	29.9(13)	-0.4(11)	5.1(11)	2.0(12)
C21	51.9(17)	29.2(15)	34.7(15)	-0.7(12)	5.4(13)	1.5(13)
C22	53(2)	34.1(17)	55(2)	3.7(15)	-0.9(15)	8.1(15)
C23	46.3(19)	41.9(19)	80(3)	4.4(18)	-15.4(18)	2.1(16)
C24	47.5(18)	36.7(17)	67(2)	-2.7(17)	-14.2(16)	-1.9(15)
C25	41.0(15)	29.2(15)	39.8(16)	1.2(12)	1.1(12)	-1.5(12)
C26	46.6(16)	26.0(14)	37.5(15)	-1.4(11)	-1.2(12)	0.1(12)
C27	64(2)	27.2(15)	49.0(19)	-7.0(13)	-16.8(16)	1.4(14)
C28	67(2)	30.6(15)	30.8(15)	-10.0(12)	-5.5(14)	8.6(15)
C29	46.4(16)	34.3(15)	30.4(14)	4.1(12)	8.8(12)	-1.9(13)
C30A	61(3)	40(4)	68(4)	-17(3)	-15(3)	12(3)

Atom	U_{11}	U_{22}	U_{33}	U_{23}	U_{13}	U_{12}
C30B	63(4)	47(4)	70(4)	-14(4)	-13(3)	14(4)
C31A	63(4)	80(5)	96(5)	-6(4)	25(4)	20(4)
C31B	78(6)	65(5)	114(7)	-1(5)	15(5)	25(5)
C32A	75(4)	72(5)	119(6)	-22(5)	5(4)	-10(4)
C32B	66(5)	75(5)	115(7)	-18(5)	-7(5)	5(4)
C33A	44(3)	72(5)	86(6)	-1(4)	-9(4)	10(3)
C33B	85(7)	97(8)	129(9)	0(7)	-9(7)	26(7)
C34A	160(8)	122(8)	158(9)	-16(7)	34(8)	-50(7)
C34B	77(6)	84(7)	129(9)	-25(7)	-1(8)	6(6)

Table 11: Bond Lengths in Å for **vg-2-122**.

Atom	Atom	Length/Å
O1	C2	1.226(4)
O2A	C30A	1.206(16)
O2B	C30B	1.28(2)
O3A	C30A	1.321(18)
O3A	C31A	1.415(13)
O3B	C30B	1.30(2)
O3B	C31B	1.488(15)
O4A	C32A	1.243(15)
O4B	C32B	1.310(19)
N1	C1	1.496(4)
N1	C2	1.348(4)
N1	C28	1.472(4)
N2	C7	1.375(4)
N2	C8	1.385(4)
N3	C6	1.475(4)
N3	C16	1.480(4)
N3	C17	1.469(4)
N4	C19	1.394(3)
N4	C20	1.384(4)
C1	C18	1.565(4)
C1	C19	1.520(4)
C1	C29	1.537(4)
C2	C3	1.526(4)
C3	C4	1.530(4)
C3	C30A	1.491(11)
C3	C30B	1.537(15)
C4	C5	1.528(4)
C4	C18	1.522(4)
C5	C6	1.517(4)
C6	C7	1.493(4)
C7	C14	1.362(5)
C8	C9	1.392(5)
C8	C13	1.406(5)
C9	C10	1.397(6)
C10	C11	1.393(7)
C11	C12	1.375(6)
C12	C13	1.397(5)
C13	C14	1.438(4)
C14	C15	1.491(5)
C15	C16	1.524(5)
C17	C18	1.531(4)
C19	C26	1.364(4)
C20	C21	1.396(4)
C20	C25	1.411(4)
C21	C22	1.384(5)
C22	C23	1.405(6)
C23	C24	1.379(5)
C24	C25	1.398(4)
C25	C26	1.422(4)
C26	C27	1.499(4)
C27	C28	1.506(5)
C32A	C33A	1.469(15)
C32A	C34A	1.415(15)
C32B	C33B	1.473(18)
C32B	C34B	1.55(2)

Table 12: Bond Angles in ° for **vg-2-122**.

Atom	Atom	Atom	Angle/°
C30A	O3A	C31A	116.3(10)
C30B	O3B	C31B	115.8(13)
C2	N1	C1	124.9(2)
C2	N1	C28	117.7(2)
C28	N1	C1	117.2(2)
C7	N2	C8	108.0(3)
C6	N3	C16	110.6(3)
C17	N3	C6	110.7(2)
C17	N3	C16	108.6(2)
C20	N4	C19	108.0(2)
N1	C1	C18	107.6(2)
N1	C1	C19	105.2(2)
N1	C1	C29	109.2(2)
C19	C1	C18	111.8(2)
C19	C1	C29	109.3(2)
C29	C1	C18	113.3(2)
O1	C2	N1	122.5(3)
O1	C2	C3	117.7(3)
N1	C2	C3	119.8(3)
C2	C3	C4	116.8(2)
C2	C3	C30B	105.8(12)
C4	C3	C30B	107.6(9)

Atom	Atom	Atom	Angle/˚
C30A	C3	C2	107.9(8)
C30A	C3	C4	114.5(7)
C5	C4	C3	110.7(2)
C18	C4	C3	111.0(2)
C18	C4	C5	111.8(2)
C6	C5	C4	111.1(2)
N3	C6	C5	109.5(2)
N3	C6	C7	107.9(2)
C7	C6	C5	113.7(3)
N2	C7	C6	124.5(3)
C14	C7	N2	110.6(3)
C14	C7	C6	124.9(3)
N2	C8	C9	129.7(4)
N2	C8	C13	108.1(3)
C9	C8	C13	122.2(3)
C8	C9	C10	117.5(4)
C11	C10	C9	120.4(4)
C12	C11	C10	121.9(3)
C11	C12	C13	118.8(4)
C8	C13	C14	106.7(3)
C12	C13	C8	119.1(3)
C12	C13	C14	134.2(4)
C7	C14	C13	106.6(3)
C7	C14	C15	121.5(3)
C13	C14	C15	131.7(3)
C14	C15	C16	110.2(3)
N3	C16	C15	111.4(3)
N3	C17	C18	110.7(2)
C4	C18	C1	109.7(2)
C4	C18	C17	109.9(2)
C17	C18	C1	113.8(2)

Atom	Atom	Atom	Angle/˚
N4	C19	C1	123.4(3)
C26	C19	N4	109.2(3)
C26	C19	C1	127.4(3)
N4	C20	C21	129.2(3)
N4	C20	C25	108.1(2)
C21	C20	C25	122.6(3)
C22	C21	C20	116.8(3)
C21	C22	C23	121.6(3)
C24	C23	C22	121.0(3)
C23	C24	C25	119.0(3)
C20	C25	C26	106.6(3)
C24	C25	C20	119.0(3)
C24	C25	C26	134.4(3)
C19	C26	C25	108.0(3)
C19	C26	C27	122.1(3)
C25	C26	C27	129.4(3)
C26	C27	C28	107.2(3)
N1	C28	C27	111.4(3)
O2A	C30A	O3A	124.0(10)
O2A	C30A	C3	124.6(12)
O3A	C30A	C3	111.3(10)
O2B	C30B	O3B	120.8(13)
O2B	C30B	C3	121.9(14)
O3B	C30B	C3	116.5(15)
O4A	C32A	C33A	114.5(12)
O4A	C32A	C34A	129.3(12)
C34A	C32A	C33A	114.8(12)
O4B	C32B	C33B	126.7(14)
O4B	C32B	C34B	112.1(15)
C33B	C32B	C34B	120.5(14)

Table 13: Torsion Angles in ˚ for **vg-2-122**.

Atom	Atom	Atom	Atom	Angle/˚
O1	C2	C3	C4	178.3(3)
O1	C2	C3	C30A	-51.2(7)
O1	C2	C3	C30B	-62.0(9)
N1	C1	C18	C4	-58.4(3)
N1	C1	C18	C17	178.1(2)
N1	C1	C19	N4	179.5(2)
N1	C1	C19	C26	1.9(4)
N1	C2	C3	C4	-2.1(4)
N1	C2	C3	C30A	128.4(7)
N1	C2	C3	C30B	117.6(9)
N2	C7	C14	C13	-0.9(4)
N2	C7	C14	C15	175.6(3)
N2	C8	C9	C10	-179.3(4)
N2	C8	C13	C12	178.1(4)
N2	C8	C13	C14	-2.3(4)
N3	C6	C7	N2	-155.7(3)
N3	C6	C7	C14	22.3(5)
N3	C17	C18	C1	179.6(2)
N3	C17	C18	C4	56.2(3)
N4	C19	C26	C25	0.2(3)
N4	C19	C26	C27	-172.4(3)

Atom	Atom	Atom	Atom	Angle/˚
N4	C20	C21	C22	-178.1(3)
N4	C20	C25	C24	177.8(3)
N4	C20	C25	C26	0.2(3)
C1	N1	C2	O1	-179.9(3)
C1	N1	C2	C3	0.5(4)
C1	N1	C28	C27	65.9(4)
C1	C19	C26	C25	178.1(3)
C1	C19	C26	C27	5.5(5)
C2	N1	C1	C18	29.6(4)
C2	N1	C1	C19	148.9(3)
C2	N1	C1	C29	-93.8(3)
C2	N1	C28	C27	-119.3(3)
C2	C3	C4	C5	-152.9(3)
C2	C3	C4	C18	-28.0(4)
C2	C3	C30A	O2A	114.5(18)
C2	C3	C30A	O3A	-69.4(13)
C2	C3	C30B	O2B	-80(2)
C2	C3	C30B	O3B	90(2)
C3	C4	C5	C6	176.8(3)
C3	C4	C18	C1	58.7(3)
C3	C4	C18	C17	-175.5(2)

Atom	Atom	Atom	Atom	Angle/˚
C4	C3	C30A	O2A	-113.7(16)
C4	C3	C30A	O3A	62.4(15)
C4	C3	C30B	O2B	46(3)
C4	C3	C30B	O3B	-144.1(19)
C4	C5	C6	N3	-56.7(3)
C4	C5	C6	C7	-177.5(3)
C5	C4	C18	C1	-177.1(2)
C5	C4	C18	C17	-51.4(3)
C5	C6	C7	N2	-34.0(5)
C5	C6	C7	C14	144.1(3)
C6	N3	C16	C15	67.9(4)
C6	N3	C17	C18	-62.4(3)
C6	C7	C14	C13	-179.1(3)
C6	C7	C14	C15	-2.7(6)
C7	N2	C8	C9	-177.2(4)
C7	N2	C8	C13	1.8(4)
C7	C14	C15	C16	13.0(5)
C8	N2	C7	C6	177.7(3)
C8	N2	C7	C14	-0.6(4)
C8	C9	C10	C11	-0.4(7)
C8	C13	C14	C7	1.9(4)
C8	C13	C14	C15	-174.0(4)
C9	C8	C13	C12	-2.8(6)
C9	C8	C13	C14	176.8(4)
C9	C10	C11	C12	0.0(7)
C10	C11	C12	C13	-0.9(6)
C11	C12	C13	C8	2.2(6)
C11	C12	C13	C14	-177.2(4)
C12	C13	C14	C7	-178.6(4)
C12	C13	C14	C15	5.5(8)
C13	C8	C9	C10	1.9(6)
C13	C14	C15	C16	-171.5(4)
C14	C15	C16	N3	-44.2(4)
C16	N3	C6	C5	-177.5(3)
C16	N3	C6	C7	-53.2(4)
C16	N3	C17	C18	176.0(3)
C17	N3	C6	C5	62.2(3)
C17	N3	C6	C7	-173.6(3)
C17	N3	C16	C15	-170.5(3)
C18	C1	C19	N4	-64.0(3)
C18	C1	C19	C26	118.4(3)
C18	C4	C5	C6	52.4(3)
C19	N4	C20	C21	178.3(3)
C19	N4	C20	C25	-0.1(3)
C19	C1	C18	C4	-173.5(2)
C19	C1	C18	C17	63.0(3)
C19	C26	C27	C28	19.4(4)
C20	N4	C19	C1	-178.0(2)
C20	N4	C19	C26	-0.1(3)
C20	C21	C22	C23	0.8(5)
C20	C25	C26	C19	-0.3(3)
C20	C25	C26	C27	171.6(3)
C21	C20	C25	C24	-0.8(5)
C21	C20	C25	C26	-178.3(3)
C21	C22	C23	C24	-1.1(6)
C22	C23	C24	C25	0.4(6)
C23	C24	C25	C20	0.5(5)
C23	C24	C25	C26	177.2(4)
C24	C25	C26	C19	-177.3(4)
C24	C25	C26	C27	-5.4(6)
C25	C20	C21	C22	0.1(4)
C25	C26	C27	C28	-151.4(3)
C26	C27	C28	N1	-51.4(4)
C28	N1	C1	C18	-156.1(3)
C28	N1	C1	C19	-36.7(3)
C28	N1	C1	C29	80.5(3)
C28	N1	C2	O1	5.7(4)
C28	N1	C2	C3	-173.9(3)
C29	C1	C18	C4	62.4(3)
C29	C1	C18	C17	-61.1(3)
C29	C1	C19	N4	62.4(3)
C29	C1	C19	C26	-115.2(3)
C30A	C3	C4	C5	79.7(9)
C30A	C3	C4	C18	-155.4(8)
C30B	C3	C4	C5	88.5(12)
C30B	C3	C4	C18	-146.7(12)
C31A	O3A	C30A	O2A	4(2)
C31A	O3A	C30A	C3	-172.3(11)
C31B	O3B	C30B	O2B	-5(3)
C31B	O3B	C30B	C3	-175.6(16)

Table 14: Hydrogen Fractional Atomic Coordinates (×10[4]) and Equivalent Isotropic Displacement Parameters (Å[2]×10[3]) for **vg-2-122.** U_{eq} is defined as 1/3 of the trace of the orthogonalised U_{ij}.

Atom	x	y	z	U_{eq}
H2	2132.47	6090.47	5128.41	55
H4	5325.8	1278.59	8391.7	38
H3A	4042.52	8061.72	7361.62	45
H3B	3953.55	8153.37	7352.55	45
H4A	2639.19	5246.29	7570.6	39
H5A	2530.27	6414.58	6400.51	45
H5B	3972.38	6375.57	6360.22	45
H6	2457.77	3529.85	6400.98	45
H9	1897.03	6702.06	3622.81	74
H10	2355.8	5533.86	2482.77	78

Atom	x	y	z	U_{eq}
H11	3411.64	3072.83	2446.85	74
H12	4026.54	1692.25	3515.8	67
H15A	5259.47	1788.23	5212.79	66
H15B	4162.56	525.03	5071.83	66
H16A	3460.52	947.72	6220.58	57
H16B	4896.71	805.26	6362.83	57
H17A	5021.75	2242.73	7428.77	45
H17B	3577.53	2386.28	7425.74	45
H18	5211.63	5121.95	7505.32	36
H21	7085.83	-1001.2	8824.59	46
H22	8884.08	-1229.27	9557.11	57
H23	9681.83	1018.54	10212.68	68
H24	8746.01	3573.13	10126.5	61
H27A	7010.35	6589.13	9317.83	57
H27B	6823.96	5802.67	10113.76	57
H28A	4742.05	5744.65	9901.88	52
H28B	5171.83	7559.54	9704.94	52
H29A	2645.38	4397.17	8658.78	55
H29B	3433.26	3752.46	9362.2	55
H29C	3259.81	2622.85	8645.69	55
H31A	62.24	8177.64	7722.43	119
H31B	523.64	9464.75	8338.27	119
H31C	18.7	7715.31	8575.21	119
H31D	283.84	9612.03	7676.11	128
H31E	817.78	11434.47	7673.92	128
H31F	856.18	10397.14	8421.02	128
H33A	-123.27	11032.9	5555.11	102
H33B	1197.02	11776.17	5734.45	102
H33C	748.51	10223.54	6183.41	102
H33D	-77.29	7278.2	5835.77	156
H33E	-528.55	8949.77	6190.93	156
H33F	661.37	8057.62	6529.61	156
H34A	1806.36	10108.01	4170.3	219
H34B	776.52	11346.92	4408.85	219
H34C	426.55	9519.26	4160.12	219
H34D	479.17	11632.5	5874.51	145
H34E	-59.57	11154.39	5070.09	145
H34F	1346.22	11583.85	5197.87	145

Table 15: Atomic Occupancies for all atoms that are not fully occupied in **vg-2-122**.

Atom	Occupancy	Atom	Occupancy	Atom	Occupancy
O2A	0.573(9)	H31C	0.573(9)	H33E	0.415(8)
O2B	0.427(9)	C31B	0.427(9)	H33F	0.415(8)
O3A	0.573(9)	H31D	0.427(9)	C34A	0.585(8)
O3B	0.427(9)	H31E	0.427(9)	H34A	0.585(8)
O4A	0.585(8)	H31F	0.427(9)	H34B	0.585(8)
O4B	0.415(8)	C32A	0.585(8)	H34C	0.585(8)
H3A	0.573(9)	C32B	0.415(8)	C34B	0.415(8)
H3B	0.427(9)	C33A	0.585(8)	H34D	0.415(8)
C30A	0.573(9)	H33A	0.585(8)	H34E	0.415(8)
C30B	0.427(9)	H33B	0.585(8)	H34F	0.415(8)
C31A	0.573(9)	H33C	0.585(8)		
H31A	0.573(9)	C33B	0.415(8)		
H31B	0.573(9)	H33D	0.415(8)		

Table 16: Solvent masking (PLATON/SQUEEZE) information for **vg-2-122**.

No	x	y	z	V	e	Content
1	0.098	-0.064	0.221	106.6	31.4	1acetone
2	-0.098	0.436	0.779	106.6	31.3	1acetone

X-ray crystallographic data of **1** (CCDC 2350841): **VG-2-162-2.**

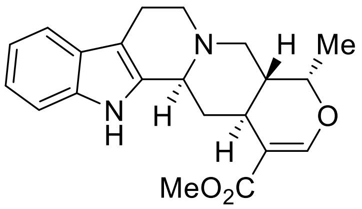

1 (-)-Ajmalicine

R_1=4.27%

Solved by: **Rosario Scopelliti**

Crystal Data and Experimental

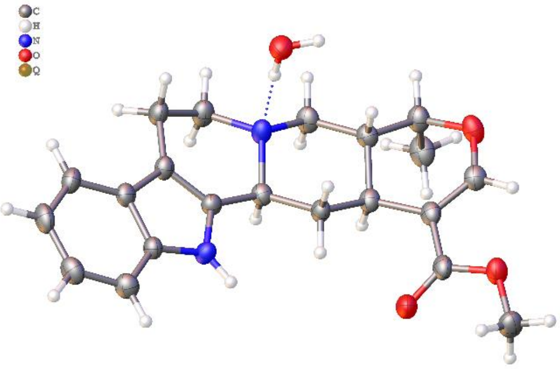

*X-Ray tructure of **1** with a 50% ellipsoid probability level*

Experimental. Single clear pale colourless plate-shaped crystals of **vg-2-162-2** were used as supplied. A suitable crystal with dimensions 0.24 × 0.16 × 0.04 mm³ was selected and mounted on a XtaLAB Synergy R, DW system, HyPix-Arc 150 diffractometer. The crystal was kept at a steady T = 139.99(10) K during data collection. The structure was solved with the ShelXT (Sheldrick, 2015) solution program using dual methods and by using Olex2 1.5 (Dolomanov et al., 2009) as the graphical interface. The model was refined with ShelXL 2019/3 (Sheldrick, 2015) using full matrix least squares minimisation on $\boldsymbol{F}^2$.

Crystal Data. $C_{21}H_{26}N_2O_4$, M_r = 370.44, monoclinic, $P2_1$ (No. 4), a = 6.8621(3) Å, b = 8.3813(3) Å, c = 16.5368(7) Å, β = 101.363(4)°, α = γ = 90°, V = 932.44(7) Å³, T = 139.99(10) K, Z = 2, Z' = 1, μ(Cu K_α) = 0.744, 11651 reflections measured, 3550 unique (R_{int} = 0.0403) which were used in all calculations. The final wR_2 was 0.1103 (all data) and R_1 was 0.0427 (I≥2 σ(I)).

**Compound**	**vg-2-162-2**
Formula	$C_{21}H_{26}N_2O_4$
$D_{calc.}$/ g cm^{-3}	1.319
μ/mm^{-1}	0.744
Formula Weight	370.44
Colour	clear pale colourless
Shape	plate
Size/mm³	0.24×0.16×0.04
T/K	139.99(10)
Crystal System	monoclinic
Flack Parameter	-0.09(16)
Hooft Parameter	-0.09(16)
Space Group	$P2_1$
a/Å	6.8621(3)
b/Å	8.3813(3)
c/Å	16.5368(7)
α/°	90
β/°	101.363(4)
γ/°	90
V/Å³	932.44(7)
Z	2
Z'	1
Wavelength/Å	1.54184
Radiation type	Cu K_α
Θ_{min}/°	2.725
Θ_{max}/°	74.508
Measured Refl's.	11651
Indep't Refl's	3550
Refl's I≥2 σ(I)	2905
R_{int}	0.0403
Parameters	259
Restraints	4
Largest Peak	0.187
Deepest Hole	-0.189
GooF	1.022
wR_2 (all data)	0.1103
wR_2	0.1017
R_1 (all data)	0.0573
R_1	0.0427

Structure Quality Indicators

**Reflections:**	d min (CuKα) 2Θ=149.0° 0.80	I/σ(I) 21.5	Rint m=3.29 4.03%	Full 135.4° 95% to 149.0° 99.8	
**Refinement:**	Shift 0.000	Max Peak 0.2	Min Peak -0.2	GooF 1.022	Hooft -.09(16)

A clear pale colourless plate-shaped crystal with dimensions 0.24 × 0.16 × 0.04 mm^3 was mounted. Data were collected using a XtaLAB Synergy R, DW system, HyPix-Arc 150 diffractometer operating at T = 139.99(10) K.

Data were measured using ω scans with Cu K_α radiation. The diffraction pattern was indexed and the total number of runs and images was based on the strategy calculation from the program CrysAlisPro system (CCD 43.116a 64-bit (release 22-03-2024)). The maximum resolution that was achieved was Θ = 74.508° (0.80 Å).

The unit cell was refined using CrysAlisPro 1.171.43.118a (Rigaku OD, 2024) on 5357 reflections, 46% of the observed reflections.

Data reduction, scaling and absorption corrections were performed using CrysAlisPro 1.171.43.118a (Rigaku OD, 2024). The final completeness is 99.90 % out to 74.508° in Θ. An analytical absorption correction was performed using CrysAlisPro 1.171.43.118a (Rigaku Oxford Diffraction, 2024). The analytical numeric absorption correction was done using a multifaceted crystal model based on expressions derived by R.C. Clark & J.S. Reid. (Clark, R. C. & Reid, J. S. (1995). Acta Cryst. A51, 887-897). The empirical absorption correction was carried out using spherical harmonics, implemented in SCALE3 ABSPACK scaling algorithm. The absorption coefficient μ of this crystal is 0.744 mm^{-1} at this wavelength (λ = 1.54184Å) and the minimum and maximum transmissions are 0.880 and 0.971.

The structure was solved and the space group $P2_1$ (# 4) determined by the ShelXT (Sheldrick, 2015) structure solution program using dual methods and refined by full matrix least squares minimisation on $\boldsymbol{F}^2$ using version 2019/3 of ShelXL 2019/3 (Sheldrick, 2015). All non-hydrogen atoms were refined anisotropically. Most hydrogen atom positions were calculated geometrically and refined using the riding model, but some hydrogen atoms were refined freely.

There is a single formula unit in the asymmetric unit, which is represented by the reported sum formula. In other words: Z is 2 and Z' is 1. The moiety formula is C21 H24 N2 O3, H2 O.

The Flack parameter was refined to -0.09(16). Determination of absolute structure using Bayesian statistics on Bijvoet differences using the Olex2 results in -0.09(16). The chiral atoms in this structure are: C3(S), C5(S), C17(R), C18(S). Note: The Flack parameter is used to determine chirality of the crystal studied, the value should be near 0, a value of 1 means that the stereochemistry is wrong and the model should be inverted. A value of 0.5 means that the crystal consists of a racemic mixture of the two enantiomers.

Data Plots: Diffraction Data

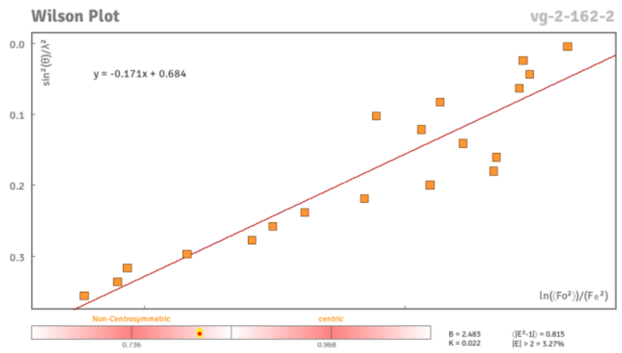

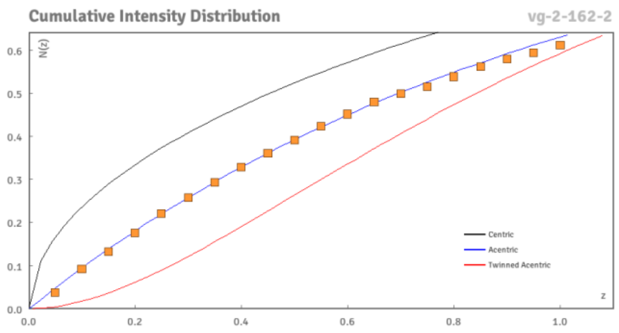

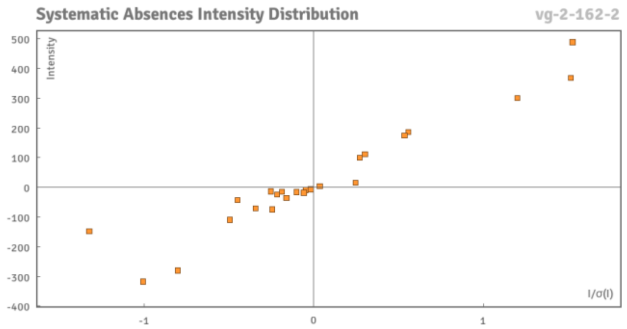

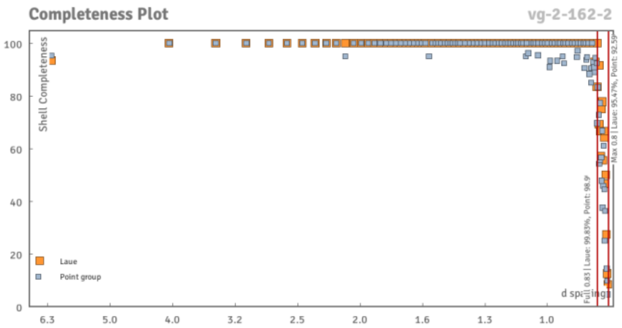

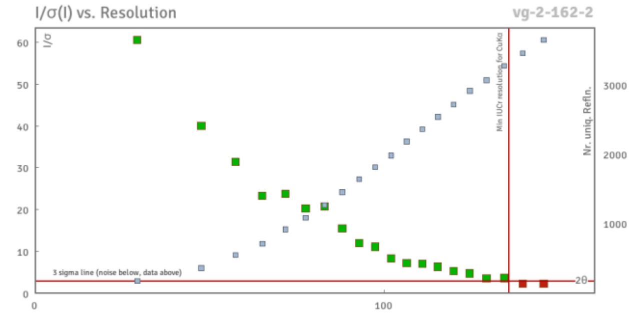

Data Plots: Refinement and Data

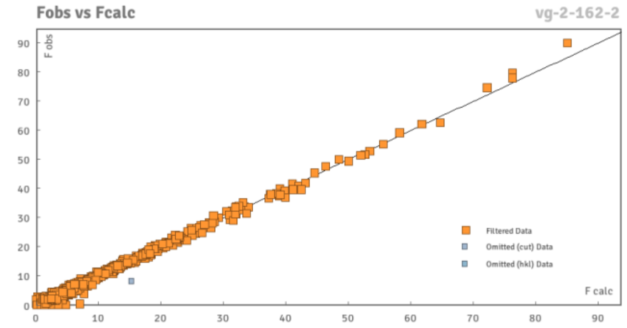

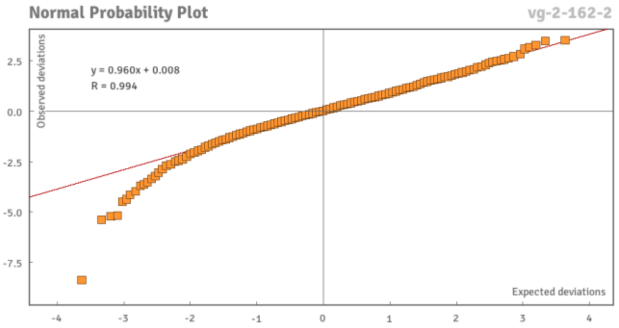

Reflection Statistics

Total reflections (after filtering)	11676	Unique reflections	3550
Completeness	0.926	Mean I/σ	16.32

hkl_{max} collected	(8, 10, 20)	hkl_{min} collected	(-8, -10, -18)
hkl_{max} used	(8, 10, 20)	hkl_{min} used	(-8, -10, 0)
Lim d_{max} collected	100.0	Lim d_{min} collected	0.77
d_{max} used	16.21	d_{min} used	0.8
Friedel pairs	1855	Friedel pairs merged	0
Inconsistent equivalents	6	R_{int}	0.0403
R_{sigma}	0.0465	Intensity transformed	0
Omitted reflections	1	Omitted by user (OMIT hkl)	0
Multiplicity	(1908, 1710, 663, 358, 185, 115, 58, 46, 28, 12, 8, 1, 4, 0, 1)	Maximum multiplicity	16
Removed systematic absences	25	Filtered off (Shel/OMIT)	0

Table 17: Fractional Atomic Coordinates (×10⁴) and Equivalent Isotropic Displacement Parameters ($Å^2×10^3$) for **vg-2-162-2.** U_{eq} is defined as 1/3 of the trace of the orthogonalised U_{ij}.

Atom	x	y	z	U_{eq}
O1	5700(4)	623(3)	4458.9(14)	35.1(6)
O2	-459(4)	3399(4)	4116.0(15)	45.3(7)
O3	1193(4)	2537(3)	5338.6(13)	35.5(6)
N1	385(4)	6880(3)	1904.5(17)	27.2(6)
N2	4608(4)	4062(3)	2041.9(16)	27.5(6)
C1	4133(5)	1342(4)	4682(2)	30.6(8)
C2	2731(5)	2213(4)	4201(2)	27.5(7)
C3	2836(5)	2531(4)	3311.7(19)	27.4(7)
C4	2303(5)	4231(4)	3011(2)	27.2(7)
C5	2526(5)	4394(4)	2115.0(19)	25.7(7)
C6	1996(5)	6014(4)	1770.3(19)	25.3(7)
C7	329(5)	8301(4)	1470.0(19)	25.5(7)
C8	-1023(5)	9555(4)	1398(2)	33.1(8)
C9	-749(5)	10818(4)	894(2)	35.1(8)
C10	840(6)	10839(4)	469(2)	36.8(8)
C11	2192(5)	9594(4)	555(2)	30.3(8)
C12	1947(5)	8308(4)	1056.2(18)	25.9(7)
C13	2987(5)	6823(4)	1257.4(19)	26.2(7)
C14	4758(5)	6145(4)	989(2)	30.9(8)
C15	4901(5)	4358(4)	1191(2)	33.3(8)
C16	5169(6)	2417(4)	2312(2)	31.9(8)
C17	4965(5)	2210(4)	3209(2)	27.8(7)
C18	5730(6)	608(4)	3578(2)	33.2(8)
C19	1027(5)	2780(4)	4534(2)	30.5(8)
C20	-478(6)	3054(5)	5688(2)	40.5(9)
C21	4592(6)	-844(4)	3200(2)	40.5(9)
O4	7628(4)	5924(3)	2933.9(15)	35.5(6)

Table 18: Anisotropic Displacement Parameters (×10⁴) for **vg-2-162-2**. The anisotropic displacement factor exponent takes the form: $-2\pi^2[h^2a^{*2} \times U_{11} + ... +2hka^* \times b^* \times U_{12}]$

Atom	U_{11}	U_{22}	U_{33}	U_{23}	U_{13}	U_{12}
O1	43.9(14)	29.1(13)	29.9(13)	2.6(11)	1.6(11)	9.4(12)
O2	34.2(15)	61.7(19)	40.3(15)	22.8(14)	8.2(12)	4.7(13)
O3	41.6(15)	37.5(15)	28.2(13)	5.6(11)	8.5(11)	7.7(12)
N1	30.2(15)	22.8(15)	30.7(15)	4.0(12)	11.2(12)	3.3(12)
N2	34.8(15)	22.4(14)	27.0(14)	2.8(12)	10.1(12)	6.9(13)
C1	37(2)	24.6(17)	30.1(18)	-1.8(14)	5.3(14)	-0.2(15)

C2	32.9(18)	19.1(16)	29.3(17)	3.4(14)	3.5(14)	-2.0(15)
C3	32.8(18)	21.5(17)	27.5(18)	0.6(14)	4.9(14)	-1.9(14)
C4	31.2(17)	23.9(17)	27.7(17)	5.5(14)	8.5(13)	3.9(15)
C5	29.2(17)	21.0(17)	26.2(17)	-0.6(13)	3.6(13)	0.4(14)
C6	26.7(17)	25.2(18)	24.1(16)	0.1(14)	5.2(13)	1.5(14)
C7	29.3(17)	22.5(16)	24.2(17)	2.9(14)	4.2(13)	1.6(14)
C8	37(2)	28.0(19)	35(2)	-0.7(15)	7.1(15)	4.5(16)
C9	41(2)	23.5(17)	37(2)	1.8(17)	-0.1(15)	6.0(17)
C10	49(2)	24.8(18)	33.2(19)	8.9(17)	-0.5(16)	0.8(18)
C11	39(2)	27.5(19)	24.8(18)	3.2(14)	7.6(14)	-3.9(16)
C12	32.8(18)	23.9(17)	20.1(16)	-1.1(13)	3.2(13)	-0.2(15)
C13	33.7(18)	21.8(17)	23.7(17)	0.4(14)	7.4(13)	1.2(14)
C14	36.1(19)	28.4(19)	29.8(18)	7.2(15)	10.6(14)	2.9(16)
C15	42(2)	30(2)	30.1(18)	1.9(16)	12.4(15)	7.4(17)
C16	40(2)	21.8(17)	34.1(19)	1.4(15)	9.0(15)	8.4(15)
C17	32.6(18)	21.2(17)	29.2(18)	-0.3(14)	4.9(14)	2.4(14)
C18	41(2)	26.7(19)	32(2)	2.2(15)	8.0(15)	6.6(16)
C19	35.2(19)	25.5(18)	29.5(18)	7.5(14)	3.5(14)	-3.0(15)
C20	42(2)	45(2)	37(2)	4.8(17)	15.3(16)	9.5(18)
C21	56(2)	23.1(19)	40(2)	-0.7(16)	4.1(17)	2.9(18)
O4	33.6(14)	38.2(15)	34.5(14)	3.1(12)	6.3(11)	-3.1(12)

Table 19: Bond Lengths in Å for **vg-2-162-2**.

Atom	Atom	Length/Å
O1	C1	1.347(4)
O1	C18	1.461(4)
O2	C19	1.228(4)
O3	C19	1.329(4)
O3	C20	1.448(4)
N1	C6	1.377(4)
N1	C7	1.387(4)
N2	C5	1.484(4)
N2	C15	1.480(4)
N2	C16	1.477(4)
C1	C2	1.338(5)
C2	C3	1.510(4)
C2	C19	1.466(5)
C3	C4	1.530(4)
C3	C17	1.528(4)
C4	C5	1.525(4)

Atom	Atom	Length/Å
C5	C6	1.489(5)
C6	C13	1.367(4)
C7	C8	1.392(5)
C7	C12	1.414(4)
C8	C9	1.383(5)
C9	C10	1.409(5)
C10	C11	1.385(5)
C11	C12	1.390(5)
C12	C13	1.441(5)
C13	C14	1.486(5)
C14	C15	1.534(5)
C16	C17	1.526(5)
C17	C18	1.525(5)
C18	C21	1.513(5)

Table 20: Bond Angles in ° for **vg-2-162-2**.

Atom	Atom	Atom	Angle/°
C1	O1	C18	116.5(3)
C19	O3	C20	115.8(3)
C6	N1	C7	108.1(3)
C15	N2	C5	111.0(2)
C16	N2	C5	110.4(2)
C16	N2	C15	111.6(3)
C2	C1	O1	126.9(3)
C1	C2	C3	120.6(3)
C1	C2	C19	119.1(3)
C19	C2	C3	120.2(3)
C2	C3	C4	115.2(3)

Atom	Atom	Atom	Angle/°
C2	C3	C17	107.9(3)
C17	C3	C4	107.5(3)
C5	C4	C3	109.6(3)
N2	C5	C4	110.2(3)
N2	C5	C6	107.8(3)
C6	C5	C4	113.0(3)
N1	C6	C5	124.2(3)
C13	C6	N1	110.6(3)
C13	C6	C5	125.2(3)
N1	C7	C8	130.0(3)
N1	C7	C12	108.0(3)

Atom	Atom	Atom	Angle/°
C8	C7	C12	122.0(3)
C9	C8	C7	117.5(3)
C8	C9	C10	121.4(3)
C11	C10	C9	120.5(3)
C10	C11	C12	119.2(3)
C7	C12	C13	106.6(3)
C11	C12	C7	119.3(3)
C11	C12	C13	134.1(3)
C6	C13	C12	106.7(3)
C6	C13	C14	122.1(3)
C12	C13	C14	131.1(3)
C13	C14	C15	109.2(3)

Atom	Atom	Atom	Angle/°
N2	C15	C14	110.8(3)
N2	C16	C17	109.4(3)
C16	C17	C3	111.2(3)
C18	C17	C3	111.8(3)
C18	C17	C16	113.5(3)
O1	C18	C17	108.7(3)
O1	C18	C21	108.2(3)
C21	C18	C17	115.7(3)
O2	C19	O3	121.7(3)
O2	C19	C2	124.1(3)
O3	C19	C2	114.1(3)

Table 21: Torsion Angles in ° for **vg-2-162-2**.

Atom	Atom	Atom	Atom	Angle/°
O1	C1	C2	C3	-2.2(6)
O1	C1	C2	C19	174.2(3)
N1	C6	C13	C12	0.4(4)
N1	C6	C13	C14	-178.3(3)
N1	C7	C8	C9	-177.8(3)
N1	C7	C12	C11	178.2(3)
N1	C7	C12	C13	0.2(4)
N2	C5	C6	N1	-164.0(3)
N2	C5	C6	C13	18.4(4)
N2	C16	C17	C3	59.4(4)
N2	C16	C17	C18	-173.6(3)
C1	O1	C18	C17	39.1(4)
C1	O1	C18	C21	-87.3(4)
C1	C2	C3	C4	-138.4(3)
C1	C2	C3	C17	-18.3(4)
C1	C2	C19	O2	-169.9(4)
C1	C2	C19	O3	8.7(5)
C2	C3	C4	C5	178.5(3)
C2	C3	C17	C16	176.9(3)
C2	C3	C17	C18	48.9(3)
C3	C2	C19	O2	6.6(5)
C3	C2	C19	O3	-174.8(3)
C3	C4	C5	N2	-60.4(3)
C3	C4	C5	C6	179.0(3)
C3	C17	C18	O1	-60.5(4)
C3	C17	C18	C21	61.4(4)
C4	C3	C17	C16	-58.2(3)
C4	C3	C17	C18	173.8(3)
C4	C5	C6	N1	-42.0(4)
C4	C5	C6	C13	140.4(3)
C5	N2	C15	C14	68.9(4)
C5	N2	C16	C17	-59.6(3)
C5	C6	C13	C12	178.3(3)
C5	C6	C13	C14	-0.4(5)

Atom	Atom	Atom	Atom	Angle/°
C6	N1	C7	C8	178.9(3)
C6	N1	C7	C12	0.1(4)
C6	C13	C14	C15	13.9(5)
C7	N1	C6	C5	-178.2(3)
C7	N1	C6	C13	-0.3(4)
C7	C8	C9	C10	-0.1(5)
C7	C12	C13	C6	-0.4(4)
C7	C12	C13	C14	178.2(3)
C8	C7	C12	C11	-0.7(5)
C8	C7	C12	C13	-178.7(3)
C8	C9	C10	C11	-0.8(5)
C9	C10	C11	C12	1.0(5)
C10	C11	C12	C7	-0.2(5)
C10	C11	C12	C13	177.2(4)
C11	C12	C13	C6	-178.0(4)
C11	C12	C13	C14	0.6(6)
C12	C7	C8	C9	0.8(5)
C12	C13	C14	C15	-164.5(3)
C13	C14	C15	N2	-46.4(4)
C15	N2	C5	C4	-174.8(3)
C15	N2	C5	C6	-51.1(3)
C15	N2	C16	C17	176.4(3)
C16	N2	C5	C4	60.9(3)
C16	N2	C5	C6	-175.4(3)
C16	N2	C15	C14	-167.5(3)
C16	C17	C18	O1	172.8(3)
C16	C17	C18	C21	-65.3(4)
C17	C3	C4	C5	58.1(3)
C18	O1	C1	C2	-8.9(5)
C19	C2	C3	C4	45.1(4)
C19	C2	C3	C17	165.2(3)
C20	O3	C19	O2	-0.3(5)
C20	O3	C19	C2	-178.9(3)

Table 22: Hydrogen Fractional Atomic Coordinates (×10[4]) and Equivalent Isotropic Displacement Parameters (Å[2]×10[3]) for **vg-2-162-2**. U_{eq} is defined as 1/3 of the trace of the orthogonalised U_{ij}.

Atom	x	y	z	U_{eq}
H1	-430(60)	6570(50)	2230(20)	33(10)
H1A	4008.79	1218.49	5240.79	37
H3	1927.68	1770.35	2953.82	33
H4A	916.5	4472.68	3056.89	33
H4B	3190.36	5000.78	3358.84	33
H5	1637.35	3595.96	1775.28	31
H8	-2094.99	9544.18	1684.44	40
H9	-1651.27	11690.05	833.47	42
H10	984.72	11714.6	120.86	44
H11	3273.46	9618.81	274.39	36
H14A	5973.86	6696.82	1277.49	37
H14B	4642.41	6304.76	388.49	37
H15A	3878.24	3772.48	795.87	40
H15B	6222.24	3953.75	1133.1	40
H16A	6558.17	2204.88	2259.77	38
H16B	4295.05	1646.39	1958.69	38
H17	5815.82	3049.4	3533.08	33
H18	7142.58	489.56	3515.02	40
H20A	-1642.63	2398.89	5464.61	61
H20B	-771.37	4175.51	5545.85	61
H20C	-150.21	2937.98	6288.93	61
H21A	5206.76	-1809.07	3471.96	61
H21B	4621.32	-889.98	2609.96	61
H21C	3211.11	-772.52	3271.74	61
H4C	8110(70)	5320(60)	3410(20)	85(18)
H4D	6550(50)	5300(50)	2630(20)	67(16)

Citations

CrysAlisPro Software System, Rigaku Oxford Diffraction, (2024).

Dolomanov, O. V.; Bourhis, L. J.; Gildea, R. J.; Howard J. A. K.; Puschmann, H. Olex2: A complete structure solution, refinement and analysis program. *J. Appl. Cryst.* **2009**, *42*, 339-341.

Sheldrick, G.M. Crystal structure refinement with ShelXL, *Acta Cryst.* **2015**, *C71*, 3-8.

Sheldrick, G.M. ShelXT-Integrated space-group and crystal-structure determination. *Acta Cryst.* **2015**, *A71*, 3-8.

Rose, H. A. Ajmalicine, Ajmalicine Hydrate, and *py*-Tetrahydroserpentinol. *Anal. Chem.* **1955**, *27*, 469-470.

Dubost, J.-P.; Léger, J.-M.; Goursolle, M.; Colleter, J.-C.; Carpy, A. Structure de la Raubasine Hydratée. *Acta Crystallogr., Sect. C: Cryst. Struct. Commun.* **1984**, *C40,* 152–154. https://doi.org/10.1107/S0108270184003383.
